# Supramolecular chemistry for optical detection and delivery applications in living plants†

**DOI:** 10.1039/d4cs00500g

**Published:** 2025-07-17

**Authors:** Maria Vittoria Balli, Frank Biedermann, Luca Prodi, Pierre Picchetti

**Affiliations:** a Department of Chemistry “Giacomo Ciamician”, Università degli Studi di Bologna, Via Selmi 2 40126 Bologna Italy luca.prodi@unibo.it; b Institute of Nanotechnology (INT) Karlsruhe Institute of Technology (KIT) Kaiserstrasse 12 76131 Karlsruhe Germany pierre.picchetti@kit.edu; c IRCCS Azienda Ospedaliero-Universitaria di Bologna, via Albertoni 15 40138 Bologna Italy

## Abstract

Over the past century, modern agriculture, through the use of synthetic fertilisers, pesticides, and improved plant breeding, has greatly increased food production. However, this progress has brought serious environmental consequences, including excessive water use and harmful pesticide exposure. In addition, future farming must adapt to the growing challenges posed by climate change and natural disasters through more sustainable practices and resilient crop management. In this context, emerging supramolecular strategies offer promising alternatives through responsive molecular assemblies capable of precise sensing and controlled delivery. In this review, we thus discuss the application of supramolecular chemistry principles to plant science and agriculture, with a particular emphasis on the design and implementation of host–guest systems, chemosensors, and supramolecular (nano)delivery vehicles for use in living plants. We report and analyse recent advances in sensing and monitoring of plant processes, the detection of pesticides, the preparation of safer and more effective supramolecular pesticides, and nucleic acid-based crop protection strategies, highlighting key design principles specific to the plant biological context. Moreover, key challenges are discussed regarding the application of supramolecular systems to plants, and examples are highlighted to promote new interdisciplinary strategies for designing next-generation tools for real-time, *in vivo* plant studies and sustainable crop management.

## Abstract 2

This review highlights the advances in the use of supramolecular (nano)systems for optical detection and delivery applications in plant sciences.

## Graphical abstract

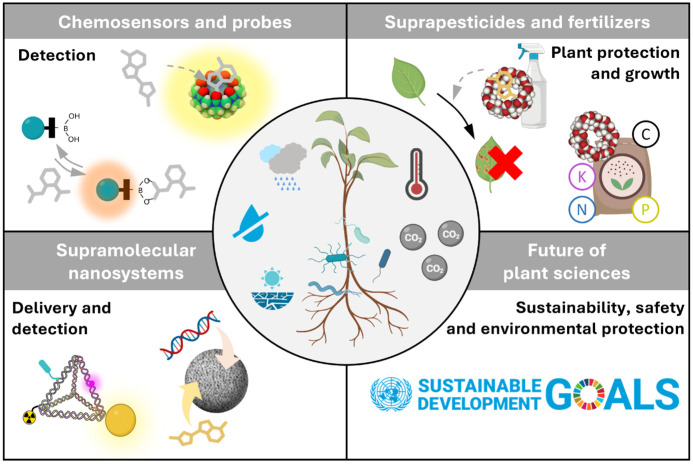

## 1. Introduction

### 1.1 Introductory remarks

Global population forecasts project that, by the year 2050, approximately 9 billion people will need food supplies,^1^ posing major challenges to current agricultural practices and the environment. Over the past century, the development of modern agriculture, including the use of synthetic fertilisers,^2,3^ pesticides, advanced farming, and plant breeding techniques,^4,5^ has led to a substantial increase in food production. However, this progress has not come without notable negative environmental consequences. Although food production has increased, unsustainable practices have led to serious environmental consequences, such as excessive and disruptive water consumption,^6^ and the overuse of pesticides, which adversely affect human health.^7–11^ For example, epidemiological studies have highlighted a strong correlation between pesticide exposure and the risk of developing Parkinson's disease.^12,13^ It has been shown that individuals who have been actively exposed to the herbicide paraquat face a 2.2-fold increased risk of developing Parkinson's disease,^12,13^ with organochlorine insecticides being identified as the specific class of pesticides most closely associated with this disorder.^14^ In addition, improper pesticide usage can indeed lead to several risks to public health, too, *e.g.*, through the residual contamination of the food chain,^15–17^ giving rise to other severe diseases, such as Hodgkin's^18^ and Alzheimer's diseases,^19,20^ as well as being involved in the pathogenesis of neoplasia, oxidative stress, and various respiratory and reproductive disorders.^21,22^ Furthermore, the presence of climate-related events, such as heatwaves, forest fires, droughts and the decline of pollinator insects, as well as glacier melting, pose an additional threat to global agriculture.^23–26^

Therefore, new sustainable and environmentally friendly agricultural practices need to be developed to mitigate the harmful effects of agriculture and climate change.^7,27^ The situation is not hopeless; recent technologies, like safer nucleic acid-based pesticides that rely on the effective delivery of messenger RNA (mRNA) or deoxyribonucleic acid (DNA), have already shown promising results in creating less harmful pesticides. However, this approach still faces challenges related to effective mRNA delivery to plants.^28,29^

Agricultural breeding practices remain a valuable option for obtaining more resistant crops. However, they cannot solve all problems, as they sometimes result in plant varieties that are more susceptible to environmental stresses. For instance, this vulnerability has resulted in the imminent extinction of widely cultivated fruit crops, like the Cavendish banana, which faces the threat of *Fusarium oxysporum*, the pathogen responsible for the Panama disease.^30,31^ In light of these challenges and limitations of conventional pesticides, there is an urgent need for advanced tools in plant science and in innovative agricultural practices, such as sensors for monitoring plant growth and “smart” strategies for improving pesticide application. We anticipated that these innovations possess the potential to enhance crop protection, promote safer practices, and foster greater sustainability in agriculture.

In this context, supramolecular chemists are well-positioned to tackle the emerging critical challenges in plant science and agriculture. By utilising their expertise, supramolecular scientists can contribute to the development of cutting-edge sensors that monitor fundamental plant processes in real-time with unprecedented precision, enabling efficient pesticide detection. In addition, substantial progress can be achieved in creating cutting-edge pesticide delivery systems that minimise the overall use of current state-of-the-art pesticides while facilitating the adoption of innovative mRNA-based pesticide technologies. This know-how encompasses supramolecular systems and tools – molecular assemblies that interact through noncovalent interactions, particularly those responsive to environmental stimuli – which have already shown groundbreaking applications in biomedicine.^32–34^ Thus, we anticipate similar breakthroughs in agriculture and plant science if supramolecular chemists succeed in applying chemosensors, probes, and (nano)-delivery systems to this field and bring them to the market. Particularly, crystal engineering,^35,36^ a technique rooted in the first principles of supramolecular chemistry, has already demonstrated its ability to improve plant resilience, increase overall pesticide efficiency and diminish their toxicity towards mammals.^37,38^ More sophisticated synthetic supramolecular tools, such as self-assembling chemosensors and probes, despite significant advances in targeted imaging and drug delivery for biomedical applications,^32^ are currently difficult to adapt for agricultural use. This is mainly due to the unique physical and biochemical properties of plants, such as their cell walls, phloem, and organelles like chloroplasts, which are absent in mammals, as well as the currently limited understanding of how plants take up, distribute, and eliminate supramolecular systems and nanoparticles. Furthermore, developing supramolecular systems to specifically target certain plant organelles for improved crop protection and plant growth will undoubtedly play an increasingly important role in future research. Equally important will be demonstrating their environmental compatibility and non-toxicity to ensure the approval of new generations of supramolecular (nano)pesticides, chemosensors and probes by national and state environmental and agricultural agencies.

In the spirit of the emphasis of Lowry, Giraldo, and coworkers, who state in their recent review that “there is a tremendous need for disruptive technologies to overcome challenges to meeting future food demand and to meet many of the 17 sustainable development goals…developing solutions to these challenges will require the convergence of thought, approaches and technologies across disciplinary and societal boundaries”,^39^ we believe that supramolecular sensors, producing an optical (*i.e.*, luminescent) signal readout, and delivery systems, particularly those leveraging host–guest chemistry in living plants, represent such essential technologies.

This review aims to summarise and highlight key examples of these systems. Other reviews have previously outlined some specific aspects of the role of supramolecular chemistry in plant science, and interested readers are encouraged to consult these examples as well.^40,41^ Herein, we provide a comprehensive and critical overview of the application of supramolecular tools, with a special emphasis on host–guest chemistry-based examples in plant science and agrochemistry, focussing on their utilisation in living plants. We provide examples related to pest control and highlight developments of new strategies, such as RNA delivery to plants, where supramolecular design principles play an important role. Additionally, an overview of the most relevant patents published in this area is provided. Finally, the overall impact of applying supramolecular principles to sustainable agriculture is discussed, particularly emphasising the key challenges that still need to be addressed in this field. Our goal is to encourage additional research that develops innovative solutions to existing and emerging challenges while promoting interdisciplinary collaboration and networking across a wide range of fields, including biology, chemistry, materials science, and engineering.

### 1.2 Overview of supramolecular interactions

**Fig. 1:**
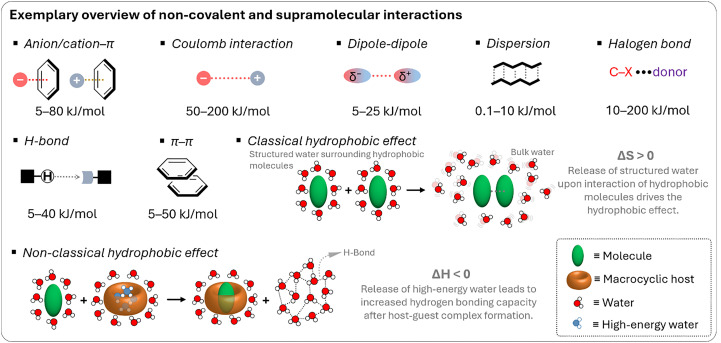
Types of supramolecular interactions and related interaction strengths (average ranges derived from experimental and theoretical calculations).

This section provides non-expert readers with a brief overview of supramolecular interactions and systems, such as molecular probes and chemosensors,^42–46^ with a particular focus on those that operate through host–guest interactions involving cavity-containing macrocycles, referred to as “hosts”, which are being explored for detection and delivery purposes in living plants. To begin with, supramolecular interactions (Fig. 1) encompass salt bridges and ion pairing, hydrogen bonding, halogen bonding, Coulomb interactions, dispersive and stacking interactions, cation/anion–π interactions.^47^ These interactions vary significantly in strength in most organic solvents (see types of supramolecular interactions and related interaction strengths; Table 1), but are generally substantially reduced in strength in water, where strong competition and interference from water molecules impede hydrogen bonding and Coulomb interactions. On the other hand, water as the solvent and main component of any biological fluid gives rise to the classical hydrophobic hydrophobic effect, the displacement of water from hydrophobic molecular surfaces, which provides a favourable entropic contribution to complex formation between hosts and guests.^48^ In addition to the classic hydrophobic effect, a non-classical hydrophobic effect arises from the host molecules due to the displacement of high-energy hydrogen bond-deficient water from the host cavity upon guest inclusion, which enthalpically promotes the formation of the host–guest complex.^49,50^

**Table 1:** General overview of supramolecular interactions

Supramolecular interaction	Enthalpy range^a^	Gibbs free energy (Δ*G*)	Description
Dispersion forces	0.1–10 kJ mol^−1^	Δ*G* is typically close to Δ*H*	Weak, non-specific interactions due to transient dipoles in atoms or molecules, acting over short distances. Minimal entropy changes.
Dipole–dipole interactions	5–25 kJ mol^−1^	Δ*G* is typically close to Δ*H*	Interactions between polar molecules where the positive end of one dipole aligns with the negative end of another. Small entropy changes.
Hydrogen bonding	5–40 kJ mol^−1^	Δ*G* is typically less negative than Δ*H*	A directional interaction between a hydrogen atom bonded to a very electronegative atom (O, N, F) and another very electronegative atom. Entropy changes can reduce Δ*G*.
π–π stacking	5–50 kJ mol^−1^	Δ*G* is typically less negative than Δ*H*	Interactions between aromatic rings due to their π-electron clouds. Entropy loss from ring stacking can affect Δ*G*.
Cation–π interactions	5–80 kJ mol^−1^	Δ*G* can be similar to Δ*H*	Interaction between a cation (positively charged ion) and the π-electron cloud of an aromatic ring. Often entropically favourable.
Halogen bonding	10–200 kJ mol^−1^	Δ*G* can vary significantly from Δ*H*	Interaction involving a halogen atom acting as an electrophile that forms a non-covalent bond with a nucleophile. Entropic effects vary.
Coulomb interactions	50–200 kJ mol^−1^	Δ*G* is typically similar to Δ*H*	Strong attraction between oppositely charged ions or molecules. Δ*G* and Δ*H* can be close if entropy changes are minimal.
Hydrophobic interactions	Varies	Δ*G* is often less negative (classical hydrophobic effect) or more negative (non-classical hydrophobic effect) than/to Δ*H*	Collective effect of nonpolar molecules avoiding water and aggregating together to reduce the interface of water with non-water molecules.

a These are experimentally derived values found in the literature and applied to organic solvents.

To introduce selectivity in host molecules for specific guests or classes of guests, one can explore empiric concepts that are related to the supramolecular interactions mentioned above. These concepts include entropy,^51,52^ multivalency,^53,54^ the packing coefficient,^55,56^ the molecular electrostatic potential surface,^57^ the energetic cost of receptor organisation,^58,59^ conformational freedom and effective molarities,^60,61^ the solvent cohesiveness,^62–64^ the surface site interaction points,^65^ the Hofmeister and chaotropic effect,^66,67^ the solvent accessible surface area,^68^ differential cavitation energies,^69^ and the high-energy water release concept, as previously mentioned. While these concepts are helpful in the design of host–guest systems, they fall short of providing a direct thermodynamic interpretation of binding events and should not be regarded as either a complete picture of the interactions or as mutually exclusive. In the next section, we introduce the most prominent and widely used macrocyclic hosts for sensing and delivery applications. Special emphasis is given to examples of hosts that are characterised by ease of preparation (through low-cost or one-pot synthesis), water solubility, non-toxicity, ease of functionalisation, and high binding affinity for biologically relevant molecules in aqueous environment – factors that are of critical importance in plant sciences.

### 1.3 Macrocyclic hosts

**Fig. 2:**
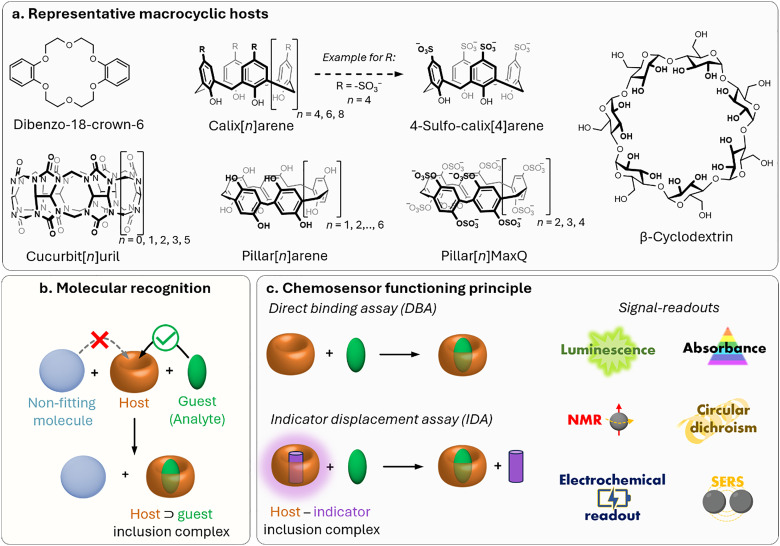
(a) Chemical structures of the most prominent macrocyclic hosts for sensor and delivery applications. For calix[*n*]arene, the residue R can be, for example: –alkyl, –SO_3_^−^, –CH_2_–CH_2_–COO^−^, –CH_2_–NH_3_^+^, –CH_2_–PO_3_^2−^. (b) Host–guest inclusion complex formation occurs when the guest molecule fits into the host's cavity, and intermolecular non-covalent interactions, such as those described in Section 1.2, promote the formation of the complex. This process enables the selective binding of molecular species, leading to the concept of molecular recognition. (c) Basics of the main functioning principles for chemosensors DBA, IDA, and main signal readouts such as nuclear magnetic resonance (NMR) spectroscopy, circular dichroism, electrochemical readout, and surface-enhanced Raman scattering (SERS).

To begin with, it is important to highlight that, in this review, macrocyclic chemosensors are defined as supramolecular tools composed of macrocyclic hosts possessing a cavity that reversibly binds their guests (*i.e.*, small organic molecules, cations or anions, among others) under experimental assay conditions. These macrocyclic hosts rely on noncovalent (supramolecular) interactions to selectively bind the guest inside or around their cavity, a process known as molecular recognition (Fig. 2).^70^ In this context, the use of macrocyclic hosts offers the opportunity to transport and protect plant-active molecules, such as pesticides or nutrients, for improved delivery applications (see Sections 3.2 and 3.3). Moreover, macrocyclic hosts equipped with a suitable reporter molecule can produce a detectable signal change (*e.g.*, optical, electrical, *etc.*) in the presence of an analyte, thereby forming a chemosensor ensemble (see Section 2.2). Importantly, they can dynamically adjust (*i.e.*, equilibrate) to changes in the sample's composition, in contrast to molecular probes, which are small organic molecules (see Section 2.3), that typically provide a static representation, thus making macrocyclic systems particularly suited for real-time monitoring of (bio)chemical and (bio)physical processes. From a historical viewpoint, the first synthetic macrocyclic supramolecular host compounds for detecting metal cations (*e.g.*, K^+^, Li^+^) were crown and aza-crown ethers, and bicyclic compounds of the crown ether type, also known as cryptands.^71–75^ Notably, these have been successfully commercialised into sensor cassettes for biomedical applications;^76,77^ however, to our knowledge, they have not been used in plant science or agrochemical research due to their selectivity for alkali metal cations, which limits their applicability. This review primarily discusses macrocyclic compounds that act as cavity-bearing hosts for small organic molecules, which they bind through a combination of various host–guest interactions.

#### 1.3.1 Cyclodextrins

Cyclodextrins^78–80^ (CDs, Fig. 2a) are typically classified into three main homologs: α-cyclodextrin (αCD), composed of six glucopyranose units; β-cyclodextrin (βCD), containing seven units; and γ-cyclodextrin (γCD), comprising eight units. The size of the internal hydrophobic cavities, which possess a conical shape, increases in the following sequence: 4.7–5.3 Å (αCD), 6.0–6.5 Å (βCD), and 7.5–8.33 Å (γCD).^81^ Generally, CDs show acceptable chemical stability, although they tend to hydrolyse under very acidic conditions. Regarding the acidity of the hydroxyl groups, their p*K*_a_ values range from 12.1 to 13.5.^82^ In addition, CDs are water-soluble; solubility values of 130 mg mL^−1^ for αCD, 18.5 mg mL^−1^ for βCD, and 249 mg mL^−1^ for γCD were reported.^83^ Functionalisation of βCDs through the reaction of the secondary and/or primary hydroxyl groups to their respective hydroxypropyl or sulfobutyl ether^84^ increases their solubility, up to 700 mg mL^−1^,^85^ and significantly reduces their otherwise inherent tendency to aggregate.^86^

CDs are already widely used in industry, including in agrochemicals, more than other synthetic macrocyclic compounds.^87^ This is mainly due to the efficient synthesis and purification processes that are available, and their acquired approval by the U.S. Food and Drug Administration (FDA) as “generally recognised as safe” (GRAS) status in the early 1990s.^88–90^ Furthermore, when used as hosts for hydrophobic molecules, CDs facilitate their uptake by plants as they increase solubility while also improving the chemical stability of the guest molecules by protecting them from degradation by light, heat, and reactive oxygen species (ROS).^91–94^ Some examples of commercially available products from and for the agrochemical sector include CAVAMAX® (composed of αCD) and CAVASOL® (composed of hydroxypropyl-derivatives of α-, β- and γCDs) cyclodextrins (by Wacker Chemie),^95,96^ which are used as additives to biocides to reduce their volatility, extend efficacy (prolonged pesticide release), and improve stability and water solubility. The use of cyclodextrins to trigger defence reactions in plant cells and promote the accumulation of secondary metabolites has been detailed and summarised in a recently published review.^97^ Interested readers are directed to that review, as this topic lies beyond the scope of the present work. Concerning pesticide reformulation strategies with CDs applied to fruits, food and packaging, we focus only on recent examples, as their specific applications have been excellently reviewed elsewhere.^98,99^

#### 1.3.2 Calix[*n*]arenes and resorcin[*n*]arenes

Calix[*n*]arenes (CX*n*, Fig. 2a) were first synthesised by A. Zinke *via* the reaction of *p*-alkyl phenols, such as cyclic oligomers of *p*-hydroxyalkylphenols, with formaldehyde in strongly basic solutions.^100^ Later, J. Niederl and H. J. Vogel produced similar cyclic tetramers through the acid-catalysed reaction of 1,3-dihydroxybenzene (resorcinol) with aldehydes, such as benzaldehyde, yielding to what was successively identified as resorcin[*n*]arenes. The cyclic structure of calixarenes was confirmed by C. D. Gutsche^101^ and by the group of Andreetti, Ungaro, and Pochini,^102–104^ whereas that of resorcin[*n*]arenes was elucidated by H. Erdtman and coworkers.^105^ CX*n* consists predominantly of 4, 6, or 8 phenolic units linked by methylene bridges, forming a cup-shaped structure with a hydrophobic cavity and phenolic OH groups at the bottom. The phenolic –OH groups can be further functionalised to improve water solubility, for example, by introducing sulfonate, phosphonate, or trimethylammonium groups.^106–108^ From a physicochemical standpoint, CX*n* possess cavity sizes determined by the number of phenolic units: CX4 has a cavity of 3–5 Å, CX6 of 6–8 Å, and CX8 of 9–11 Å (Table 2), whereas more recently also so-called giant calixarenes have been described with 90 phenolic subunits.^109^ Their different sizes, conformational flexibility and ability to be functionalised with a variety of groups strongly influence binding with guests, making this macrocyclic host class extremely versatile.

**Table 2:** Physicochemical properties of macrocyclic hosts. Shown are average values for the cavity diameter, molecular weight and solubility reported in the literature

Host	Cavity diameter [Å]	Molecular weight [g mol^−1^]	Solubility [mmol L^−1^]
Cyclodextrin^81^			
αCD	5.00	972.80	133.60
βCD	6.30	1134.98	16.30
γCD	8.00	1297.12	192.00
Sulfobutyl-βCD^85^	—	2242.10	31.25
Calix[*n*]arene^182^			
CX4	3.00	424.50	—
CX6	7.60	636.73	—
CX8	10.00	848.98	—
Cucurbit[*n*]uril^183^			
CB5	4.40	830.70	25.00
CB6	5.80	996.80	0.03
CB7	7.30	1162.98	≤20.00
CB8	8.80	1329.10	<0.01
CB10	11.70	1661.37	<0.05
Pillar[*n*]arene^170,184,185^			
PA5	4.70	610.62	—
PA6	7.50	718.71	—
PA7	8.70	826.81	—
Sulfated PA5	—		100.00
Sulfated PA6	—		20.00

Host–guest complexes of calixarenes have been extensively studied by the aforementioned groups, leading to the development of chemosensors based on indicator displacement assays (IDAs) by the groups of Inouye^110^ and Shinkai,^111^ using resorcin[*n*]arenes and CX*n* based chemosensors. Further significant contributions were made by the Anslyn^112–114^ and the Dalcanale groups.^115^ Anionic derivatives of resorcin[*n*]arenes and CX*n* have been used for the detection of cationic analytes, such as viologen derivatives,^116^ toxic cations,^117,118^ illicit drugs,^119^ and protein,^120^ as well as anions such as phosphates,^121^ or carboxylates, amongst others.^122^

Regarding their biological safety, anionic CX*n*, such as sulfonated calixarenes, appear not to elicit acute toxicity in cells or mice – an important consideration for their application in plant sciences.^123–125^ We refer readers interested in CX*n* applications in fields other than plant science to comprehensive overviews on these topics.^126–128^

#### 1.3.3 Cucurbit[*n*]urils

Cucurbit[*n*]urils (CB*n*, Fig. 2a) were first synthesised by R. Behrend in 1905 through an acidic condensation reaction between glycoluril and formaldehyde.^129^ However, it wasn’t until 1981 that W. A. Freeman, W. L. Mock, and N.-Y. Shih elucidated the cyclic nature of CB6,^130^ revealing that glycoluril monomers are linked by methylene bridges, forming a hydrophobic “barrel” that is flanked with two carbonyl-functional portals as polar cavity entrances. Later, the group of Kimoon Kim reported the structure of the cucurbituril homologues CB5, CB7, and CB8^131,132^ shortly before Day and Nau also revisited the CB*n* forming reactions independently.^133–135^ The Stoddart group reported the structure of decamethylcucurbit[5]uril while the group of Isaac reported the CB10 homologue.^136^ More recently, the CB14 homolog has also been discovered by the Tao group,^137^ which adopts a twisted conformation. The average inner cavity diameter varies with the number of glycoluril units, following the order CB5 (∼4.4 Å), CB6 (∼5.8 Å), CB7 (∼7.3 Å), CB8 (∼8.8 Å), and CB10 (∼11.70 Å), whereas the height of the cavity is ∼9 Å for all homologs (Table 2).^138^ Due to its small cavity size, CB5 binding to noble gases have also been reported,^69,139^ whereas larger homologues can encapsulate larger organic molecules with biological relevance. While CB5–CB7 are generally, with some exceptions, form 1 : 1 host–guest complexes, CB8 with its larger cavity often yields homoternary (1 : 2) or heteroternary (1 : 1 : 1) host–guest complexes, especially with aromatic guests.^140^ In addition, CB*n* exhibit exceptionally high binding affinities for a wide range of biomolecules and drugs in aqueous media (typically *K*_a_ ≈ 10^3^–10^9^ M^−1^), ranging up to attomolar affinities in some specific cases.^141–143^ The high binding affinities arise from the size complementarity between the host and the guest, an enthalpic gain from the binding event, the expulsion of high-energy water molecules (non-classical hydrophobic effect), and, in some cases, the removal of the guest's solvation shell (classical hydrophobic effect), resulting in additional entropic and in some cases also enthalpic gains^50,63,144–146^ during host–guest complex formation.^49,147^ It is important to keep in mind that CB*n* presents not negligeable interactions with some metal ions, that should be taken into account when working in medium-high ionic strength.^148^

Functionalisation of CB*n* is more cumbersome than that of CDs or CX*n*: current strategies include the direct functionalisation of CB*n* or the use of functionalised glycolurils for CB*n* synthesis.^132,149–155^ However, the efficient functionalization of CB*n* remains a largely underexplored area, and further research is essential to expand their applicability. Nonetheless, mono-functionalized CB*n* derivatives bearing reporter dyes have been developed,^155–158^ enabling the design of unimolecular indicator displacement assays. Such systems are particularly promising for creating salt- and dilution-stable chemosensors, including those with multimodal readouts. Toxicological studies have shown that CB*n* exhibits no acute toxicity to mammalian cells and mammals such as mice (the tolerated dose for injected CB7 was 250 mg kg^−1^, while it was 600 mg kg^−1^ for an orally administrated mixture of CB7 and CB8).^159,160^ Moreover, the Wang group demonstrated that administering CB7 to mice, whether orally at 5 g kg^−1^, intraperitoneally at 500 mg kg^−1^, or intravenously at 150 mg kg^−1^, did not lead to any significant differences in body weight across the treatment groups.^137^ As for their water-solubility (Table 2), the concentration of CB5 and CB7 in neat water can reach as high as 5 mM, with values as high as 20 mM also reported, whereas CB6, CB8 and CB10 only dissolve on a micromolar scale. Combined with their excellent physicochemical properties for binding biomolecules, drugs, and pesticides, this makes them promising candidates for plant sciences and agrochemical applications.

#### 1.3.4 Pillar[*n*]arenes

Pillar[*n*]arenes (PA*n*; with *n* = 5, 6, 7) are macrocyclic hosts composed of hydroquinone units linked by methylene bridges at the para positions, and were first reported by Ogoshi, Nakamoto, and co-workers (Fig. 2a).^161^ Functionalised PA*n*s are prepared by reacting their alkoxy or hydroxyl groups, either after hydrolysing alkoxy-PA*n* or by directly using mixed dialkoxybenzenes during synthesis, with suitable elctrophiles.^162–167^ Particularly, PA5 has a unique, symmetrical, and conformationally stable structure, appearing as a pentagon from the top view and a pillar from the side. PA5, PA6, and PA7 have inner diameters of 4.7, 7.5, and 8.7 Å, respectively (Table 2).^167^ The host–guest behaviour of PA5, as studied by the Li group and Ogishi's group, showed the formation of 2 : 1 external complexes with *N*,*N*′-dialkyl-4,4′-bipyridiniums and 1 : 1 pseudorotaxane-type inclusion complexes with methylene-bridged bis(pyridinium) derivatives of appropriate chain length.^161,168^ While functionalised PA*n* can exhibit structural rigidity, non-functionalised PA*n* possesses rotational freedom that is largely dependent on intramolecular H-bond interactions, temperature, solvent and the addition of a guest.^167,169^ Water-soluble PA*n* variants exist and include, for example, the previously mentioned carboxy derivatives, as well as sulphated and sulfonated derivatives such as sulphated PA5 and sulfated-PA6, with solubilities of 100 mM and 20 mM, respectively.^170^

Concerning their toxicity to mammalian cells, pristine PA*n*s and carboxy-bearing derivatives showed low toxicity, whereas certain functionalised derivatives, *e.g.*, those with alkyl groups, exhibited some toxicity.^171,172^

#### 1.3.5 Other macrocyclic hosts

While these aforementioned host classes remain up to date the most prominent examples of macrocyclic hosts in research and applications, the search for new macrocyclic hosts continues to be an active and valuable field, with the potential to yield novel structures possessing enhanced binding properties, such as improved selectivity and the ability to host larger guests. This is exemplified by the development of the so-called deep cavitands (or deep cavity cavitands), which are prepared from resorcinarene or calixarene scaffolds and possess extended aromatic “walls”.^173–176^ Furthermore, while most of these macrocycles exhibit strong binding affinity for hydrophobic and cationic guests, bambus[*n*]urils have been reported to show strong binding properties for anions.^177,178^ In addition to macrocyclic hosts, coordination compounds also exhibit interesting host–guest properties and multifunctionality that can be used for drug delivery and sensing applications.^179–181^ However, these latter structures, which are nowadays gaining increasing attention in the biomedical field, have so far not been investigated in plant science.

### 1.4 (Supra)molecular probes

**Fig. 3:**
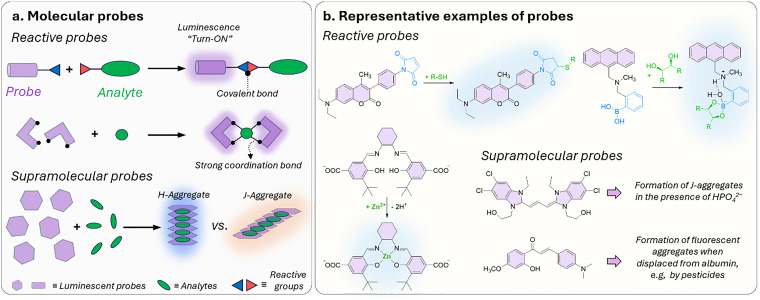
(a) Schematic representation of reactive and supramolecular probes, and (b) examples of their applications. Except for the boronic ester-containing probe, which is adapted from ref. 193, all other examples are discussed within this review.

For the purposes in this review, it is important to clarify now the distinction between molecular and supramolecular probes, both of which will be explored in detail. While the distinction is sometimes blurred in the literature, we follow a simple and widely accepted terminology where molecular probes (Fig. 3) are defined as non-cavity-containing molecular systems that form strong, often irreversible, bonds with target analytes, typically accompanied by a distinct optical response, such as changes in light emission or absorption spectra. These reactive probes, *e.g.*, maleimide-linked fluorophores,^186^ react with nucleophiles like thiols and amines to produce luminescent conjugates, being covalently bonded with their targets. As another example, boronic acid esters can form reversible covalent bonds with 1,2-diol-containing analytes, generating luminescent derivatives.^187^ The latter concept of employing reversible covalent binding, also known as dynamic covalent chemistry,^188,189^ enables the exploitation of discrete molecular components that reversibly associate through covalent bonds, allowing them to assemble and disassemble under equilibrium control. This approach facilitates the design of novel dynamically behaving systems for several applications, such as sensing^190^ among others. For example, by taking advantage of the equilibrium-controlled formation of boronic esters from boronic acids and diols, glucose sensors have been developed and commercialised, *e.g.*, the glucose sensor marketed by GlySens.^191^

In addition, some ligands (*e.g.*, salen-based ligands) coordinate with divalent ions, like Zn^2+^, to form emissive metal complexes, thus being them considered reactive probes, too.

In contrast, supramolecular probes are non-cavity-containing molecular systems; however, they interact with analytes through the formation of reversible, non-covalent interactions, which are much weaker compared to the typically covalent nature of interactions observed with molecular probes.^188^ In this context, the interaction of the supramolecular probe with the target analyte leads to the formation of supramolecular assemblies (often referred to as aggregates) with distinct optical properties, such as detectable changes in luminescence or characteristic color shifts, which enables their use as sensors. For instance, cyanine dyes can form supramolecular assemblies,^192^ such as J-aggregates or less ordered structures, in the presence of analytes, exhibiting distinct photophysical properties compared to the monomer forms, thus enabling the development of luminescence-based sensors.

### 1.5 Nanomaterials

Nanomaterials are typically defined as nanoscale systems that have at least one dimension within the range of 1 nm to 100 nm and exhibit physicochemical properties that significantly differ from those of their bulk state, such as altered photophysical behaviour or catalytic activities.^194,195^

It is important to note that materials with sizes in the range of several hundred nanometres can also be considered nanomaterials if their physicochemical properties differ significantly from the bulk state, a definition that is increasingly accepted in the literature.^196^ Nanomaterials have been extensively utilised in both sensing and delivery applications over the past decades. From a sensing perspective, they have enabled the study of cellular metabolic processes down to the single-molecule level,^197–200^ improved environmental monitoring,^201,202^ and facilitated the development of portable sensors for continuous biosensing applications.^203,204^

**Fig. 4:**
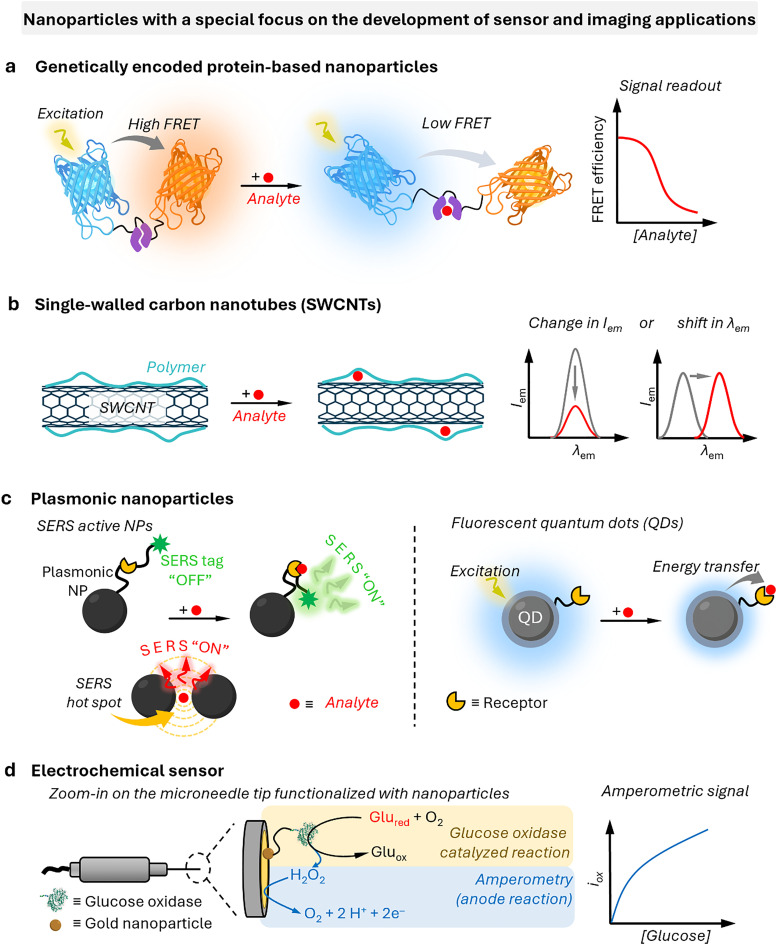
Representative examples of nanomaterials used for developing nanosensors in plant research, *i.e.*, (a) genetically encoded protein-based nanoparticles, (b) semiconducting single-walled carbon nanotubes, (c) plasmonic nanoparticles (*e.g.*, gold and silver nanoparticles), (d) nanoparticles-based (electrochemical) sensors.

**Fig. 5:**
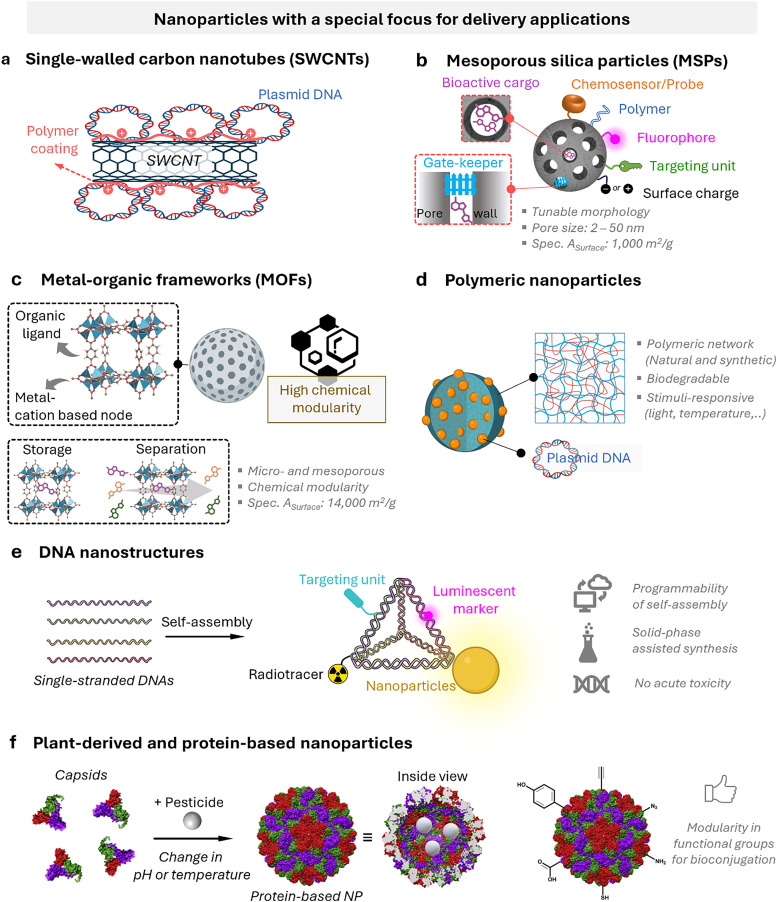
Representative examples of nanoparticles used for the delivery of bioactive molecules to plants include: (a) single-walled carbon nantorubes, (b) mesoporous silica nanoparticles, (c) metal–organic frameworks, (d) polymeric nanocarriers, (e) DNA nanostructures, (f) plant-derived and protein-based nanoparticles, *e.g.*, virus capsid nanoparticles.

In drug delivery, nanomaterials have enabled the precise transport of a wide range of therapeutic agents, from small organic molecules to large biomolecules, such as nucleic acids, allowing for their delivery to diseased cells and tissues.^205,206^ Furthermore, nanomaterials can be designed to be stimuli-responsive, breaking down in the presence of specific physical or chemical triggers. This property enhances their degradation and excretion, ultimately leading to potentially safer drug formulations by facilitating drug release and particle clearance.^207–209^ Currently researched nanomaterials in plant sciences are based on carbon nanotubes,^210–213^ liposomes,^214,215^ organic polymers,^216–218^ metals (*e.g.*, silver and gold),^219^ oxides (*e.g.*, zinc oxide, titanium oxide, silica, aluminosilicates),^220–229^ chalcogens (sulphur or selenium)^230–232^ or peptide-based nanoparticles (see Fig. 4 and 5).^233^

The agrochemical research and industry has already implemented nanomaterials, for example to reformulate pesticides, and fertilisers, encapsulating them in nanometre-sized delivery systems.^39,234–239^ The pesticide formulations developed by Vive Crop incorporate polymer nanoparticles that encapsulate active ingredients. This advanced methodology effectively safeguards the sensitive pesticide compounds from chemical degradation, thus enhancing the stability and efficacy of the pesticides over time.^240^ In addition, Banner MAXX 67 (from Syngenta) is a fungicide stabilised with tetrahydro furfuryl alcohol in micro/nanoemulsions.^236^ Furthermore, porous nanoparticles, such as zeolites, are not only useful as carriers for biomolecules but can, when mixed with the soil, improve water retention and soil structure, as was shown in the case of ZeoSand (marketed by Zeocem).^241^

One promising direction for future research is targeted delivery, such as methods that utilise triggered cargo release for extended pesticide effectiveness and the potential for nanocarriers to break down into non-toxic byproducts that are environmentally friendly. Moreover, the use of nanoparticles has shown a positive impact on improving gene therapies in plants by stabilising fragile nucleic acid-based cargo against degradation while simultaneously enabling organelle-specific delivery in plants.^242^ Therefore, considering the emerging and promising potential to revolutionize crop protection and efficacy, the use of nanomaterials will be pivotal in the development of CRISPR/Cas9 gene editing tools and RNA interference (RNAi)-based pesticides,^28,243–245^ by providing the necessary protection and improving the mode of action. For example, the company AgroSpheres has developed genetically modified bacteria that produce double-stranded RNA (dsRNA) encapsulated within a lipid-bilayer nanoparticle, leading to a promising RNA-based pesticide to combat diamondback moth pests.^246^ Given the delicate nature of bilipid membranes, there is significant interest in developing new nanomaterials that can provide both a well-controlled, stimuli-responsive release profile and long-term stability.

In addition to delivery applications, it is important to note that the utilization of nanomaterials for the real-time detection of plant metabolites and pesticides remains in an early stage. This limitation is primarily attributed to the restricted capacity of nanomaterials larger than 20 nanometres to effectively penetrate plant cellular structures, thereby hindering their application in agricultural monitoring processes.^247,248^ In addition, questions remain regarding the fate and safety of these materials in the environment, which is essential for determining their bioavailability.^216,249,250^ Specific applications in plant sciences will be discussed in more detail in later sections. We first provide an overview of the main nanomaterials currently being researched for applications in this field.

#### 1.5.1 Genetically encoded protein-based nanoparticles

The discovery of the green fluorescent protein (GFP)^251,252^ and advances in protein engineering provided the starting point for the development of genetically encoded sensors based on fluorescent proteins (genFPs), which are among the best-studied examples of nanosensors in plant science (Fig. 4a).^253^ For genFPs, signal transduction based on Förster resonance energy transfer (FRET)^254^ represents the main signal readout.^255–257^ FRET-based sensors operate by detecting the light emitted from a FRET donor–acceptor dye pair. During the FRET process, photoexcitation of the donor dye results in energy transfer from its excited state to the energetically lower excited state of the acceptor dye, causing the latter's sensitised emission. The FRET efficiency is highly dependent on the distance between the donor–acceptor pair, thus enabling the detection of plant analytes modulating the distance or conformation of the FRET donor–acceptor pair.^258^ Therefore, in their most basic design principle, genetically encoded and FRET-based nanosensors consist of two FRET-active proteins linked by an analyte binding domain (*i.e.*, recognition domain). Following analyte binding, a conformational change occurs in the nanosensor, thus modifying the relative distance between the two FRET donor–acceptor dyes. This process can be monitored by recording the relative emission intensities of the two genFPs after the excitation of the donor.

#### 1.5.2 Single-walled carbon nanotubes

Semiconducting single-walled carbon nanotubes (SWCNTs, Fig. 4b)^259,260^ can be used for preparing fluorescent nanosensors because of their near-infrared (NIR) emission^261^ – a favourable feature for *in vivo* analyses, as plant constituents do not strongly absorb in the long-wavelength region. Signal transduction with SWCNTs can be achieved by taking advantage of the fact that their emission properties, *e.g.*, emission wavelengths or intensities, strongly depend on the chemistry of their surface (corona)composition (Fig. 4b). Strano and co-workers introduced this concept by wrapping SWCNTs with (bio)polymers, such as DNA, which can bind specific analytes. This binding altered the dielectric environment of the SWCNTs, a phenomenon known as corona phase molecular recognition (CoPhMoRe),^260,262–264^ which ultimately leads to modulated fluorescent signals.

As drug delivery carriers (Fig. 5), SWCNTs have gained considerable attention due to their extremely high surface area (>1000 m^2^ g^−1^)^265^ and the possibilities with which their surface can be functionalised with hydrophilic polymers like nucleic acids to enhance water solubility, as well as with other performance enhancers such as dyes, targeting moieties, and drugs.^266–268^ Additionally, SWCNTs also enable a relatively novel uptake mechanism in plants, known as the lipid exchange envelope penetration (LEEP) model,^269^ which allows them to be transported into and trapped within intact chloroplasts.

#### 1.5.3 Carbon dots

Carbon dots (CDots, Fig. 4)^270,271^ have emerged as promising nanomaterials for fluorescence-based sensing and bioimaging due to their excellent dispersibility in aqueous media, ease of synthesis, and attractive photoluminescent (PL) properties. These properties include excitation wavelength-dependent emission, robust photostability, and high photoluminescence quantum yields (PLQY) of up to 99%.^272^ Thus, CDots are interesting for a wide range of applications in the fields of biomedicine, catalysis, and optoelectronics.^273–275^ In particular, the excitation-dependent luminescence of CDots, which enables multicolour bioimaging without altering their chemical structure or size, is attributed to multiple photoluminescent centres and a broad distribution of energy levels.^271^ Functionalisation of CDots is possible to extend their applications,^276^ through techniques such as heteroatom doping and surface modification, enhancing their performance and expanding their application. These modifications can provide CDots with additional reaction sites, enhanced stability, and other tailored functionalities for specific tasks. The ability to fine-tune these nanoscale carbon-based materials has expanded their use in targeted drug delivery and bioimaging,^277,278^ amongst other uses, highlighting their versatility.

#### 1.5.4 Plasmonic nanoparticles

Plasmonic nanoparticles are a class of nanomaterials that include various metal nanoparticles, such as gold, silver, and copper. Their distinctive optical properties arise from their interaction with light, where the oscillating electric field induces a collective oscillation of conduction electrons at the metal surface – a phenomenon known as localised surface plasmon resonances (LSPRs).^279^ Surface-enhanced Raman spectroscopy (SERS)^280^ is a technique used to detect molecules (analytes) by exploiting the large amplification of the Raman signal (by up to 10^14^ times) when molecules are positioned on or between plasmonic nanostructures.^281^ This enhancement occurs in LSPR hotspots, where light-excited plasmonic nanoparticles create strong local electric fields. Molecules in these hotspots experience a much stronger electromagnetic field, leading to a significantly amplified Raman response. While exciting near the LSPR maximum is generally preferred, as it corresponds to the strongest collective oscillation of conduction electrons, a slightly red-shifted excitation may be preferable if either the analyte or the SERS substrate exhibits fluorescence at or near the plasmon resonance wavelength, improving the signal-to-noise ratio.

Gold and silver nanoparticles (AuNPs and AgNPs) represent prominent substrates for the design of SERS nanosensors (Fig. 4c),^282–285^ because their morphology can be precisely tuned.^286–288^ Moreover, the nanoparticles’ surface can be easily modified with additional functional groups, such as stability enhancers,^289,290^ dyes,^291^ and receptor molecules,^292–295^ through various methods,^296^*e.g.*, thiol-mediated ligand exchange reactions, which have been shown to increase performances of nanosensors.

#### 1.5.5 Quantum dots

Similarly to metal nanoparticles, QDs (Fig. 4c)^297–299^ are semiconductor nanoparticles with unique photophysical properties related to their characteristic small size (*i.e.*, 2–10 nm). The emergence of discrete quantized energy levels in these systems determines their luminescent properties, which relates them more closely to atoms than to bulk materials.^300,301^ The most popular materials for biological applications are CdSe,^302^ CdTe,^302^ but also III/V group semiconductors or ternary semiconductors, such as InP,^303,304^ InGaP^305^ or AgInS,^306^ which do not contain cytotoxic cadmium ions, are noteworthy alternatives. The colour of the resulting fluorescent emission can be tuned by changing the diameter and composition of the nanoparticle. Functionalisation, *i.e.*, organic capping ligands or passivation, *e.g.*, by creating a ZnO shell around the QD core,^307–310^ is possible and often crucial for achieving high fluorescence quantum yields, obtaining longer decay kinetics, and high stability.^311^ As for their physical features, QDs display a broad excitation spectrum while the resulting emission remains narrow, *i.e.*, full width at half maximum (FWHM) intensity ranges from 20 to 40 nm. In addition, QDs are characterised by a relatively large Stokes shift, which facilitates the acquisition of their entire emission spectrum. Therefore, these properties facilitate their application to multiplexed imaging. For example, their broad absorption spectrum implies that a single excitation wavelength can be used to excite multiple quantum dots with different emission colours simultaneously. In a biological context, QDs are also attractive as fluorophores because they have a relatively good multiphoton excitation cross section and can emit infrared and far-infrared light. In addition, the surface of QDs can be functionalised with fluorescent molecules participating in energy transfer processes,^312^ and receptor molecules^313–315^ – all features that can be exploited for developing advanced QD-based nanosensors.

#### 1.5.6 Mesoporous silica nanoparticles

Shortly after the successful research into producing micrometre-sized mesoporous silica particles (MSPs, Fig. 5b),^316–318^ sol–gel-based bottom-up synthesis techniques for producing nano-sized particles were proposed,^319–321^ with the most recent ones comprising single-micelle templated synthesis methods.^322–325^ The presence of ordered mesopores (pore size: 2–50 nm) in MSPs allows for the encapsulation of bioactive molecules, such as pesticides,^326^ enzymes^327–329^ and nucleic acids,^330,331^ while the outer surface of the particles can be functionalised *via* alkoxysilane-based chemistry with performance enhancers, such as dyes, catalysts, targeting moieties (*e.g.*, cell surface receptor ligands) and polymers, for enhanced colloidal stability.^332–334^ Importantly, silica is environmentally friendly as it is a major constituent of soil and has received GRAS approval from the FDA. Besides, several clinical trials using silica-based nanoparticles are currently underway.^335^ An intriguing aspect of employing sol–gel-based synthesis methodologies to prepare MSPs is the ability to covalently integrate reactive organic functional groups into a silica framework. This integration results in the formation of what is known as mesoporous organosilica particles. In this context, various stimuli-responsive groups have been used to design on-demand breakable nanoparticles, including redox-reactive (*e.g.*, disulfide and diselenide),^324,336–338^ and hydrolysable (*e.g.*, amide, oxamide, carbamates, imines and nucleic acids) groups.^339–344^ This functional design helps reduce unwanted bioaccumulation, because particles can be engineered to degrade quickly after being exposed to a specific external or in-plant-occurring stimulus.

#### 1.5.7 Metal–organic frameworks

Metal–organic frameworks (MOFs, Fig. 5c)^345^ represent a class of crystalline microporous materials synthesised through the self-assembly of metal ions or clusters, such as Zr^4+^, Fe^3+^, and Zn^2+^, with organic ligands like carboxylic acids or amines.^346^ An exceptionally attractive feature is the ability to impart a high degree of chemical modularity to them, as a wide variety of metal nodes and organic linkers are available for the preparation of functional MOFs. Characterised by exceptionally high porosity, MOFs can achieve internal surface areas (sometimes exceeding 6000 m^2^ g^−1^) and pore volumes approaching 90%, making them important candidates for various advanced applications such as in gas storage (*e.g.*, hydrogen and methane storage) and selective adsorbents for separation processes.^347–349^ Recently, their high porosity has prompted the exploration of drug-delivery applications,^350,351^ given their capacity for high drug loading and potential for controlled release. However, challenges remain, including concerns about their toxicity and stability in water, which limit the scope of their applications. Yet, some MOFs have reached applications in environmental contexts, such as in water harvesting,^352^ showcasing how intelligent design can address stability concerns and potentially reduce ecotoxicity.

#### 1.5.8 Polymeric nanoparticles

Polymeric nanocarriers (Fig. 5d) consisting of natural or synthetic polymers generally exhibit greater stability in biological fluids than lipid-based nanoparticles that are prominently used in the biomedical and agrochemical fields.^353^ Their physicochemical properties can be easily tuned as various synthetic methods are available to produce polymers and block copolymers with well-defined chain lengths, chemical compositions and morphologies.^353^ An effective strategy for developing degradable polymer nanoparticles is to incorporate labile crosslinkers. For instance, acid-labile crosslinkers allow for the degradation of nanoparticles in acidic environments, such as those present in certain tumors or lysosomes compartments.^354^ Polymeric nanoparticles offer extensive chemical modularity as their building blocks can be easily modified to achieve tunable properties such as molecular weight, charge, chemical functionality and hydrophobicity. A wide range of polymeric nanoparticles for biomedical applications is currently being investigated, typically categorized into synthetic polymers and biopolymers such as chitosan or hyaluronic acid.

#### 1.5.9 DNA nanostructures

DNA nanotechnology^355,356^ has paved the way for the preparation of nanoparticles with a variety of shapes with unrivalled precision, driven by the programmable and predictable Watson–Crick–Franklin base pairing of nucleobases.^357^ These DNA-based assemblies (Fig. 5e) provide fine-tuneable control over size, shape, and function, making them versatile tools for biological applications, such as imaging, sensing, and drug delivery.^358–361^ The now relatively straightforward synthesis and functionalisation of DNA using various methods, such as solid-phase peptide synthesis, enables production on a laboratory scale, and some larger-scale synthesis methodologies exist.^362–364^ However, new methods must be developed to enhance production and facilitate the economical use of these materials for commercial purposes. Despite the many successful stories of DNA nanostructures for their biomedical applications, challenges remain in achieving effective *in vivo* stability, targeted distribution, and cellular uptake of DNA nanostructures.^365–367^ These challenges need to be addressed to fully realize their potential in the medical field, and also for plant science in the context of this review.

#### 1.5.10 Virus capsid nanoparticles

The self-assembly of virus capsids (Fig. 5f) into hollow protein-based nanoparticles, *i.e.*, virus-like nanoparticles,^368–371^ has been used to enable the synthesis of soft (nano)containers for a variety of applications, ranging from drug delivery^372^ to catalysis^373^ and imaging applications.^374^ Another interesting aspect of the use of certain plant virus-like nanoparticles, such as tobacco milkweed mosaic virus (TMGMV) and cowpea mosaic virus, is their excellent penetration depth into the soil (up to 30 cm). This makes them ideal carriers for nematicides in the rhizosphere – a property that contrasts with mesoporous silica particles or poly(lactic-*co*-glycolic acid) particles, which penetrate relatively poorly into the soil.^375^ Nanopesticides based on TMGMV to control the invasive weed are already commercially available (manufactured by BioProdex),^376^ having previously been approved by the United States Environmental Protection Agency (EPA).^377^

### 1.6 Delivery strategies to plants

Generally speaking, the delivery of substances to plants can be accomplished in various ways, including uptake *via* the foliar surface, through the roots, or by infiltration methods.

**Fig. 6:**
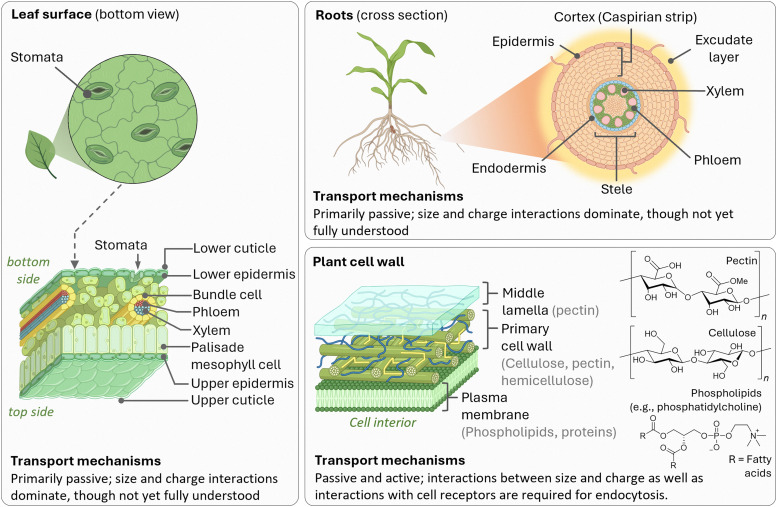
Schematic representation of the generalized structural barriers in plants those exogenous substances, such as chemosensors and nanomaterials, must traverse to reach plant cells. The illustration highlights the principal transport mechanisms involved, along with representative chemical structures of key cell membrane components.

The foliar delivery route primarily exploits the stomatal pathway as the main route through which plants absorb substances *via* their leaves (Fig. 6).^378^ Stomata are pore-forming structures within the micrometre size range, located in the epidermis of leaves, and consist of two guard cells that are essential for gas exchange during photosynthesis. In addition to the stomatal pathway, the cuticular pathway also contributes to leaf uptake. The plant cuticle is an extracellular, hydrophobic layer that covers the aerial epidermis of all land plants and serves as a protective barrier against dehydration and various environmental stresses.^379^ This layer is composed predominantly of cutin,^380^ a complex polyester-based biopolymer, and includes C20–C34 wax compounds such as alkanes, aldehydes, primary and secondary alcohols, ketones, and esters. Due to its waxy and hydrophobic nature, and with pore sizes typically smaller than 2.4 nm,^381,382^ the cuticle presents a major barrier to the penetration of substances. For efficient delivery *via* the foliar pathway, the adhesive properties of the applied substances are of crucial importance. Particle size, whether in the form of molecular aggregates or nanoparticles, plays an important role, as larger particles are more easily washed off and exhibit reduced adhesion to leaf surfaces,^383^ a phenomenon that is particularly pronounced for hydrophilic substances since plant leaves, especially those with thick wax layers, tend to repel aqueous formulations. To enhance leaf uptake, surfactants such as Triton X-100, Silwet L-77, sodium dodecyl sulfate, and dodecyltrimethylammonium bromide are often employed, as these surfactants reduce surface tension and facilitate the release of active ingredients at the leaf surface.^247^ After penetrating the epidermis *via* either the stomatal or the cuticular pathway, substances are primarily transported within the plant *via* the phloem.^384^ This vascular tissue transports plant metabolites from the leaves to the basal parts and consists of various cell types, including sieve elements, companion cells, and phloem parenchyma (in trees, the phloem represents the innermost part of the bark). More in detail, it is generally believed that the mass flow of the phloem, which constitutes the principal sugar-transporting tissue in plants, is simply driven by an osmotically generated pressure gradient.^385^ The accumulation of sugars and other substances in the phloem is the starting point for the long-distance sugar transport in plants, which can be either apoplastic (crossing of the cell wall and the plasma membrane) or symplastic (direct cytoplasm-to-cytoplasm crossing).^386^ A critical factor that may limit the systemic transport of non-plant metabolites *via* the phloem is the size restriction imposed by the pores of the sieve plates, which is largely species-dependent and can typically range from several micrometers to a few hundred nanometers.^387,388^ However, a comprehensive understanding of the factors that determine the efficiency of phloem translocation from the plant surface, particularly regarding physicochemical properties such as charge and surface coating of small molecules and nanoparticles, has still not been reached.

Concerning the uptake *via* the roots (Fig. 6), natural absorption processes of the plant can be exploited for introducing molecules by the hydroponic system, which allows systemic distribution in the vascular system.^389,390^ The initial barrier for transport through plant roots is the root exudate layer, a layer of organic compounds released by plant roots into the soil consisting of low-molecular-weight compounds (such as amino acids, sugars, organic acids, phenols, and secondary metabolites) and high-molecular-weight substances (primarily mucilage and proteins).^391^ The exact influence of exudate composition on the uptake of substances, particularly nanoparticles, is not well understood and further complicated by species-specific variations. After traversing the exudate layer, substances encounter the root epidermis and cortex before reaching the endodermis. At this stage, the Casparian strip,^392^ a suberin- and lignin-rich structure encircling endodermal cells, forms a critical barrier, restricting the apoplastic transport. Although the Casparian strip theoretically impedes nanoparticle passage, some studies have reported successful translocation across this barrier.

Even after substances have successfully entered the plant *via* foliar or root uptake, they still face the subsequent challenge of internalization by the targeted plant cells (Fig. 6), representing the next critical hurdle in the delivery process. Structurally, the plant cell wall is a dynamic fibrous composite material that is essential for the maintenance of cell shape, mechanical stability and regulated growth.^393^ Particularly, primary cell walls, produced during cytokinesis, are thin (0.1–1 μm) and flexible, composed mainly of cellulose microfibrils embedded within a hydrated matrix of pectins and cross-linking glycans.^394,395^ These components are primarily interconnected through hydrogen bonds, forming a coherent network that facilitates cell expansion driven by turgor pressure. The resulting structure is porous, with pore sizes, although dependent on species, typically around 13 nm, and a common size exclusion limit for nanomaterials is in the range of 5 to 20 nm.^396^ The cell wall carries a negative charge, and electrical gradients across it can range from −50 mV to −100 mV,^397^ which is substantial enough to hinder the transport of charged substances. Once growth is complete, many cells reinforce their primary walls by depositing a secondary wall that is enriched with cellulose and lignin, a phenolic polymer that provides additional stiffness and hydrophobicity, particularly in xylem and sclerenchyma tissues.^398^ The passage of molecules and nanoparticles therefore initially encounters a passive selective barrier in terms of size and charge, before encountering the plasma membrane of the cell. The plasma membrane, composed of a lipid bilayer embedded with proteins, constitutes a selective barrier that generally requires active transport mechanisms, such as endocytosis, for translocation.^399,400^ Following internalization, particles may be directed to vacuoles or endosomes, where they can be subject to degradation or sequestration.^401^ Consequently, efficient intracellular delivery may depend on the ability to escape endosomal compartments, a process that remains not fully understood in plant cells. Additionally, intracellular enzymes can degrade small organic molecules or functional groups present on nanoparticle surfaces, posing another challenge for effective delivery.

A relatively straightforward approach to achieving more precise delivery into individual cells or tissues involves injection methods, in which fine needles are used to directly introduce substances, in addition to spraying or spot application.^402,403^ In particular, microinjection allows precise delivery into individual cells or tissues using fine needles,^404,405^ but it is technically demanding and can cause tissue damage. In contrast, electroporation introduces molecules directly into cells by using electrical pulses to create temporary pores in cell membranes.^406,407^

Another method, particularly used for delivering nucleic acids, relies on biolistic particle delivery instead,^408^ in which the cargo is loaded onto microscopic particles and physically ‘shot’ into plant tissue or cells using high-pressure gas or an electrical discharge. The downsides of the latter two techniques are the need for specialised equipment and, in some instances, careful optimisation to avoid cell damage. Additional methods, such as pressure-driven infiltration and (v) grafting techniques, are also used, each with unique advantages and limitations. The choice of the delivery method depends on factors such as the properties of the molecule, the plant species, the tissue type and the specific research objectives.

However, the presence of the previously mentioned plant cell wall poses an additional physical barrier for the intracellular delivery of biomolecules, which thus currently lacks an efficient and passive method of long-distance transports into a broad range of plant phenotypes and species without the aid of external force and without causing tissue damage.^409^ For this reason, nowadays great attention has been given to the phloem tissue, which represents an efficient network of plumbings, facilitating the loading, transport, and subsequent unloading from source to sink tissues.^410^ Therefore, it is essential to consider the crucial role that cell walls play in enabling the high-pressure flow of photoassimilates through the sieve elements.^410^

More generally, for nanoparticles whose uptake is strongly influenced by their size and the porosity of various plant membranes, several strategies have been developed for their delivery and interfacing with plants: (i) protein-based nanosensors are introduced into plants by translocating their corresponding nucleic acid sequences, such as plasmid DNA, into the plant cells.^411,412^ In other instances, nanoparticles are transferred to plant leaves by various infiltration methods (*e.g.*, infiltration through leaf laminae)^413^ (ii) by direct injection, or (iii) *via* soil drenching techniques.^414^ Nevertheless, it should be noted that the transport of nanomaterials remains an active area of research aimed at elucidating the mechanisms underlying their traversal across plant barriers and cellular uptake.^247,383,415–417^ These processes are still largely unexplored, with many elusive mechanisms yet to be discovered. Factors such as nanoparticle size, shape, zeta potential, surface chemistry, and the formation of protein coronas in plants must be further researched to advance this field.

While the aforementioned transfection methods effectively introduce nanoparticles into plants, significant concerns persist regarding their toxicity and, therefore, their impact on plant health metabolism. The future application of supramolecular chemosensors, probes, and nanosensors in plants requires standardized transfection procedures and nanoparticle analysis methods, as well as consistent and comparable environmental conditions for plant-related experiments.^418–420^

It is important to note that plant cell walls, regarded as the most likely route for nanoparticles to penetrate plant cells, possess pores measuring between 5 and 30 nm. Therefore, future nanoparticles should be synthesised to be smaller than this 30 nm threshold.^421,422^ Interestingly, a recent study has shown that cellular internalisation of nanoparticles is not necessarily required for RNA transport into mature plant leaves.^423^ Specifically, small interfering RNA (siRNA)-functionalised gold nanoparticles (AuNPs) of varying sizes and shapes were tested for uptake in *Nicotiana benthamiana* cells, and surprisingly, 10 nm spherical AuNPs already achieved 99% efficiency in cell penetration, suggesting that the particles associate with the cell wall and gradually release their siRNA cargo.

Unlike genetically encoded biosensors, introducing plasmonic nanoparticles into living plants is not as straightforward, as it necessitates invasive delivery methods, such as injections. However, since metal NPs are relatively more stable than protein-based nanoparticles, they can be drop-casted or attached *via* a patch onto the surface of plant parts, where they can be used to detect analytes such as volatile organic compounds (VOCs), for example, through SERS detection.^424^ An alternative approach for introducing metal nanoparticles into plants relies on the activity of enzymes that have been shown to produce the corresponding metal nanoparticles in the presence of transition metal salts.^425^ Thus, oxidases, such as glucose oxidase, reduce oxygen to H_2_O_2_ upon oxidation of the corresponding substrate, while the resulting hydrogen peroxide reduces [AuCl_4_]^−^ to Au^0^, leading to the formation of AuNPs in plants and on plant surfaces.^426,427^

### 1.7 Plant metabolites, pesticides and nucleic acid-based pesticides

**Fig. 7:**
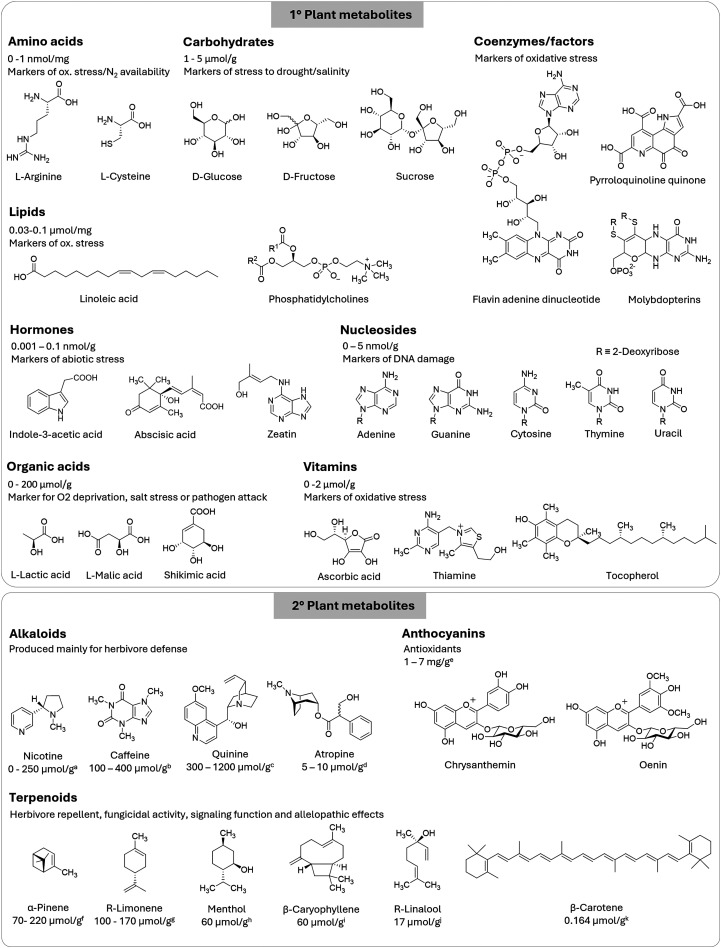
Chemical structures of exemplary primary and secondary plant metabolites and their respective roles in plants. For the primary metabolites, the concentration levels represent a general average typically found in *Arabidopsis thaliana*. Concentration levels for secondary metabolites are averaged across various plant species: (a) tobacco plant; (b) green tea leaves; (c) *Cinchona* bark; (d) *Atropa belladonna*; (e) fruits; (f) *Rosmarinus officinalis* leaves; (g) lemon peels; (h) oil of *Mentha canadensis*; (i) *Piper nigrum*; (j) basil; (k) potato leaves.

Plant metabolites can be divided into primary and secondary metabolites. The former are directly involved in processes that are important for the homeostasis of the plant, such as growth, development and reproduction, while the latter mainly serve for defence purposes, signalling and interaction with the environment. Primary metabolites include amino acids,^428^ carbohydrates,^429^ coenzymes/factors, lipids,^430^ phytohormones,^431,432^ nucleosides,^433^ organic acids^434^ and vitamins (Fig. 7).^435^

Secondary metabolites include alkaloids, nitrogen-containing plant compounds involved in defence against herbivores and pathogens (*e.g.*, nicotine, caffeine, quinine, atropine),^436^ anthocyanins^437^ and terpenoids (*e.g.*, α-pinene, limonene, menthol, caryophyllene or β-carotene),^438^ which play a role in plant defence, growth regulation and communication.

It is clear that, in plant science, the development of sensor technologies, pesticides and nucleic-acid-based pesticides is key for improving the resilience of plants to external stress factors (*e.g.*, pests, diseases, environmental conditions) and increasing crop yields. Thus, monitoring stress-related metabolites, such as alkaloids, terpenoids, proline, abscisic acid, and reactive oxygen species (ROS), can provide real-time insights into plant responses. This promotes sustainable agricultural practices by improving resource efficiency, reducing pesticide usage, and aiding in the development of new strategies to enhance plant resilience to ongoing climate change. Hormone levels, such as auxins, cytokinins, and gibberellins, play a crucial role in regulating growth. At the same time, monitoring nitrogen, including amino acids and nitrate, as well as phosphorus metabolism, such as phosphate esters, offers insights into the nutritional status of the organism plants.

Pesticides are chemical substances or biological agents used to control, repel, or destroy pests that damage crops, livestock, or other valuable resources. Particularly, pests include insects, weeds, fungi, rodents, and microorganisms such as bacteria and viruses that can harm plants or transmit diseases. For this reason, pesticides are classified into various categories, such as insecticides, herbicides, fungicides, rodenticides, and bactericides. Globally, approximately three million tons of pesticides are used annually to control pests, according to estimates by the Food and Agriculture Organization of the United Nations,^439^ with usage following an increasing trend.

**Fig. 8:**
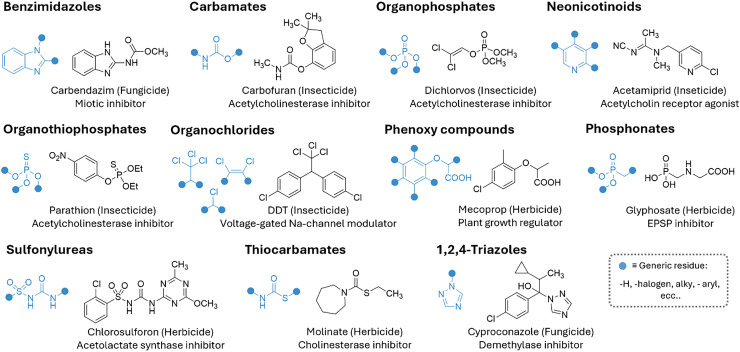
Representative list and chemical structures of the most common pesticides.

From a chemical perspective, pesticides can be classified into organophosphates, phosphonates organochlorines, (thio)carbamates, neonicotinoids, phenoxy acetic acids, triazines, sulfonylureas, and benzimidazoles, among the most prominent classes (see Fig. 8).

The production, use, and disposal of pesticides have serious disruptive consequences for ecosystems, making it crucial to reduce their usage by developing more effective pesticides that require smaller quantities or by discovering new, more selective alternatives.^440^ This effort must also ensure that pesticide production remains cost-effective while minimizing its ecological impact. Efficient application of pesticides and nutrients is indeed crucial for effective plant protection. Nowadays, conventional methods such as foliar spraying, soil application, and fertilization face several challenges, such as low efficiency, environmental pollution through run-off and accumulation in the soil, non-specific action on insects and mammals, and long retention times.

**Fig. 9:**
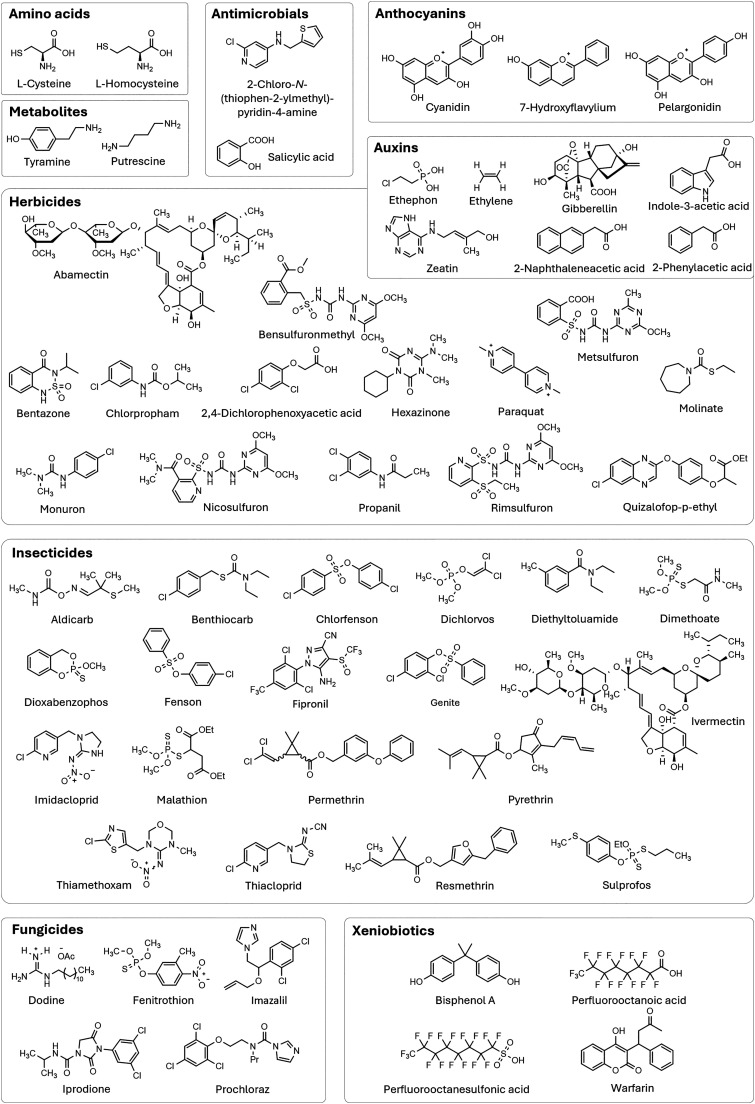
Representative examples of pesticides and plant metabolites discussed in this review.

Furthermore, classic pesticides (reported in Fig. 9) pose a significant health risk to humans, mammals and the environment, whether through acute or chronic exposure. For example, 1,1′-(2,2,2-trichloroethane-1,1-diyl)bis(4-chlorobenzene) (DDT), which was once hailed as a “wonder pesticide”, is now banned in most countries due to its toxicity. Tetrachloroetherphthalate (DCPA) was banned by the EPA in 2024 because of its associated health hazards, following its use since its introduction in 1998.^441^ Although neonicotinoids were initially regarded as a safer alternative to organophosphates, early studies indicate that they may lead to respiratory, cardiovascular, and neurological issues, along with genetic damage and birth defects.^442^ However, more research is needed to fully characterize their toxicity profiles in mammals.

In this context, given the toxicity of traditional pesticides, RNAi-based pesticides could offer a safer and more targeted alternative. Specifically, RNAi works by disrupting mRNA transcription, stability, and translation through argonaute family proteins and small RNAs, such as siRNA^443^ and microRNA (miRNA),^444^ thereby reducing gene expression and function.^445^ RNAi technology^446–448^ has been successfully tested across various crops and trees for protection against insect damage:^449–452^ these RNA-based pesticides are considered safer in general because they specifically target the genes of the pests. One approach to RNA pest control is the exploitation of genetically engineered plants that produce RNA to disrupt key genes of pests. However, this method is inefficient due to the time, cost and regulatory challenges involved in developing genetically modified plants. Therefore, more effective strategies for RNA delivery, such as sprayable formulations, are needed for faster and more cost-effective applications.

In addition, major challenges in RNA pesticide delivery include degradation by RNA-degrading enzymes and difficulties with cellular uptake, such as entrapment in the endosome.

## 2. Supramolecular sensors

**Fig. 10:**
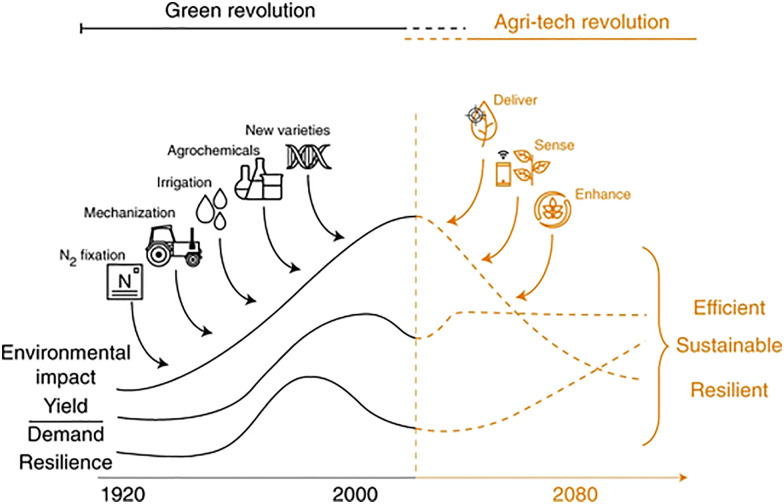
The green revolution and the new agritech revolution. Image adapted with permission from ref. 455.

Global food production has more than tripled over the past half-century;^453^ the “green revolution”, which began in the 1960s,^454^ has successfully delivered year-over-year yield gains with minimal expansion of land use, as illustrated in Fig. 10.^455^ However, global food demand is projected to increase by 35% to 56% between 2010 and 2050,^456^ necessitating a corresponding growth in global crop productivity. Therefore, exploring and understanding plant-environment interactions is crucial for ensuring crop production and food security, which are fundamentally tied to national development, social stability, and self-reliance.

In this context, supramolecular chemistry has increasingly gained attention due to its potential to drive advances in sensing and delivery technologies. The use of tools such as (supra)molecular probes and chemosensors offers transformative possibilities for the detection and delivery of bioactive molecules, especially when integrated into innovative assay methodologies.^141,457,458^ Currently, state-of-the-art methodologies like tissue staining,^459^ high-performance liquid chromatography coupled with mass spectrometry (HPLC-MS), gas chromatography coupled with mass spectrometry (GC-MS), and nuclear magnetic resonance spectroscopy (NMR) are widely used for biological sample analysis. However, despite being highly sensitive, these techniques require large, complex equipment, limiting their use for on-site detection. In recent decades, molecular detection techniques based on supramolecular principles, such as the use of synthetic macrocyclic hosts, have led to diverse and promising advancements in sensor technology. These advancements could eventually complement biological components and processes, such as immune-based diagnostics (antibody–antigen interactions),^460,461^ genetically engineered receptor proteins,^462,463^ DNA and RNA technologies,^464^ including polymerase chain reaction (PCR) tests,^465^ and enzymatic reaction-based diagnostics.

The ability to locate, track and analyse plant metabolites, pesticides and pollutant is as critical as assessing their persistence in the environment. These challenges highlight the need for advanced analytical tools that go beyond conventional methods such as HPLC, MS and immunoassays, which are often costly and lack the live imaging capabilities required for real-time monitoring. The integration of more advanced tools would not only help biophysicists to develop predictive *in silico* models for (nano)pesticides, but would also significantly expand the fundamental knowledge in this field. Therefore, the use of chemosensors (working through host–guest interactions) offers promising tools for the development of new, fast and cost-effective sensing technologies that can be easily integrated into miniaturizable devices.^466–469^ In addition, dynamic processes, *e.g.*, the uptake, distribution, accumulation and elimination of bioactive molecules in the environment, plants and soil, could be efficiently monitored with these systems.

A representative list of pesticides, phytohormones and other analytes of significant environmental- and human health-related concern reported in this chapter is delineated in Table 3, together with their advisory safe limits and their typical concentrations in planta.

**Table 3:** A representative list of the analytes reported in this review, with the related achieved LoDs and advisory safe/maximum residue limits and in planta concentrations

Analyte	Analyte classification	Advisory safe limits	Common analyte content in planta	LoDs reported in this review
1-Naphthalene acetic acid (NAA)	Herbicide	0.05–0.10 ppm in food^470^	—	8.20 μM^471^
2,4-Dichlorophenoxy acetic acid (2,4-D)	Herbicide	0.317 μM in drinking water^472,473^	—	0.35 μM^471^
Abscisic acid (ABA)	Phytohormone	—	1 nmol g^−1^ in *Pinus sylvestris*^474^	0.098 nM^475^
Aluminium ion	Herbicide	148–371 μmol g^−1^ dry weight for roots inhibition in old leaves of *Oryza rufipogon*^476^	—	10.0 μM (even as low as 37 nM)^477^
Aniline	Pollutant	2.68 μM in water^478^	—	0.05–0.50 μM^479^
Bentazone	Herbicide	104 nM in water^472^	—	54.1 nM^480^
Cadmium ions	Toxic metal ions	0.0702 μM in water^481^	—	0.044 μM^479^
Carbendazim (CBZ)	Fungicide	0.523 μM in water^472^	—	0.17 μM^482^
Chlormequat (CQ)	Herbicide	8.64 μmol kg^−1^, *e.g.*, in table grapes^483^	—	1.75 μM^484^
Copper ions	Pollutant	31.5 μM in water^485^	—	9.40 μM,^486^ 20.4 μM^479^
Cysteine (Cys)	Metabolite	—	12.4–16.5 μmol g^−1^ in *Triticum aestivum*, *i.e.*, wheat^487^	2.31 μM^488^
Difenzoquat (DFQ)	Herbicide	0.401 μM in water^472^	—	0.25 μM^484^
Dihydrogen phosphate (H_2_PO_4_^−^)	Fertiliser	2060–5150 nM in water^489^	—	33.0 nM^490^
Diquat (DQ)	Herbicide	2.71 μM in drinking water^472^	—	1.15 μM^484^
Dodine (DD)	Fungicide	5.22 μmol kg^−1^ in food^491^	—	1.83 μM^492^
Ethylene (gas)	Phytohormone	—	Tens of ppm^493^	∼27.0 ppm in air^494^
Fipronil (FPN)	Insecticide	11.4 nM in water^472^	—	∼22.0 nM^495^
Fuberidazole (FBZ)	Fungicide	54.3 μmol kg^−1^ in cereals grain^496^	—	0.13 μM^482^
Glucose	Metabolite	—	—	50 nM to 250 nM^497^
Homocysteine (Hcy)	Metabolite	15.0 μM^498^	—	4.67 μM^488^
Hydrogen peroxide (H_2_O_2_)	ROS	—	0.20–1.00 μmol mg^−1^ (ref. 499)	10.0–100 μM^500^
Imazalil	Fungicide	33.6–1680 μmol kg^−1^ in food^501^	—	43.7 μM^480^
Imidacloprid	Pesticide	7.8 μM in water^472^	—	50.0 μM^480^
Iron ion	Toxic metal ion	17.9 μM in water^502^	—	0.05–10.0 μM^479^
Mepiquat (MQ)	Herbicide	87.5 μmol kg^−1^ in mushrooms^503^	—	0.90 μM^484^
Nicosulfuron	Pesticide	24.4 μmol kg^−1^ in sweet corn and maize grain^504^	—	31.0 μM^480^
Paraquat (PQ)	Herbicide	3.89 μM in water^472^	—	0.80 μM^484^
Perchlorate (ClO_4_^−^)	Pollutant	563 μM in water^505^	—	Down to 100 nM^506^
Perfluorooctane sulfonic acid (PFOS)	Pollutant	0.0080 nM in water, advisory safe limit^507,508^	—	0.20 nM, only in specific cases down to 0.020 nM^507,508^
Perfluorooctanoic acid (PFOA)	Pollutant	0.242 nM in drinking water^509^	—	0.242 nM, only in specific cases down to 0.0242 nM^507,508^
Putrescine	Metabolite	—	From 10.2 μmol kg^−1^ to 6230 μmol kg^−1^ in several food products^510^	Down to 26.0 μM^511^
Quizalofop-*p*-ethyl	Herbicide	536 nM in water^472^	—	29.8 nM^512^
Rimsulfuron	Pesticide	0.0232 μM in food^513^	—	30.0 μM^480^
Salicylic acid (SA)	Phytohormone	—	—	4.00–20.0 nM^514,515^
Silver ions	Toxic metal ions	0.0176 μM in saltwater^516^	—	0.46 μM^479^
Sodium chloride (NaCl)	Environmental stressor	—	≥13.7 mM^517^	170 μM and 448 μM^518^
Thiabendazole (TBZ)	Preservative, parasiticide, fungicide	1.99 μM in water^472^	—	0.12 μM^482^
Thiacloprid	Insecticide	0.0791–39.6 μmol kg^−1^ in food^519^	—	30.0 μM^480^
Thiamethoxam	Insecticide	0.0343 μmol kg^−1^ in food^520^	—	30.0 μM^480^
Thidiazuron (TDZ)	Preservative	Banned in 2008 for use in agriculture^521^	—	0.12 μM^482^
*trans*-Zeatin	Phytohormone	—	<100 pmol g^−1^ in *Arabidopsis thaliana* leaves and roots (upon heat stress)^522^	3.00 nM^523^
Tricyclazole (TCZ)	Fungicide	0.529 μM in water^472^	—	0.26 μM^482^
Tyramine	Metabolite	No specific criteria in EU legislation^510^	—	Down to 190 μM^511^
Zinc ions	Plant nutrient	1.38 μM in water^516^	—	56.0 nM^524^

### 2.1 Design features for luminescence-based sensors

The majority of chemosensors and nanosensors discussed in this section rely on a luminescent signal readout. With this in mind, it may be beneficial for the reader to begin with an overview that highlights key design considerations essential for mitigating potential pitfalls in luminescence-based detection methods, while also providing guidelines for best practices in setting up fluorescence-based probes and chemosensor assays.

It is a matter of fact that the widespread use of luminescence-based sensors is largely justified by their high sensitivity, ease of instrumental setup, and cost-effectiveness. However, despite these advantages, obtaining reliable and quantitatively relevant data requires careful attention to data acquisition and analysis. Unlike spectrophotometric measurements, where the outcoming electrical signal can be expressed on an absolute scale, being directly related to the absorbance of the investigated sample, spectrofluorimetric signals represent the total luminescence intensity (*i.e.*, the total number of emitted photons) of the considered sample. This intensity is thus related to the outcoming signal through both instrumental factors and the characteristics of the measured solution itself, making a direct correlation with sample concentration a non-trivial challenge. Therefore, appropriate corrections must be applied to the obtained data to ensure an accurate quantification.

In this section, we focus on the practical steps involved in setting up a chemosensor assay, rather than on the instrumental corrections required for precise luminescence measurements. For readers interested in the latter, we refer to comprehensive discussions on correcting instrument sensitivity errors, including those arising from diffraction grating effects and the spectral response of the detector itself.^525,526^

Before setting up the measurement conditions for a chemo- or nanosensor, its stability in aqueous media and complex biological fluids must be thoroughly characterised; molecular probes and chemosensors should resist decomposition (*e.g.*, hydrolysis) and remain unaffected by interferents in challenging media, such as protein-rich fluids or contaminated water containing unexpected pollutants like microplastics. Similarly, nanosensors must retain their structural integrity over time, ensuring that both the nanomaterials and their functional groups do not degrade through chemical decomposition, dissolution, aggregation, or precipitation.

Additionally, studies evaluating sensing performance should include titrations covering the full biologically and practically relevant range of analyte concentrations, from complete absence to stoichiometric excess; when sensing is based on analyte-probe association, intensity *vs.* concentration plots should exhibit a plateau. The following considerations must also be taken into account:^527^

• Inner filter effects (IFEs): IFEs occur whenever the analyte significantly absorbs at the selected excitation and/or emission wavelengths, leading to a decrease in observed intensity without any actual interaction between the probe and analyte. Therefore, intensity values must be properly corrected for IFEs before any further data processing.^528,529^ Furthermore, in our view, luminescence probes relying on IFEs are unsuitable because any chemical species absorbing at these wavelengths can interfere. On the other hand, any species with absorption or luminescence overlapping with the target analyte's absorption could be used non-specifically as a probe.

• Data fitting: the intensity *vs.* concentration relationship should be analysed using appropriate software, avoiding linearization methods such as the Benesi–Hildebrand equation, which relies on oversimplifications that are rarely accounted for.

• Stern–Volmer plots: plots of *I*°/*I* against analyte concentration (or its reciprocal, *I*/*I*°) must, by definition, have an intercept of 1. Significant deviations from this value are unacceptable. For a detailed discussion on luminescence quenching, we refer readers to ref. 530.

• Sensor benchmarking: the probe's performance should be compared to state-of-the-art systems for the same analyte, with any differences in experimental conditions (*e.g.*, solvent system) clearly specified. A well-justified set of potential interfering substances should be tested, and the results carefully analysed and interpreted.

• Limits of detection (LoDs): LoDs should be calculated using experimental data at comparable concentrations and adequately supported by evidence.

Many chemosensor examples rely on calculating the LoDs^531^ by either a blank-based or a calibration curve method. The blank-based method is simple and widely accepted but is sensitive to noise variability. The calibration curve method, typically using the 3-sigma criterion (*i.e.*, the concentration producing a signal three times the noise standard deviation), is statistically robust but assumes linearity and requires careful regression analysis with sufficient replicates and error propagation. However, because supramolecular chemosensor responses may deviate from linearity at low concentrations, where saturation, rather than noise, becomes limiting, it is often preferable to report the lowest analyte concentration analysed in a standard sample instead of the calculated LoD. The reported LoD should also consider the binding affinity for the analyte to ensure consistency. For example, if the LoD is orders of magnitude lower than the chemosensor's reported binding affinity for the analyte, this discrepancy should be carefully evaluated. For guidance on reporting LoD, please also refer the reader to the guidance document on the estimation of LoD and limit of quantification (LoQ) for measurements by the EU.^532^

There are many parameters that could guide the design of chemosensors for plant applications, including the ease of their synthesis, and the many examples that we are reporting in this reviews witness a diversity of approaches. However, designing an effective system should be guided by its specific application. In this context, we would like to underline here that it is crucial selecting the appropriate receptor/reporter combination, making use of the binding affinities (see Tables 3 and 4), as it determines its useful dynamic range^533^ in which the system can deliver optimal sensitivity and selectivity. This range should in fact ideally align with the most relevant concentration levels, such as those near advisory safety thresholds, in the conditions, such ionic strength and presence of interferents, that would be met in the field. Their use in aqueous solution or on suitable solid supports would be also crucial for their final application, while, in case of IDAs, the use of unimolecular systems could simplify the assay's architecture, and thus its reproducibility, ease, and cost.

**Table 4:** Collection of host–guest systems, analytes, related binding affinities, LoDs and LoQs reported in this chapter. Reported are the analytes with the related binding affinities in brackets: (—) indicates no binding affinity given

Macrocyclic hosts		Dye or reporting molecule (binding affinity)	Analytes (binding affinity)	LoDs	LoQs	Ref.
Cyclodextrins	β-CD	Aggregation-induced emission-enhancing organic molecules, AIETPA (3.20 × 10^4^ M^−1^)	*trans*-Zeatin (—)	3.00 nM	—	523
Calix[*n*]arenes	SC4	4′-Hydroxy-10-methylpyranoflavylium dye^a^ (1.34 × 10^4^ M^−1^, SC4; 4.85 × 10^3^ M^−1^, SC6; 8.41 × 10^4^ M^−1^, SC8)	Putrescine (—), tyramine (—)	0.080 mM (SC4, Putr); 0.350 mM (SC6, Putr); 0.0260 mM (SC8, Putr); 1.47 mM (SC4, Tyr); 1.79 mM (SC6, Tyr); 0.19 mM (SC8, Tyr)	0.026 mM (SC4, Putr); 1.17 mM (SC6, Putr); 0.085 mM (SC8, Putr); 4.90 mM (SC4, Tyr); 5.97 mM (SC6, Tyr); 0.62 mM (SC8, Tyr)	511
	SC6					
	SC8					
	SC5A	LCG for SCAs (—), AlPcS_4_ for QAAC4A (—)	Nicosulfuron (—), rimsulfuron (—), bentazon (—), imazalil (—), thiamethoxam (—), thiacloprid (—), imidacloprid (—)	—	—	480
	SAC4A					
	SAC5A					
	QAAC4A					
	SC5A					
Cucurbit[*n*]uril	CB8	Thioflavine T (10^6^ M^−1^)	Fuberidazole (FBZ, 2.45 × 10^6^ M^−1^); thiabendazole (TBZ, 2.79 × 10^6^ M^−1^); carbendazim (CBZ, 1.13 × 10^6^ M^−1^); thidiazuron (TDZ, 9.19 × 10^5^ M^−1^); tricyclazole (TCZ, 1.18 × 10^6^ M^−1^)	1.25 × 10^−7^ M (FBZ); 1.71 × 10^−7^ M (CBZ); 1.17 × 10^−7^ M (TBZ); 1.22 × 10^−7^ M (TDZ); 2.60 × 10^−7^ M (TCZ)	—	482
	CB10	Acridine (—)	Dinotefuran (—), oxadixyl (—), penconazole (—), thiamethoxam (—), carbaryl (—), flutriafol (—), acetamiprid (—), ethiofencarb (—), flusilazole (—), pyroquilon (—), pymetrozine (—), triadimefon (—), dodine (—), azaconazole (—), tricyclazole (—), metalaxyl (—), tebuconazole (—), paraquat (—), pyrimethanil (—), triadimenol isomer A (—)	1.83 × 10^−6^ M (dodine)	—	492
	CB7	Adamantane (AD)-modified rhodamine derivative, RAD (3.40 × 10^4^ M^−1^)	Salicylic acid (—)	10.0 nM	—	515
	CB8	3-(2-*N*-Methylbenzimidazolyl)-7-*N*,*N*-diethylaminoc coumarin, S1 (—); 3-(2-benzimidazolyl)-7-(diethylamino)coumarin, S2 (—)	Paraquat (PQ, log *K*_a_ = 4.15 (S1) or 3.96 (S2)); diquat (DQ, log *K*_a_ = 3.49 (S1) or 3.85 (S2)); difenzoquat (DFQ, log *K*_a_ = 5.64 (S1) or 4.59 (S2)); chlormequat (CQ, log *K*_a_ = 3.65 (S1) or 3.67 (S2)); mepiquat (MQ, log *K*_a_ = 4.64 (S1) or 4.27 (S2))	0.80 μM (PQ); 1.15 μM (DQ); 0.25 μM (DFQ); 1.75 μM (CQ); 0.90 μM (MQ)	—	484
Pillar[*n*]arenes	RD-P5	—	Perfluorooctane sulfonic acid (PFOS, 2.60 × 10^6^ M^−1^); perfluorooctanoic acid (PFOA, 5.20 × 10^4^ M^−1^)	0.20 nM (PFOS, only in specific cases down to 0.020 nM); 0.242 nM (PFOA, only in specific cases down to 0.0242 nM)	—	507

a Determined *via* UV-vis spectroscopy at pH = 10.

### 2.2 Host–guest chemistry based chemosensors

As mentioned previously, supramolecular chemistry has enabled several host–guest systems over the last few decades, useful for various applications, *e.g.*, sensing,^113,141,534^ catalysis,^535,536^ smart materials, switches,^537^ and medicine.^538^ The nature of the non-covalent interactions involved in the complexation between host and guest molecules has been discussed in Section 1.2. Therefore, this section will focus on the application of the macrocyclic compounds considered, namely CDs, CX*n*, CB*n*, and PA*n*, in the detection of pesticides and plant phytohormones. It should be noted that such systems have no useful optical properties on their own; therefore, they are traditionally used for molecular recognition in the design of a chemosensor and are associated with a signalling component, which is typically an indicator dye that changes its photophysical properties (primarily luminescence) in the presence of a wide range of analytes. This results in luminescent probes capable of producing specific changes in their responses (*i.e.*, turn-off, turn-on, or ratiometric features), achieving suitable detection limits.

Among the various available signal transduction mechanisms, one of the simplest and most widely used involves the displacement of a reporter dye from the macrocycle, which serves as the fundamental operating principle of IDAs. As will be highlighted, there is significant room for improvement in the design of new chemosensors for in planta and on planta applications. Therefore, this subchapter provides a critical summary of examples that hold potential for future applications in and on living plants. Table 4 contains a summary of the chemosensors discussed here based on host–guest sensors and their performance.

#### 2.2.1 Cyclodextrin-based chemosensors

CDs are macrocyclic hosts known for binding a variety of pesticides and fluorophores,^539^ making them useful for setting up chemosensors with potential applications in agriculture and public health research.

**Fig. 11:**
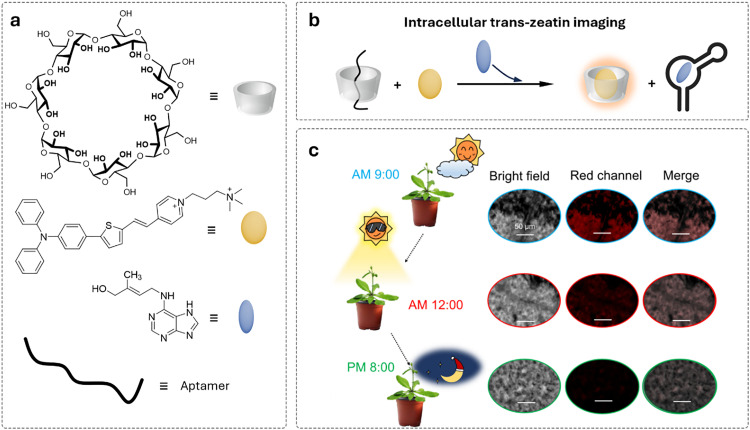
(a) Chemical structures of βCD, AIETPA, *trans*-zeatin, and a schematic representation of the *trans*-zeatin-selective aptamer. (b) Working principle of fluorescent intracellular *trans*-zeatin imaging: binding of the aptamer to *trans*-zeatin reduces its affinity for the host, allowing it to be displaced by the AIETPA dye, which exhibits enhanced fluorescence upon forming a host–guest complex with the macrocycle. (c) Fluorescence imaging of *trans*-zeatin bioactivity in wheat coleoptiles. Figure adapted with permission from ref. 523.

Recently, Niu, Chen and co-workers^523^ exploited a βCD to develop a supramolecular biosensor making use of aggregation-induced emission-enhancing organic molecules (AIETPA) for the *in situ* detection of the cytokinin *trans*-zeatin (Fig. 11a). *trans*-Zeatin is a key regulator of cell division, chloroplast development, and leaf senescence, found primarily at the site where cell division occurs,^540^ and it is critical for understanding plant stress responses to different agents, such as oxidative stress and pathogen presence. As the biocompatible βCD is with approx. 1 nm in diameter much smaller than 5 nm the size threshold, it can freely pass through the plant cell wall barrier and incubate within plant tissues, making it possible to perform fluorescence imaging in planta. The underlying mechanism, reported in Fig. 11b, is based on the competition between Apt, an aptamer able to bind the analyte, and AIETPA for preferential access to the βCD. More in detail, in the presence of *trans*-zeatin, Apt dissociates from βCD (*K*_a,βCD_ = 8.9 × 10^3^ M^−1^) and binds to *trans*-zeatin, resulting in a lower-affinity conformation that allows displacement by the dye AIETPA, which, at this point, has a higher affinity for the βCD cavity (*K*_a,βCD_ = 3.2 × 10^4^ M^−1^). As a result, the dye becomes confined within the macrocycle's cavity, enhancing the PLQY. The LoD for *trans*-zeatin was 3 nM, which is surprisingly low given the milli- to micromolar affinity of βCD for the target. Furthermore, fluorescence imaging of *trans*-zeatin bioactivity was successfully demonstrated (Fig. 11c), providing a visual, non-invasive alternative to traditional quantification methods. Importantly, AIETPA diffuses into plant cells, while βCD and the aptamer are internalised *via* vesicle transport. Therefore, the reported biosensor selectively imaged *trans*-zeatin and its riboside, confirmed through *in vivo* studies on *Arabidopsis thaliana* and in tobacco plants, allowing for real-time monitoring of cytokinin bioactivity.

#### 2.2.2 Calix[*n*]arenes-based chemosensors

CXs have been utilised in the development of supramolecular sensors^541–543^ and have been suggested for pesticide detection. However, to the best of our knowledge, this application has not been explored in living plants. Water-soluble *p*-sulfonato CX*n* (SC*n*) comprise a widely investigated subclass of receptor hosts,^544,545^ characterised by π-electron-rich cavities with multiple sulfonate groups. They display good binding ability (*K*_a_ up to 10^7^)^546,547^ and high selectivity toward various organic cations together with a good biocompatibility.

**Fig. 12:**
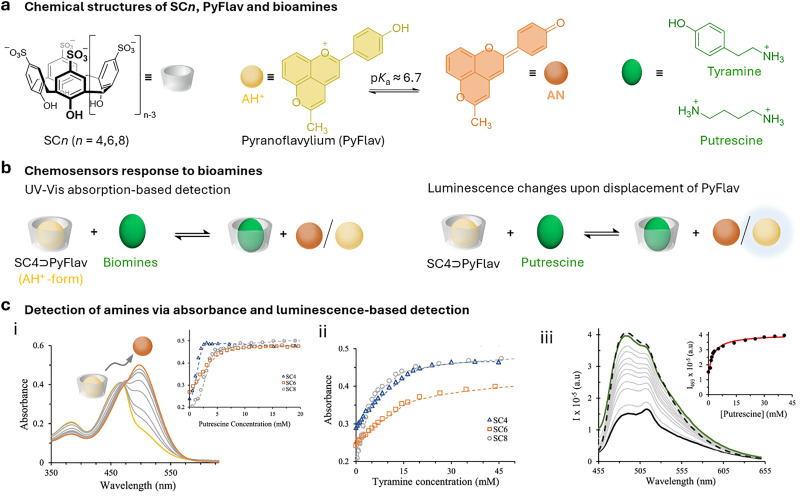
(a) Chemical structures of SC*n*, PyFlav in its protonated (AH^+^) and non-protonated (AN) forms, and the chemical structures of selected bioamines. (b) Schematic representation of the IDA principle for bioamine detection. Left: UV-Vis detection is enabled by the displacement of PyFlav, which shifts the equilibrium toward the AN form in the uncomplexed state, characterized by a distinctly more red-shifted absorption compared to the AH^+^ form. Right: Luminescence-based detection of putrescine is achieved through supramolecular displacement: only the unbound form of the dye AH^+^ released from the SC4·AH^+^ complex upon analyte binding exhibits strong emission, whereas the complexed state is effectively quenched. (c) (i) UV-Vis absorbance-based detection of putrescine using the SC4⊃PyFlav (2 mM) chemosensor in 10 mM PB at pH 7.2. The inset shows the change in absorbance at 500 nm as a function of increasing putrescine concentration for SC4⊃PyFlav, SC6⊃PyFlav (2 mM), and SC8⊃PyFlav (1 mM) chemosensors. (ii) UV-Vis absorbance-based detection of tyramine. (iii) Fluorescence-based detection of putrescine with the SC4⊃PyFlav chemosensor (*c*_PyFlav_ = 3.2 μM, *c*_SC4_ = 700.0 μM); *λ*_ex_ = 440 nm. Images adapted from ref. 511.

Basilio and co-workers^511^ developed a SC*n*-pyranoflavylium-based chemosensors (Fig. 12a) for the detection of bioamines, which are related to food quality, safety, and freshness. The operational principle of the chemosensor relies on the p*K*_a_ shift that the indicator dye (4′-hydroxy-10-methylpyranoflavylium, PyFlav) undergoes upon host complexation with different macrocycles, resulting in a p*K*_a_ change from 6.72 to 7.68 (SC4), 7.79 (SC6), and 8.45 (SC8). This p*K*_a_ shift drives host–guest complex formation (Fig. 12b), accompanied by measurable changes in the absorbance and emission properties of PyFlav. Under the assay conditions (10 mM phosphate buffer, pH 7.2 or 7.6), displacement of PyFlav from SC*n* host complexes by the bioamines putrescine (Putr) and tyramine (Tyr) induces a pronounced UV-vis absorbance change, characterized by a p*K*_a_-dependent bathochromic shift of the absorption maximum from 450 nm to 500 nm (Fig. 12c-i and ii). This shift reflects the transition from the protonated flavylium species (AH^+^) to the neutral quinoidal base (AN) upon release into the solution. Based on this, a ratiometric IDA using SC4, SC6, and SC8 in absorbance mode achieved millimolar-level limits of detection (see Table 4). In addition to UV-vis measurements, complexation of PyFlav with SC4, SC6, and SC8 under assay conditions led to a general decrease in luminescence intensity, attributed to excited-state electron transfer from the electron-rich phenolic units to the guest. Addition of bioamines restored the luminescence, enabling emission-based detection. For example, fluorescence detection of putrescine (Fig. 12c-iii) was demonstrated by monitoring intensity changes in a system containing PyFlav (3.2 μM) and SC4 (0.70 mM) with increasing putrescine concentrations at pH 7.2 in 10 mM phosphate buffer.

**Fig. 13:**
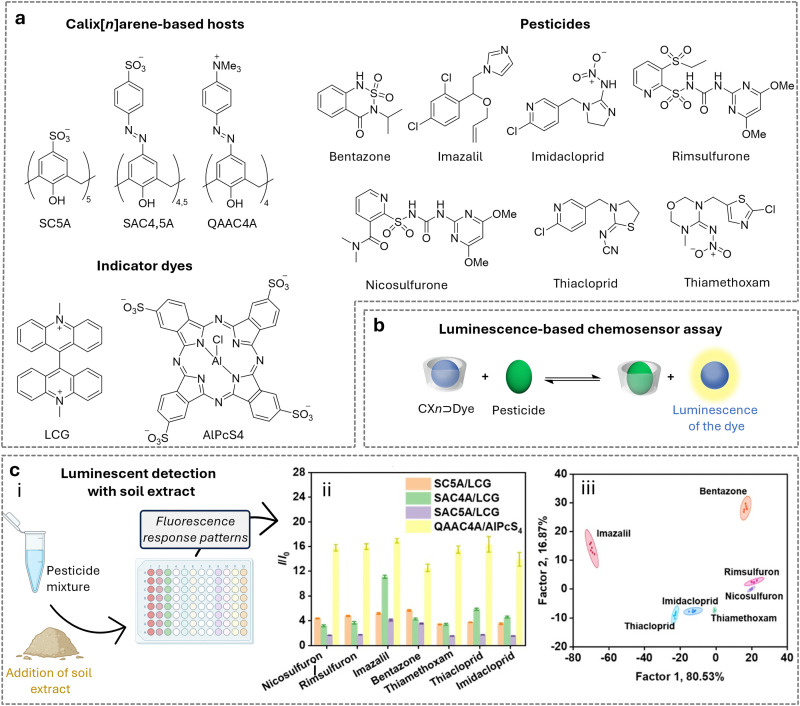
(a) Chemical structures of CX*n*-based host molecules, fluorescent dyes, and pesticide analytes. (b) Schematic illustration of the operating principle underlying the chemosensor assays employed for pesticide detection. (c) (i) Schematic representation of the operating principle, and (ii) fluorescence response patterns of the sensor array (*c*_CX*n*_ = 2.0 μM, *c*_dye_ = 2.0 μM) toward various pesticides in the presence of 20% soil extract. (iii) Canonical score plot derived from linear discriminant analysis of the fluorescence response patterns in the presence of 20% soil extract, including 95% confidence ellipses (*n* = 6). Adapted with permission from ref. 480.

A chemosensor assay based on SC5A and sulfonated azocalix[4]arene (SAC4A) as well as sulfonated azocalix[5]arene (SAC5A) and the quaternary ammonium-modified azocalix[4]arene (QAAC4A) was reported by D.-S. Guo and co-workers for the detection of seven tested pesticides (nicosulfuron, rimsulfuron, bentazon, imazalil, thiamethoxam, thiacloprid, imidacloprid), through an IDA (Fig. 13a and b).^480^ The presence of an azo group in the calixarene extended its elongated cavity, thus increasing its binding affinity for the target analytes.^548^ The resulting “off–on” fluorescence behaviour (Fig. 13c) and colour changes upon binding of pesticides were analysed using linear discriminant analysis (LDA), achieving 95% identification accuracy for 20 blind water samples, each containing 13.0 μg mL^−1^ of pesticide. The assay, which used calixarene (2.0 μM) and dye (2.0 μM), can be completed in 3 minutes and enables the accurate detection of imazalil concentrations in the range of 0–65.8 μM. Besides, when performing the detection in water containing an extract from soil (up to 20%), and thus containing some interferents found in soil, the detection of the pesticides was not compromised. Although the approach seems to be promising, further studies could still investigate its performance in undiluted soil samples. Furthermore, advances in machine learning could expand the array's ability to detect a wider range of analytes.

#### 2.2.3 Cucurbit[*n*]uril-based chemosensors

CB*n* are particularly interesting hosts for chemosensors, as they exhibit some of the highest binding affinities in water among all macrocycles.^138,141^ As for all macrocycles, CB*n* are optically transparent and not luminescent, having to interact with suitable dyes to form luminescent probes, which can represent useful sensors for the detection of single analytes or the discrimination and detection between multiple analytes (*e.g.*, a series of different pesticides). While potentially useful, to the best of our knowledge, no practical application of CB*n* in living plants has yet been reported.

**Fig. 14:**
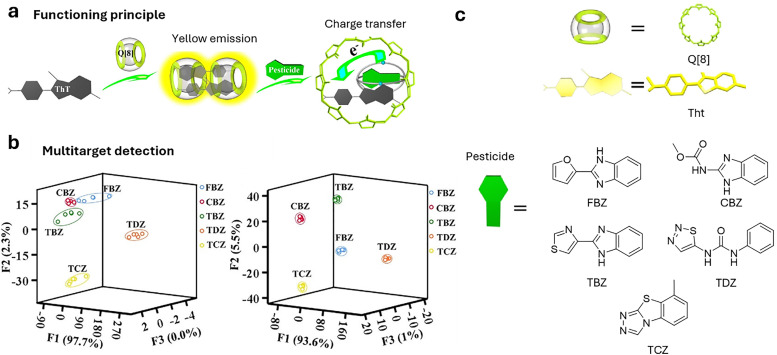
(a) Proposed mechanism of fluorescence quenching in the ThT@Q[8] system. Its application enables multitarget detection of five aromatic pesticides under single-wavelength excitation (*λ*_ex_ = 365 nm), including in a paper-strip-based assay. (b) Canonical score plot from LDA for the discrimination of pesticides in tap water (left) and Huaxi river water (right). (c) Cartoon representations of Q[8] and ThT, as well as the chemical structures of the tested pesticides. Adapted with permission from ref. 482.

For example, Huang and co-workers reported a “lab-on-a-molecule” fluorescent chemosensor assay,^482^ based on paper strip technology using a CB8-thioflavin T host–guest complex (ThT@Q[8], *K*_a,CB8_ = 10^6^ M^−1^) for the detection of pesticides such as fuberidazole (FBZ), thiabendazole (TBZ), carbendazim (CBZ), thidiazuron (TDZ) and tricyclazole (TCZ, Fig. 14a). For all of the considered analytes, low LoDs have been achieved, reporting values of 0.1–0.2 μM in ultrapure water containing DMSO (0.2% v/v). The gradual addition of Q[8] to the free ThT solution initially forms a 2 : 1 π-stacked ThT dimer–guest complex (2ThT@Q[8]), which emitted green fluorescence under UV irradiation. Further addition of Q[8] forms a 2 : 2 excimer complex (ThT@Q[8]), which exhibited yellow fluorescence and was selected as a fluorescent probe (Fig. 14b). Thus, after binding each different pesticide to form a ternary complex, the chemosensor shows distinct emission maxima shifts, including varying degrees of blue shifts and intensity changes for each of the five pesticides under UV-light excitation (at 365 nm). Paper strips impregnated with ThT@Q[8] (100 μM) were used to detect pesticides (100 μM) in real river water samples, whereby the ThT@Q[8] probe produced clear RGB colour reaction patterns in the presence of samples spiked with pesticides, and LDA analysis achieved 100% correct classification of the pesticides (Fig. 14c).

**Fig. 15:**
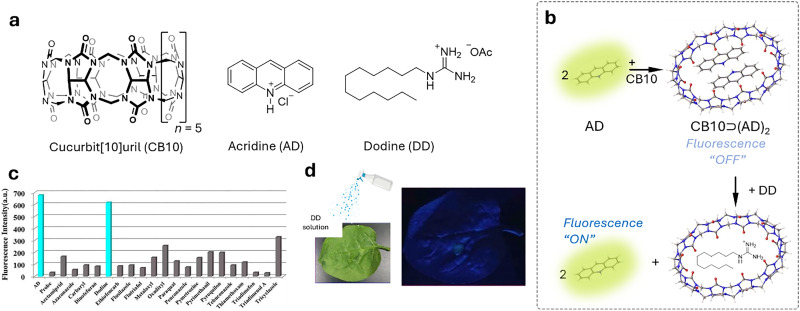
(a) Chemical structures of CB10, the indicator dye acridine, and the pesticide dodine. (b) Schematic representation of the fluorescence-based guest displacement assay, where the presence of a strongly binding analyte, such as dodine, enables the displacement of the indicator dye from the macrocycle's cavity. (c) Fluorescence response of 20 pesticides (10 equivalents of the host–guest complex) on the relative fluorescence intensity (*λ*_em_ = 472 nm) of CB10⊃(AD)_2_. (d) Photographs of *G. cusimbua* treated with dodine (5.0 × 10^−7^ M solution). Adapted with permission from ref. 492.

Another fluorescence turn-on chemosensor was reported in 2020 by Xiao, Liu, and co-workers,^492^ exploiting the quenching of acridine (AD) fluorescence upon the formation of a ternary host–guest complex with CB10 (CB10⊃(AD)_2_; Fig. 15a). In this way, it was possible to develop a rapid fluorescence-based displacement assay for detecting several pesticides (dinotefuran, oxadixyl, penconazole, thiamethoxam, carbaryl, flutriafol, acetamiprid, ethiofencarb, flusilazole, pyroquilon, pymetrozine, triadimefon, dodine, azaconazole, tricyclazole, metalaxyl, tebuconazole, paraquat, pyrimethanil, and triadimenol isomer A) in water at concentrations ranging from 0.0 to 4.0 × 10^−5^ M (with *c*(CB10⊃(AD)_2_) = 2.0 × 10^−5^ M, pH 4.0), as depicted in Fig. 15b. The detection limit for dodine was determined to be 1.83 × 10^−6^ M, thus the chemosensor was further applied to detect this pesticide on the surface of *G. cusimbua*, previously sprayed with a dodine solution (5.0 × 10^−7^ M), as well as on kidney beans. The presence of the pesticide on these surfaces was detectable *via* fluorescence recovery when exposed to UV light.

**Fig. 16:**
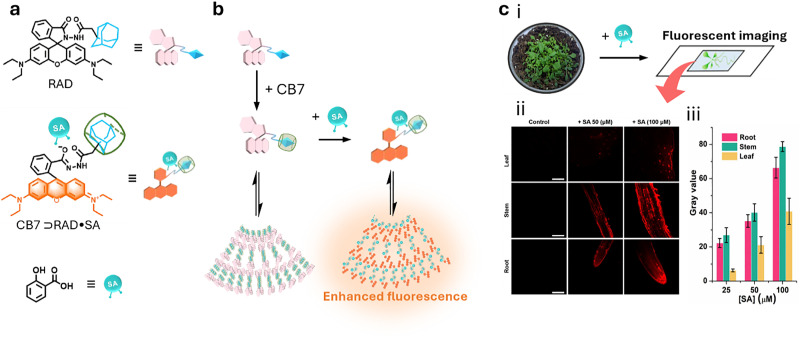
(a) Chemical structures of adamantane (AD)-modified rhodamine derivative (RAD), and when it is bound to CB7 and salicylic acid (SA). (b) Functioning principle of the chemosensor response to SA, highlighting the SA-induced spirolactam ring opening of RAD, which leads to the observed fluorescence enhancement. (c) (i) Schematic diagram of the CB7⊃RAD chemosensor for SA detection in *Arabidopsis thaliana*. (ii) Fluorescence microscopy images of SA detection in *Arabidopsis thaliana* roots, stems, and leaves. Scale bars = 100 μm. (iii) Relative fluorescence intensity of *Arabidopsis thaliana* segments. Adapted with permission from ref. 515.

Salicylic acid (SA) quantification is important, being it a ubiquitously endogenous phenolic hormone (*i.e.*, phytohormone) found in plants, which exists in both free and bound states.^549^ It plays a key role in regulating the plant's defence mechanisms against a wide range of biotic and abiotic stresses, such as UV radiation, ozone, temperature extremes, metal toxicity, and salinity.^550^ In addition, SA is biosynthesised by plants to fight against a broad spectrum of phytopathogens, including fungi, bacteria, and viruses.^551,552^ Recently, Yang and colleagues^515^ reported an adamantane-modified rhodamine derivative (RAD, Fig. 16a) sensor using a CB7 host–guest complex (CB7⊃RAD; *K*_a,CB7_ = 3.4 × 10^4^ M^−1^) for fluorescence-based imaging of salicylic acid (SA) in plants (living *Nicotiana glutinosa* L. callus, *Arabidopsis thaliana* and tomato seedlings) and in EtOH/H_2_O mixtures (1 : 1 v/v, *λ*_ex_ = 555 nm). The detection using CB7⊃RAD relied on SA-induced spirolactam ring opening of RAD, resulting in a 330-fold fluorescence enhancement upon analyte addition (Fig. 16b), whereas CB7 prevents RAD self-aggregation through macrocyclic confinement. The chemosensor was reported to exhibit excellent selectivity and a LoD of 10 nM, which seems surprisingly good considering the reported binding affinity. Potential competing analytes *e.g.*, acetylsalicylic acid, benzoic acid, 3-hydroxybenzoic acid, *o*-methylbenzoic acid, and other related compounds, as well as to plant hormones such as cytokinin 6-BAP, abscisic acid, auxin (*e.g.*, IAA), jasmonic acid, and ethylene do not cause any interference. These results highlighted the sensor specificity for SA, enabling the real-time detection of SA-induced stomatal closure in *Arabidopsis thaliana* leaves for the first time (Fig. 16c).

**Fig. 17:**
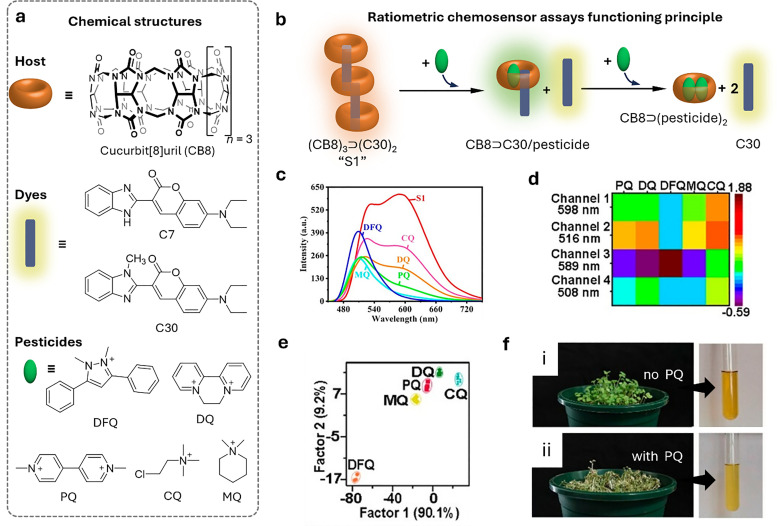
(a) Chemical structures of CB8, coumarin-based indicator dyes, and the pesticides used for detection. (b) Schematic representation of the ratiometric chemosensor assay's working principle. (c) Fluorescence emission spectra of the S1 chemosensor in the presence of pesticides in water (*λ*_ex_ = 481 nm). (d) Heat map showing the chemosensor's wavelength-dependent response to different pesticides. (e) Canonical score plot from the LDA analysis for pesticide discrimination in water. (f) Photograph of Chinese cabbage seedlings treated with deionised water and PQ (1 mM) for 5 days. Qualitative detection of Chinese cabbage seedling extract using S1. Adapted with permission from ref. 484.

Whereas, switching to ratiometric chemosensor assays, a supramolecular sensor array was recently developed by Huang and co-workers^484^ for the detection of quaternary ammonium pesticides (QAPs) in water (Fig. 17a). QAPs include two non-selective contact herbicides, PQ and diquat (DQ), the selective herbicide difenzoquat (DFQ), and two plant growth regulators, chlormequat (CQ) and mepiquat (MQ).^553^ Their detection is of significant interest since they have been associated with poisoning cases or accidental ingestion and analysed in biological fluids for occupational exposure.^554,555^ Here, authors exploited a fluorescence-based ratiometric chemosensor assay based on chemosensors formed by CB8 and coumarin dyes, either 3-(2-*N*-methylbenzimidazolyl)-7-*N*,*N*-diethylaminoc coumarin (coumarin 30, C30) or 3-(2-benzimidazolyl)-7-(diethylamino)coumarin (coumarin 7, C7). The two ratiometric sensors (CB8)_3_⊃(C30)_2_ or (CB8)_3_⊃(C7)_2_, namely S1 and S2, show different fluorescence responses and varying degrees of blue shift upon the interaction of the five QAPs with the supramolecular complex (Fig. 17b and c). These interactions cause cooperative and competitive effects, leading to multiple signal changes. Pesticides were detected at concentrations ranging from 0.2 to 1.75 μM, and the interference by anions and cations in water, such as ClO^−^, Br^−^, Cl^−^, OH^−^, SO_4_^2−^, K^+^, Na^+^, Mg^2+^, Mn^2+^, Ca^2+^ and Fe^3+^ (at 300 μM), was minimal, except for differential responses to ClO^−^, Cl^−^ and Br^−^. In spiked real river water and plant extracts treated with cationic pesticides, the chemosensor assay enabled the effective quantification of pesticides. In plant extracts, paraquat was specifically detectable, demonstrating the practical applicability of the sensor.

#### 2.2.4 Pillar[*n*]arenes-based chemosensors

PA*n* are a class of synthetic macrocycles that hold much promise in several sensing and imaging applications.^556^ The use of PA*n* for detecting pesticides and other pollutants has recently been emphasised, particularly in quantifying per- and poly-fluoroalkyl substances (PFAS), such as perfluorooctane sulfonic acid (PFOS) and perfluorooctanoic acid (PFOA), which are commonly referred to as “forever chemicals.” Indeed, these chemicals have emerged as significant environmental concerns worldwide since PFOS can lead to a variety of health issues, according to toxicity studies,^557–559^ and they are nowadays present in air, water, soil and animals.^560–567^ In 2016, the EPA established a health advisory limit of 70.0 ng L^−1^ for PFOS and PFOA, but this threshold was drastically reduced to 4.00 ng L^−1^ in 2023 in drinking water,^508^ posing a significant challenge for the detection of PFAS. In Europe, PFAS regulation is becoming stricter, with new EU limits taking effect in 2026. The revised drinking water directive sets a maximum of 0.1 μg L^−1^ for 20 individual PFAS compounds and 0.5 μg L^−1^ for total PFAS.^568^

**Fig. 18:**
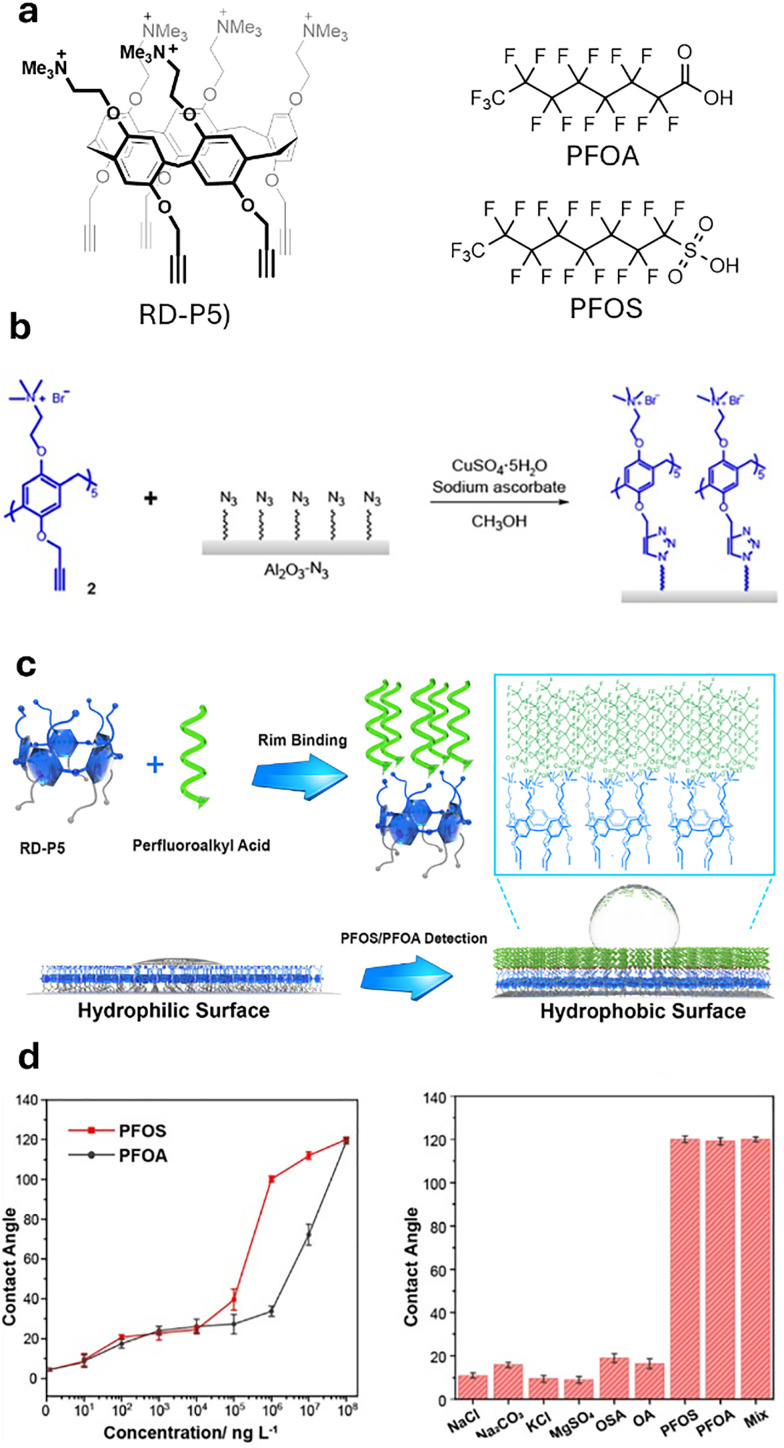
(a) Chemical structures of the ammonium and alkyne-rim differentiated pillar[5]arene (RD-P5), PFOA and PFOS. (b) Schematic representation of RD-P5 immobilisation *via* CuAAC onto an azide-functionalised Al_2_O_3_ surface. (c) Contact angle-based detection of PFOA and PFOS. (d) The plot of contact angle (CA) *versus* PFOS/PFOA concentration (left) and CA response for different compounds at 100 mg L^−1^ in mixed sample compositions (right). Figure adapted with permission from ref. 507.

To be able to reach such a detection limit, a novel supramolecular approach has been reported in 2024 by Zuilhof, Miloserdov, and co-workers (Fig. 18a).^507^ In their design, an ammonium and alkyne rim-differentiated pillar[5]arene (RD-P5) was immobilised onto an Al_2_O_3_ surface *via* a CuAAC reaction, forming a P5-Al_2_O_3_ surface (Fig. 18b). This macrocycle, previously described in another authors’ study,^569^ exhibits a binding affinity of 2.60 × 10^6^ M^−1^ for PFOS (1 : 5.6) and 5.20 × 10^4^ M^−1^ for PFOA (1 : 5.9) in phosphate buffer (20 mM at pH 5.6), with the lower affinity attributed to PFOA's higher water solubility. Thus, upon immobilisation on the Al_2_O_3_ surface, the RD-P5 complex enabled PFAS detection by monitoring changes in the surface static water contact angle. More in detail, the P5-Al_2_O_3_ surface was initially super-hydrophilic (CA < 5°), but upon immersion in PFOS solution, CA increased, indicating a change from super-hydrophilic to hydrophobic behaviour. This method achieved a LoD of 100 ng L^−1^, even as low as 10.0 ng L^−1^ in some cases (*i.e.*, 0.20 nM for PFOS – down to 0.020 nM, and 0.242 nM for PFOA – down to 0.0242 nM) and enables the distinction between perfluorinated and non-fluorinated alkane contaminations. The exceptionally high affinity arises from the distinctive architecture of RD-P5, which present five closely arranged amine groups at each rim, enabling the binding of up to five PFAS molecules. This multivalent interaction promotes the formation of a stable local fluorous microenvironment, thereby accounting for the observed high binding constant. Future work will be needed to determine whether this method can be applied to real soil samples that contain other negatively charged interferents such as fatty acids, to expand its use to more complex environments beyond water samples.

In summary, it can be concluded that the use of supramolecular chemosensors, particularly those based on the working principle of IDAs, remains relatively underexplored for in planta and on planta applications. Therefore, a major challenge could be represented by their potential deactivation by plant components, *e.g.*, proteins,^570,571^ high salt concentrations,^148^ or insufficient uptake by plant cells. For this reason, as will be discussed, future research will focus on addressing these issues through the use of nanoformulations, specifically encapsulating chemosensors in nanoparticles carriers. For example, encapsulation in polymeric and permeable nanoparticles has already been shown to prevent protein-based deactivation:^572^ this approach can also protect the cargo against interference from competing salts, impeding the diffusion through the hydrophobic bilayer membranes shielding the chemosensor. Besides, chemosensors encapsulated within lipid bilayers for assessing the permeability of such membranes can also potentially be investigated for the detection of the pesticides and metabolites discussed herein.^573^ Furthermore, nanocarrier functionalisation could facilitate targeted delivery by providing specific features to enhance their usefulness. Future research should focus on improving plant uptake, distribution and in increasing chemosensor stabiility for effective in planta and on planta applications.

### 2.3 Luminescence-based probes

As introduced in the previous chapters, traditional assays used for the detection of pesticides and plant hormones *e.g.*, GC/LC-MS,^574^ HPLC,^575^ enzyme-linked immunosorbent assay – ELISA^576^ are characterised by considerable limitations in their application, particularly regarding on-site and real-time imaging especially in the context of on-site and real-time imaging.^577^ For this reason, luminescence-based probes have begun to be widely employed for the detection of plant hormones (and their receptors), utilising a variety of luminescent materials, such as inorganic materials, nanoparticles, and genetically encoded luminescent probes. Their straightforward structural modification, convenient functional modulation and good biocompatibility are indeed coupled to rapid response, high sensitivity, and good selectivity,^114,141,578^ making luminescent assays an ideal method for on-site detection in a practical setting. Their structural modification, convenient functional modulation, and excellent biocompatibility are indeed linked to rapid response, high sensitivity, and good selectivity, making luminescent assays an ideal method for on-site detection in a practical setting.

In this subchapter, a representative ensemble of fluorescent probes utilised for various detection purposes are discussed, which are summarised in Table 5.

**Table 5:** Summary of reported probes, listed with the related excitation and emission wavelengths (*λ*_ex_/*λ*_em_) and LoDs. Reported are the analytes with the related binding affinities in brackets: (—) indicates no binding affinity given

Detection mode	Luminescent probe	Analyte (binding affinity)	*λ*_ex_/*λ*_em_	LoDs	Ref.
Aggregation induced emission	AIEgens	Abscisic acid, ABA (—)	480/617 nm	0.098 nM	475
	Cyanostilbene-based probe	Quizalofop-*p*-ethyl (3.20 × 10^6^ M^−1^)	390/535 nm	2.98 × 10^−8^ M	512
Fluorescence	Fluorescent rhodamine 6G derivatives (Rh6G-Py, Rh6G-Th, Rh6G-BT)	Salicylic acid, SA (4.69 × 10^3^ M^−1^, Rh6G-Py; 1.43 × 10^4^ M^−1^, Rh6G-Th; 8.61 × 10^3^ M^−1^, Rh6G-BT)	532/555 nm	20.0 nM (Rh6G-Py), 6.00 nM (Rh6G-Th), 4.00 nM (Rh6G-BT)	514
	Pyrrole-pyridine derivative (receptor 1)	Perfluorooctanoic acid, PFOA (1.5 × 10^6^ M^−1^)	340/505 nm	0.24 nM	579
Ratiometric fluorescence	Dual-state-emissive chalcone dye (4MC)	Fipronil, FPN (*K*_a,alb_ = 4.00 × 10^5^ M^−1^)	430/515 nm	22.0 nM	495
	*N*-Benzylox-ycarbonyl (Cy-CO_2_Bz), *N*-ethyloxycarbonyl (Cy-CO_2_Et)	NaCl (—)	740/798 nm	170 μM (Cy-CO_2_Bz), 448 μM (Cy-CO_2_Et)	518
	FRET aptamer	Glucose (—)	488/580 nm	—	497

#### 2.3.1 Small organic molecular probes

Luminescence-based probes have been extensively utilised for the detection of phytohormones and metal ions and are anticipated to facilitate the monitoring of changes occurring in plants, for example, when they experience biotic or abiotic stress.^549^

Fluorescent probes offer numerous advantages, as discussed in this review, making them ideal candidates for real-time detection and imaging in planta. They exhibit the necessary stability and brightness, alongside specific absorption and emission characteristics at designated wavelengths.^580^ Small-molecule fluorescent probes and labels are particularly popular owing to their biocompatibility and excellent spatiotemporal resolution, among other advantages.

**Fig. 19:**
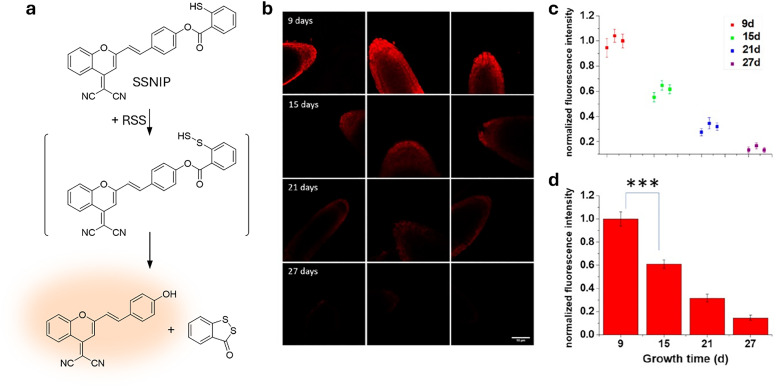
(a) Chemical structure and detection mechanism of reactive sulfur species by SSNIP. (b) Confocal microscopy images of *Arabidopsis thaliana* at different growth stages (9, 15, 21, 27 days), incubated with 50 μM SSNIP for 25 minutes, followed by replacement with fresh PBS before imaging (*λ*_ex_ = 560 nm, scale bar = 10 μm). (c) Normalisation of the confocal microscopy imaging data. (d) Normalised imaging data (each set representing three *Arabidopsis thaliana* samples for one specific growth stage). Figure adapted with permission from ref. 581.

A near-infrared fluorescent probe (SSNIP) for the selective imaging of sulfane sulfur was reported by Yuan, Liu and co-workers (Fig. 19).^581^ Sulfane sulfur represents a class of analytes belonging to the group of reactive sulphur species (RSS), which are sulphur-containing molecules playing important roles in physiological and pathological processes in plants.^582,583^ These include thiosulfoxide, present in the form of either a hydrogen polysulfide (H_2_S_*n*_, *n* ≥ 2) or per- and polysulfides (RSSH and R–S_*n*_–R, *n* ≥ 3), and inorganic sulfur derivatives (S_8_).^584^ Sulfane sulphurs represent the metabolites and/or precursors of H_2_S, which is involved in seed germination, as well as in plant growth and development at physiological concentrations. To monitor the overall levels of sulfane sulphur in living systems, the probe utilised an “off–on” strategy involving SSNIP, synthesised from 2-thiobenzoic acid and 1-(2-(4-hydroxystyryl)-4*H*-chromen-4-ylidene)malononitrile (DPCO, NIR fluorophore), which reacts with sulfane sulphur and releases its fluorogenic moiety, enabling highly sensitive detection (Fig. 19a). SSNIP provided a LoD of 4.6 nM for Na_2_S_2_ with a linear range of 0–10 μM, whereas detection can be completed within three minutes. Furthermore, in contrast to conventional methods for sulfane sulphur detection, which usually require post-mortem processing (*e.g.*, cyanolysis-based UV assay, ion chromatography, gas chromatography, or HPLC), SSNIP enabled real-time imaging of exogenous and endogenous sulfane sulphur in living plant tissues. Remarkably, its application to the roots of *Arabidopsis thaliana* showed that the levels of sulfane sulphur correlated with root growth stages (Fig. 19b), suggesting that sulfane sulphur could function as a signalling molecule promoting plant growth and root elongation.

Other important low molecular weight molecules involved in many physiological and pathological processes are intracellular thiols, such as cysteine (Cys), homocysteine (Hcy), and glutathione (GSH), which play vital roles in maintaining biological homeostasis.

**Fig. 20:**
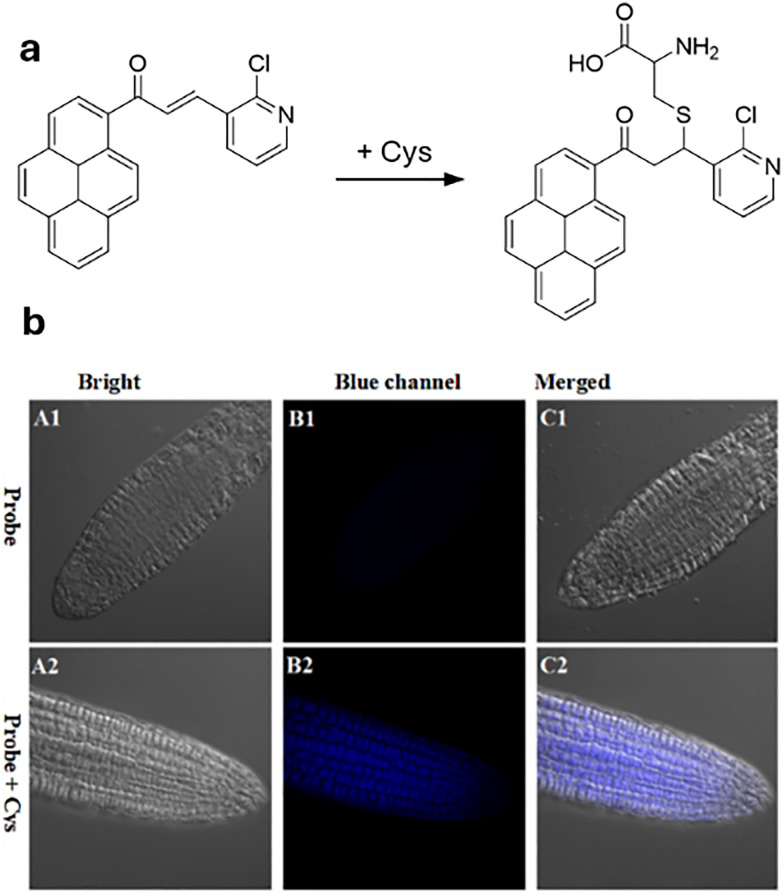
(a) Chemical structure and reaction mechanism of the thiol-selective probe (addition reaction). (b) Confocal imaging of the probe (10 μM) incubated with Cys in *Arabidopsis* root tip. (A1) *Arabidopsis* root tip incubated with the probe (10 μM) for 5 minutes; (A2) co-incubation of the probe with Cys (200 μM) for 5 minutes (blue channel: *λ*_em_ = 420–550 nm, *λ*_ex_ = 405 nm). Figure adapted with permission from ref. 488.

For this reason, fluorescence detection and imaging of Cys and Hcy in Zebrafish and *Arabidopsis thaliana* was achieved by the Yin group^488^ through a novel probe based on perylene-conjugated 2-chloropyridine (Fig. 20). The probe reacts *via* a Michael addition of Cys or Hcy to the α,β-unsaturated ketone system, disrupting conjugation, and leading to an enhanced fluorescence emission. This strategy enabled selective and sensitive thiol detection with a LoD of 2.31 μM for Cys and 4.67 μM for Hcy and a linear response range of 0–90 μM (for Cys). Furthermore, the probe was successfully applied to the root tips of *Arabidopsis thaliana*. Confocal imaging displayed minimal fluorescence after five minutes of incubation with the probe (at 10 μM); however, significant fluorescence emission was observed when incubated simultaneously with 200 μM Cys for five minutes (Fig. 20b). The rapid detection achieved, along with the probe's excellent cell membrane permeability, provided a distinct advantage over conventional thiol detection methods, such as BODIPY-based dyes^585^ or coumarin-hemicyanine fluorescent probes,^586^ which are often irreversible or require further processing after uprooting the plant. The versatility of the system in both plant and animal models thus underscored its potential for studying thiol-related physiological and pathological processes in real time.

**Fig. 21:**
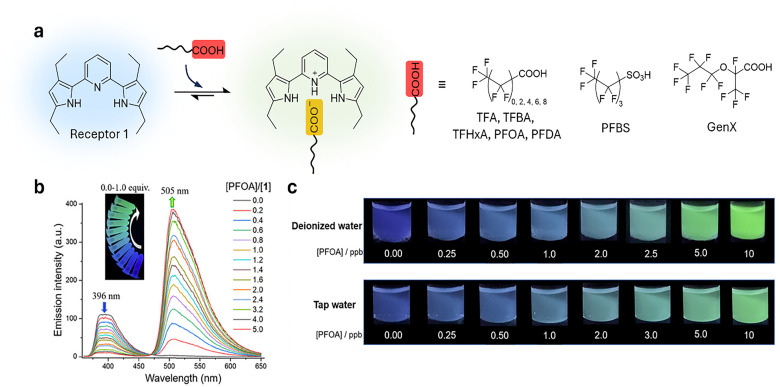
(a) Schematic representation of the binding and signal transduction mechanism of receptor 1 upon interaction with PFAS. The chemical structures of receptor 1 and a representative PFAS are also shown. (b) Luminescence response of receptor 1 (1.0 μM) upon addition of varying concentrations of PFOA (0–5.0 μM) in hexane (*λ*_ex_ = 340 nm). The inset displays photographs of the hexane solutions under UV irradiation (*λ*_ex_ = 365 nm). (c) Photographs of hexane solutions containing receptor 1 (1.0 μM) after contact with PFOA (up to 10 ppb) initially present in either deionized water or tap water. Figure adapted with permission from ref. 579.

Henkelman, Chi, Gong, Hooley, and Sessler reported the detection of perfluorooctanoic acid (PFOA) using 2,6-bis(3,5-diethyl-1*H*-pyrrol-2-yl)pyridine (receptor 1) as the probe (Fig. 21).^579^ This receptor shows good binding affinities (log *K*_a_ = 4.9–6.2) and generates a pronounced “turn-on” fluorescence response upon interaction with representative PFAS. The cleft-like structure of the probe contains both hydrogen bond donor and acceptor sites (N–H to COO^−^), enabling binding interactions with PFAS acids in the organic phase, in addition to interactions of a cation–anion nature and potential C–F⋯π interactions. Upon addition of PFOA (*K*_a_ = 1.5 × 10^6^ M^−1^) to a hexane solution of receptor 1, fluorescence titrations show a decrease in emission at 400 nm and a simultaneous increase in a broad emission cantered at 505 nm. These changes result in a distinct shift in emission colour from weak blue to intense yellow-green, with a fivefold increase in relative quantum yield. These spectral changes are attributed to protonation of receptor 1 by PFOA, forming the cation–anion complex H1^+^˙PFOA^−^. Protonation alters the HOMO–LUMO energy levels of receptor 1, accounting for the observed shifts in UV-vis and fluorescence spectra. In addition to the hydrogen bonds mentioned above, electrostatic interactions within the ion pair also play a role in the interaction of PFAS with receptor 1. Spectral titrations were also performed with other fluorinated species (Fig. 21a), including trifluoroacetic acid (TFA), perfluorobutanoic acid (PFBA), perfluorohexanoic acid (PFHxA), perfluorodecanoic acid (PFDA), GenX (2,3,3,3-tetrafluoro-2-(heptafluoropropoxy)propanoic acid), and perfluorobutanesulfonic acid (PFBS). PFAS with long fluorinated alkyl chains (≥C4) showed higher binding affinities (log *K*_a_ = 5.9–6.2), while TFA exhibited the weakest binding (*K*_a_ = 7.9 × 10^4^ M^−1^). Affinity increases with chain length (TFA < PFBA < PFHxA < PFOA, PFDA). Terminal functional groups also influence binding: PFBS (sulfonate) binds more strongly than PFOA (carboxylate), likely due to enhanced electrostatics. Among carboxylates, GenX exhibits the highest affinity, presumably due to additional ether-mediated interactions. The LoD for PFOA was as low as 250 ppt (0.60 nM) in both deionized and tap water by naked-eye observation (Fig. 21b and c). This could be further reduced to 40 ppt (0.09 nM; deionized water) and 100 ppt (0.24 nM; tap water) using a smartphone colour-scanning app to analyse the emissive hexane layer. Interference from shorter-chain PFAS, inorganic ions, or common organic contaminants was minimal. These findings suggest that receptor 1 may serve as a practical supramolecular sensor for field-based PFAS detection in the absence of conventional instrumentation.

**Fig. 22:**
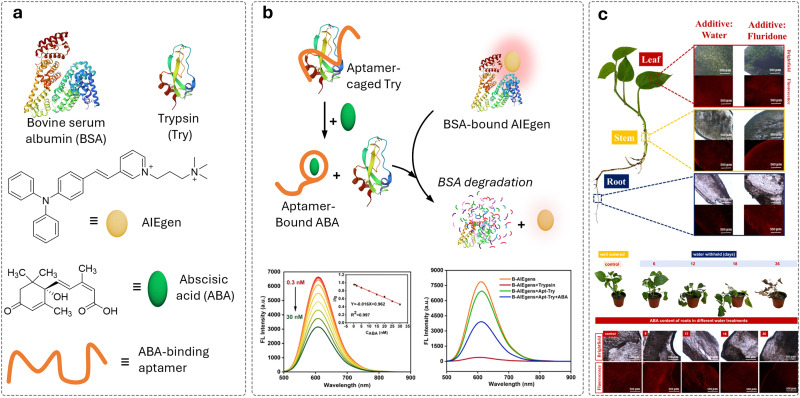
(a) X-ray crystal structures of BSA and Try, adapted from the RCSB Protein Data Bank. (b) Schematic diagram of the preparation and detection mechanism of the BAAT probe, along with the fluorescence spectra. (c) Spatial (top) and temporal (bottom) monitoring of ABA concentrations in plant tissues using the BAAT probe, including the detection of endogenous ABA content in plant roots *via* fluorescence imaging. Figure adapted with permission from ref. 475.

Chen and co-workers developed a fluorescent probe for the detection and imaging of the hormone abscisic acid (ABA) in stressed living cells through the use of a biocompatible hybrid supramolecular fluorescent probe (BAAT, Fig. 22a).^475^ Bovine serum albumin (BSA) serves as a protein host for an aggregation-induced emitting fluorophore (AIEgen), which becomes emissive only upon encapsulation in the hydrophobic BSA cavity, due to conformational rigidification of its structure. The detection system also includes an ABA-selective aptamer that, in absence of the hormone, interacts with the surface of trypsin (Try), blocking its hydrolytic activity. In contrast, in the presence of ABA, Try is displaced and released in solution, hydrolysing the α-helical structure of BSA and allowing the AIEgens to be released into the physiological environment, ultimately causing its quenching (Fig. 22b). In an aqueous solution, this chemosensor exhibits a LoD of 0.098 nM, whereas typical biological concentrations of ABA are found within the range of 0.3–30 nM. It is crucial to highlight that the primary advantage of this approach lies in the use of small AIEgen molecules. This effectively prevents the typical problem of probe aggregation and inactivation encountered in the complex biological environments of plants, enabling effective incubation within the plant tissues. This fact facilitated the colorimetric detection of ABA content by the naked eye, offering high biocompatibility, a small probe size, and spatiotemporal detection of both endogenous and exogenous ABA in plants. Importantly, other compounds, such as brassinolide, isopentenyl adenine, indole acetic acid, cytokinin, gibberellic acid zeatin, ethylene, jasmonic acid, salicylic acid, tryptophan, leucine, methionine, glutathione, cysteine, F^−^, Cl^−^, Br^−^, I^−^, NO^−^, HSO^−^, SO_3_^2−^, PO_4_^2−^, K^+^, Na^+^, Ag^+^, Fe^2+^, Hg^2+^, Cu^2+^, Co^2+^, Mg^2+^, Cd^2+^, Ni^2+^, Zn^2+^, Ba^2+^, Pb^2+^, Mn^2+^, Ca^2+^, Fe^3+^, Ce^3+^, Al^3+^, do not interfere with ABA detection. Furthermore, the ability to detect ABA in living plants was tested on *Epipremnum aureum* seedlings, focusing on ABA content at different sites (leaves, stems and roots). Specifically, endogenous ABA levels in plant roots were detected using fluorescence imaging under various water treatments (Fig. 22c).

**Fig. 23:**
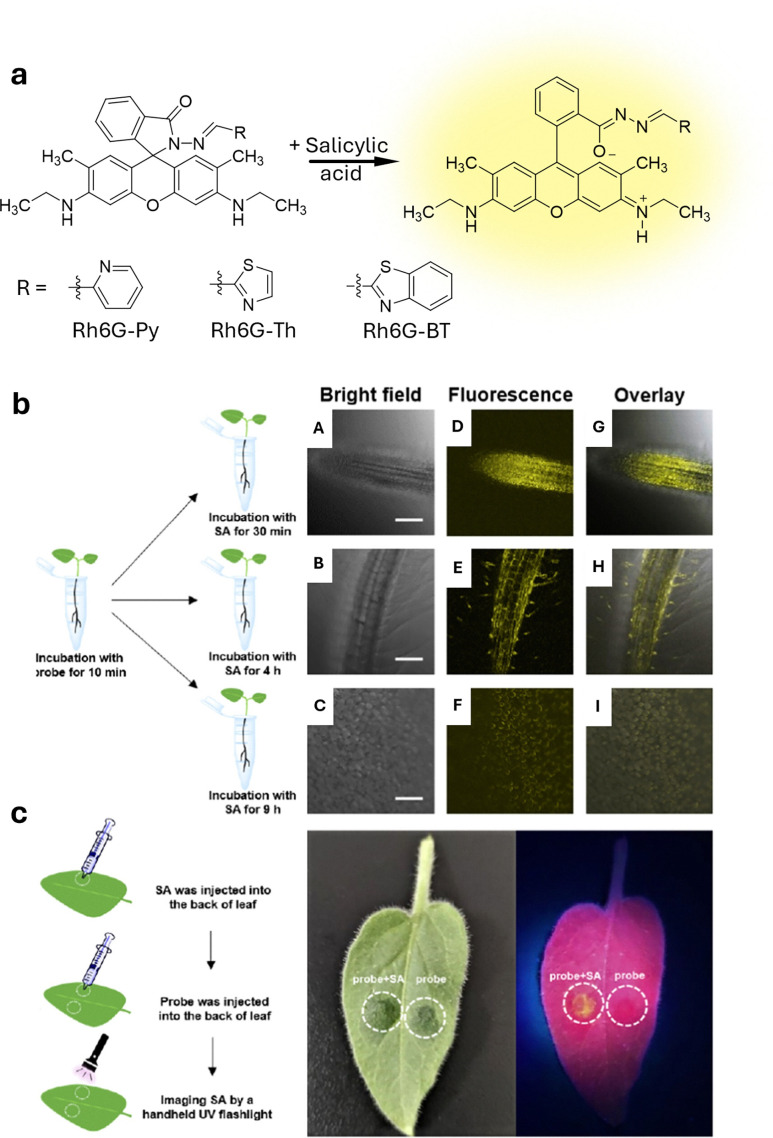
(a) Chemical structure of Rh6G-based probes and SA-mediated conversion of the spirolactam structure from a ring-closed to a ring-opened form, resulting in a significant enhancement of fluorescence. (b) Schematic illustration of SA imaging in different plant parts. (b) Two-photon fluorescence imaging of SA in *B. chinensis* L. plants were first incubated with the probe (10 μM) for 10 minutes, followed by incubation with water containing SA (125 μM) for various times to image different parts, *i.e.*, (A)–(C) root tip, (D)–(F) rootstock, and (G)–(I) leaf. (c) Schematic illustration of the leaf staining experiment and photos of the leaf: the left image shows the leaf under natural light, and the right image shows it under ultraviolet light (probe Rh6G-Py: 100 μM; SA: 1 mM). Figure adapted with permission from ref. 514.

As mentioned, the phytohormone SA regulates plant resistance to stressors and has become an important biomarker in plant sciences. However, its use has been prohibited in some countries, as it could pose a serious risk for sensitised individuals and cause various adversities, including urticaria and angioedema.^587^ Yang and co-workers previously reported fluorescent assays for the detection of SA in mammalian cells,^588^ using a rhodamine-based fluorescent probe (Fig. 23). The binding of SA induced the conversion of the probe's spirolactam structure from a closed-ring to an open-ring form, accompanied by a strong enhancement in fluorescence. More recently, the same group developed three rhodamine 6G (Rh6G)-based fluorogenic probes for the detection of SA in plants (Fig. 23a),^514^ that have high selectivity, fast response times (<60 s), and nanomolar detection limits for SA in MeOH/H_2_O (9 : 1 v/v). This is due to the fact that heterocyclic rings demonstrate improved response speed and fluorescence stability. The probes reported, namely Rh6G-Py, Rh6G-Th, and Rh6G-BT, selectively interact with SA through the formation of hydrogen bonds, which induces the previously mentioned spirolactam ring-opening, resulting in a fluorescence turn-on response. The obtained LoDs were 20 nM (Rh6G-Py), 6 nM (Rh6G-Th), and 4 nM (Rh6G-BT), with linearity ranges of 0.8–65 μM (Rh6G-Py), 0.2–13 μM (Rh6G-Th), and 10–55 μM (Rh6G-BT), respectively. Impressively, SA imaging was achieved in *Brassica chinensis* L. seedlings cultured with Rh6G-Py, followed by treatment with SA solution. SA visualisation was obtained using an FVMPE-RS two-photon confocal fluorescence microscope (Fig. 23b). Additionally, injections of SA in the presence of the probe were successfully applied to the plant leaf and fruit epidermis, allowing to determine its presence even with simple visual inspection.

#### 2.3.2 Supramolecular probes based on aggregation phenomena

J- and H-aggregates are supramolecular assembled structures of dye-molecules,^589–591^ such as cyanine dyes, *via* π–π and/or electrostatic interactions.^592^ These self-assembled quasi-one-dimensional nanostructures of π-conjugated molecules are characterised by special optoelectronic properties, including sharp exciton transitions,^593^ strong circular dichroism,^594^ high exciton mobilities,^595^ and photoconductivity.^596^

**Fig. 24:**
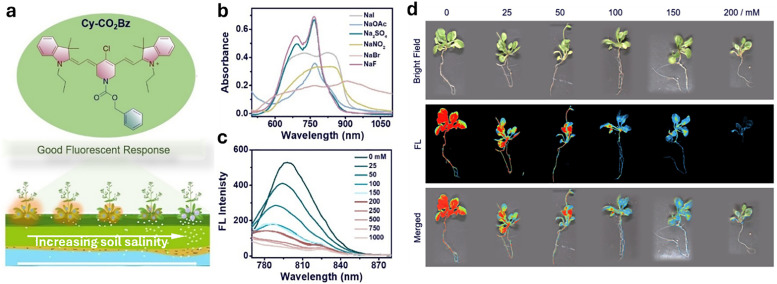
(a) Chemical structure of Cy-CO_2_Bz. This probe enables *in vivo* tracking of NaCl in plants through its fluorescence response to elevated salt levels. (b) Absorption spectra of Cy-CO_2_Bz in PBS (150 mM, pH 7.4, *c*_Cy-CO_2_Bz_ = 10 μM, 1% DMSO as cosolvent) with various salts (*c*_Cy-CO_2_Bz_ = *c*_salts_ = 200 mM). (c) Fluorescence spectra of Cy-CO_2_Bz (K) and Cy-CO_2_Et in water with different NaCl concentrations (*λ*_ex_ = 740 nm; *c*(Cy-CO_2_Bz) = 10 μM, 1% DMSO as cosolvent). (d) *In vivo* images of plants treated with different NaCl concentrations in deionised water and incubated with Cy-CO_2_Bz for 5 hours. Figure adapted with permission from ref. 518.

Salt-induced stress, such as high NaCl concentrations during dry periods, hinders plant growth, highlighting the importance of NaCl monitoring. Utilising aza-containing heptamethine cyanines dye derivatives, Yin, Yang and co-workers^518^ reported a probe for NaCl-induced salt stress in plants. The two carbamate-containing derivatives, *N*-benzylox-ycarbonyl (Cy-CO_2_Bz, Fig. 24a) and *N*-ethyloxycarbonyl (Cy-CO_2_Et) were synthesised and served as supramolecular probes. In the presence of Na^+^ ions, the Cy-CO_2_Bz compound forms J-aggregates, displaying a pronounced red-shifted, broad absorption band and a blue-shifted emission band with decreased fluorescence intensity, making it useful for ratiometric detection of salt concentrations in plants. The LoD for NaCl in water was reported as 170 μM. Remarkably, by incubating *Arabidopsis thaliana* with Cy-CO_2_Bz and exposing it to increasing NaCl concentrations, it was possible to monitor the presence of NaCl in living plants, as depicted in Fig. 24d, with negligible interference from plant metabolites reported.

**Fig. 25:**
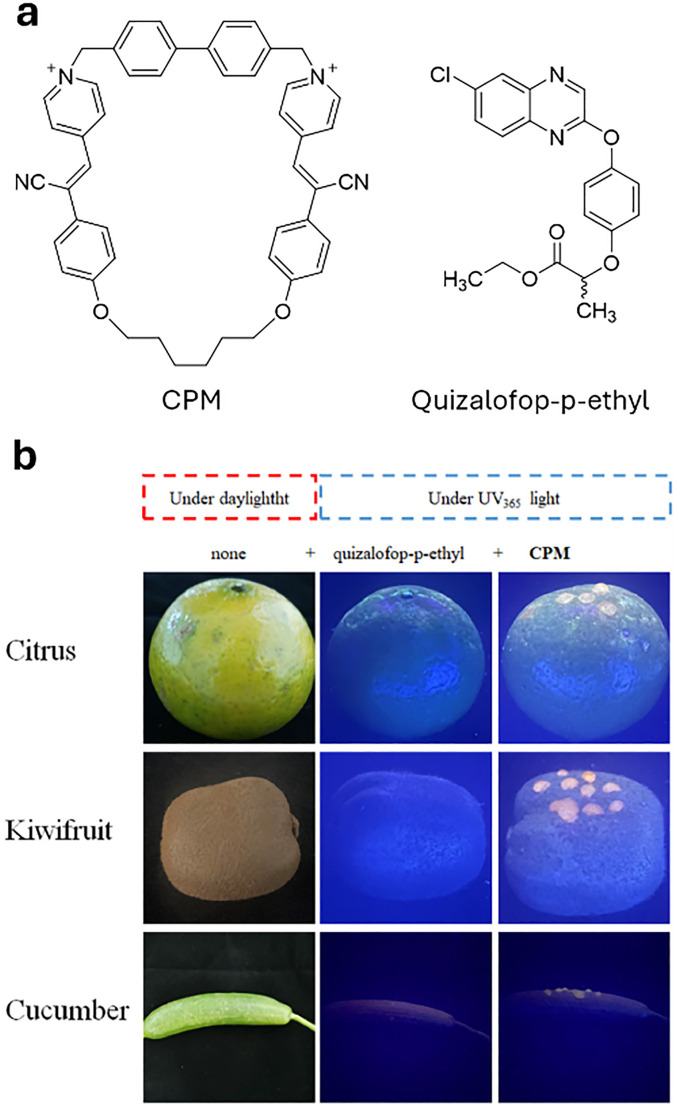
(a) Chemical structures of the chemosensor CPM and the pesticide quizalofop-*p*-ethyl. (b) Photographs of food samples (citrus, kiwifruit, and cucumber) for quizalofop-*p*-ethyl detection under UV light (365 nm). The samples were sprayed with a solution of quizalofop-*p*-ethyl and a solution of CPM successively. Figure adapted with permission from ref. 512.

Lin, Guo, Yang and co-workers^512^ reported a cyanostilbene-pyridine macrocycle (CPM)-based probe (Fig. 25) for the fluorescence-based detection of the herbicide quizalofop-*p*-ethyl (*K*_a,CPM_ = 3.20 × 10^6^ M^−1^) both in DMSO/H_2_O (5 : 95) mixtures and on the surface of fruits (kiwi, citrus) and vegetables (cucumber). The solvent composition has been selected based on the fact that increasing the DMSO content (a poor solvent for CPM) strengthens aggregation and enhances the AIE effect. Simultaneously, fluorescence is increased as J-aggregates are formed. In the presence of the pesticide, their red fluorescence is then shifted back to the blue wavelength region. Other pesticides, including glufosinate-ammonium, *N*-(phosphonomethyl)glycine 2-propylamine, carbendazim, hymexazol, clopyralid, fluroxypyr, thiophanate-methyl, hexazinone, sulfometuron-methyl, niclosamide ethanolamine salt, metaldehyde, 1-naphthaleneacetic acid, bromoxynil octanoate, thiamethoxam, tricyclazole, monosultap, isultap, and cartap, did not elicit a response from the probe. Additionally, the probe's selectivity was confirmed by testing against ions such as Na^+^, K^+^, Mg^2+^, Ca^2+^, HCO_3_^−^, CO_3_^2−^, PO_4_^3−^, and NO_3_^−^, none of which caused interference. A LoD of 29.8 nM and a LoQ of 99.4 nM were reported in aqueous solutions.

**Fig. 26:**
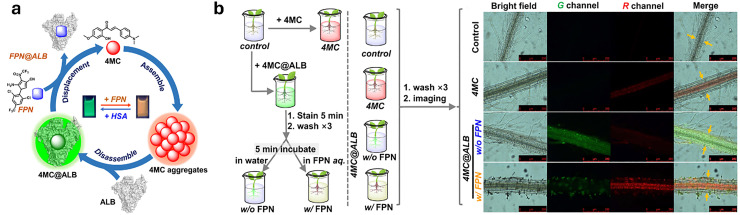
(a) Design and mechanism of the 4MC-ALB complex for ratiometric detection of FPN. (b) Staining and treatment procedure for *in situ* tracking of FPN. Fluorescence imaging of *Arabidopsis thaliana* root segments: the first row shows the control group incubated in nutrient solution for 5 min. The second row shows incubation in 4MC-spiked solution for 5 min. The third row shows incubation in 4MC@ALB-spiked solution for 5 min, followed by nutrient solution for another 5 min. The fourth row shows incubation in 4MC@ALB-spiked solution for 5 min, then transferred to FPN-spiked solution for another 5 min. Scale bar: 250 μm. Figure adapted with permission from ref. 495.

Fipronil (FPN) is a widely used phenylpyrazole pesticide used for agricultural pests control, as it can block chloride channels associated with γ-aminobutyric acid (GABA) receptors.^597^ However, FPN exposure can cause a series of acute neurological disorders, as well as several chronic damages in the liver and kidney. For this reason, FPN usage is largely prohibited in China, the European Union, and United States.^598^ A probe for the detection of FPN was reported by Liu, Xu, Zhao and co-workers^495^ by making use of a fluorescent probe based on a dual-state-emissive chalcone dye (4MC), which shows a pronounced green fluorescence (*λ*_em_ = 515 nm) when bound to the inner cavity of albumin (ALB, Fig. 26). Ratiometric detection of FPN was feasible by its competitive binding to ALB, which displaces 4MC (Fig. 26a). Once released in solution, 4MC self-assembled into red-emissive aggregates, causing a red-shift of more than 60 nm in the emission spectra. This method achieved a LoD of 22 nM (∼0.01 ppm) in PBS buffer (1 mM, pH 7.4), which is much lower than the toxicity threshold for humans (reference dose (RfD) = 0.5 ppm) set by EPA. The presented assay can be completed within three minutes and displays good selectivity against other pesticides, *e.g.*, chlorantraniliprole, thiamethoxam, carbaryl, diafenthiuron, permethrin, chlorpyrifos, and indoxacarb. Other inorganic ions (K^+^, Na^+^, Mg^2+^, Ca^2+^, NH_4_^+^, SO_4_^2−^, NO_3_^−^, Cl^−^, PO_4_^3−^, HPO_4_^2−^, H_2_PO_4_^−^) did not interfere with the fluorescence response (*c*_pesticides_ = 0.5 mM, *c*_ions_ = 1 mM). Furthermore, the probe was successfully applied to detect FPN in the root segments of *Arabidopsis thaliana* seedlings by incubating them with 4MC@ALB, followed by treatment with the pesticide (Fig. 26b).

#### 2.3.3 DNA aptamer-based biosensors

Nucleic acid-based aptamers, *i.e.*, RNA and DNA aptamers, are a class of synthetic single-stranded oligonucleotides capable of selectively binding non-nucleic acid targets with high affinity and specificity. Over the past few decades, they have been extensively studied, primarily through the systematic evolution of ligands by exponential enrichment (SELEX procedure) and similar selection methodologies.^599–601^ DNA aptamers, in particular, have emerged as a major class of biosensors, showing widespread applications for their employment as probes in the detection of proteins^602–604^ toxins,^605,606^ small organic molecules,^607,608^ and metal ions.^609^ When used for detection purposes, aptamers are typically functionalized with luminescent functional groups as reporter molecules; in some cases, however, fluorescence quenchers can also be introduced. When binding to an analyte, aptamers undergo conformational changes, a property that can be exploited by incorporating organic fluorophores into regions of the aptamer that are sensitive to structural modulation.^464,610^ This strategy enables the conversion of ligand binding events into changes in the local chemical environment of the fluorophore, thereby altering its fluorescence properties, such as excimer formation or increased fluorescence intensity due to structural stiffening and influencing measurable parameters such as intensity, emission wavelength maximum and anisotropy. When two reporter molecules are introduced, signal transmission can be mediated by FRET. Alternatively, if one reporter acts as a luminescence quencher, the binding event can be detected by monitoring changes in luminescence intensity, such as “turn-on” or “turn-off” effects.

**Fig. 27:**
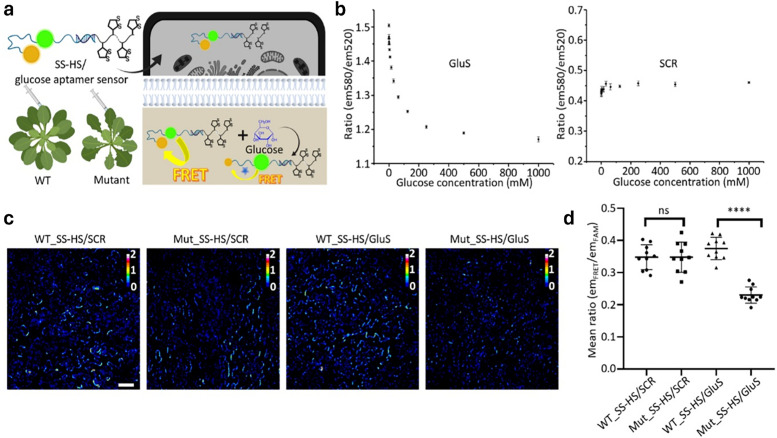
(a) Glucose sensing with glucose aptamer sensor delivered *via* thiol-mediated uptake in WT *Arabidopsis* and *Arabidopsis* atsweet[11;12] double mutants. Schematic illustration of the infiltration, uptake of SS-HS/glucose aptamer sensor, and the glucose aptamer sensor's FRET ratio change after conformation rearrangement upon binding to glucose in WT *Arabidopsis* and *Arabidopsis* atsweet[11;12] double mutants. (b) The FRET responses between donor, FAM, and acceptor (TAMRA) were monitored concerning increasing glucose concentrations for glucose aptamers and scrambled control. (c) The FRET ratio images of WT *Arabidopsis* leaf cells and atsweet[11;12] mutant leaf cells infiltrated by SS-HS/SCR and SS-HS/GluS. Scale bar, 50 μm. (d) Quantification of the FRET ratio images of WT *Arabidopsis* leaf cells and atsweet[11;12] mutant leaf cells infiltrated by SS-HS/SCR and SS-HS/GluS. Figure adapted with permission from ref. 497.

A DNA aptamer-based biosensor was developed by Chen, Lu and co-workers^497^ for the ratiometric FRET detection of glucose in *Arabidopsis* and tobacco leaf cells (Fig. 27a). The aptamer, first reported in 2018,^607^ shows high selectivity for glucose over other sugars, such as galactose and fructose. The aptamer has the sequence 5′-CGACCGTGTGTGTA/i6-FAMK/TTC TAT ACA GTG TCC ATT GTC G/36-TAMTSp/-3′, where i6-FAMK denotes a fluorescein modification and 36-TAMTSp a tetramethylrhodamine (TAMRA) dye. These dyes form a luminescent FRET pair for glucose detection. Upon glucose binding, *via* non-covalent interactions with six nucleotides in the aptamer's bulge region,^611^ the aptamer undergoes a conformational change that increases the distance between the dyes, leading to reduced FRET efficiency, observed as a decreased emission ratio *I*_em_(580 nm)/*I*_em_(520 nm) (TAMRA/fluorescein, Fig. 27b). To enhance cellular uptake, the aptamer was hybridized with a disulfide-modified helper strand, 5′-ACACGGT CGTT/iSp18//SS/15-3′ (SS-HS), which includes an 18-atom hexaethylene glycol spacer (/iSp18/) and 15 disulfide units (/SS/15). This modification enables thiol-mediated uptake, previously described in mammalian cells, involving dynamic covalent disulfide exchange with thiol-containing transporters on the cell surface.^612,613^ This oligonucleotide is under 20 nm in size, meeting the plant cell wall exclusion limit, and its disulfide units have been shown to enhance nucleic acid delivery into plant cells. The resulting disulfide-linked aptamer complex (SS-HS/GluS) was infiltrated into leaves of wild-type *Arabidopsis thaliana* and atsweet11;12 double mutants, which accumulate higher glucose levels. Harvested leaves were imaged to assess FRET signal ratios, confirming glucose detection by SS-HS/GluS, which showed decreased FRET efficiency in wild-type plants (Fig. 27c and d). A scrambled sequence control (SS-HS/SCR) was used to validate specificity. This study serves as a proof-of-concept for using aptamers to detect plant metabolites and highlighting the potential of DNA aptamer sensors for functional studies of diverse plant targets, including metabolites, hormones, metal ions, and proteins.

### 2.4 Organic- and metallorganic-based probes

Currently, numerous organic and metal–organic fluorescent probes have been developed, including luminescent probes for the detection of thiols and biothiols, utilising various mechanisms such as bond cleavage reactions, conjugate additions, or nucleophilic substitutions.^614^ It has been well established that fluorogenic methods, when paired with suitable probes, provide an excellent sensing option in plant sciences. This is particularly due to their high selectivity and sensitivity, low detection limits, ease of use, and considerable potential for application in live cell imaging with fluorescent probes.^615^

Furthermore, fluorogenic methods are non-destructive and can afford real information on the localisation and quantity of the targets of interest. Generally, fluorescent probes may contain various groups as binding sites, such as Schiff bases, ureas, pyridine, pyrenes, anthracenes, quinolines, and naphthalene, coumarins, and rhodamines.^141^ A summary of the reviewed fluorescent probes is presented in Table 6.

**Table 6:** Summary of probes for pesticides, metabolites and metal ions detection, listed with detection medium, excitation and emission wavelengths (*λ*_ex_/*λ*_em_) and reported LoDs. Reported are the analytes with the related binding affinities in brackets: (—) indicates no binding affinity given

Fluorescent probe	Medium	Analyte (binding affinity)	*λ*_ex_/*λ*_em_	LoDs	Ref.
Salamo-salen-salamo hybrid Mg^2+^ complex (MT)	DMSO/H_2_O (9 : 1, v/v)	H_2_PO_4_^−^ ions (2.6 × 10^4^ M^−1^)	389/461 nm (emission red-shifted to 470 nm upon H_2_PO_4_^−^ addition)	3.3 × 10^−8^ M	490
DACH-fhba or 1,2-cyclohexanediamine + 3-(*tert*-butyl)-5-formyl-4-hydroxybenzoic acid	DMSO 5% in H_2_O	Zn^2+^ (6.05 μM, 1 : 1 coordination ratio) and OH^−^	405/455 nm (Zn^2+^ addition), 405/530 nm (OH^−^ addition)	56 nM (Zn^2+^); response for pH 7–9.4 (p*K*_a_ = 8.4)	524
Schiff base-based fluorescent turn-on sensor (probe L)	DMSO/H_2_O (1 : 9, v/v)	Al^3+^ (3 × 10^7^ M^−1^)	370/472 nm	1 × 10^−5^ M	477
SSNIP (2-thiobenzoic acid) + 1-(2-(4-hydroxystyryl)-4*H*-chromen-4-ylidene)malononitrile	DMSO 1% in H_2_O	Sulfane sulfur or reactive sulphur species, RSS (—)	560/680 nm	4.6 nM (Na_2_S_2_)	581
Fluorescent probe (1-acetyl pyrene + 2-chloropyridine-3-carbaldehyde)	H_2_O/CH_3_CN (3 : 1, v/v)	Cysteine, Cys (—); homocysteine, Hcy (—)	370/464 nm	2.31 μM (Cys); 4.67 μM (Hcy)	488
Artificial metalloenzyme (ArM)	H_2_O	Ethylene gas (—)	420/463 nm	34.4 μL (∼27 ppm in air) *in vitro*	494

#### 2.4.1 Schiff base-based probes

Among several fluorescent probes, Schiff base-based chemosensors are particularly important due to their straightforward synthesis, which involves a condensation reaction between aldehydes and amines.^616,617^ Particularly, Schiff base-based chemosensors offer an ideal electronic and geometrical environment for coordinating with single metal ions or multiple metal ions simultaneously; thus, they are currently widely employed in the design of metal ions probes.

In more in detail, Schiff bases typically consist of hydrazones, acyl hydrazones, salicylimines, and azines, among others, providing nitrogen and oxygen atoms for coordination with various metal ions. In addition to their strong chelating ability to metal ions, they also possess low toxicity along with antibacterial and antiviral activities, which makes them particularly appealing for biomedical applications. The Schiff bases themselves exhibit weak fluorescence;^618^ however, this increases significantly after cation chelation. Salamo-based analogues, first introduced by Nabeshima and co-workers,^619–621^ have also been extensively used as probes, that feature improved stability in aqueous environments.

**Fig. 28:**
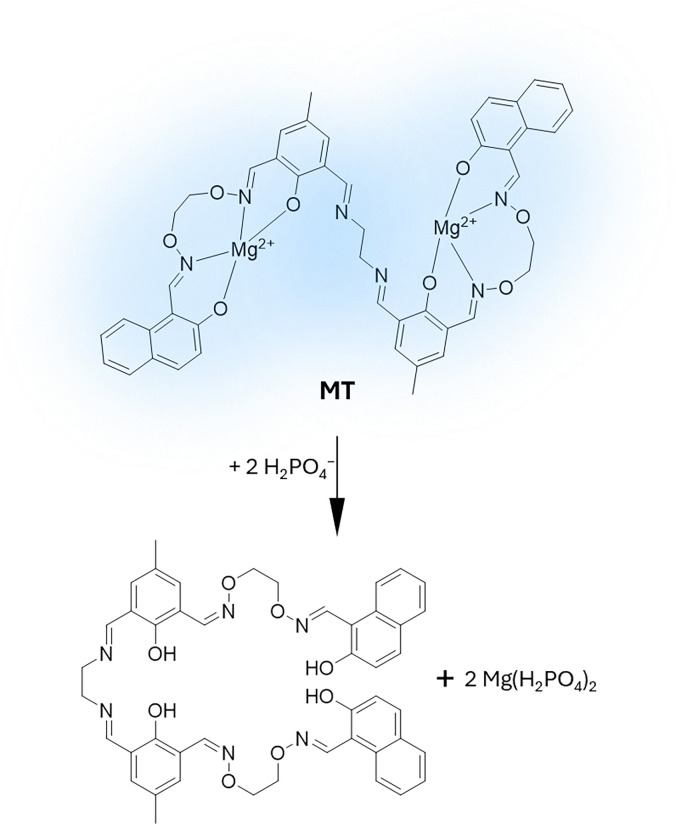
Salamo-salen-salamo hybrid Mg^2+^ complex for the fluorescence detection of H_2_PO_4_^−^ ions.

Recently, Sun, Dong and their colleagues^490^ reported a novel salamo-salen-salamo hybrid Mg^II^ complex fluorescent chemosensor (MT, Fig. 28) for detecting H_2_PO_4_^−^ (used as fertiliser) in Zebrafish and plants. Briefly, pre-complexation of the probe with the Mg^2+^ cation yielded a bright and blue-emitting complex. However, in the presence of H_2_PO_4_^−^, which binds more strongly to Mg^2+^ and displaces it from the probe, the emission intensity decreases, accompanied by a slight red shift, enabling anion detection in a DMSO/H_2_O (9 : 1, v/v) solvent mixture. As previously mentioned, the underlying mechanisms causing the fluorescence changes can be explained by intramolecular charge transfer (ICT) and the CHEF effect. Other anions, such as PO_4_^3−^, HPO_4_^2−^, P_2_O_7_^4−^, F^−^, Cl^−^, Br^−^, I^−^, C_2_O_4_^2−^, CO_3_^2−^, HCO_3_^2−^, SO_4_^2−^, HSO_4_^−^, SO_3_^2−^, HSO_3_^−^, NO_3_^−^, NO_2_^−^, S_2_^−^, S_2_O_8_^2−^, SCN^−^, CN^−^, OAc^−^, B_4_O_7_^2−^ and CrO_4_^2−^, caused no significant interference when detected in the presence of H_2_PO_4_^−^ in DMSO/H_2_O (9 : 1, v/v). Furthermore, the visualisation of H_2_PO_4_^−^ in soybean sprouts showed that the probe has the potential for H_2_PO_4_^−^-detection in plants.

**Fig. 29:**
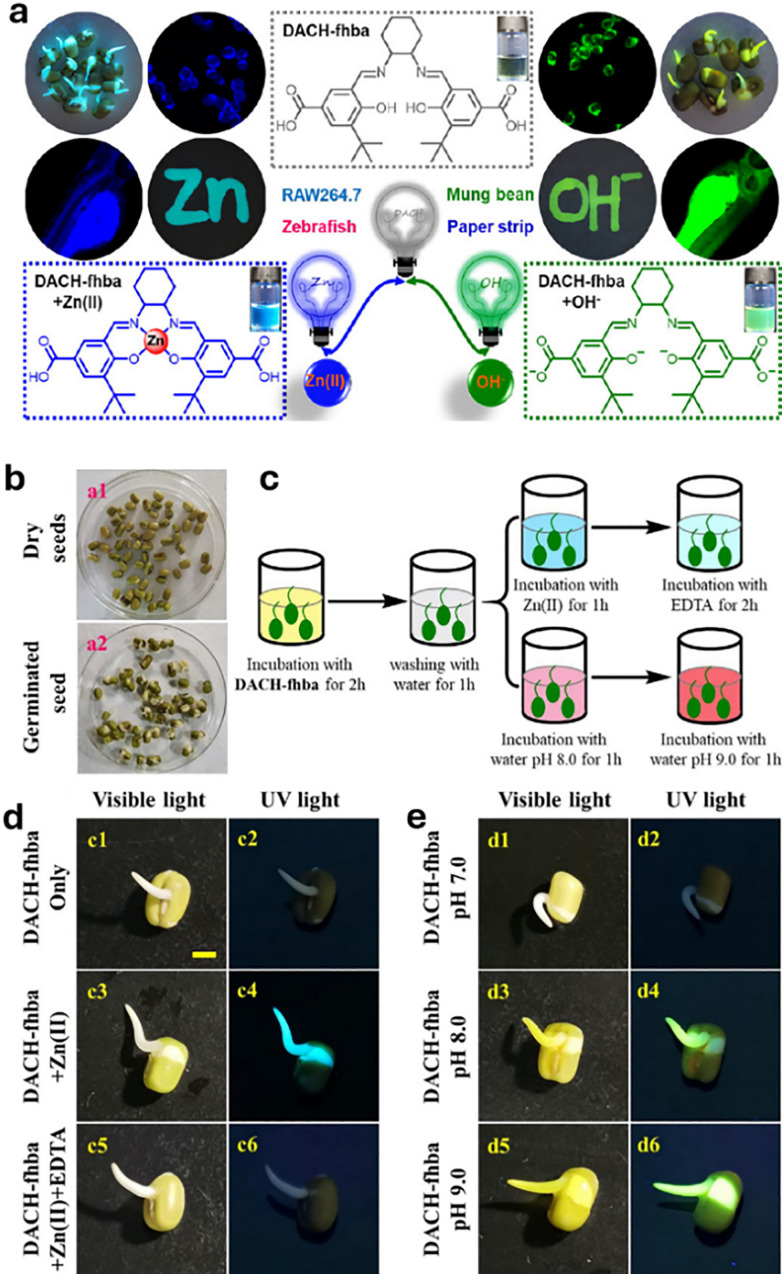
(a) Fluorescence-based detection mechanism of Zn(ii) and pH using the DACH-fhba sensor through a emission turn-on strategy. (b) Growth of mung bean sprouts. (c) Schematic diagram of the experimental design for fluorescence imaging in plants. (d) Fluorescence images of sprouts in a solution of DACH-fhba (10 μM) with Zn^2+^/EDTA. (e) Fluorescence images of sprouts after the addition of DACH-fhba (10 μM) followed by different pH buffer solutions. Scale bar = 2500 μm. Figure adapted with permission from ref. 524.

In excessive amounts, Zn^2+^ is a serious toxic pollutant.^622^ Chen, Shen and co-workers^524^ developed a dual-functional fluorescent probe (DACH-fhba, Fig. 29a) for the selective detection of Zn^2+^ ions and OH^−^ in mung bean sprouts. The sensor was synthesised by condensing 1,2-cyclohexanediamine with 3-(*tert*-butyl)-5-formyl-4-hydroxybenzoic acid. DACH-fhba functions as a probe with a two-channel fluorescence signalling turn-on strategy that allows its use for the bioimaging and mapping of Zn^2+^ in living cells and Zebrafish. Furthermore, it facilitated the visualisation of these analytes on paper strips and in mung bean sprouts. DACH-fhba exhibited high sensitivity with a reported detection limit of 56 nM for Zn^2+^ and a reactive pH range of 7 to 9.4 with a p*K*_a_ of 8.4. Notably, DACH-fhba exhibited significant changes in absorption and fluorescence emission depending on the pH, making it useful for detecting pH fluctuations or Zn^2+^ concentrations in various samples. However, pH variations may complicate the detection of Zn^2+^; this factor should be evaluated when using this chemosensor.

**Fig. 30:**
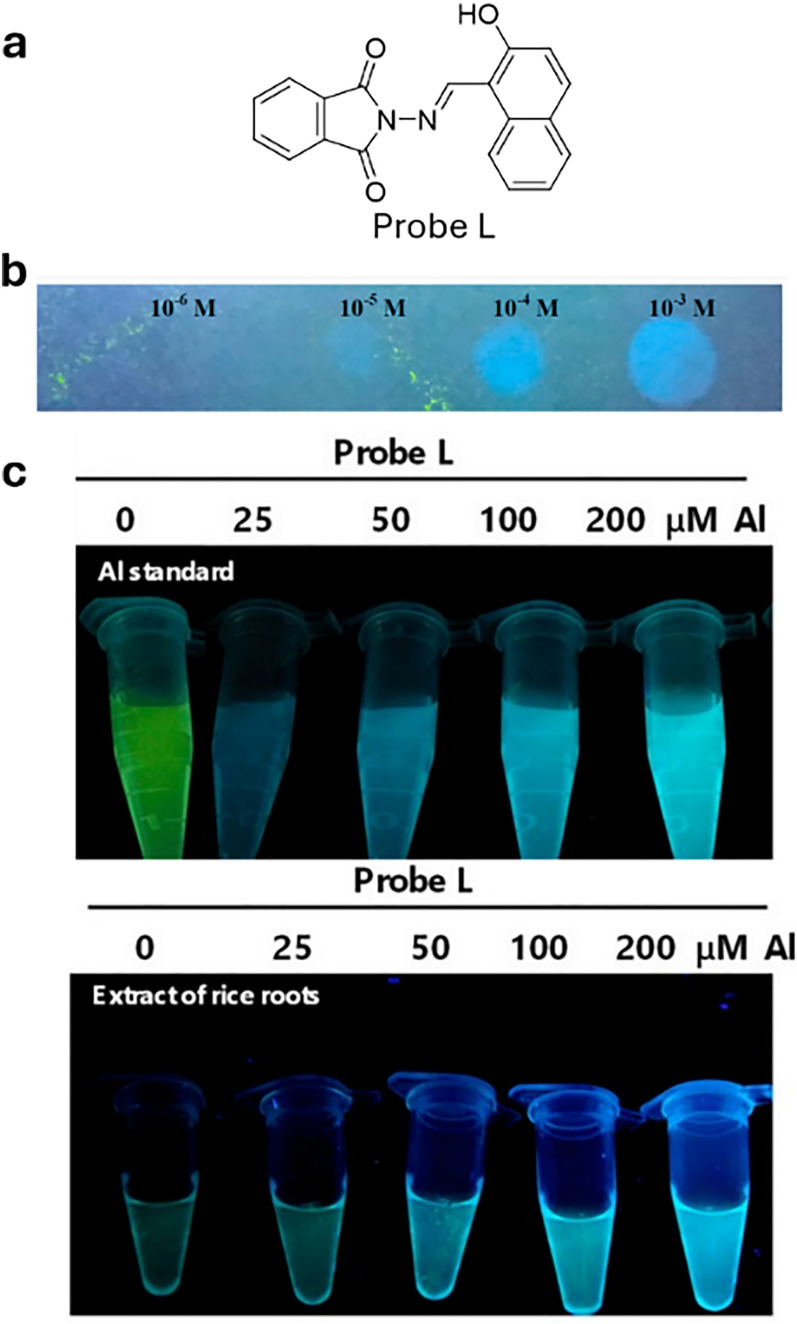
(a) Chemical structure of probe L. (b) Colour changes of probe L observed under UV light upon the addition of Al^3+^ at different concentrations on filter paper. (c) Top image: Fluorescence of probe L after the addition of various concentrations of Al(ClO_4_)_3_ solution (0, 25, 50, 100, and 200 μM), excited by a handheld UV lamp at 345 nm. The blue emission was photographed immediately in the dark. Bottom image: Fluorescence of probe L after the addition of rice extracts treated with various concentrations of Al^3+^. Figure adapted with permission from ref. 477.

Moreover, a new Schiff-based fluorescent turn-on sensor (probe L, Fig. 30a) was developed for the selective detection of Al(iii) ions by coupling 2-hydroxy-1-naphthaldehyde with 2-aminoisoindoline-1,3-dione.^477^ Probe L exhibited good selectivity and sensitivity towards Al^3+^ ions (*K*_a_ = 3.00 × 10^7^ M^−1^, based on Hill plot analysis) over other cations such as Li^+^, Na^+^, K^+^, Ca^2+^, Mg^2+^, Mn^2+^, Hg^2+^, Fe^2+^, Fe^3+^, Co^2+^, Ni^2+^, Cu^2+^, Pb^2+^, Cd^2+^, and Zn^2+^ in a DMSO/H_2_O (1 : 9 v/v) mixture. In a paper strip assay, where the probe was simply impregnated onto test papers, the LoD was calculated to be 1 ppb (1 × 10^−5^ M for probe L-coated strips, Fig. 30b). Additionally, probe L enabled the detection of Al^3+^ in rice seedlings incubated with this ion. Fluorescence measurements of extracts from Al^3+^-treated rice seedlings showed a mild fluorescence at 50.0 μM Al^3+^, with maximal fluorescence intensity observed at 200 μM Al^3+^.

#### 2.4.2 Artificial metalloenzyme bioprobes

Regarding phytohormones, ethylene is a challenging metabolite to detect because it lacks targetable functional groups for conventional electrophilic or nucleophilic probes. Ethylene plays a crucial role in regulating numerous aspects of plant growth, immunity, development and senescence.^623,624^ For example, exogenous ethylene sources can greatly accelerate abscission and ripening in planta,^625^ and agricultural research is partly also focused on the development of improved sensors for ethylene gas. Current sensing tools for ethylene detection in plants generally rely on: (i) electrochemical sensors, chromatography, and laser-based techniques, *i.e.*, photoacoustic spectroscopy;^626^ (ii) genetically encoded fluorescent proteins (*e.g.*, EBS:GUS);^627^ (iii) chemical probes that are based on metal complexes.^628–630^ However, metal complexes have several practical limitations, such as decomposition in water and metal quenching in complex biological environments. Therefore, advanced strategies are required to provide them with the necessary stability. For this goal, artificial metalloenzymes (ArM) have been exploited, incorporating transition metal catalysts into a protein scaffold, *e.g.*, streptavidin,^631–633^ or myoglobin.^634^

**Fig. 31:**
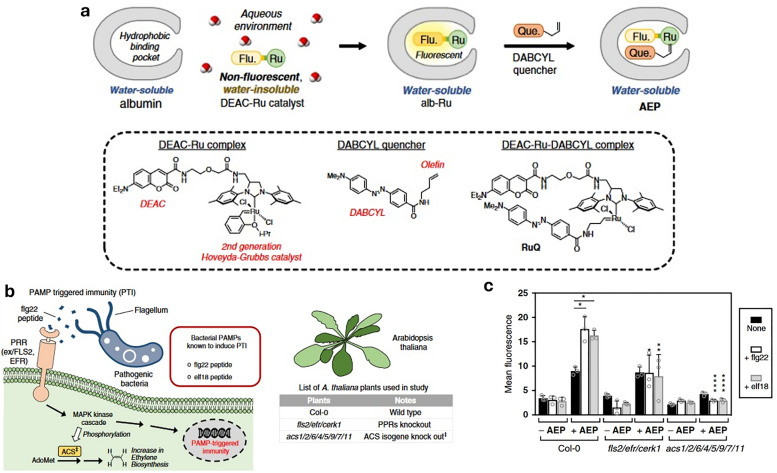
(a) Scheme of the general steps involved in converting albumin into the ethylene-detecting AEP probe. The chemical structures of the DEAC–Ru complex, DABCYL quencher, and RuQ are shown. (b) Illustration of the pathway leading to the pathogen-associated molecular pattern (PAMP)-triggered immunity (PTI) and subsequent ethylene production. A list of *A. thaliana* plants used in this experiment is also shown. (c) Summary of the fluorescence measurements under the various experimental conditions studied. Fluorescence and brightfield imaging (×40 magnification) of epidermal peels treated with AEP (100 μM) for wild-type Col-0 are presented. Figure adapted with permission from ref. 494.

Recently, a novel ArM biosensor, *i.e.*, ArM ethylene probe (AEP), was developed by the group of Tanaka^494^ for spatiotemporal detection of ethylene gas in fruits and *Arabidopsis* leaves (Fig. 31). The probe made use of a scaffold of human serum albumin (HSA): in the hydrophobic binding pocket, the bound metal complex was composed of (i) the fluorophore 7-diethylaminocoumarin (DEAC), (ii) the second generation Hoveyda–Grubbs catalyst,^635^ and (iii) the DABCYL quencher, giving FRET interactions. In the presence of ethylene, the complex catalysed a cross-metathesis reaction, releasing DABCYL and turning on DEAC fluorescence. The LoD was 34.4 μL of ethylene (∼27 ppm in air) *in vitro*. To be highlighted that AEP is one of the first reported methods to analyse ethylene in living samples with spatial and temporal precision. However, its size (∼66 kDa) prevented it from crossing the cell membrane, so it was limited to extracellular detection, and its adhesion to waxy plant surfaces diminished over time. Furthermore, its responsiveness was not yet fast enough for real-time measurements. Despite these limitations, the AEP proved to be a promising tool for non-invasive ethylene analysis in plant biology.

In summary, it can be concluded that the analyte scope to which (supramolecular) probes can be nowadays applied remains somewhat limited, as many detection systems rely on a – to some extent – selective reaction with a functional group commonly found in numerous pesticides and biomolecules in plants. To address this limitation, new strategies could include designing more selective probes through classical covalent chemistry, introducing novel concepts such as dynamic covalent chemistry, or combining host–guest complexation with covalent chemistry. These approaches could thus significantly advance the development of innovative probes. Furthermore, employing multicomponent analysis methodologies^636^ could assist in overcoming these challenges by producing fingerprint signals, thereby facilitating analyte discrimination. Therefore, further experiments in this area, following the principles of the so-called “chemical noses”,^637^ will be highly interesting. Moreover, the combination of chemosensors and probes could be explored to further enhance detection capabilities.

However, it must be emphasised that a significant disadvantage of reactive probes lies in their inability to function as dynamic systems, as is the case with chemosensors. This fact limits the ability of these reactive probes to detect dynamic changes in analyte concentrations, as they lack an equilibration mechanism with the analyte itself. Another important limitation is represented by the scarce information available regarding probe uptake mechanisms in planta distribution and elimination. These aspects are critical for implementing new sensor technologies in plant sciences and should be addressed in future research.

### 2.5 Nanosensors

#### 2.5.1 Fluorescent nanosensors

Before the introduction of synthetic luminescence-based nanosensors for use in plant detection, the first nanosized structures employed were based on fluorescent proteins (FPs). Thus, from a historical perspective, these examples are important. Indeed, not long after the group of Tsien and co-workers^638^ reported the first genetically encoded FPs for the detection of calcium ions in plant stromata, or small organic molecules, *i.e.*, carbohydrates.^256,639^ Several other examples of synthetic nanosensors have been reported that are not genetically encoded, which will also be discussed. A summary is found in Table 7.

**Table 7:** Representative list of supramolecular nanosensors discussed. Reported are the analytes with the related binding affinities in brackets: (—) indicates no binding affinity given

Nanosensor		Detection mode	Analytes (binding affinity)	LoDs and/or detection ranges	Media	Ref.
Carbon nanotubes	ss(GT)_15_-(7,6)SWCNT and ss(AT)_15_-(6,5)SWCNT	Luminescence	H_2_O_2_	100 μM in water and mM range in plant	*Arabidopsis thaliana* leaves	640
	ss(GT)_15_-(7,6)SWCNT and PVA-(6,5)SWCNT			500 μM in water and mM range in plant	*Arabidopsis thaliana* leaves	640
	HeAptDNA-SWCNT	Luminescence	H_2_O_2_	10–100 μM in TES buffer and 100 μM range in plant	*Arabidopsis thaliana* leaves	641
	Phospholipid-coated SWCNT	Luminescence	Polyphenols, *i.e.*, genistein and THP	100 μM in plant	Toccoa leaves	642
	PVA- and bombolitin II-coated SWCNTs	Luminescence	Picric acid	400 μM in plant	*Spinacia oleracea* leaves (inside)	643
	Polymer coated-SWCNTs	Luminescence	Synthetic auxins, *e.g.*, NAA or 2,4-D (*K*_a_,_NAA_ = 1.10 × 10^4^ M^−1^*K*_a_,_2,4-D_ = 3.60 × 10^4^ M^−1^)	8.2 μM (NAA), 0.35 μM (2,4-D) in MES buffer. Imaging in plant leaves possible	*Arabidopsis thaliana* and pak choi leaves	471
	CNTs	Amperometry	Indole-3-acetic acid	10 μM range	Surface of *Zea mays*	644
Gold/silver NPs	AuNPs	Luminescence	Sucrose	2.00 nM	Acetate buffer. Imaging of onion membranes	645
	AuNPs	SERS	Thiobendazole	mM range on the plant surface	Tomato plant surface	646
	AgDeNPs@MHQ	SERS	H_2_O_2_	—	*Oxalis corniculata* leaves	647
	DNA-functionalised Ag-coated Au nanorods (AgNS@Ag)	SERS	miRNA (miR858)	—	Plant leaf (not specified)	648
	DNA-functionalised Ag-coated Au nanorods (AgNS@Ag)	SERS	miRNA (miR156)	60.0 fM in PBS buffer and at 0.20 μM in plant	*Arabidopsis thaliana* leaves	649
	Ag NPs deposited on ZnO–Co_3_O_4_ core–shell shell nanoparticles	SERS	Triazophos, fonofos and thiram	1.00 nM (triazophos),100 nM (fonofos) 1.00 μM (thiram) in water. Low μM range in plants		650
	Halothiol-functionalised AuNPs immobilised on rGO	Chemoresistance	VOCs	10.0 μM range	*P. infestans* sporanglia leaves	651
Quantum dots	Boronic acid capped CdTe QDs	Luminescence	Glucose	>500 μM	*Arabidopsis thaliana* leaves	652
Metal–organic framework	HPR-loaded ZIF-8	Luminescence	H_2_O_2_/ROS	100 μM range on plants	On the leaf, petiole or root of *Nicotiana benthamiana*	653
	[Tb_2_(BDC)_3_(H_2_O)_4_]	Luminescence	Ag^+^, Cd^2+^, Cu^2+^, Fe^3+^, aniline	0.46 μM (Ag^+^) 44.0 nM (Cd^2+^) 20.4 μM (Cu^2+^)	In the vascular system of *Syngonium podophyllum*	479
	Cu-MOF	Electrochemical	Salicylic acid	150 μM range	In cucumber seedlings	654
	Co/Fe(TA)-MOFs	Electrochemical	BF_4_^−^, PF_6_^−^, OTf^−^, ClO_4_^−^	nM range	—	506
Polymer–protein hybrids	PNC–roGFP	Luminescence	ROS	—	On *Nicotiana benthamania* leaves	655
Polymeric nanochannel	Nafion nanotubes	Electrochemical	H_2_O_2_	5.00 nm in water, nm range in protoplasts	In protoplasts	656

**Fig. 32:**
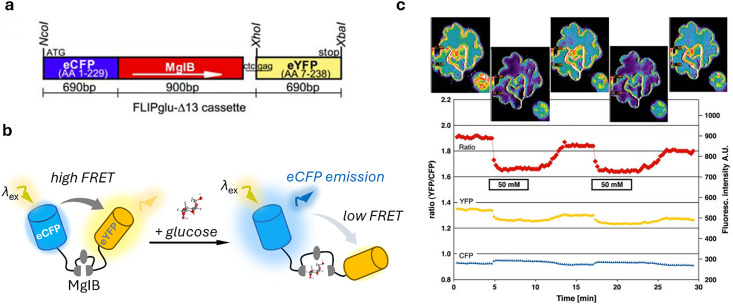
(a) FLIPglu-D13 cassette containing linearly fused eCFP-mglB-eYFP genes. The size of each gene, restriction sites, and transcription start and stop are indicated. (b) Schematic working principle of the glucose-sensitive FLIP nanosensor. (c) Glucose-induced FRET signal changes in the cytosol of leaf epidermal cells. Figure adapted with permission from ref. 657.

In particular, the use of fluorescent and genetically encoded protein nanosensors in living plants was described in a seminal paper by the group of Frommer.^657^ In this example, the authors described the detection of glucose in the leaves and intact roots of *Arabidopsis thaliana* using a nanosensor (Fig. 32a) composed of two FPs, *i.e.*, eCFP (FRET donor) and eYFP (FRET acceptor), which are translationally fused to an affinity mutant of the glucose binding protein, mglB. Besides, site-directed mutagenesis was used to generate a series of affinity mutants with *K*_d_ for glucose of 170 nM, 2.00 μM, 600 μM, and 3.20 mM in 20.0 mM MES/Tris buffer at pH 7.0. The detection principle was based on the observation that a FRET signal can be detected in the absence of glucose, as eCFP and eYFP are in close spatial proximity, leading to sensitised emission of the acceptor (Fig. 32b). In the presence of glucose, which binds to the recognition domain of the nanosensor, a conformational change is induced, increasing the distance between the two FPs and thus attenuating the FRET process. With this type of nanosensors, the flux of glucose in the mM regime was detected in leaves (range 1.00–50.0 mM, Fig. 32c) and roots (range 0.25–5.00 mM).

The same group has used FLIP-based nanosensors for pH-insensitive detection of glucose and sucrose (μM range) in root tips^658^ and for detecting cytosolic glucose levels^659^ (μM range) in *Arabidopsis thaliana*. Recently, Frommer, Jones, and co-workers reported using a protein-based FRET biosensor to detect various gibberellins (GAs), which are important phytohormones for plant growth and development in the roots of *Arabidopsis* seedlings.^660^ This nanosensor (GSP1) consists of two fluorescent FRET protein pairs, *i.e.*, edeCFP (donor) and edAFP (acceptor), linked *via* the GA binding domain (*K*_d,G4_ = 24.0 nM, *K*_d,G3_ = 240 nM, *K*_d,GA1_ = 110 nM in 50.0 mM MOPS pH 7.4). When employing a targeted core variant of GPS1 (nlsGPS1), the authors showed that exogenous GA4 (dose: 1.00 μM) could be detected as it results in increased nlsGPS1 FRET emission ratios specifically in the elongation zone of roots. In contrast, other GAs, *i.e.*, GA_1_ and GA_3_, do not elicit a signal response, indicating their altered bioaccumulation mechanism.

**Fig. 33:**
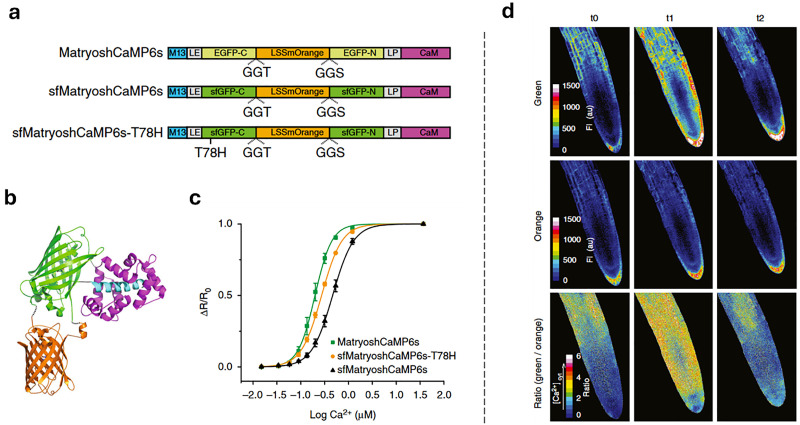
(a) Schematic representation of MatryoshCaMP6s sensors, composed of GO-Matryoshka (LSSmOrange sandwiched between the C and N termini of either EGFP, sfGFP, or sfGFP-T78H) inserted between the M13 peptide and calmodulin domain. (b) Schematic representation of a MatryoshCaMP6s sensor based on X-ray crystal structure data. (c) Calcium-affinity titrations (*I*_510nm_/*I*_570nm_ ratio). (d) Average *z*-stack projections of confocal images showing *Arabidopsis* lateral root before NaCl (Ca^2+^ flux trigger) treatment (*t*_0_ = 100 s) and after treatment (*t*_1_ = 186 s; *t*_2_ = 334 s). Figure adapted with permission from ref. 661.

In the same year, Gaulin and co-workers reported the use of genetically encoded protein-based nanosensors to detect protein–nucleic acid interactions, *i.e.*, RNA or DNA, at the subcellular level in plants.^661^ To this end, proteins capable of associating with specific nucleic acids, *i.e.*, the Aaecrn13 effector from the oomycete *Aphanomyces euteiches* and the defensive transcription factor AatWrKY22 from *Arabidopsis*, were labelled with GFP, that serves as FRET donor through standard methods for the *in situ* generation of fusion proteins. The protein–DNA interaction was imaged by fluorescence microscopy after plant leaves containing the nanosensor were fixed and treated with Sytox Orange, a nucleic acid dye that acts as a FRET acceptor. Consequently, the FRET signal was detected solely when the nanosensor bound nucleic acids of a specific sequence, as both the donor and acceptor pairs were in close proximity in that case. Besides detecting small organic molecules and nucleic acid polymers, the detection of Ca^2+^ using protein-based fluorescent nanosensors has also been reported.^638,662–664^ In plants, the cytosolic Ca^2+^ concentration generally ranges between 100 and 200 nM, whereas in certain organelles it can even reach mM levels.^665,666^ For example, the detection of Ca^2+^ ions using the calcium-binding GCaMP6s recognition moiety, which was covalently linked to two FRET-active fluorescent proteins using different dpFP variants (Matryosh sensor), was reported by Frommer and co-workers (Fig. 33).^667^ In this way, the authors prepared a series of nanosensors with affinities for Ca^2+^ varying from *K*_d_ = 197 ± 23 nM (MatryoshCaMP6s, Fig. 33a and b), 271 ± 10 nM (sfMatryoshCaMP6s-T78H), 303 ± 28 nM (sfGCaMP6s-T78H), 481 ± 45 nM (sfGCaMP6s), 501 ± 64 nM (sfMatryoshCaMP6s) in 10.0 mM K_2_EGTA, 100 mM KCl, 30.0 mM MOPS pH 7.2 (Fig. 33c). Specifically, the MatryoshCaMP6s nanosensor (Fig. 33a and b) was used to detect the cytosolic Ca^2+^ flux in *Arabidopsis* seedlings and mammalian cells (Fig. 33d).

In addition to the aforementioned examples, two FP-based nanosensors have been reported for detecting the plant hormone abscisic acid in the roots of *Arabidopsis*, with affinities of *K*_d_ = 2.00–80.0 μM^668^ and *K*_d_ = 100–600 nM, respectively.^669^ Recently, Rizza and co-workers reported the detection of the growth-regulating hormone gibberellin in the roots of *Arabidopsis thaliana* using a genetically encoded fluorescent biosensor, *i.e.*, nlsGPS,^670^ which exhibited a low micromolar binding affinity for this hormone.^660^ The detection of indole-3-acetic acid, one of the major regulatory small molecules in the root tip of individual seedlings of *Arabidopsis*, was then recently reported by Höcker, Jürgens, and co-workers, who developed a nanosensor based on mNeonGreen-Aquamarine-TrpR for this purpose, referred to as “AuxSen” (*K*_d_ = 2.00–8.00 μM).^671^ As for non-organic small molecules, FPs have also been reported for detecting reactive oxygen species, which have already been reviewed elsewhere.^672^

Although genetically encoded biosensors offer versatile options for creating ratiometric FRET-based nanosensors *in situ*, this technology remains, at the moment, limited to genetically modified *Arabidopsis* (see examples above) and rice.^673^

**Fig. 34:**
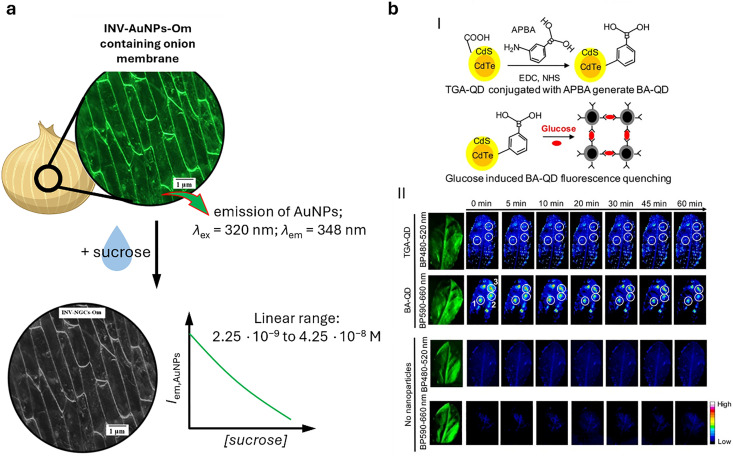
(a) Schematic representation in the fluorescence-response of INV-AuNPs-Om. The images show confocal fluorescence microscopy images of INV-AuNPs-OM before and after incubation with sucrose-containing solution (the image after the addition of sucrose has been adapted and modified for visual representation). The presence of glucose stains the fluorescence of AuNPs in a concentration-dependent manner. (b) (I) Schematic representation of surface functionalisation of QDs with boric acids and their aggregation induced by glucose, which in turn leads to attenuation of their fluorescence; (II) glucose detection in *Arabidopsis* leaves using the QD fluorescent probe in the presence of TGA-QD and BA-QD (top two rows) and the absence of the nanosensors (bottom two rows). Images were recorded with two Raspberry Pi cameras equipped with bandpass optical filters (BP 480–520 nm and BP 590–660 nm for TGA-QD and BA-QD, respectively). Figure adapted with permission from ref. 645.

In a recent example, Chiang and co-workers showed that the bio-catalysed formation of gold nanoparticles (AuNPs) from AuCl_4_^−^ solutions in onion membranes can be used for the detection of sucrose (Fig. 34a).^645^ The catalysed formation of AuNPs can be attributed to the activity of the invertase enzyme in conjunction with reducing agents such as flavonoids, vitamin C, and thiosulfonates, which facilitate the reduction of Au salts. In essence, the authors succeeded in forming AuNPs *in situ* within onion membranes through the above-discussed biocatalysed process. Sucrose detection was achieved by monitoring the fluorescence of the INV-AuNPs-Om. This nanosensor has a dynamic range for sucrose between 2.25–43.0 nM concentrations in acetate buffer (20.0 mM, pH 5.0), with a response time of 30 s and a LoD for sucrose of 2.00 nM.

In 2018, Giraldo and co-workers reported a ratiometric fluorescent nanosensor for *in vivo* detection of glucose in the single chloroplast of algal cells (*Chara zeylanica*) and plant leaf tissue (*Arabidopsis thaliana*) at concentrations greater than 500 μM (*in vivo* experiments) using confocal microscopy (Fig. 34b).^652^ To this end, the authors prepared two types of CdTe quantum dots (QDs): first, QDs capped with thioglycolic acid (TA), which remained invariant to glucose (TA-QDs) changes and served as an internal fluorescent reference control for ratiometric detection. Secondly, QDs conjugated with boronic acid (BA), which quenched their fluorescence in response to glucose (BA-QDs), and were therefore used as the sensing unit. Particularly, the quenching of BA-QDs in the presence of glucose occurs due to the cross-linking of glucose by the reaction of its diol functional groups with the surface-bound boric acid moieties of the QDs, which caused aggregation-induced fluorescence quenching. The BA-QDs showed selective aggregation response in the presence of glucose, whereas other sugars without *cis*-diol functionality, *i.e.*, fructose, galactose, and mannose, did not cause significant photophysical changes.

**Fig. 35:**
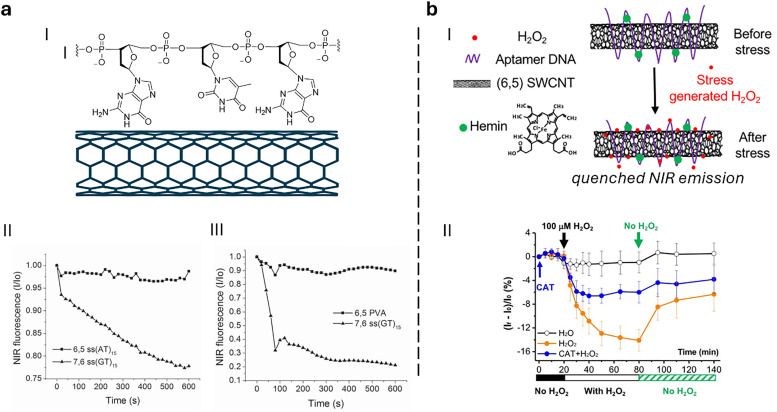
(a) (I) Truncated and simplified structure of ss(GT)_15_-wrapped SWCNT. (II) Temporal changes in 6,5 and 7,6 SWCNT peak intensity in the presence of H_2_O_2_ (100 μM). (III) Temporal changes in 6,5 and 7,6 SWCNT peak intensity in the presence of NO (500 μM). (b) (I) Structure and general working principle of HeAptDNA-SWCNT used for the detection of H_2_O_2_. (II) NIR intensity changes in response to H_2_O_2_ (100 μM) added topically on the leaf surface. Sensor emission quenches upon exposure to H_2_O_2_, followed by partial recovery and stabilisation of the luminescence signal in the absence of H_2_O_2_. Figure adapted with permission from ref. 640.

Furthermore, Strano and co-workers reported the ratiometric detection of H_2_O_2_ or NO in plant leaves by capitalising on the Corona phase molecular recognition (CoPhMoRe) phenomenon observed with SWCNTs.^640^ For the detection of H_2_O_2_, the a ss(GT)_15_ nucleic acid-wrapped 7,6 SWCNTs (*λ*_em_ = 1131 nm; Fig. 35a) was used, whose fluorescence was quenched in the presence of H_2_O_2_ (100 μM in water). Ratiometric detection was made possible including also ss(AT)_15_-wrapped 6,5 SWCNTs (*λ*_em_ = 984 nm), which did not elicit any response in the presence of H_2_O_2_ and therefore served as internal fluorescence reference (Fig. 35a). For the detection of NO (500 μM in water), the authors used the ss(GT)_15_-wrapped 7,6 SWCNTs (*λ*_em_ = 1135 nm) as NO-responsive element, while the PVA-wrapped 6,5 SWCNTs (*λ*_em_ = 1004 nm) is used as reference. The fluorescence quenching mediated by H_2_O_2_ and its radical species, *e.g.*, OH˙, was attributed to the reversible charge transfer quenching occurring when such chemical species adsorb on the nucleic acid-wrapped SWCNTs. In addition, the radical species can oxidise the purine bases of the DNA coating of the SWCNTs, thereby changing the polarity of the corona, resulting in an attenuation of the fluorescence response through a modified charge transfer process.^674^ Regarding the detection of NO, the primary mechanism of its fluorescence quenching by the SWCNTs can be explained through the previously described electron transfer process.^675,676^ The SWCNT nanosensors were introduced into *Arabidopsis thaliana* leaves by excising leaf sections and treating the sections with solutions of equimolar mixtures of the nanosensors. After 3 hours of incubation, the leaf samples were treated with a solution of H_2_O_2_ (10.0 mM) or NO (50.0 mM) and then subjected to fluorescence analysis using an infrared-sensitive microscope.

In addition, Kruss, Giraldo, and co-workers reported SWCNTs coated with hemin and DNA aptamers (HeAptDNA-SWCNT; Fig. 35b) for the detection of H_2_O_2_ (10.0–100 μM) in TES buffer (10.0 mM, pH 7) and in *Arabidopsis thaliana* leaves by infiltration of the peroxide (100 μM) into the plant.^641^ It is known that the accumulation of H_2_O_2_ is a hallmark of the plant stress response,^677,678^ but current precision agriculture tools often detect stress only after detrimental effects have already occurred.^679,680^ This nanosensor addresses this gap by facilitating *in vivo*, remote NIR imaging of plant health in response to environmental factors and pathogens stresses. More in detail, the nanosensor was prepared by wrapping the heme-aptamer polymer around the 6,6-enriched SWCNT samples. In the presence of H_2_O_2_, this nanosensor responded with quenched NIR emission, which can be explained by an analogous mechanism as described by Strano and co-workers. The detection of H_2_O_2_ was also possible after irradiation of the plant with UVB light or after the perfusion of a pathogen-like peptide (flg22) known to induce ROS formation in plants. The presence of ROS, the presence of Ca^2+^, sucrose, glucose, methyl salicylate, abscisic acid, and jasmonic acid did not cause any significant interference. It must be noted that this is the first known example of a sensor capable of reacting to H_2_O_2_ in the physiological range of the plant.

**Fig. 36:**
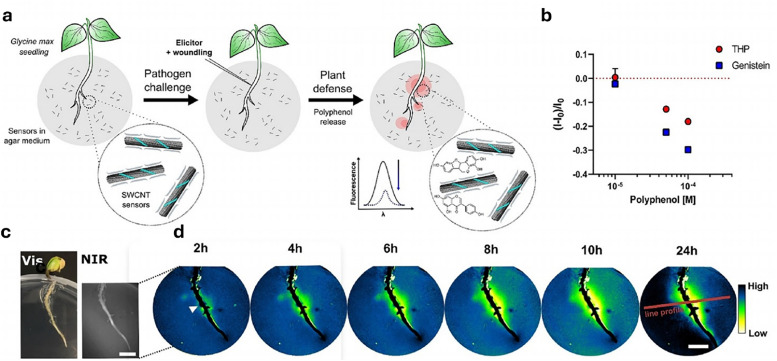
(a) Schematic illustration of sensor incorporation into plants through an agar medium enriched with nanosensors. As the soybean seedlings grow in this agar, the plants are challenged with a pathogenic trigger, while polyphenol release in response to this challenge is monitored *via* NIR imaging. (b) Genistein, and trihydroxypterocarpan (THP) as prominent components of the soybean polyphenol profile quench the fluorescence of PEG-PL-SWCNTs in agar. (c) Visible and NIR image of the soybean seedling (scale bar = 1 cm). (d) NIR response close to the challenged root position (root tissue is overlayed with black; white triangle = position for elicitor induction; red line is the line profile position, scale bar = 1 cm). Figure adapted with permission from ref. 642.

In a recent example, Kruss and co-workers reported a SWCNT-based NIR nanosensor for detecting polyphenols and pathogen-induced polyphenol accumulation in *Toccoa* leaves (Fig. 36a).^642^ The authors investigated various SWCNs coatings with single-stranded DNA (ssDNA) of different nucleotide sequences and polyethylene glycol (PEG)-phospholipid macromolecules for their fluorescence response in the presence of various polyphenols, *i.e.*, tannic acid, ellagic acid, resveratrol, caffeic acid, gallic acid methyl ester, cyanidin-3-sambubioside, delphinidin-3-sambubioside, chlorogenic acid, catechol hydrate, genistein, and trihydroxypterocarpan. It was found that most ssDNA-SWCNTs responded with a fluorescence increase to the presence of polyphenols, whereas PEG-PL-SWCNTs and PEG-phospholipid-SWCNTs responded with a fluorescence decrease. Other aromatic compounds, such as salicylic acid and methyl 3,4,5-trimethoxybenzoate, did not cause any change in the fluorescence response. Although the fluorescence response of ssDNA-coated SWCNTs was inconsistent, PEG-phospholipid-coated SWCNTs exhibited a clear emission response that depended on polyphenol concentration (*K*_d_ = 91.0 nM for tannic acid) (*K*_d_ = 91.0 nM for tannic acid) and saturated at lower mM levels range. Plant roots were grown on agar enriched with PEG-phospholipid SWCNTs to map the presence of polyphenols in soybean. Genistein (100 μM) and THP (100 μM) were selected as representative polyphenols to test the nanosensor response after treating the roots with the polyphenol solutions (Fig. 36b). After 30 minutes of post-treatment, a fluorescence intensity decrease of about 30% was observed, while potential interfering substances, such as sugars or H_2_O_2_, did not cause any detectable response. The authors also observed significant NIR attenuation when their nanosensor was applied to mechanically damaged soybean roots (Fig. 36c). Mechanical stress also occurred with parasite infestation, suggesting that this nanosensor can, in principle, be used to monitor the presence of parasites.

**Fig. 37:**
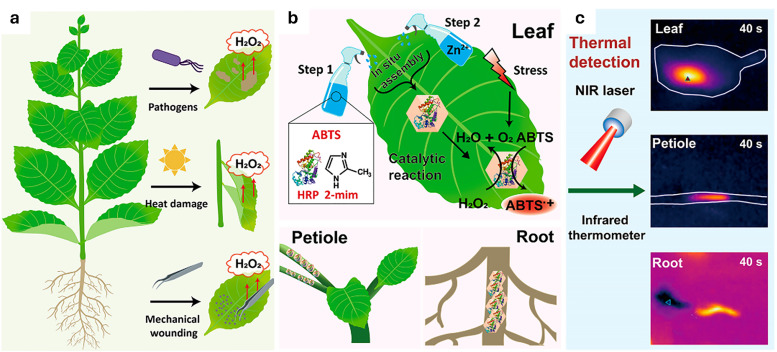
(a) Schematic representation of the external factors that induce ROS, *e.g.*, H_2_O_2_, formation in plants. (b) The MOF nanosensor was used to detect H_2_O_2_ on plant leaves, stems, and roots. (c) ABTS˙^+^ generates thermal signals under a NIR laser that are detectable by a thermometer. Figure reproduced with permission from ref. 653.

More recently, Wang, Lee, and co-workers utilized an imidazolate scaffold, (ZIF-8) MOF, for the detection of H_2_O_2_ and ROS in plant roots, petioles, or leaves (Fig. 37a) at concentrations in the 0–100 μM range.^653^ The MOF, which carries horseradish peroxidase (HPR), and the reporter dye, 2,2-azino-bis(3-ethylbenzothiazoline-6-sulfonic acid) (ABTS), were prepared *in situ* on plant leaves through a two-step procedure (Fig. 37b). First, a solution containing HPR, ABTS, and 2-methylimidazole was sprayed onto the desired surface of the plant (leaf, petiole, or root). Then, a solution containing Zn^2+^ ions was sprayed onto the treated surfaces, resulting in the formation of the final MOF-based nanosensor, in which the reporter dye and the peroxidase are encapsulated in the metal–organic framework. The sensing mechanism of this nanosensor is based on the peroxidase activity, which in the presence of H_2_O_2_ oxidises the reporter dye to its radical cation (ABTS˙^+^) and which can be detected by thermal cameras through its heat emission when excited by NIR light (Fig. 37c).

**Fig. 38:**
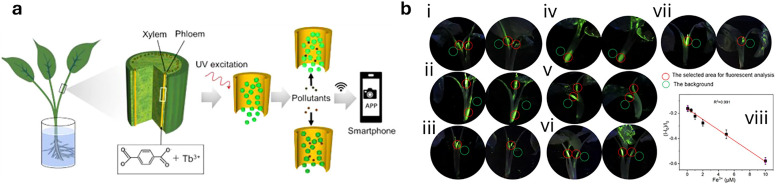
(a) Illustration of MOF–plant nanobiohybrids for environmental pollutant sensing. (b) Representative photos of fluorescence emissions from MOF–plant nanobiohybrids were taken under a UV lamp (320 nm) as a function of Fe^3+^ concentration in aqueous solutions. The left columns are images of blank nanobiohybrids, and the right columns are the corresponding images of nanobiohybrids after incubation with Fe^3+^ for 4 hours: (i) 0.05 μM; (ii) 0.1 μM; (iii) 0.5 μM; (iv) 1 μM; (v) 2 μM; (vi) 5 μM; and (vii) 10 μM. (viii) Their fluorescence emission dose responses were analysed by ImageJ. Figure adapted with permission from ref. 479.

Another MOF-based sensor, specifically a luminescent lanthanide (Ln)-MOF-plant nano biohybrid,^681^ was reported by Yun, Liang and co-workers^479^ as living sensors for the on-site detection of environmental pollutants. This was achieved by integrating luminescent MOF of [Tb_2_(BDC)_3_(H_2_O)_4_] (BDC = terephthalate) into the vascular system of *Syngonium podophyllum* plants (Fig. 38). The accumulation of environmental pollutants – including toxic metal ions such as Ag^+^, Cd^2+^, Cu^2+^, Fe^3+^, and organic compounds such as aniline – causes measurable changes in luminescence intensity: an enhanced response for Ag^+^, Cd^2+^, and aniline, and a decreased response for Fe^3+^ and Cu^2+^. The nanohybrids showed superior sensitivity in water with detection limits of ∼50.0 μg L^−1^ (0.46 μM) for Ag^+^, 5.00 μg L^−1^ (0.044 μM) for Cd^2+^, and 1.30 mg L^−1^ (20.4 μM) for Cu^2+^, showing linearity ranges of 0.05–0.50 μM for Ag^+^, Cd^2+^, and aniline (5% accuracy), and of 0.05–10.0 μM for Fe^3+^ and Cu^2+^ (10% accuracy). The detection mechanism involves energy transfer through host–guest interactions; for example, intersystem crossing and efficient energy matching between Tb^3+^ ions and ligands promote energy transfer, leading to luminescence changes. Visual signals under UV light can be readily detected and transformed into digital information through a smartphone app, facilitating on-site monitoring of environmental pollutants with commendable sensitivity specificity.

**Fig. 39:**
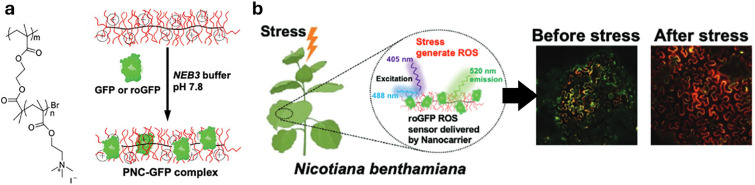
(a) Chemical structure of the polymer and the process of protein grafting onto cationic PNCs. (b) *In vivo* plant stress imaging by the PNC–roGFP complex sensor in *Nicotiana benthamiana*, tomato, and maize plants. Fluorescence microscopy images showing the detection of ROS by PNC–roGFP in *Nicotiana benthamiana* leaves. Figure adapted with permission from ref. 655.

The Marelli group^655^ reported high-aspect-ratio cationic polymer nanocarriers (PNCs) to efficiently deliver proteins into mature plants, overcoming the challenges posed by the size and weak charge of proteins (Fig. 39a). By complexing PNCs with the redox-sensitive green fluorescent protein (roGFP), they created a ratiometric stress sensor capable of penetrating plant cell walls and membranes. It is known that oxidation and reduction of cysteine residues in roGFP cause a change in the ratio of fluorescence intensity (520 nm), when excited at 405 nm and 488 nm (*R*_405/488_). Thus, this study exploited the fact that, under stress, plants over-accumulate ROS, such as H_2_O_2_, resulting in increased emission by roGFP when excited at 405 nm and a decreased emission when excited at 488 nm. The PNC–roGFP complex shows an increased sensitivity and response rate to ROS, possibly due to a local increase in free-ROS concentration surrounding the sensor through electrostatic interactions. *In vivo*, confocal microscopy in *Nicotiana benthamiana* (Fig. 39b) and *Arabidopsis thaliana* showed that PNC–roGFP sensors rapidly detect biotic stressors (wounding, pathogenic peptide flg22 exposure) and abiotic stressors (heat) by monitoring *R*_405/488_ variation. The sensor also enables species-independent protein delivery, ROS-selective stress detection, and reversibility *in vivo*. While the PNC platform effectively delivers small, stable sensor proteins, the delivery of larger, less stable cargo, such as Cas9 ribonucleoprotein complexes, remains challenging. Future research aims to optimise PNC design and protein loading to expand application possibilities. Furthermore, the development of portable sensor technologies is necessary to transition from laboratory-based microscopy to field applications. The successful delivery of PNC–protein into various plants will pave the way for new technologies in plant sensing and engineering, contributing to the sustainable large-scale production of food, energy and functional materials.

Contamination of crops by nitroaromatic compounds is a severe safety concern. The Strano group prepared SWCNTs coated with polyvinyl alcohol (PVA) and bombolitin II for the detection of nitroaromatic compounds.^643^ Specifically, in the presence of picric acid, the NIR emission of the nanosensor decreased (*λ*_ex_ = 785 nm). When the nanosensor is introduced into the leaf of a *Spinacia oleracea* plant together with a picric acid-invariant set of SWCNTs (serving as reference), it is possible to image the subsequent infiltration of picric acid (400 μM) into the plant using an NIR-sensitive microscope.

**Fig. 40:**
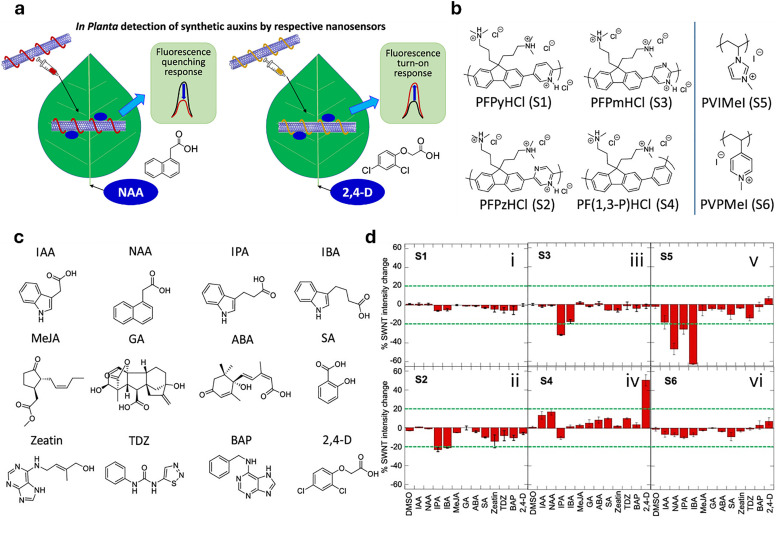
(a) Real-time sensing of NAA and 2,4-D uptake in hydroponically grown pak choi and rice plants using SWCNT nanosensors. (b) Chemical structures of the cationic polymer series, comprising (i) a polyfluorene (PF)-based A–B copolymer backbone and (ii) poly(4-vinylpyridine) (PVP) and poly(*N*-vinylimidazole) (PVI) backbones. (c) Chemical structures and abbreviations of the screened plant hormones. (d) *In vitro* screening results of SWCNTs against plant hormone analytes for: (i), S1; (ii), S2; (iii), S3; (iv), S4; (v), S5; and (vi), S6. Figure adapted with permission from ref. 471.

Later, in 2021, the same group exploited CoPhMoRe sensors made of SWCNTs coated with a library of cationic polymers, *i.e.*, an A–B copolymer backbone based on (a) polyfluorene (PF) and (b) poly(4-vinylpyridine) (PVP) and poly(*N*-vinylimidazole) (PVI) backbones, for the detection in planta of synthetic auxins (Fig. 40a), including NAA and 2,4-D.^471^ These polymer-wrapped SWNTs interact with auxin-derived anionic analytes through π–π and electrostatic interactions, allowing for selective detection through NIR fluorescence modulation. Thus, the CoPhMoRe platform shows a ratiometric response to the presence of auxins with a 51% turn-on response for 2,4-D and a 50% quenching response for NAA, displaying dissociation constants *K*_d_ = 28.0 μM for 2,4-D and *K*_d_ = 91.0 μM for NAA, with LoDs of 0.35 μM and 8.20 μM, respectively (in 10 mM MES buffer containing 10.0 mM MgCl_2_, pH 5.5). Tested analytes (Fig. 40c) included natural auxins – 3-indole acetic acid (IAA), 3-indole propionic acid (IPA), and 3-indole butyric acid (IBA); synthetic auxins – NAA and 2,4-D; and additional hormones, such as zeatin, thidiazuron (TDZ), 6-benzylaminopurine (BAP), methyl jasmonate (MeJA), gibberellic acid (GA), abscisic acid (ABA), and salicylic acid (SA). Imaging of these phytohormones was possible in plat leaves of *Arabidopsis thaliana*, *Brassica rapa*, and *Oryza sativa*, underscoring their utility in planta auxins detection and in studying auxins dynamics and herbicide susceptibility across species and media, promising enhanced tools for agricultural and plant biological research.

#### 2.5.2 SERS-based nanosensors

**Fig. 41:**
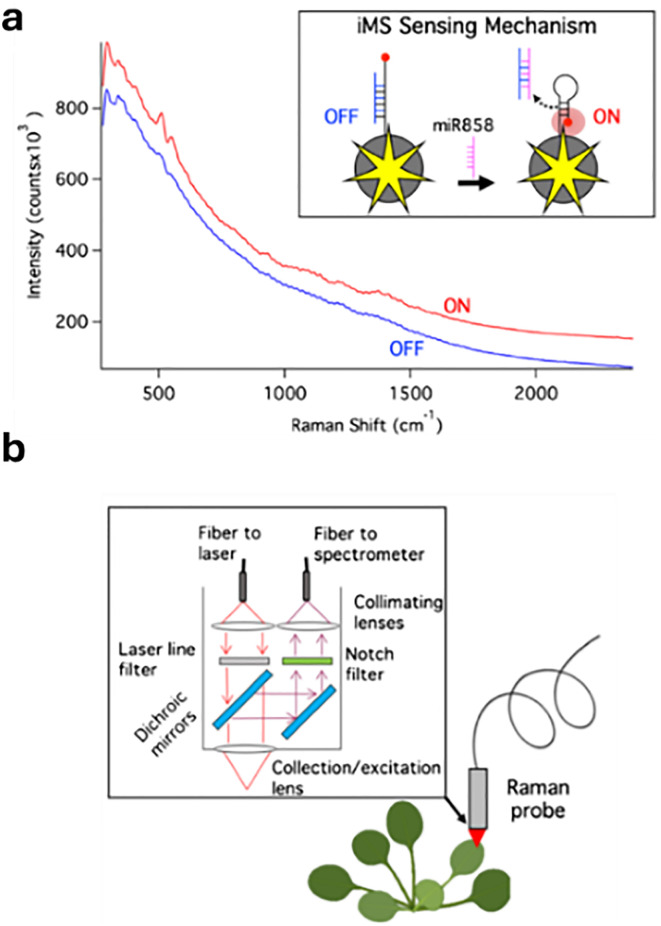
(a) Schematic representation of the working principle for the detection of miRNA. (b) Schematic representation of the optical setup used for the SERS-based miRNA in plants. Figure adapted with permission from ref. 648.

Vo-Dinh reported the use of silver-coated gold nanorods that were functionalised with a s ssDNA capable of binding to a complementary miRNA (iMS nanosensors; Fig. 41).^648^ Furthermore, the tail of the ssDNA was covalently linked to the SERS reporter, *i.e.*, cyanine7 (Cy7; Fig. 41a). In its “off” state, the Cy7 reporter is kept at a fixed distance from the surface of the nanoparticle due to the presence of a placeholder strand, which was hybridised with the reporter ssDNA. The placeholder strand could hybridise with the targeted miR858 analyte, leaving a flexible reporter strand on the surface of the NP. In this state, the Cy7 dye is brought in proximity to the NP surface, as the unhybridised reporter strand has formed a hairpin-like conformation (Fig. 41a). In this “on” state, the Raman scattering intensity of the dye increases. The SERS-active nanosensor was transfected into plants by injecting it into the leaves, where the authors showed that the nanosensor responded to artificially injected miR858 when the Raman signals were analyzed by shifted excitation Raman difference spectroscopy (SERDS) – a procedure that corrects for strong background signals (Fig. 41b).

In more recent work, Vo-Dinh and co-workers presented the time-dependent miRNA (miR156) detection in *Arabidopsis* leaves using SERS nanosensors based on AuNS@Ag, functionalised with a DNA strand.^649^ The detection mechanism was analogous to that previously described by the group using iMS sensors (see example above). Here, the functional DNA reporter conjugate was designed to alter its conformation in the presence of the target miRNA, using the distance dependence of the SERS signal from Cy7 as a transduction mechanism. In this way, miR156 can be detected with a LoD of 60 fM (in PBS buffer solution containing 0.01% Tween-20) and imaged in plant leaves (at a miRNA dose of 0.20 μM).

**Fig. 42:**
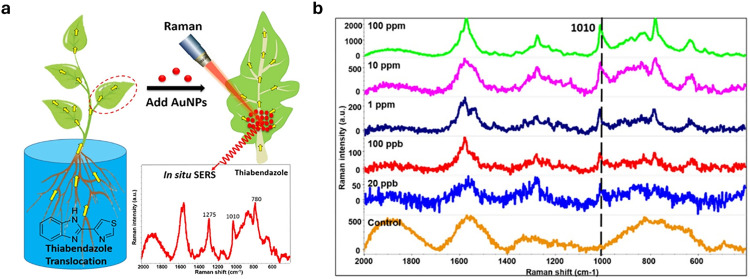
(a) SERS-based detection method of thiabendazole on tomato plant leaves. (b) Thiabendazole-dependent SERS spectra recorded on tomato plant leaves. Figure adapted with permission from ref. 649.

He and co-workers used AuNPs to detect the pesticide thiabendazole (exposure at 1.00 mM) in tomato plants when it was introduced into the hydroponic systems for growing the plants (Fig. 42a).^646^ SERS signals were recorded *in situ* after drop-casting an AuNPs-containing solution onto the plant tissue (Fig. 42b). The same group previously employed a similar approach for the detection of isocarbophos and phorate (both organophosphonates), deltamethrin (a pyrethroid), and imidacloprid (a neonicotinoid) – all insecticides – at low μM concentrations.^682^

In the same year, Lei and co-workers also reported the detection of the pesticide methyl parathion on the surface of fruits/leaves at 110–440 ng cm^−2^.^683^

**Fig. 43:**
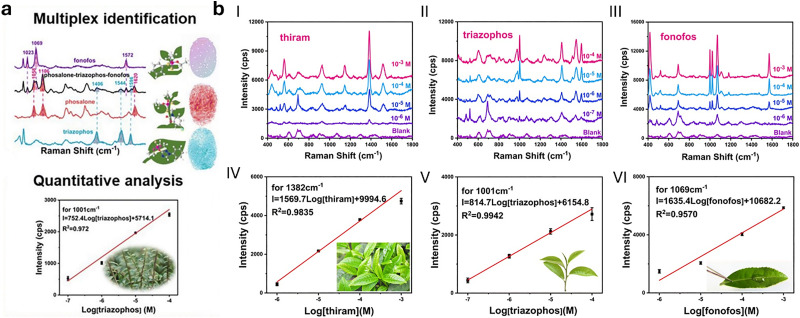
(a) SERS signals of different pesticides can be used for their multiplex identification and quantitative detection in plant leaves using ZnO@Co_3_O_4_@Ag NPs. (b) SERS spectra of (I) thiram, (II) triazophos, and (III) fonofos on tea leaves using ZnO@Co_3_O_4_@Ag NPs as SERS-active components. Corresponding linear regression curves (IV–VI). Figure adapted with permission from ref. 650.

Recently, Zhang, Huang, Jiang, and colleagues used ZnO-core Co_3_O_4_-shell nanoparticles, onto which silver nanoparticles were surface-deposited (ZnO@Co_3_O_4_@Ag), for multiplex SERS-based detection of the pesticides triazophos (LoD = 1.00 nM), fonofos (LoD = 100 nM), and thiram (LoD = 1.00 μM) in water and at low μM concentrations on plant leaves (Fig. 43).^650^

**Fig. 44:**
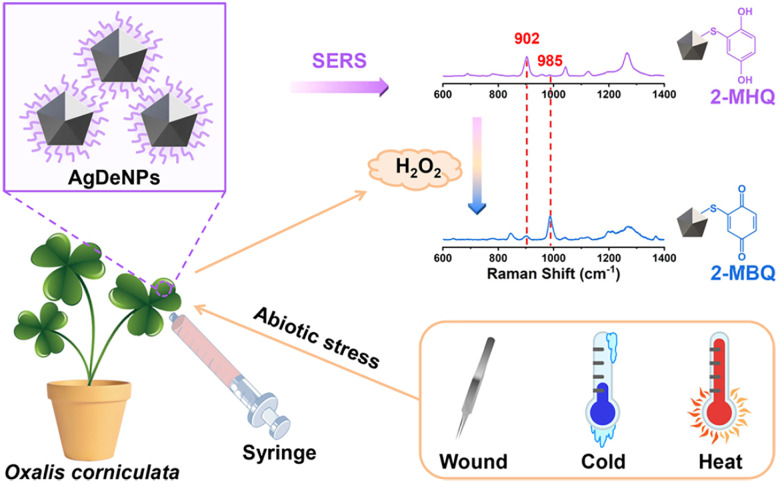
Schematic illustration of the SERS-based detection of H_2_O_2_ by AgDeNPs@MHQ in *Oxalis corniculata* leaves subjected to abiotic stresses, such as heat and mechanical damage. Figure reproduced with permission from ref. 647.

In addition, Niu and coworkers^647^ reported an *in vivo* SERS-sensor for non-destructive, *in situ*, and highly sensitive imaging of H_2_O_2_ in plant leaves (Fig. 44). The SERS-sensor consisted of decahedral, SERS-active Ag nanoparticles with an average size of 90.0 nm, capped with 2-mercaptohydroquinone (2-MHQ), which served as probe for H_2_O_2_, since it can be oxidised to 2-mercaptobenzoquinone (2-MBQ) by H_2_O_2_. Thus, the SERS intensity ratio of the oxidation-related peaks of 2-MBQ to the non-oxidation-related peaks of 2-MHQ can be reliably used to determine the concentration of H_2_O_2_ and monitor its dynamics. The infiltration of 2-MHQ-functionalised Ag nanoparticles (AgDeNPs@MHQ) into *Oxalis corniculata* leaves *via* needle infiltration enabled the detection of ROS generated from H_2_O_2_ production in response to stressors, such as mechanical damage and temperature changes (4 °C *vs.* 30 °C). Fluorescently labelled nanoparticles showed that the nanosensor accumulated in the stomatal pores on the epidermis and the intercellular spaces of mesophyll cells. Importantly, SERS detection was performed using a 785 nm laser 30 minutes after infiltration. The use of 785 nm excitation light minimised interference from chlorophyll autofluorescence in the cells.

#### 2.5.3 Electrochemical and chemoresistance-based sensors

In this subchapter, we will present some exemplary cases of (micro)electrode-based sensors, noting that microelectrodes are not nano-sized systems but are instead comprised of nanoparticles. We also direct the reader to other recently published literature reviews on electrochemical sensors in plant research.^684,685^

**Fig. 45:**
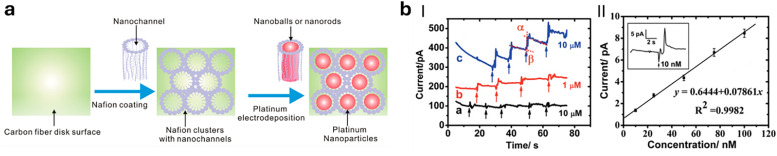
(a) Schematic representation of the formation of Nafion nanochannels on the surface of the microelectrodes. The pores of the nanochannels were filled with Pt nanoparticles in a second step by an electrodeposition process. (b) (I) Amperometric response curves of platinum deposited microelectrodes (black cure) and NPts/CFMDE (red and blue curves) to a series of increases of H_2_O_2_ concentration in a stirred deaerated PBS solution (pH 7.4). (II) The calibration curve for H_2_O_2_ solution over the concentration range from 10 to 100 nM, and the amperometric response to 10 nM H_2_O_2_ is magnified in the inset. Figure adapted with permission from ref. 686.

In 2009, Huang and co-workers reported a microelectrochemical sensor for the amperometric detection of H_2_O_2_ (LoD = 5.00 nM) in aqueous solutions and individual plant protoplasts, allowing real-time visualisation of oxidative bursts (Fig. 45).^686^ In this case, the electrode system consisted of carbon ultramicroelectrodes, surface functionalised with Nafion-based nanochannels (Fig. 45a). The presence of these polymer nanochannels facilitated the subsequent electrodeposition of platinum NPs, which resulted in Pt particles with well-defined and homogeneous nanostructures and a high specific surface area – prerequisites that allowed for the acquisition of an amperometric current due to the oxidation of H_2_O_2_. With these nanoparticle-bearing microelectrodes (NPts/CFMDE), the detection of ROS-mediated stress was achieved in aqueous solutions (Fig. 45b) and in single protoplasts with rapid response times (milliseconds). Later, Huang, Huo, and co-workers used nanowire functionalised microelectrodes for amperometric detection of vesicular exocytotic auxin efflux from single plant protoplasts with nM sensitivity.^656^

Despite their clear advantages, including simplified fabrication of wearable devices and straightforward real-time signal readout, electrochemical microsensors struggle with a low signal-to-noise ratio and temporal drift. However, a self-referencing electrochemical microsensor can self-correct for environmental drift and noise through phase-sensitive filtering,^687^ based on Fick's first law of diffusion. Real-time flux measurements are indeed based on the oscillatory translation of the probe and the quantification of concentration differences of the analyte between two spatial positions separated by a known excursion distance.

Therefore, Porterfield and co-workers reported a self-referencing electrochemical microsensor for the non-invasive amperometric detection of endogenous indole-3-acetic acid (IAA) flux (*ca.* 10.0 μM) on the surface of *Zea mays* roots without the addition of exogenous IAA.^644^ The presented microsensor was modified by using platinum black and carbon nanotubes (CNTs) on the surface, while the microelectrodes were made of tapered Pt wires (tip diameter of 2.00–4.00 μm) and insulated with parylene. Other potential interfering analytes (10.0 μM), such as citrate, oxalate, malate, ascorbate, nitric oxide, glucose, malate, citrate, oxalate, NaNO_3_, NH_4_NO_3_, Ca(NO_3_)_2_, NaH_2_PO_4_, MgSO_4_, KCl, CuSO_4_, KH_2_PO_4_, KNO_3_, MnCl_2_, NaN_3_, and the herbicide 2,4-dichlorophenoxyacetic acid (2,4-D), did not interfere with the detection of IAA. Notably, although the sensor response to ascorbate reached 28% of that observed for IAA, ascorbate is not considered a relevant interferent since it is not released as a root exudate. These findings support the suitability of this method for real-time monitoring of IAA transport in surface tissues and demonstrate its compatibility with existing live imaging techniques.

**Fig. 46:**
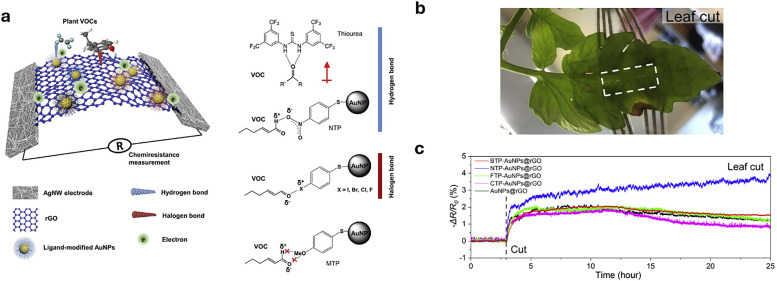
(a) Schematic representation of the soft and wearable electrochemical sensor for the chemiresistive detection of VOCs. The gold nanoparticles deposited on the surface of the reduced graphene oxide layer, which can be functionalised with various ligands, enabled hydrogen bond-assisted detection of VOCs. (b) Photograph of the location of the wearable sensor and the mechanical damage site. (c) Response curves of the 5-channel sensor array after a mechanical cut on the leaf. Figure adapted with permission from ref. 651.

In addition, Zhu, Wei, and co-workers reported a chemo-resistive sensor for real-time detection of volatile organic compounds (*e.g.*, aldehydes, ketones, alcohols) on living tomato plants (Fig. 46).^651^ Authors prepared two different types of reduced graphene oxide (rGO)-based sensors: (i) rGO nanosheets functionalised with 1,3-dis[3,5-bis(trifluoromethyl)phenyl]thiourea, which can form strong hydrogen bonds with carbonyl groups, and (ii) receptor-functionalised AuNPs (ligand-modified AuNPs, Fig. 46a). The surface receptor molecules of the AuNPs were halothiophenols *i.e.*, iodothiophenol (ITP), bromothiophenol (BPT), chlorothiophenol (CTP), and ([fluorothiophenol]) FTP, a nitrothiophenol (NTP; hydrogen interaction), and a methoxythiophenol (MTP; served as a control). These electropositive surface modifications can form halogen-bonding interactions with electron donors such as pyridine or pyrrole, resulting in negative resistance changes. In contrast, the binding of electron donors to the thiourea@rGO resulted in positive changes. Since most plant VOCs are rich in nitrogen- or oxygen-containing functional groups, the different hydrogen or halogen bonding interactions with the VOCs could be used for multiplex detection of structurally similar plant volatile compounds. Thus, 13 different VOCs (at 10 ppm) were detected in the air using a sensor array of 8 functionalised chemiresistive sensors (1 min exposure). When applied to plant leaves (Fig. 46b), which were incised to emit the VOCs, simulating physical stress from plant insects, a saturation of the sensor response occurred after several hours. Furthermore, elevated VOCs were detectable when tomato plants were mechanically cut (Fig. 46c) or exposed to *P. infestans* sporangia.

Recently, Li, Chen and co-workers reported the detection of SA *in vivo* in cucumber seedlings at concentrations around 150 μM.^654^ The electrochemical sensor was functionalised with a copper-based MOF (Cu-MOF), prepared from Cu(NO_3_)_2_ and 2-amino terephthalic acid. The stability of the Cu-MOF in air and aqueous environment was enhanced by mixing the particles with carbon black powder, and in combination with Nafion, the resulting solution was used to modify the working electrode. The detection of SA was possible because the authors observed that the peak current for the reduction of the copper sponge was significantly reduced in the presence of the analyte. Thus, this suggests that Cu-MOF was able to catalyse the oxidation of SA while Cu^2+^ was reduced to Cu^+^, which, in turn, led to a lower current upon Cu^2+^ reduction potential application.

**Fig. 47:**
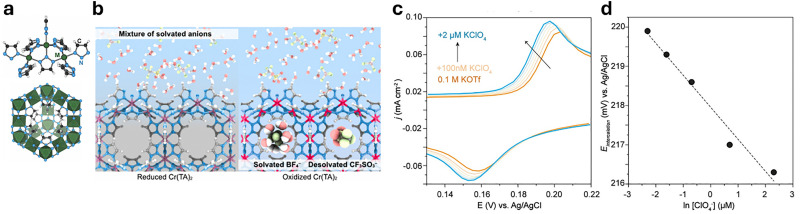
(a) Cr(TA)_2_ and Fe(TA)_2_ nanoparticles. Shown are secondary building unit clusters of M(TA)_2_, M = Fe or Cr and the idealised representation of Cr/Fe(TA)_2_ pore structure based on the bulk crystalline structure. (b) Representation of Cr(TA)_2_ pores before (left) and after (right) oxidation-induced anion intercalation. (c) Sensing of ClO_4_^−^ anions using a Cr(TA)_2_ nanoparticle film in aqueous solution by CV measurements and (d) the variation of *E*_1/2_ for the intercalation redox feature during titrations of KClO_4_ into a 0.1 M KOTf aqueous electrolyte solution. Figure adapted with permission from ref. 506.

By further exploiting the characteristics of MOFs, the Brozek group^506^ developed an electrochemical anion sensing method employing nanocrystalline films of conductive nano-MOF layers (approximately 20 nm) made from chromium- or iron-based materials. These films are capable of trapping larger-charge balancing anions such as BF4^−^, PF6^−^, OTf^−^, and particularly ClO_4_^−^, which is a common environmental pollutant that is highly soluble and stable in water, classified as a threat to environmental and food safety by the EPA. The reported MOFs comprise Cr or Fe cations coordinated by 1,2,3-triazolate (TA) ligands, which serve as organic cross-linkers. Further exploiting MOFs characteristics, the Brozek group developed an electrochemical anion sensing method using nanocrystalline films of conductive nano-MOF layers (∼20 nm) made of chromium- or iron-based materials, able to trap larger-charge balancing anions such as BF_4_^−^, PF_6_^−^, OTf^−^, and especially ClO_4_^−^, a common environmental pollutant highly soluble and stable in water, classified as threatening environmental and food safety by the EPA. The reported MOFs consisted of Cr or Fe cations coordinated by 1,2,3-triazolate (TA) ligands, acting as organic cross-linkers. DFT calculations showed that Cr-based nano-MOF had larger pore entrances than Fe(TA)_2_ nanopores. Therefore, while Fe(TA)_2_ only contained fully desolvated BF_4_^−^ anions, Cr(TA)_2_ allowed the incorporation of partially solvated BF_4_^−^ anions at shifted potentials – from about 1.2 V *vs.* Fc^0/+^ in Fe(TA)_2_ to about −0.6 V *vs.* Fc^0/+^ in Cr(TA)_2_. The larger pore size of Cr(TA)_2_ improved anion gating and enabled a shift from solvated BF_4_^−^ transfer to complete desolvation and intercalation of larger anions such as OTf^−^, accompanied by anodic redox potential shifts of more than 500 mV (Fig. 47c). This anion-dependent redox chemistry enabled the sensitive detection of ClO_4_^−^ in aqueous solutions at concentrations as low as 100 nM. In addition, the sensors retained their structural stability for over a month and were reusable after applying a negative voltage for the deintercalation of ClO_4_^−^. Therefore, this resulted in the first sensor being able to detect several anions simultaneously. Cr-nanoMOFs surpass commercial alternatives regarding selectivity, stability in aqueous solutions, recyclability, and detection limits, establishing Cr(TA)_2_ nanocrystals as promising supramolecular transducers for redox voltammetric anions sensing.

In summary, the examples presented in this chapter clearly indicate that nanosensor designs often offer enhanced detection capabilities. For instance, in fluorescence-based detection, quantum dots and carbon dots have several advantages over traditional molecular dyes, including improved photostability and increased brightness. Moreover, the near-infrared fluorescence of SWCNTs enables significantly deeper light penetration through biological tissues compared to visible light. This feature renders SWCNTs particularly advantageous for in-planta applications. Furthermore, carbon-based nanomaterials (such as SWCNTs and carbon dots) demonstrate minimal environmental impact; however, their effects on cellular metabolism remain an unresolved issue. Furthermore, nanocarriers can be functionalised with performance-enhancing features, such as dyes, polymers, or organelle-specific targeting units. For instance, targeting units for chloroplasts already exist, and future research could aim to expand this capability to target other areas of plant cells. In addition, porous nanoparticles serve as highly attractive carriers for hosting, stabilising, and effectively delivering nucleic acid-based cargo to cells, potentially paving the way for new gene therapies. However, further research is required to fully understand the mechanisms of nanoparticle uptake, including how material composition and morphology influence the ability of nanoparticles to cross plant cell barriers. It is also crucial to consider the size limitations that affect the permeability of nanoparticles through plant membranes. Among silica-based materials, the capacity to make them stimuli-responsive, enabling them to disintegrate on demand, opens up exciting possibilities for spatio-temporal pesticide release and strategies to minimise nanomaterial accumulation in plants, which could otherwise lead to adverse effects on plant health responses.

## 3. Delivery systems

### 3.1 Design considerations for delivery applications to plants

Designing an effective plant delivery system, whether based on supramolecular host–guest complexes or nanoparticles, requires careful consideration of morphological and physicochemical properties. Factors such as size, shape, surface roughness, charge, and coatings (*e.g.*, polymeric shells or targeting units) influence in planta accumulation and biodistribution (see Section 1.6).^39^ However, their behaviour should be assessed not only under controlled laboratory conditions but also in real-world environments, where interactions with salts, proteins, carbohydrates, and cells may hinder successful delivery through physical adsorption.^148,688–692^

Particularly, some critical challenges can be highlighted in the preparation of multicomponent-based nanomaterials, which must be highly controlled and characterised to achieve optimal reproducibility and performance, required for translation and approval by state agencies for commercialisation. Primarily:

• having information on the stability of deliver vehicles during preparation and long-term storage is crucial, not only in water but also in real samples, such as biofluids, water, soil, and under atmospheric conditions (*e.g.*, light exposure, temperature, and humidity changes). To assess stability, complementary characterisation techniques, including electron microscopy, dynamic light scattering, and small-angle X-ray scattering, should be employed. Pre-evaluation tests help guide material selection, ensuring stability in both colloidal and solid states, given that nanoparticles may be applied *via* foliar sprays, soil amendments, or direct deposition;

• for nanomaterials, homogeneity in morphology (size and shape) and surface functionalisation, such as decoration with targeting units or dyes, must be thoroughly analysed to minimise batch-to-batch variability and potential reproducibility issues;

• standardised delivery protocols, including nanoparticle concentrations, doses, and plant growth conditions, are crucial for assessing delivery vehicles and ensuring study comparability and reproducibility. Indeed, the rigid plant cell wall, absent in animal cells, hinders biomolecule uptake, slowing genetic engineering advances in plants. Since mammalian models are unsuitable, dedicated research must clarify plant-specific uptake mechanisms. Standardising procedures and comparing results with prior studies will be essential for meaningful insights;

• additionally, release kinetics must be carefully studied and characterised, as fast or slow release profiles can impact delivery efficacy. Understanding the fate of nanomaterials post-release and their exclusion mechanisms could further refine mRNA-based delivery strategies. This is especially relevant for nanoparticles applied externally, such as those in foliar sprays or root infiltration methods;

• eventually, the toxicity of the vehicles to both mammals and non-mammals must be well documented, and their persistence in soil and potential distribution in the food chain must be thoroughly assessed for risk. Using non-toxic components, ideally pre-approved by regulatory agencies, such as the FDA or EMA, may be preferable. However, improving the translation of research from universities to companies, spin-offs, or startups and bringing a product to market requires stronger communication and collaboration across organisations.

As it can be seen, this list indicates the need of an intense and dedicated effort for the development of effective delivery systems. We are trying to underline these aspects as presented in the literature, but a higher attention should be paid in future research to address these needs.

### 3.2 Delivery examples based on macrocyclic hosts and their micellar and vesicular assemblies

Supramolecular systems have been explored to improve the delivery and, consequently, the efficacy of agriculturally relevant substances. Macrocyclic hosts discussed in Section 1.3 are capable of forming host–guest or association complexes with pesticides through a combination of the supramolecular interactions described in Section 1.2. These interactions are non-covalent, enabling both the complexation and controlled release of pesticide molecules. For instance, physicochemical changes, such as pH shifts that alter the protonation state and consequently the charge of the host or guest, can be employed to trigger release, as will be discussed in the following sections. Similarly, photo responsive units such as azobenzene moieties can act as molecular switches, allowing precise temporal control over pesticide deployment and thereby supporting sustainable pest management and environmentally conscious agricultural practices. The use of macrocyclic hosts offers significant advantages, including improved pesticide stability against deactivation (*e.g.*, chemical degradation), enhanced solubility, and increased leaf wettability. Encapsulation also mitigates toxicity. For example, host–guest complexes are less readily absorbed by mammalian cells, resulting in safer and more us er-friendly formulations.

The use of synthetic macrocyclic hosts for pesticide delivery was already reported in the 1990s.^693,694^ Early studies by Maeda, Tsuji, Muramoto and co-workers highlighted the protective effect against the thermal decomposition of pesticides when they form inclusion complexes with βCD, as observed with dimethoate (*O*,*O*-dimethyl-*S*-(*N*-methylcarbamoyl-methyl)phosphorodithioate)^695^ or salithione (2-methoxy-4*H*-1, 3,2-benzodioxaphosphorin-2-sulfide).^696^ Katsuda and co-workers reported on βCD inclusion complexes of pyrethroids, such as permethrin, (*S*)-α-cyano-3-phenoxybenzyl (1*R*)-*cis*-3-(2,2-dibromovinyl)-2,2-dimethylcyclopropanecarboxylate (NRDC 161), and fenvalerate, describing the reduced volatility of these compounds when complexed with CD. While free pyrethrins and resmethrin quickly lose activity by photodecomposition, the included preparations remained active even after 2 weeks of exposure to sunlight.^697^ Further early examples of the protective effect of pesticides in the form of their host–guest complexes with CDs were found with pesticides such as sulprofos (*O*-ethyl *O*-[4-(methylthio)phenyl] *S*-propyl phosphorodithioate),^698^ aldicarb (7,7-dimethyl-4-oxa-8-thia-2,5-diazanon-5-en-3-on),^698^ molinate (*S*-ethyl-*N*,*N*-hexamethylene-thiocarbamate),^699^ bentiocarb (*S*-4-chlorobenzyl-diethylthio-carbamate),^699^ dichlorphos (2,2-dichlorovinyl-dimethyl-phosphate),^700^ fenitrothion (*O*,*O*-dimethyl *O*-(3-methyl-4-nitrophenyl) phosphorothioate),^701^ malathion (diethyl 2-[(dimethoxyphosphorothioyl)sulfanyl]butanedioate),^701^ fenson (4-chlorophenyl benzenesulfonate),^702^ chlorfenson (4-chlorophenyl 4-chlorobenzenesulfonate),^702^ genite (2,4-dichlorophenyl benzenesulfonate),^702^ or warfarin (4-hydroxy-3-(3-oxo-1-phenyl-butyl)chromen-2-one).^703^ Aside from pesticides, host–guest complexes of insect repellents, *e.g.*, diethyltoluamide (*N*,*N*-diethyl-3-methylbenzamide),^701^ or pesticide synergists, such as MGK-264 (*N*(2-ethylhexyl)-8.9,1 *O*-trinorborn-*S*-ene-2,3-dicarboximide),^701^ as well as plant growth regulators, such as ethephon ((2-chloroethyl)phosphonic acid),^704^ have been reported in those early years. Since then, agrochemical products to improve crop production and crop protection using macrocycles, mainly CDs, have been introduced to the market.

Several water-soluble macrocyclic hosts that are not CDs, such as CB*n*, CX*n*, and PA*n*, have demonstrated low toxicity. However, their commercialisation is limited due to the lack of FDA or EPA approval, and to their relatively high synthesis and purification costs compared to the biotechnological production of CDs. In this context, other reviews have discussed the protective effect of inclusion and host–guest complexation on pesticides.^705^

As mentioned in the introduction, we also included examples from patent literature on the practical application of macrocyclic host molecules and bioactive molecules in plant sciences, which are summarised in Table 8. The use of CDs prevails by large; however, other hosts such as CB*n* or PA*n*-based macrocycles have been reported.

**Table 8:** Representative list of patents related to the application of supramolecular host–guest systems

Year	Hosts	Guests	Effect	Ref.
1974	αCD, βCD	Resmethrin	Improved stability	706
		Furamethrin		
		Tetramethrin		
		Proparthrin		
		5-Propargyl-α-ethynyl-2-furylmethyl chrysanthemate		
1985	αCD, βCD, γCD	Piperonyl butoxide	Improved efficiency	707
1986	βCD	Benomyl	Improved bioavailability	708
		BCM		
		Metomeclam		
		Phenamirol		
1986	βCD	Benezensulfonamides	Improved stability	709
		Chlorosulfuron		
1987	βCD and γCD	Benomyl	Improved activity	710
		Benzimidazole of abem(2-carbomethoxy)		
1989	αCD, βCD	Amitraz	Improved formulation for dust and spray applications	711
1990	αCD, βCD, γCD	Benomyl	Improved bioavailability	712
		BCM (2-carbomethoxyamino-benzimidazole)		
		Metomeclan		
		Phenamirol		
1992	βCD	Isoxaben	Improved activity	713
1993	αCD, βCD, γCD	Benzohydroxy-moylazole derivatives	Prolonged activity	714
1995	αCD, βCD, γCD	Cartap hydrochloride	Improved stability	715
		Nitenpyram		
		Allethrin		
		Acephate		
		Oxydeprofos		
		Vamidothion		
		Trichlorfon		
		Validamycin A		
		Diquat		
		Bialaphos		
1996	αCD, βCD, γCD	Nitenpyram	Improved stability	716
		Cartap hydrochloride		
		Bensultap		
		Fenitrothion		
		Acephate		
		Ferimzon/fthalide mixture		
1996	CDs	Pyriproxyfen	Improved stability	717
1996	αCD, βCD, γCD	Cartap hydrochloride	Improved efficiency, controlled release	718
		Clothianidin		
		Imazosulfuron		
1999	αCD, βCD, γCD, and their methyl-, 2-hydroxypropyl-, acetyl-derivatives	Bitertanol	Sap flow delivery	719
		Propoxur		
		Transfluthrin		
		Cyfluthrin		
		Transfluthrin		
2000	αCD, βCD, γCD [(2,6-di-*o*-methyl)-β-cyclodextrin]	Azadirachtin-A	Increased stability	720
2000	αCD, βCD, γCD	Folithion	Improved efficacy	721
		Bismethylarsine		
		Carbamyl-lindane		
		Parathion		
		Rogor		
		Phosmet		
		Dimehypo		
		Isopropyl methoxalamine		
		Butachlor		
		Dichloroquinolinic acid		
		Sulfadiazine		
		Bensulfuron-methyl		
		Metsulfuron-methyl		
		Pyrazosulfuron		
		Tribenuron-methyl		
		Fenclorim		
		Fenchlorazole		
		Gibberellin		
		Cytokinin		
		Jinggangmeisu		
		Topsin		
2001	αCD, βCD, γCD, and their C1–4 alkyl-, C1–4 alkanoyl-, C1–4 hydroxyalkyl-derivatives	Diflufenican	Improved efficacy	722
		Picolinafen		
		TTP (4-(3-trifluoromethylphenoxy)-2-(4-trifluoromethylphe-20 nyl)-pyrimidine)		
2001	αCD	Cyclopropene	Improved storage	723
		Methylcyclopropene		
		Cyclopentadiene		
		Diazocyclopentadiene		
2001	CB8	Paraquat	Release features	724
2002	*N*,*N*-Dimethyl-*N*-dodecyl-functionalised βCD	Glyphosate	Improved stability	725
2002	αCD, βCD, γCD	Methoxone phenoxy acetic acid butyl ester	Improved efficacy	726
		Methoxone phenoxy propionic acid isopropyl ester		
		Butachlor		
		Bbenthiocarb		
		Bensulfuron-methyl		
2006	αCD, βCD, γCD	Imidacloprid	Improved efficacy	727
	Hydroxypropyl βCD	Acetamiprid		
		Thiamethoxam		
		AKD1022 ((2*E*)-1-[(2-chloro-1,3-thiazol-5-yl)methyl]-3,5-dimethyl-*N*-nitro-1,3,5-triazinan-2-imine)		
2009	βCD	Citral	Improved stability and controlled release	728
2010	βCD	Pymetrozine	Improved efficacy	729
2011	βCD	Polyethyleneglycol aryloxyacetate	Improved efficacy	730
2015	αCD, βCD, γCD, and their hydroxypropyl-, methyl-, and sulphated derivatives	Bifenthrin	Improved safety	731
		Tebuconazole		
		Bendiocarb		
		Acetamiprid		
		Alpha-cypermethrin		
2018	αCD, βCD, γCD	DEET (*N*,*N*-diethyl-meta-toluamide)	Improved efficacy	732
	Hydroxypropylated and methylated βCD			
	Sulfobutyl ether βCD			
	C1–C5 alkylated γCD			
2020	Hydroxypropylated βCD	Bifenthrin	Improved bioavailability	733
	Methylated βCD	Epoxiconazole		
	Hydroxypropylated γCD	Deltamithrin		
		Propiconazole		
		Prothioconazole		
		Tau-fluvalinate		
2021	Anionic and cationic pillar[5,6,7]arenes	Paraquat	Improved efficacy	734
		Diquat		
		DDT		
		Aldrin		
2022	αCD, βCD, γCD	Benfluralin	Improved efficacy	735
	Hydroxypropylated and methylated βCD	Butrualin		
	Sulfobutyl ether βCD	Chlornidine		
	C1–C5 alkylated γCD	Dinitramine		
		Dipropalin		
		Etalflularin		
		Fluchloralin		
		Isopropalin		
		Metalpropalin		
		Niitraline		
		Oryzarin		
		Pendimethalin		
		Prodiamine		
		Proflularin		
		Folpet		
		Captan		
		Manzeb		
		Chlorantraniliprole		
		Indoxacarb		
		Metaflumison		
		Pendimethalin		
2023	Cationic biphen[*n*]arene	Purpurine	Molecular recognition	736
		*o*-Phenanthroline		
		Paraquat		

A summary of all the collected examples of delivery systems of plant pesticides based on macrocycles is presented in Table 9.

**Table 9:** Summary of reported delivery examples based on macrocycles for plant pesticides

Guests	Hosts	Binding affinities	Guest role	Aimed improvement	Tested on	Ref.
1,3-Diphenylurea	βCD	250 M^−1^ (βCD)	Cytokine	Improved water solubility	Broccoli sprouts	737
	2HP-βCD	427 M^−1^ (2HP-βCD)				
		196 M^−2^ (2HP-βCD, 2 : 1)				
2-Amino-3*H*-phenoxazin-3-one	CB7	(1.80 ± 0.7) × 10^6^ M^−1^	Herbicide	Improved water solubility	—	738
2-Chloro-*N*-(thiophen-2-ylmethyl)pyridin-4-amine	βCD (1 : 1)	3.70 × 10^4^ M^−1^ (βCD)	Bactericide	Improved foliar wettability	Rice seedlings	739
	HP-βCD					
	Me-βCD					
2,4-Dichlorophenoxyacetic acid	CB7	—	Auxin	Potentiality of controlled release	—	740
2,2′-Disulfanediyldianiline	CB7	(3.90 ± 0.5) × 10^4^ M^−1^	Herbicide	Improved water solubility	Wheat coleoptiles	738
		(1.20 ± 0.4) × 10^5^ M^−1^ (for diammonium ion)				
2-Naphthalene-acetic acid	CB7	—	Auxin	Potentiality of controlled release	—	740
7-Hydroxyflavylium	CB7	—	Anthocyanine	Improved stability	—	741
	βCD					
	HP-βCD					
Arylazopyrazole	Guano-βCD	1.18 × 10^3^ M^−1^	Crosslinker for hydrogels	Supramolecular crosslinking	Chinese cabbage, *Alfalfa* seedlings	742
Bensulfuronmethyl	βCD	317 M^−1^ (βCD)	Herbicide	Increased water solubility	*Eclipta prostrata* (false daisy)	743
	2HP-βCD	278 M^−1^ (2HP-βCD)				
Carvone	ACB1	(3.50 ± 0.1) × 10^4^ M^−1^ (ACB1)	Essential oil	Decreased volatility	—	744
	ACB2	(1.50 ± 0.1) × 10^5^ M^−1^ (ACB2)				
Chlorpropham	βCD	370 M^−1^	Herbicide	Potentially improved water solubility	—	745
Cyanidin	CB7	—	Anthocyanine	Improved stability	—	741
	βCD					
	HP-βCD					
(*E*)-3,3′-((Diazene-1,2-diylbis(4,1-phenylene))bis(oxy))bis(1-((3-methoxybenzyl) (methyl)amino)propan-2-ol)	βCD	2.91 × 10^4^ M^−1^	Bactericide	Improved foliar wettability and foliar uptake	Rice plant	746
Gibberellic acid	βCD	(2.90 ± 0.6) × 10^3^ M^−1^ (βCD)	Auxin	Improved water stability	Cucumber seedlings	747
	γCD	(1.60 ± 0.3) × 10^3^ M^−1^ (γCD)				
	HP-βCD	(3.00 ± 1.0) × 10^3^ M^−1^ (HP-βCD)				
Imazalil	βCD	(5.30 ± 0.9) × 10^3^ M^−1^ (βCD)	Fungicide	Stimuli-responsive release (by cadaverine)	*In vitro*	748
	CB8	(2.10 ± 0.9) × 10^6^ M^−1^ (CB8)				
Imazilil	βCD	—	Fungicide	Increased stability	Citrus fruits	749
Indole-3-acetic acid	CB7	—	Auxin	Potentiality of controlled release	—	740
Iprodione	βCD	408 M^−1^	Fungicide	Increased water solubility	*In vitro*	750
Limonene	ACB1	(2.50 ± 0.1) × 10^4^ M^−1^ (ACB1)	Essential oil	Decreased volatility	—	744
	ACB2	(1.32 ± 0.08) × 10^5^ M^−1^ (ACB2)				
Monuron	βCD	292 M^−1^	Herbicide	Potentially improved water solubility	—	745
Pelargonidin	CB7	—	Anthocyanins	Improved stability	—	741
	βCD					
	HP-βCD					
Propanil	βCD	298 M^−1^	Herbicide	Potentially improved water solubility	—	745

#### 3.2.1 Delivery examples based on cyclodextrins

Schirra and co-workers^749^ reported on the complexation of imazalil (IMZ) – a fungicide constituent of deccozil – with βCD. The resulting host–guest complex (βCD⊃IMZ) was tested against *Penicillium digitatum* and *P. italicum* both *in vitro* and on inoculated grapefruits. Particularly, IMZ forms a stable 1 : 1 complex with βCD, which showed no decomposition after six months as a powder, and only minimal release in solution after 30 minutes at 50 °C. Fresh βCD⊃IMZ (0.00–0.20 μg mL^−1^) shows an efficacy to similar to the free IMZ, with a median effective dose, ED_50_, of 0.091 μg mL^−1^. However, 1 and 4-day-old mixtures result to be more potent, with ED_50_ of 0.079 μg mL^−1^ and 0.086 μg mL^−1^, respectively, whereas βCD alone does not inhibit fungal growth. βCD⊃IMZ (250 mg L^−1^) outperforms 1200 mg L^−1^ IMZ in fruit storage tests, including lemons, suggesting that lower doses of fungicide can be used when using βCD⊃IMZ.

Interestingly, βCD alone, as mentioned above, does not inhibit the growth of *P. digitatum* and *P. italicum*, which, however, is in contrast to earlier reports of its fungistatic properties against *Alternaria tenuis*, *Sclerotinia sclerotiorum* and *Rhizoctonia solani*.^751^

**Fig. 48:**
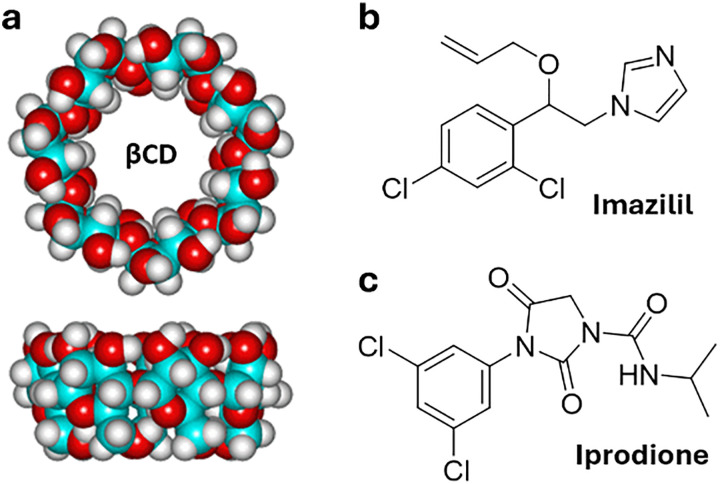
(a) 3D-rendering of βCD top and side view. Adapted with permission from Martin Chaplin (website: https://www.water.lsbu.ac.uk) chemical structures of the fungicides (b) imazilil and (c) iprodione.

Yang and co-workers^750^ reported the formation of an inclusion complex of iprodione (IPO) and βCD (βCD⊃IPO, *K*_a_ = 407.5 M^−1^ in water), as was determined *via* absorption spectroscopy through a phase solubility method and modelled *via* computational methods (Fig. 48a). Thus, the formation of the host–guest complex results in its doubled fungicidal activity against *Rhizoctonia solani* (half maximal effective concentration, EC_50,IPO_ = 1.74 μg mL^−1^*versus* EC_50,βCD⊃IPO_ = 0.76 μg mL^−1^) and *Physalospora piricola* (EC_50,IPO_ = 1.35 μg mL^−1^*versus* EC_50,βCD⊃IPO_ = 0.60 μg mL^−1^) *in vitro*, which can be attributed to the fungicides 4.7-fold increased water solubility when complexed by the cyclodextrin.

**Fig. 49:**
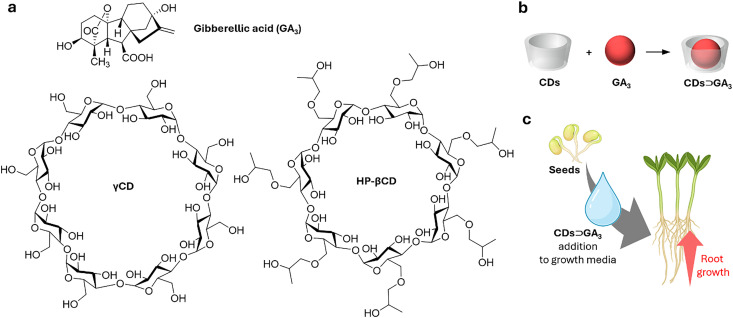
(a) Chemical structures of the plant auxin gibberellic acid (GA_3_), γCD and 2-hydropxpropyl-βCD (HP-βCD). (b) Schematic representation of the host–guest inclusion complex formation between CDs and GA_3_. (c) Schematic representation CDs⊃GA_3_ promoted root growth of cucumber seedlings (*C. sativus*). The plant seeds were put on filter paper in the Petri dishes filled with 5.0 mL diluents of different treatment solutions and were grown in a growth chamber for 5 days. Figure adapted with permission from ref. 747.

In addition, the phytohormone gibberellic acid (GA_3_) has been reported to form inclusion complexes with βCD, γCD,^747^ and 2-hydropxpropyl-βCD (HP-βCD), with *K*_a_ = (2.90 ± 0.6) × 10^3^ M^−1^, *K*_a_ = (1.60 ± 0.3) × 10^3^ M^−1^, and *K*_a_ = (3.00 ± 1.0) × 10^3^ M^−1^, respectively, for their 1 : 1 inclusion complexes (CDs⊃GA3) in water (Fig. 49). The host–guest complex formation improves the water solubility, chemical stability against hydrolysis in acidic or basic pH and thermal stability of GA_3_. Then, when CDs⊃GA_3_ was added to the medium for the growth of cucumber seedlings (*C. sativus*), it was found that GA_3_ and CDs⊃GA_3_ significantly enhances growth at concentrations of 5.00 mg L^−1^, 20.0 mg L^−1^ and 80.0 mg L^−1^ compared to the control. Moreover, HP-βCD⊃GA_3_ exhibits stronger effects than GA_3_ alone, though there was no significant difference between HP-βCD⊃GA3, βCD⊃GA_3_, and γCD⊃GA_3_ at certain concentrations. Similar results were observed in mung bean (*V. radiata*) growth, with HP-βCD⊃GA_3_ at 80.0 mg L^−1^ showing the highest growth promotion for both species. Root and seedling growth increases of up to 254.3% and 279.5%, with HP-βCD⊃GA_3_ outperforming other treatments.

**Fig. 50:**
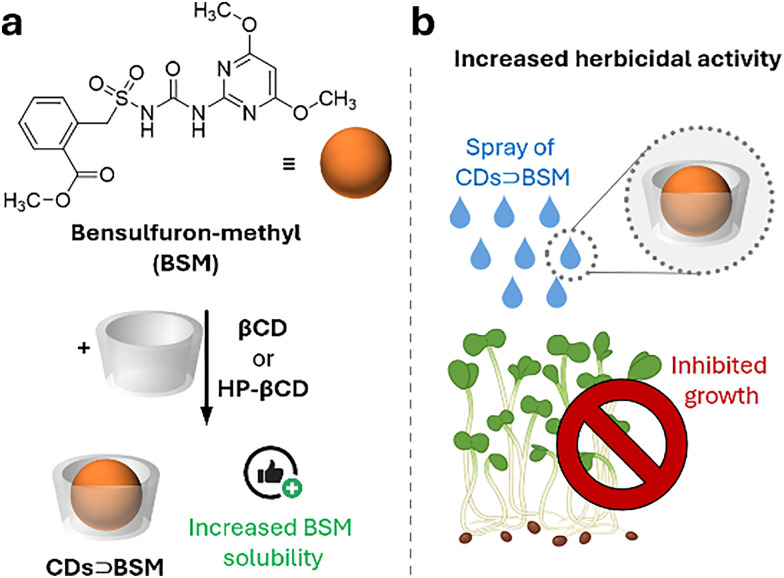
(a) The chemical structure of the herbicide bensulfuron-methyl forms inclusion complexes in water with βCD or (2-hydroxypropyl)-βCD, increasing its water solubility. (b) When applied to *Eclipta prostrata* by spraying onto the sprouts, the βCD-based formulations of BSM are more effective herbicides, as they inhibit growth more effectively than free BSM.

The inclusion complexes of the hydrophobic herbicide bensulfuronmethyl (BSM) with βCD and (2-hydroxypropyl)-βCD (2HP-βCD) were studied by Ni and co-workers (Fig. 50),^743^ who reported the formation of 1 : 1 complex for each case and moderate binding affinities of 316.6 M^−1^ (for βCD⊃BSM) and 277.6 M^−1^ (for 2HP-βCD⊃BSM) in deionised water (25 °C, pH 6.5) were determined by phase solubility experiments. The solubility of BSM in water (25 °C, pH 6.5) increases from 55.2 mg L^−1^ to 167 mg L^−1^ and 697 mg L^−1^, in βCD and 2HP-βCD solutions, respectively. Herbicide activities were tested on *Eclipta prostrata* in the greenhouse by spraying solutions of the hydrophobic herbicide and its more water-soluble βCD formulations on the plant. The percentage of inhibition for βCD⊃BSM and 2-HP-βCD⊃BSM at a dose of 5.63 g ha^−1^ after 28 days of treatment are found to be 42.3% and 50.4%, higher than that of pure BSM, respectively. When comparing the herbicidal efficacy of the two cyclodextrin-based formulations, 2HP-βCD⊃BSM proves to be more active.

**Fig. 51:**
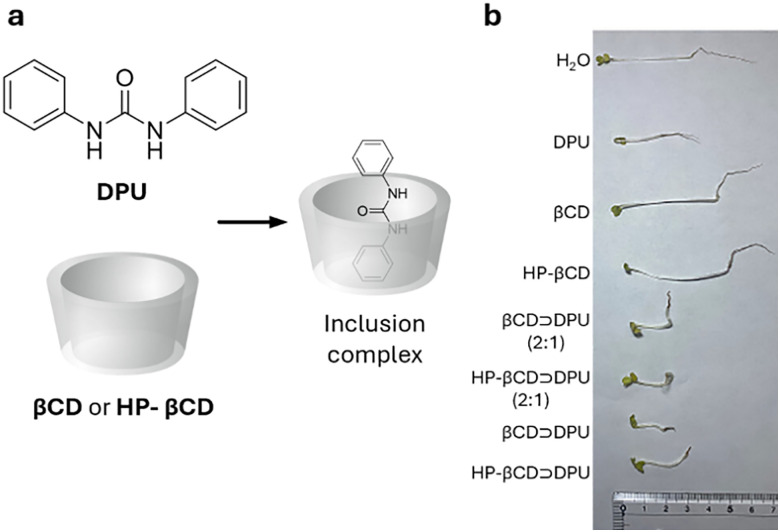
(a) The chemical structure of DPU forms an inclusion complex with βCD or HP-βCD in water. When applied to the growth medium for growing bean sprouts, the cyclodextrin formulations of DPU yielded thicker stems in the sprouts, indicating that the bioavailability of this cytokinin increased significantly. (b) Images of broccoli sprouts after 7 days of treatment. Figure adapted with permission from ref. 737.

Another example was reported by the Inoue group for enhanced delivery in promoting broccoli sprout growth (Fig. 51), wh ere 1,3-iphenylurea (DPU), a cytokine with poor water solubility, was incorporated into water-soluble βCD and hydroxypropyl-βCD (HP-βCD).^737^ DPU forms host–guest complexes with βCD (*K*_a_ = 250 M^−1^ for 1 : 1 complex) and HP-βCD (*K*_a_ = 427 M^−1^ for 1 : 1 and 196 M^−2^ for 2 : 1 complexes) in solution. Solid-state complexes prepared *via* ball mill grinding, confirmed by X-ray crystallography, revealed a mixture of βCD⊃DPU (2 : 1) and HP-βCD⊃DPU (2 : 1), with DPU solubility significantly increasing from 0.056 μg mL^−1^ to 7.03 μg mL^−1^. Broccoli sprouts treated with these complexes show increased stem thickness compared to controls, highlighting improved DPU bioavailability when delivered as a CD-based host–guest complex.

**Fig. 52:**
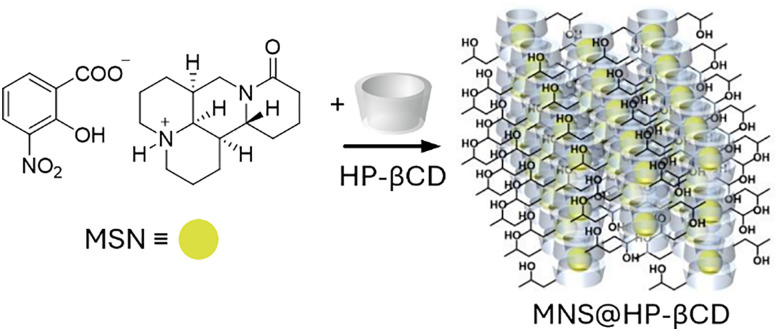
Chemical structure of MNS and schematic representation of its complex formation with HP-βCD. The presumed structure of the resulting host–guest complex and its assembly into nanometre-sized aggregates is also shown. Figure adapted with permission from ref. 752.

Rice is particularly vulnerable to damage from herbicides, such as pretilachlor (PRE). Owing to its non-selective mode of action, PRE severely impairs rice growth and yield. Despite its widespread use, no PRE-resistant rice varieties have been developed to date. To mitigate phytotoxic effects, safeners such as fenclorin (FEN) are employed to activate the plant's detoxification pathways. However, the limited efficacy of FEN necessitates high application rates, often at a 1 : 1 ratio with PRE, and its environment al persistence and toxicity to aquatic organisms raise serious ecological concerns. To address these limitations, Bai, Dang, and Zhong developed a novel PRE safener based on a matrine (MT) derivative modified with 3-nitrosalicylic acid, yielding the salt MNS (Fig. 52).^752^ This compound was further complexed with HP-βCD to produce the nanoformulation MNS@HP-βCD. Whereas unformulated MNS formed large micrometer-sized aggregates in aqueous media, encapsulation with HP-βCD reduced the particle size to 471 nm, a size range known to enhance membrane permeability and biological activity in plant tissues. The formulation was prepared *via* a cooling crystallization method, achieving a high loading efficiency of 80.2 wt%. While the precise binding mode and complex structure were not fully elucidated, encapsulation significantly improved the aqueous solubility of MNS to 278.5 g L^−1^, approximately 45.8 times greater than unformulated MNS and over 111 400 times higher than FEN. This enhanced solubility facilitated improved cellular uptake and more effective activation of the glutathione-mediated detoxification pathway, resulting in superior protection against PRE-induced phytotoxicity. Field trials demonstrated the enhanced efficacy of MNS@HPβCD over FEN, showing increases of 26% in seedling emergence, 15% in shoot height, 9% in root length, 27% in fresh weight, and 14% in overall yield, while retaining PRE's herbicidal effectiveness. Toxicological assessments further highlighted the advantages of the MNS-based formulation. MNS exhibited lower cytotoxicity than FEN, with IC_50_ values of 0.39 mg mL^−1^ (HepG2) and 0.38 mg mL^−1^ (HaCaT). MNS@HPβCD displayed even greater biocompatibility, with IC_50_ values of 9.09 mg mL^−1^ (HepG2) and 3.61 mg mL^−1^ (HaCaT). In contrast, FEN showed significantly higher toxicity, with IC_50_ values of 0.019 mg mL^−1^ and 0.02 mg mL^−1^ in the respective cell lines. These findings, further supported by zebrafish model data, underscore the improved biological and environmental safety of MNS-based nanoformulations, positioning them as a promising alternative for sustainable herbicide management in rice cultivation.

**Fig. 53:**
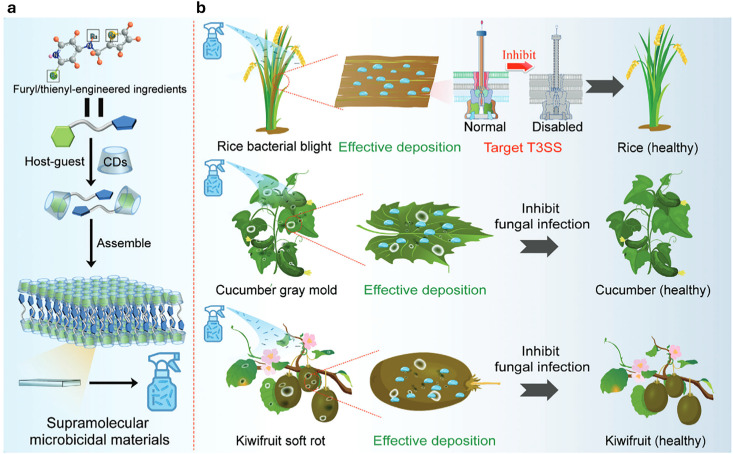
(a) Schematic representation of the formation of CD⊃F6-based supramolecular assemblies in water. (b) CD⊃F6-based supramolecular aggregates serve as T3SS inhibitors and are antifungal agents with excellent absorption properties, due to the presence of the cyclodextrin host through the leaves to control microbial infections in plants. Figure adapted with permission from ref. 739.

Wang and co-workers^739^ developed a supramolecular antimicrobial composite by combining newly synthesised type III secretion system (T3SS) inhibitors with βCDs to treat *Xanthomonas oryzae* pv. *oryzae* (Xoo) and fungi such as *Botrytis cinerea* and *Botryosphaeria dothidea* (Fig. 53). The design aimed to improve bioaccumulation in plants by enhancing leaf wetting and adhesion through dynamic interfacial tension changes, facilitated by cyclodextrins. The new T3SS inhibitors, based on furan or thiophene motifs, were synthesised *via* substitution reactions between furyl/thienyl-2-methylamines and substituted benzenes/pyridines. Among these, 2-chloro-*N*-(thiophen-2-ylmethyl)pyridin-4-amine (F6) show the highest antibacterial activity against Xoo (EC_50_ = 9.39 ± 0.1 μg mL^−1^). Complexation with βCD (*K*_a_ = 3.70 × 10^4^ M^−1^, as was determined by absorption and NMR spectroscopy) yielded nano-assembled structures (βCD⊃F6) with an average size of 465 nm, as was measured by DLS. Aqueous βCD⊃F6 formulations outperform pure F6 on rice leaves by improving wetting, reducing contact angles, and enhancing retention, leading to 48% protective activity *in vivo*, superior to the commercial bactericide thiodiazole-copper. In addition, toxicity studies show a lethal concentration 50 (LC_50_) of 12.5 μg mL^−1^ for βCD-F6. Then, antifungal screening identified 3-chloro-*N*-(furan-2-ylmethyl)-4-nitroaniline (F24) and 2-chloro-*N*1-(thiophen-2-ylmethyl)benzene-1,4-diamine (F25) as most effective against *B. cinerea* (EC_50_ = 4.10 ± 0.2 μg mL^−1^) and *B. dothidea* (EC_50_ = 3.10 ± 0.2 μg mL^−1^), respectively. Their complexes with HP-βCD and Me-βCD improve wetting, adhesion, and spreading on cucumber leaves and kiwifruit surfaces. *In vivo*, HP-βCD-F24, βCD-F24, and Me-βCD-F24 formulations show protective and curative efficiencies of 87.5% and 84.5%, 86.8% and 78.8%, and 90.1% and 77.5%, respectively, outperforming commercial pesticides such as pyrimethanil (59.9% and 62.4%) and azoxystrobin (71.1% and 65.3%), and the free FT24 (77.6% and 71.8%). Similarly, HP-βCD-F25, βCD-F25, and Me-βCD-F25 showed protective and curative activities of 86.8% and 70.8%, 76.8% and 62.5%, and 78.8% and 60.0%, respectively.

**Fig. 54:**
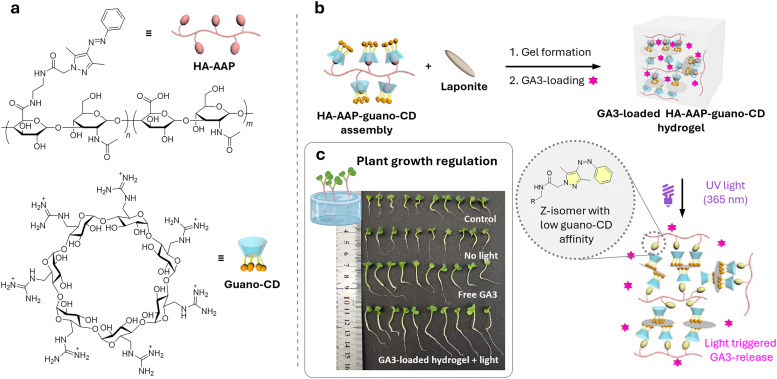
(a) Chemical structures of the arylazopyrazole-modified hyaluronic acid polymer (HA-AAP) and the guanidine-functionalised βCD (guano-CD). (b) Mixing HA-AAP with guano-CD leads to the formation of a supramolecular HA-AAP-guano-CD assembly, which, when mixed with LAPONITE® clays in water, forms a hydrogel through electrostatic attraction between the positively charged guanidine residues and the negatively charged clays. After drying the hydrogel, it can be loaded with GA3 by soaking the dried gel in a GA3-containing solution. Upon light irradiation, the arylazopyrazole moiety in HA-AAP switches from its *E* to *Z* isomer, which has a lower affinity for guano-CD, leading to hydrogel disassembly as the supramolecular crosslinking is disrupted. As the hydrogel degrades, GA3 is released and becomes bioavailable to plants. (c) Photographs of Chinese cabbage grown in media containing the GA3-loaded supramolecular hydrogel, with or without light irradiation. Figure adapted with permission from ref. 742.

Zhang, Sheng and co-workers^742^ reported a supramolecular hydrogel based on host–guest interactions between arylazopyrazole-modified hyaluronic acid (HA-AAP), guanidinium-functionalised βCD (guano-βCD) and LAPONITE® clay (LP, Fig. 54). This hydrogel facilitated the release of plant growth regulators, such as naphthalene acetic acid (NAA) and GA, as well as the uptake of heavy metal ions, *e.g.*, Cu^2+^. HA-AAP and guano-βCD form a positively charged supramolecular crosslinker, through host–guest interactions between βCD and the arylazopyrazole-moiety of HA-AAP (*K*_a_ = 1.18 × 10^3^ M^−1^), that interact electrostatically with negatively charged LP, leading to gelation. After drying and rehydration with a solution of plant growth regulators, these can be loaded into the porous gel network. Light irradiation (*λ*_ex_ = 365 nm) triggers a gel-to-sol transition by converting the arylazopyrazole in HA-AAP from its *E*- to its *Z*-isomer, weakening its binding to guano-βCD and releasing the plant growth hormone. At the same time, the now free carboxylic acids of the hyaluronic acid became accessible to bind Cu^2+^ ions. In experiments with Chinese cabbage and alfalfa seedlings, that were incubated together in Petri dishes with the hormone-loaded supramolecular hydrogel, enabled light-controlled plant growth by measuring features such as seed germination rate, stem length and seedling height, amongst others.

**Fig. 55:**
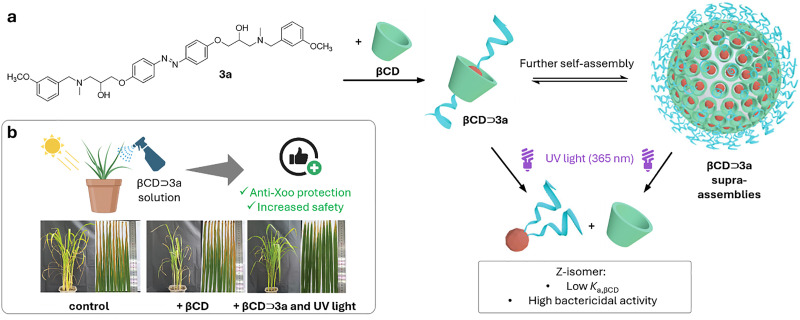
(a) Chemical structure of the antibacterial agent (*E*)-3,3′-((diazene-1,2-diylbis(4,1-phenylene))bis(oxy))bis(1-((3-methoxybenzyl)(methyl)amino)propan-2-ol) (3a). In its *E*-isomeric form, it binds to βCD, forming a βCD⊃3a host–guest inclusion complex, which in solution is hypothesised to form larger supramolecular aggregates. Upon light irradiation, the diazobenzene moiety within 3a switches to its *Z*-isomeric form, which has a lower affinity for βCD, resulting in the disassembly of the supramolecular complex. (b) Photographs of rice challenged with bacterial blight and subjected to βCD⊃3a, βCD, or the control (absence of 3a, βCD, or βCD⊃3a). Figure adapted with permission from ref. 746.

At this point, it is worth emphasising that the use of azobenzene as a light-activable photoswitch represents a prominent design feature to modulate host–guest interactions for the stimuli-responsive release of pesticides from porous nanoparticles.^742,753–756^ Therefore, in the context of pesticide delivery, Zhou, Yang and co-workers^746^ synthesised a series of double-headed azobenzenes with two isopropanolamine moieties on each benzene ring, which also serve as antibacterial agents, highlighting that the *Z* configurations of these molecules exhibit higher antibacterial activity than their *E* isomers. Of the synthesised azobenzenes, the derivative (*E*)-3,3′-((diazene-1,2-diylbis(4,1-phenylene))bis(oxy))bis(1-((3-methoxybenzyl)(methyl)amino)propane-2-ol) (compound 3a in Fig. 55) shows the highest antibacterial activity with EC_50_ = 0.52 μg mL^−1^ in both *trans* and *cis* form against *Xanthomonas oryzae* pv. *oryzae* (Xoo). The *E*-isomer showed an apparent binding constant with βCD of *K*_a_ = 2.90 × 10^4^ M^−1^ for the 1 : 1 complex (βCD⊃3a) in double distilled water with DMSO. Authors then tested the light-dependent degradation of the βCD⊃3a complex by switching from the *E*- to the lower βCD-binding *Z*-isomer, which becomes bioavailable in its non-complexed form, with higher pesticidal activity concerning the *E*-isomer. For *in vivo* experiments against rice bacterial blight, rice plants treated with 3a, and βCD⊃3a (at a concentration of 200 μg mL^−1^) were tested in an artificial climate chamber using a commercial UV light source, with another βCD⊃3a-treated plant placed in an outdoor greenhouse. Daylight is intense enough to convert 3a from its *E*- to the *Z*-isomer, resulting in protective and curative activities of 41.54% and 36.83% for free *E*-3a, 47% and 43, 34% for *Z*-3a, 51% and 48% for βCD⊃3a, and 56% and 52% using UV light, all of which outperform the commercial agents BT (35% and 32%) and TC (32% and 27%). The enhanced performance of the βCD-based complexes can be attributed to improved surface wettability, increased pesticide deposition, and better foliar uptake provided by this macrocycle.

Furthermore, Iacovino and co-workers^745^ showed the formation of inclusion complexes of βCD with chlorpropham (propan-2-yl (3-chlorophenyl)carbamate), monuron (3-(4-chlorophenyl)-1,1-dimethylurea) and propanil (*N*-(3,4-dichlorophenyl)propanamide), reporting binding constants for their 1 : 1 complex of 369.9 M^−1^, 292.3 M^−1^ and 298.3 M^−1^, respectively (determined in 1.00 mM phosphate buffer, pH 7.12).

Then, Basilio, Pina, and co-workers^741^ explored copigmentation and stabilisation effects of CB7 and (2-hydroxypropyl)-βCD (2-HP-βCD) on 7-hydroxyflavylium, pelargonidin, and cyanidin (anthocyanins). Particularly, anthocyanins are plant flavonoid pigments responsible for red, purple, blue, or black colours in fruits and flowers. Their colour is known to be affected by pH, solvent polarity, and aggregation, while they are sensitive to pH, temperature, and light, driving the search for stabilising systems. In their work the authors used UV-vis spectroscopy to show that CB7 complexation with 7-hydroxyflavylium (5 eq., 1.00 mM CB7, pH 1–7, 2–10% EtOH) shifts the p*K*_a_ from 2.3 to 4.8, stabilising the protonated form and enhancing colour intensity.^757^ Here, CB7 acts as a copigmentation factor,^758–760^ while βCD and 2-HP-βCD has the opposite effect, acting as anti-copigmentation agents. At near-neutral pH, CB7 modestly improves stability by reducing the hydration rate and acid–base equilibrium, while βCD increased the hydration rate, reducing stability. Despite some stabilisation, further modifications to macrocyclic systems are needed.

In addition, Saleh *et al.*^748^ investigated the stimulus-responsive release of imazalil (IMZ) from its complexes with CB8 and βCD, referred to as CB8-IMZ and βCD-IMZ. Cadaverine, a high-affinity binding agent for both macrocycles, was used as a stimulus to displace IMZ and activate its antifungal properties. Indeed, IMZ binds to βCD through its ethyl group (*K*_a_ = (5.30 ± 0.9) × 10^3^ M^−1^) and to CB8 at the aromatic site (*K*_a_ = (2.10 ± 0.9) × 10^6^ M^−1^), as shown by ^1^H-NMR. The antifungal activity of CB8-IMZ and βCD-IMZ was tested *in vitro* against several fungi: without cadaverine, their activity is lower than the one shown by free IMZ. However, in the presence of cadaverine, IMZ is released, enhancing antifungal activity. The results suggest a consistent release mechanism across different macrocycles and fungi. The authors attributed the difference to the results reported by Schirra *et al.*^749^ to the limited number of fungi tested in the earlier study.

**Fig. 56:**
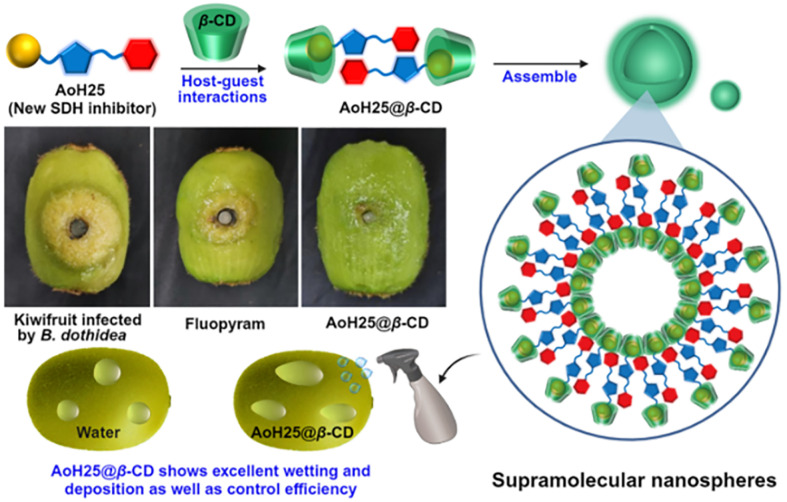
Schematic depiction of constructing fungicidal supramolecular nanovesicles (AoH25@βCD) to improve droplet wetting and deposition as well as efficiently inhibit fungal mitochondrial SDH. Figure reproduced with permission from ref. 761.

Recently, Wang and co-workers developed a βCD formulation with the novel SDH inhibitor AoH25, resulting in a host–guest complex (AoH25@βCD, Fig. 56).^761^ This complex self-assembled into biocompatible supramolecular nanovesicles in water, enhancing droplet-leaf (liquid–solid) interactions, improving wetting and retention on leaf surfaces, and thereby creating optimal conditions for increased fungicide efficacy. Mechanistic studies demonstrated that AoH25@βCD exhibited significantly higher SDH inhibition (half-maximal inhibitory concentration, IC_50_ = 1.56 μM) compared to fluopyram (IC_50_ = 244 μM) and AoH25 alone (IC_50_ = 2.29 μM). In addition, AoH25@βCD was found to increase the permeability of the *Botryosphaeria dothidea* cell membrane, thereby enabling more effective penetration of active compounds into pathogenic cells. Experimental data further demonstrate that AoH25@βCD achieves an 88.5% control rate against kiwifruit soft rot at a low concentration (100.0 μg mL^−1^), outperforming commercial fungicides such as fluopyram (52.4%) and azoxystrobin (65.4%). Furthermore, AoH25@βCD displays broad-spectrum bioactivity, reaching 87.2% efficacy against *Sclerotinia* in oilseed rape, again exceeding the performance of fluopyram (48.7%) and azoxystrobin (76.7%).

**Fig. 57:**
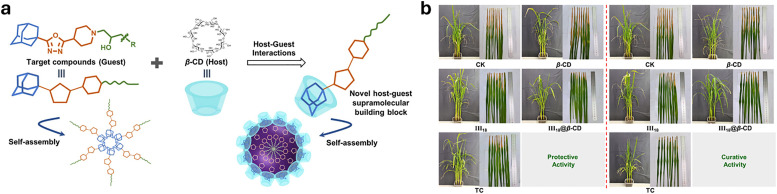
(a) Chemical structures of representative commercial adamantyl-based drugs, the molecular design strategy for adamantane-functionalised 1,3,4-oxadiazoles (guest molecules), and a schematic representation of spheroidal architectures formed through βCD-mediated host–guest interactions. (b) Protective and curative efficacies of compound III18, βCD, III18@βCD, and TC against rice bacterial blight at an effective III18 concentration of 200 μg mL^−1^. Figure adapted with permission from ref. 762.

The Wang group utilised a supramolecular spheroidal micelle constructed exploiting βCD–adamantane host–guest interactions,^762^ employing adamantane-functionalised 1,3,4-oxadiazoles as the βCD binding moiety, which also served as the primary pesticide against *Xanthomonas oryzae* pv. *oryzae* (Xoo), *Xanthomonas axonopodis* pv. *citri* (Xac), and *Pseudomonas syringae* pv. *actinidiae* (Psa, Fig. 57). Among the synthesised series of 1,3,4-oxadiazoles, the compound 1-(4-(5-((3*r*,5*r*,7*r*)-adamantan-1-yl)-1,3,4-oxadiazol-2-yl)piperidin-1-yl)-3-((3-chlorobenzyl)amino)propan-2-ol (III18) exhibits the highest antibacterial activity, with EC_50_ values of 0.94 μg mL^−1^ against Xoo, 0.89 μg mL^−1^ against Xac, and 3.3 μg mL^−1^ against Psa. Mixing III18 with βCD (*K*_a_ = 3.0 × 10^5^ M^−1^ for the presumed 1 : 1 complex in water containing 1% DMSO) leads to the formation of spheroidal nanoparticles with a relatively wide average size distribution, ranging from 1000 to 2100 nm, as was determined by TEM. This is attributed to the host–guest interactions between βCD and III18, which result in the formation of a supramolecular surfactant. In this complex, the hydrophobic adamantane moiety of III18 is encapsulated by the hydrophilic βCD, thereby promoting the self-assembly of the resulting species into the spheroidal structures in aqueous solution. The stimuli-responsive disassembly of these micelles was triggered by the addition of 1-adamantanamine (AD), a guest molecule with a higher binding affinity for βCD. This competitive displacement leads to micelle disruption, which was shown to enhance the antibacterial activity of III18 by increasing its bioavailability upon particle disassembly. This was confirmed through *in vitro* experiments, which showed that, in the absence of AD, the EC_50_ values against Xoo, Xac, and Psa were 3.95 ± 0.09 μg mL^−1^, 6.53 ± 0.23 μg mL^−1^, and 24.3 ± 0.1 μg mL^−1^, respectively. Then, upon AD addition, these values decreased to 1.04 ± 0.05 μg mL^−1^, 1.50 ± 0.28 μg mL^−1^, and 5.21 ± 0.10 μg mL^−1^, respectively. Although the stimuli-responsive behaviour was not tested *in vivo*, the particle formulation was effective against rice bacterial blight (with an effective dosage of III18 at 200 μg mL^−1^), achieving an infection elimination efficiency of approximately 43.6%. This result was slightly better than III18 alone (34.6–35.7% efficiency), and superior to the commercial bactericide thiadiazole copper (28.5–29.5%). Future studies on the evaluation of stimuli-responsive *in vivo* applications will be highly interesting, particularly in the presence of more suitable competitive binders to βCD than AD.

Li and colleagues reported large spherical microparticles (8 μm in diameter) of βCD polymer microspheres containing a light-switchable azobenzene loaded with PQ.^763^ Light irradiation triggered the *E*-to-*Z* isomerisation of the azobenzene, which weakens the βCD-azobenzene host–guest interaction, leading to the disintegration of the microparticles and the release of PQ (94.6% release efficiency after 8 h under UV-light exposure). This light-triggered release produces herbicidal activity against barnyard grass comparable to that of free PQ at the same dose and provided a safer delivery method in which the activity of PQ is only activated by the light-induced disruption of the microparticles.

A reactive oxygen-degradable micelle was constructed through a supramolecular host–guest interaction between a thioketal (TK) and adamantane (Ad)-bearing hydrophilic poly(ethylene glycol) monomethyl ether (mPEG) polymer, mPEG5000-TK-Ad, and ε-polycaprolactone (PCL) containing βCD.^764^ This interaction results in the formation of micelles (mPEG5000-TK-Ad@βCD-PCL), whereas this one-pot self-assembly approach also enables a relatively simple loading procedure for pesticides. The mPEG5000-TK-Ad@βCD-PCL have a size of 50 nm, as was determined by TEM. A ROS-responsive release of the pesticide from the interior of the micelle can be achieved as the thioketal moiety is chemically cleaved, enabling the fight against *Rhizoctonia solani* pest. For example, in the presence of H_2_O_2_ as a ROS source, the MBC-mPEG5000-TK-Ad@βCD-PCL achieved release rates of 62.7% (5.00 mg mL^−1^), 84.0% (15.0 mg mL^−1^), and 92.2% (30.0 mg mL^−1^). Overall, the micellar reformulation only performed marginally better than MBC alone; however, it represents an interesting new approach to achieving stimuli-responsive pesticide release.

**Fig. 58:**
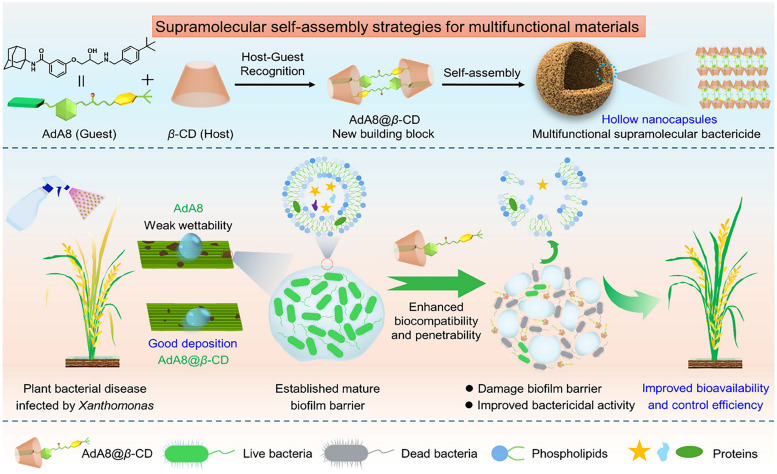
Chemical structure of AdA8, its supramolecular complexation with β-cyclodextrin, and schematic representation of the subsequent self-assembly into hollow nanoparticles. Also depicted is the spray-based application of the nanoformulation, which enhances leaf surface wettability, promotes effective biofilm disruption, and leads to improved overall bactericidal activity. Figure reproduced with permission from ref. 765.

An effective bactericide must overcome persistent biofilm barriers and achieve strong adhesion to leaf surfaces to ensure efficient bactericidal activity. To address these challenges, supramolecular self-assembly strategies have been employed to fabricate multifunctional aggregates. The Wang group designed a versatile supramolecular inclusion complex based on a novel amantadine-derived bactericide, AdA8 (Fig. 58).^765^ This molecule incorporates adamantane amide, isopropanolamine, and 4-*tert*-butylbenzylamine moieties and exhibits strong antibacterial activity, with EC_50_ values of 1.25 and 1.6 μg mL^−1^ against *Xanthomonas oryzae* pv. *oryzae* (Xoo) and *Xanthomonas axonopodis* pv. *citri* (Xac), respectively. AdA8 was subsequently complexed with βCD *via* host–guest interactions (*K*_a_ = 1.137 × 10^4^ M^−1^), in which the adamantane group preferentially resides within the hydrophobic cavity of βCD. This complex, referred to as AdA8@βCD, spontaneously assembles into hollow nanocapsules with an average diameter of 416 nm. The oligosaccharide-coated supramolecular assemblies facilitate rapid retention of the bactericidal agents on hydrophobic leaf surfaces and reduce droplet splashing and rebound. Additionally, the water solubility of the formulation is markedly enhanced. Importantly, AdA8@βCD exhibits superior biofilm-disrupting properties, significantly impairing bacterial motility and inhibiting the secretion of extracellular enzymes – key factors in bacterial propagation, colonization, and pathogenicity. At a concentration of just 5 μg mL^−1^, AdA8@βCD disrupts *Xanthomonas* biofilms by 78.3%. These multifunctional effects translate into improved *in vivo* performance, providing preventive efficacies of 51.1% and 73.2% against rice bacterial blight and citrus canker, respectively, at a concentration of 200 μg mL^−1^. These values outperform conventional agrochemicals such as thiodiazole-copper 20% (33.9% and 37.4%), kasugamycin (28.7%), and AdA8 alone (43.8% and 45.3%). Moreover, the AdA8@βCD formulation demonstrates good biosafety and improved environmental compatibility, highlighting the potential of oligosaccharide-coated supramolecular bactericides as eco-friendly alternatives in agricultural disease management. This work provides a valuable blueprint for the development of multifunctional green agrochemicals.

**Fig. 59:**
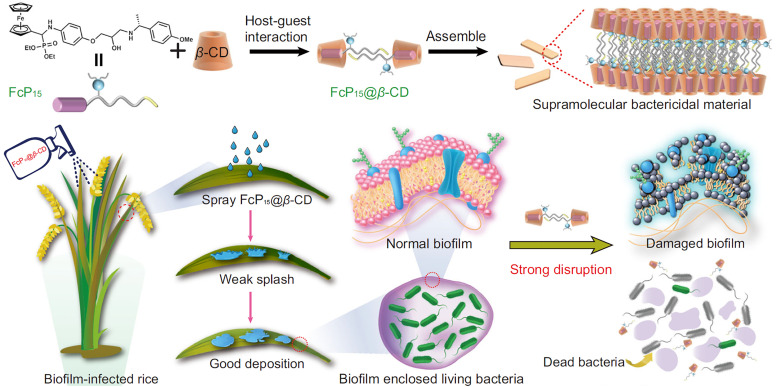
Schematic illustration of the fabrication of effective supramolecular bactericidal materials with enhanced bioavailability for controlling plant-associated biofilm infections. Reproduced with permission from ref. 766.

Wang, Du, and co-workers reported a supramolecular assembly between βCD and FcP15, a phosphate/isopropanolamine-modified ferrocene bactericide (Fig. 59).^766^ FcP15 binds within the βCD cavity (*K*_a_ = 1.6 × 10^4^ M^−1^), forming an amphiphilic complex (FcP15@βCD) that self-assembles in water into lamellar and micrometre-sized aggregates. Among several ferrocenyl analogues, FcP15 showed the best antibacterial activity (EC_50_ = 4.45 μg mL^−1^ against *Xanthomonas oryzae* pv. *oryzae*). The host–guest complex improved chemical stability of FXP15, with degradation rates reduced to ≤6.39% after 7 days, compared to ≥9.55% for the free guest. FcP15@β-CD also enhanced wettability and foliar deposition on rice leaves, boosting biofilm inhibition from 63.03% (FcP15) to 74.73% at 8.90 μg mL^−1^. Mechanistically, FcP15@β-CD disrupts biofilms by suppressing exopolysaccharides production (*gum* genes), motility (*flgB*, *motA*, *motB*), cell wall-degrading enzymes, and diffusible signal factor signaling (*rpf* genes). It consistently outperformed FcP15 in both *in vitro* and *in vivo* assays, showing higher efficacy against bacterial leaf blight (57.83%), bacterial leaf streak (53.18%), and citrus canker (79.75%) compared to commercial controls. Non-toxic to plants and zebrafish, FcP15@βCD offers a sustainable, solvent-free formulation with enhanced antibacterial activity, making it a strong candidate for environmentally friendly crop protection.

#### 3.2.2 Delivery examples using cucurbit[*n*]urils

**Fig. 60:**
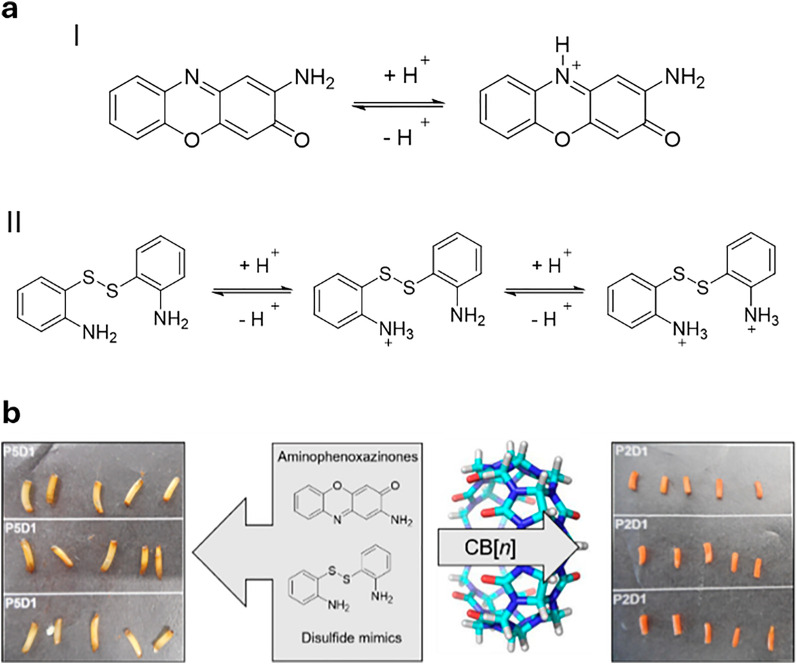
(a) Chemical structures of APO and DiS-NH_2_, along with their various protonation states. (b) Images of wheat coleoptiles after 24 hours of treatment with APO (left) or APO–CB7 (right). Figure adapted with permission from ref. 738.

At this point, it is worth emphasising that a well-known practical application of host–guest complex formation is that it can decrease the p*K*_b_ of amino compounds and render them protonatable at less acidic pH, thus enhancing their water solubility. This concept has been applied to the aminophenoxazinone 2-amino-3*H*-phenoxazin-3-one (APO) and its mimic, 2,2′-disulfanediyldianiline (DiS-NH_2_), compounds able to influence germination, growth, survival, and reproduction, but characterised by poor water solubility, limiting their agrochemical applications. Nau, Macías, and co-workers (Fig. 60)^738^ showed that their protonated forms, APOH^+^ and DiS-NH_3_^+^, formed 1 : 1 complexes with CB7, reporting *K*_a_ values of (1.80 ± 0.3) × 10^6^ M^−1^ for APOH^+^ and (3.91 ± 0.53) × 10^4^ M^−1^ for DiS-NH_3_^+^. In addition, the double protonated state of DiS-NH_3_^+^ has a higher binding affinity, *K*_a_ = (1.20 ± 0.4) × 10^5^ M^−1^ at pH 3.22. Upon complexation with CB7, APO's p*K*_a_ shifts from 2.9 to 4.1, and the one of DiS-NH_2_ from 2.1 to 3.2, improving both solubility and stability and allowing direct crop application without solution acidification, which is restricted by agricultural regulations. *In vitro* herbicidal tests using etiolated wheat coleoptile also showed that CB7 encapsulation lowers the IC_50_ values, thus enhancing bioactivity. At 300 μM, encapsulated APO at pH 4.6 shows 80% inhibition, while free APO had <10%. At pH 6.6, instead, the IC_50_ for APO⊃CB7 was 343 μM, with the free compound being inactive. Similar trends were observed for DiS-NH_2_: at 300 μM, elongation is reduced from 60% to 80% at lower pH (4.6 and 5.6), with more pronounced effects at 100 μM upon complexation.

Another important application has been found involving phytohormones of the auxins class, which are commonly involved in coordinating many plant growth processes. Particularly, synthetic auxins are used in agricultural practice to promote the rooting potential of cuttings or to prevent fruit drops in orchards. Thus, Nuzzo and co-workers^740^ proposed a CB7-based delivery system for the auxins IAA, 2-NAA, and 2,4-D, leveraging their pH-dependent complexation with this macrocyclic host. Inclusion complexes form exclusively with the protonated forms of these auxins, wherein the aromatic ring is preferentially encapsulated within the CB7 cavity, while the carboxyl group aligns with the host's carbonyl portals. At pH values exceeding the p*K*_a_ of the auxins, the guest molecules become negatively charged and are no longer retained by the macrocycle, thereby indicating the feasibility of pH-triggered release of auxin molecules from the CB7 host. Very recently,^744^ acyclic cucurbiturils, which have been first introduced by the group of L. Isaacs,^767^ have been shown to form association complexes with *R*-carvone (*K*_a,ACB1_ = (3.50 ± 0.1) × 10^4^ M^−1^, *K*_a,ACB2_ = (1.50 ± 0.1) × 10^5^ M^−1^) and l-limonene (*K*_a,ACB1_ = (2.50 ± 0.1) × 10^4^ M^−1^, *K*_a,ACB2_ = (1.32 ± 0.08) × 10^5^ M^−1^). The inclusion of these compounds also makes them more stable and enabled their temperature-dependent release behaviour. Although the temperature ranges investigated are not yet optimal for release applications in a real scenario, the increased stability and the potential for further adjustment of the release profile have been proven.

**Fig. 61:**
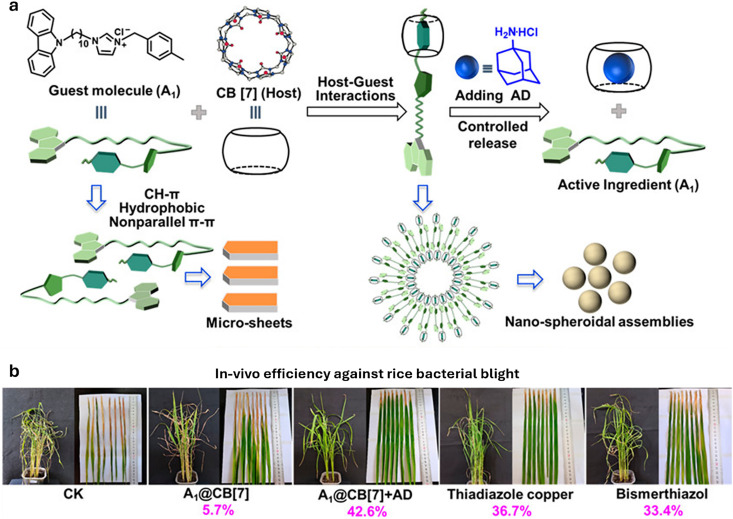
(a) Chemical structures of carbazole-functionalised QA salts (guest molecules) and a schematic representation of the stimuli-responsive host–guest supramolecular system employed for phytopathogen management. (b) *In vivo* trials against rice bacterial blight were conducted using CB7, AD, AD@CB7, and A1@CB7. BT and TC served as positive controls; CK as positive control. Figure adapted with permission from ref. 768.

It can be also emphasised that the ability of CB*n* to form host–guest interactions can be harnessed to create stimuli-responsive nanoparticles for pesticide delivery. Therefore, the Wang group developed a carbazole-decorated quaternary ammonium salt amphiphile with a cationic *N*-benzylimidazolium pendant (Fig. 61),^768^ which exhibits potent biological activity (EC_50_ = 0.647–0.892 μg mL^−1^ against *Xanthomonas oryzae* pv. *oryzae*). Among the synthesised salts, 1-(10-(9*H*-carbazol-9-yl)decyl)-3-(4-methylbenzyl)-1*H*-imidazolium chloride (A1) exhibits the highest activity (EC_50_ = 0.647 μg mL^−1^). An equimolar mixture of A1 and cucurbit[7]uril (CB7) in water initially forms a 1 : 1 host–guest complex (A1@CB7), which subsequently self-assembles into nanoparticles with an average diameter of 392 nm, as determined by DLS. The driving force for the assembly of this pesticide into spherical nanoparticles is attributed to the charge screening of the cationic *N*-benzylimidazolium moiety upon host–guest complexation with CB7. This interaction renders the complex more hydrophobic, thereby promoting self-assembly, most likely through a combination of hydrophobic effects and dispersion forces arising from π–π stacking interactions between A1@CB7 units. These nanoparticles disassemble upon adding a high-affinity CB7-binder, like adamantylamine, which competitively displaces the quaternary ammonium salts, leading to nanoparticle disassembly and the release of the pesticide. In pot experiments against rice bacterial blight, A1@CB7, triggered by the subsequent addition of adamantylamine, achieves a 42.6% control efficiency at 100 μg mL^−1^, surpassing commercial bactericides. Thus, this example illustrates very well the possibility of using the interactions between host and guest to develop innovative nanopesticides with stimulating behaviour. In the future, however, it will be important to find other ways to displace pesticides, in addition to the use of adamantylamine, a synthetic substance that is commonly used as a drug against influenza and to treat Parkinson's disease.

**Fig. 62:**
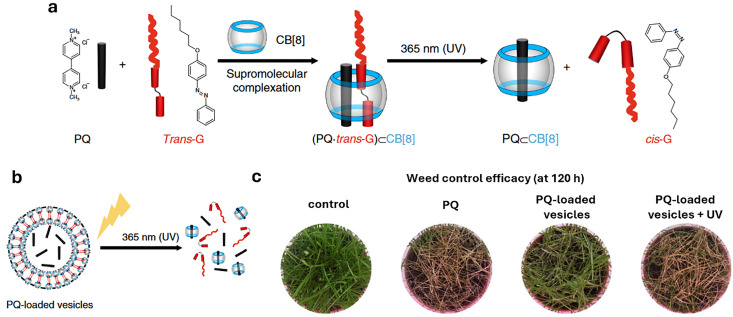
(a) Schematic representation of the complexation process involving CB8, PQ, and *trans*-G. The illustration depicts CB8-mediated binding with PQ as the primary guest and *trans*-G as the secondary guest. It also highlights the reversible, photo-induced transition between the complexation of the *trans*-G isomer and the decomplexation triggered by the *cis*-G isomer. (b) PQ-loaded vesicles release PQ upon light irradiation. (c) Weed control efficacy of free PQ and PQ-loaded photo-responsive vesicles. Foliar treatment was conducted using control (water), free PQ, and PQ-loaded vesicles under simulated sunlight irradiation, with an additional condition of PQ-loaded vesicles exposed to simulated sunlight without UV light, all at a single dose concentration of 2 mg mL^−1^. Figure adapted with permission from ref. 769.

In addition to the previously reported examples, to develop a light-controlled release system for paraquat, Wang and co-workers^769^ reported a paraquat-loaded supramolecular vesicle (Fig. 62) by the self-assembly of amphiphilic ternary host–guest complexes containing CB8, PQ, and an azobenzene-containing amphiphile, 1-[4-(hexyloxy)phenyl]-2-phenyl-diazene (*trans*-G), *i.e.*, (PQ·*trans*-G)⊂CB[8]. The stoichiometry of the adduct between *trans*-G and PQ⊂CB8 was 1 : 1, with an apparent binding constant *K*_a_ = (9.37 ± 2.37) × 10^4^ M^−1^, as measured by ITC in water. The resulting ternary complex (PQ·*trans*-G)⊂CB[8] is amphiphilic, with CB[8] acting as the hydrophilic head and the hexyl chain of *trans*-G as the hydrophobic tail. This asymmetry drives self-assembly into micelles or vesicles through supramolecular interactions. The loading capacity was 2.2% and the encapsulation efficiency was 16.4%. It was demonstrated that PQ·*trans*-G⊂CB[8] forms vesicles in water with an average diameter of 161.4 nm (as from TEM) and a wall thickness of ∼7 nm. The hydrodynamic diameter is 187.8 nm, and the vesicles show good colloidal stability over 210 days in PBS and DMEM buffers, even at different pH values (5.0, 5.8, 6.6, 7.4). Then, UV light irradiation (365 nm) led to *E*-to-*Z* isomerisation of the azobenzene amphiphile, weakening its binding to the macrocycle, resulting in vesicle disintegration and lowering PQ binding. Therefore, in the dark, PQ release is ∼10% within 10 hours, but under continuous UV irradiation, ∼90% of PQ was released within 24 minutes, whereas it took only ∼4 h to reach a cumulative PQ release ratio of 80% under natural sunlight (much less time than that under simulated sunlight). Moreover, the (PQ·*trans*-G)⊂CB[8] show effective herbicidal activity under natural sunlight, comparable to free PQ. When tested on the invasive grass species *Estuca arundinacea* (with a 2.00 mg mL^−1^ PQ dose sprayed on the grass and 120 h of natural light exposure), PQ is released within 3–4 hours, due to the apparent higher instability of the vesicle in a natural environment. In conclusion, the light-driven PQ-release properties under natural sunlight suggest that PQ-loaded vesicles hold strong potential for practical use in green agriculture, by enabling light-triggered sustained release and, as demonstrated in this study using cellular, Zebrafish, and mouse models, reducing PQ toxicity when present in the ternary complex.

**Fig. 63:**
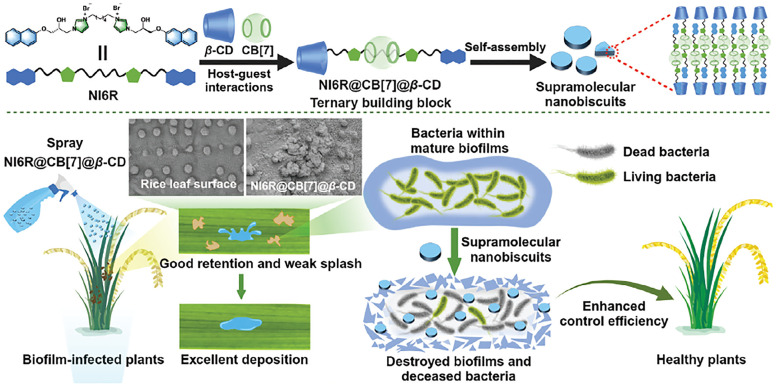
Schematic illustration depicts the assembly of a three-component supramolecular nanobiscuit system (composed of NI6R@CB7@βCD), engineered as a biosafe, multifunctional bactericidal material for improving foliar droplet deposition, eliminating persistent biofilms, and effectively controlling bacterial diseases. Figure adapted with permission from ref. 770.

He and coworkers^770^ developed a supramolecular self-assembled system composed of two macrocycles, CB[7] and βCD, along with an (*R*)-2-naphthol-based bis-imidazolium bromide salt (NI6R), which acts as a chain-like staple in the assembly. CB7 forms an inclusion complex with NI6R, yielding a rotaxane structure that further interacts with βCD *via* its naphthol pendants, creating a ternary building block (see Fig. 63). In aqueous media, these units self-assemble into disc-shaped aggregates, termed “nanobiscuits.” Spraying a solution of these nanobiscuits onto plant leaves significantly improved pesticide deposition on hydrophobic surfaces, reduced off-target droplet movement, and enhanced the inhibition and eradication of biofilm barriers, thereby mitigating bacterial virulence. Additionally, NI6R@CB7@βCD exhibited broad-spectrum bactericidal activity both *in vitro* and *in vivo*, surpassing conventional treatments in disrupting mature biofilms, inhibiting bacterial reproduction and motility, and reducing pathogenicity. Importantly, this supramolecular complex demonstrated high biosafety for crops and non-target organisms, including rice seeds, rice plants, zebrafish, and earthworms.

**Fig. 64:**
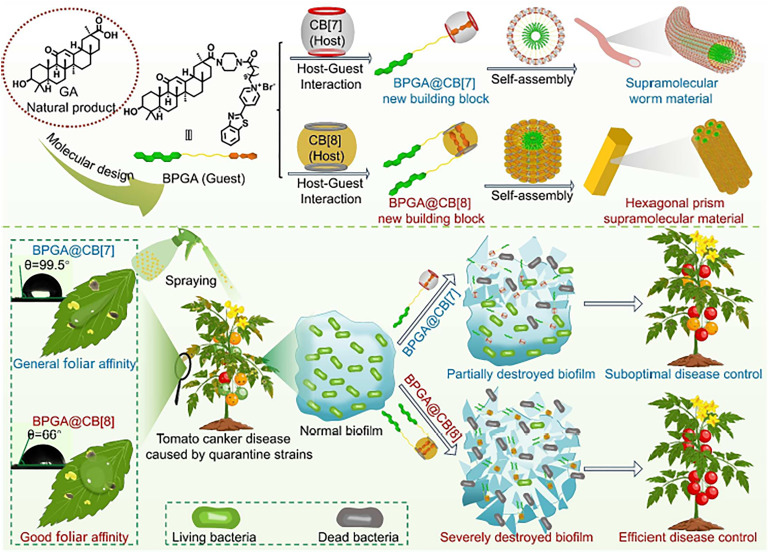
Schematic depiction of potent multifunctional supramolecular bactericidal materials derived from natural products as biofilm disintegrators with superior foliar affinity for the effective management of bacterial canker in tomato. Figure adapted with permission from ref. 771.

*Clavibacter michiganensis* (Cmm), a Gram-positive phytopathogen and A2 quarantine pest (EPPO), causes bacterial canker in tomatoes. Its dense biofilms shield bacteria from host immunity and block pesticides from their effective action. Furthermore, conventional treatments are inefficient, as splashing and bouncing disperse active ingredients away from target sites, necessitating novel antimicrobial strategies. A recent study^771^ introduced a hexagonal prism-shaped supramolecular material, BPGA@CB[8], formed *via* host–guest interactions between an 18β-glycyrrhetinic acid derivative (PBGA) and cucurbit[8]uril (CB[8]). This positively charged material disrupts biofilms, eliminates embedded bacteria, and enhances droplet retention on foliage (Fig. 64). BPGA@CB[8] demonstrated strong *in vitro* antibacterial activity and efficient deposition, translating into robust *in vivo* efficacy. At just 100 μg mL^−1^, it provided superior protective (56.9%) and curative (53.4%) effects against tomato bacterial canker.

#### 3.2.3 Delivery examples using calix[*n*]arenes

**Fig. 65:**
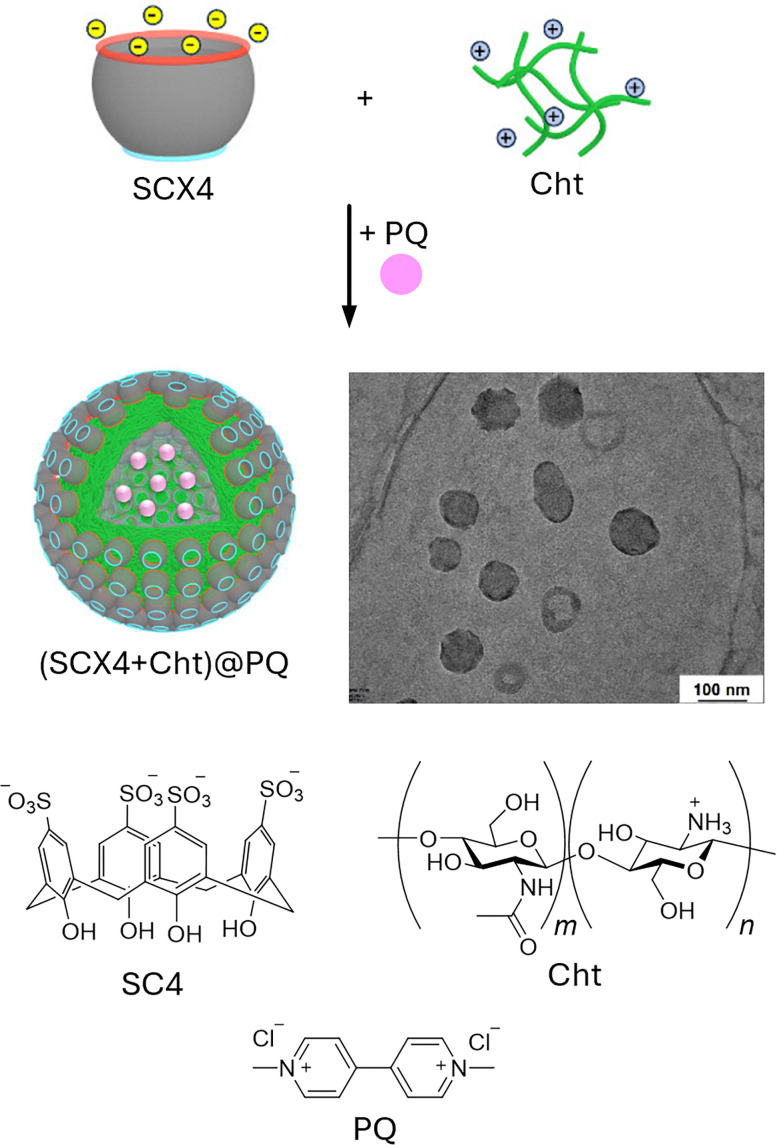
Schematic representation of the self-assembly of SCX4 with Cht to form nanometre-sized vesicles capable of loading paraquat (PQ). The resulting supramolecular formulation enhances foliar deposition and delivery efficiency of the PQ pesticide. Figure adapted with permission from ref. 772.

The Li group developed paraquat (PQ)-loaded nanovesicles using *para*-sulfonatocalix[4]arene (SCX4) and chitosan (Cht) *via* electrostatic self-assembly, forming hollow spherical nanoparticles (∼489 nm).^772^ The vesicles (Fig. 65) exhibit hydrophilic hydroxyl groups on their surfaces and encapsulate hydrophobic Cht chains through electrostatic interactions. They remain stable up to 65 °C and under high Na^+^ concentrations but disassemble at basic pH due to chitosan deprotonation. The formulation (SCX4 + Cht)@PQ showed a PQ loading of 3.74% and encapsulation efficiency of 50.70%. Improved wettability and lower contact angles enhanced foliar adhesion and pesticide uptake. In barnyard and setaria grasses, this led to greater herbicidal activity and reduced survival rates (15% and 22%, *vs.* 27% and 29% for PQ alone). Zebrafish toxicity assays confirmed higher biosafety, with >90% survival for the formulation *vs.* ∼50% for PQ. Compared to other smart delivery systems, such as light-, ROS-, or pH-responsive carriers, the (SCX4 + Cht)@PQ formulation is trigger-independent, easy to prepare, biocompatible, and highly effective on hydrophobic leaves, making it a promising tool for sustainable herbicide delivery.

In summary, the supramolecular reformulation of pesticides *via* host–guest complexation with macrocyclic hosts offers a powerful strategy to enhance efficacy, reduce toxicity, and improve environmental sustainability. Cyclodextrins have been the most widely used macrocycles due to their established biosafety and regulatory approval (*e.g.*, FDA). By encapsulating hydrophobic pesticides within their cavities, cyclodextrins increase apparent hydrophilicity, improve wettability, and protect active ingredients from degradation (*e.g.*, UV, pH, temperature). These effects collectively enhance foliar adhesion, promote plant uptake, and prolong pesticide residence time, enabling reduced application dosages and mitigating environmental contamination. However, the moderate binding affinities of cyclodextrins can limit their effectiveness in complex biological environments, where competing interactions reduce complex stability. To overcome this, alternative macrocycles such as cucurbiturils and pillararenes offer inherently higher affinities and structural tunability. Functionalized macrocycles further enable multi-stimuli-responsive and targeted release mechanisms. Importantly, such systems can also self-assemble into nanostructures with tuneable size, surface charge, and aggregation state, all of which influence transport, uptake, and bioavailability in plant systems. While most applications have focused on foliar delivery, future work should explore systemic delivery routes, including infiltration-based methods, to enable transport of agrochemicals or plant metabolites and real-time monitoring of plant responses. These advancements will require deeper insight into the fate, distribution, and biocompatibility of macrocyclic assemblies in planta, an area still in its infancy. Furthermore, extending these strategies to other agriculturally relevant compounds, such as nutrients, signalling molecules, or pheromones, could significantly broaden the scope of supramolecular agrochemistry. Despite their potential, many macrocycles beyond cyclodextrins face regulatory and societal barriers, particularly due to the nanoscale nature of their assemblies, which challenge standard formulation classifications. Moreover, the long-term environmental fate and potential bioaccumulation of modified macrocycles remain underexplored and demand rigorous toxicological evaluation. Addressing these scientific, regulatory, and ethical challenges will be critical for advancing macrocycle-based platforms in precision agriculture.

### 3.3 Delivery systems using nanoparticle scaffolds and supramolecular interactions

It should be noted that relying solely on macrocycle-based delivery systems is not the only option for developing improved products for crop protection, such as pesticides or nutrients. As previously highlighted in Section 1.5, nanomaterials are becoming increasingly attractive in plant science and agrochemical applications, as they can enhance the delivery and bioavailability of oligonucleotides, pesticides, and fertilizers, as well as improve soil properties to support better plant growth.^39,236–238^

In the following subchapter, we discuss nanomaterials that utilize supramolecular interactions to facilitate the transport and/or controlled release of plant-active substances, such as pesticides. Particular emphasis is placed on recent advancements in the delivery of pesticides, fertilizers, and oligonucleotides for gene therapy. Indeed, nanoparticles have been shown not only stabilise the nucleic acid cargo against degradation but also enable new strategies for targeted delivery to plant cells, including organelle-specific delivery.^242^

Although nanoparticles are already incorporated into commercial agrochemical formulations, primarily as passive carriers for active substances, there remains considerable potential to enhance their functionality. Next-generation nanocarriers can be engineered to enable stimuli-responsive release, targeted delivery, and enhanced uptake by plants. Furthermore, multifunctional nanoparticles can integrate delivery, imaging, and sensing capabilities within a single discrete entity. As will be discussed in subsequent chapters, nanoparticles can be functionalized with targeting ligands (*e.g.*, for chloroplast localization) and conjugated with additional moieties such as fluorescent dyes. In many cases, the intrinsic luminescent properties of nanomaterials can also be exploited for imaging applications. Furthermore, chemosensors may be attached to the particle surface to enable analyte detection, while the inherent porosity of certain nanoparticles can be utilized for cargo loading and controlled release. The porosity, which may range from microporous to mesoporous architectures, can be tailored to accommodate a broad spectrum of cargos, including small molecules and biopolymers such as proteins or nucleic acids. Indeed, the delivery of nucleic acids is a particularly promising application of nanomaterials in plant sciences, as will be highlighted in Section 3.3.2. Also, the morphology of the nanomaterials can be optimized to modulate both loading capacity and distribution within plant tissues.

Nucleic acids play a significant role in the development of next-generation pesticides, exemplified by the 2023 EPA approval of Calantha,^773^ the first sprayable RNA-based pesticide developed by GreenLight Biosciences, for commercial use. This new pesticide targets the Colorado potato beetle by disrupting gene expression in the eggs and adult beetles, leading to their death. While it is unclear what RNA-transfection agent Calantha contains, research into effective delivery systems, *e.g.*, nanoparticles, able to protect RNA from degradation and improve uptake by plants, remains crucial. Nevertheless, nanoparticle-based carriers show promise in delivering the RNA cargo to cells in an organelle-specific manner and offer a potential solution for more efficient RNA-based pest control strategies,^774^ as will be discussed in later sections. However, despite their potential benefits, many questions remain regarding the environmental fate of nanomaterials, which is critical for assessing their bioavailability and long term impact.^216,249,250^

#### 3.3.1 Delivery of small organic molecules with nanoparticles

##### 3.3.1.1 Mesoporous silica-based nanocarriers

**Fig. 66:**
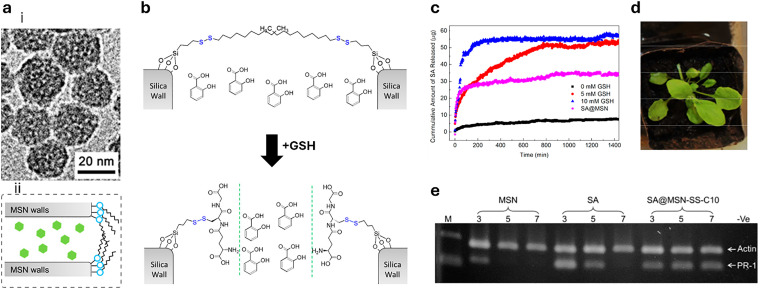
(a) Transmission electron microscopy (TEM) image of MSPs used to construct gated and SA-loaded mesoporous silica particles. (b) The presence of GSH enables the gatekeeper to open through a disulfide exchange reaction. Once the gatekeeper unit (C10-aliphatic chain) is removed, the salicylic acid is free to diffuse out from the nanoparticle's pore. (c) The cumulative amount of SA released from MSN-SS-C10 under different GSH concentrations. (d) Representative photos of *Arabidopsis thaliana* seedlings in pots after salicylic acid-loaded MSN-SS-C10 nanoparticle treatment at day 7. (e) “Housekeeping” gene actin and defence gene PR-1 expression in *Arabidopsis thaliana* following MSN, SA, and SA@MSN-SS-C10 treatment on days 3, 5, and 7. M represents HyperLadder IV (bioline), and −Ve represents the blank channel. Actin is in the top row, and PR-1 is in the bottom row. Figure adapted with permission from ref. 775.

In 2015, Cahill, Kong, and co-workers^775^ reported the use of MSPs with redox-cleavable gatekeepers at their pore entrances, formed by intertwined alkyl chains which interact through supramolecular dispersion forces, for delivering salicylic acid (SA) salic to plants (Fig. 66). In this example, 20.0 nm-sized MSPs were loaded with SA at a loading efficiency of 19.0 μg mg^−1^, and their pores were capped by functionalizing the particle surface with a dodecyl disulfide capping group (MSN-SS-C10). While the mesopores remain blocked at GSH concentration of 5.0 mM, as indicated by the low release rate of SA, the gatekeepers are removed at a higher GSH concentration (10.0 mM) *via* a disulfide exchange reaction between GSH and the disulfide moieties of the gatekeeper alkyl chains, thereby enabling the release of SA from the MSP pores. This redox-responsive opening of the gatekeepers enables the release kinetics to be tuned, ranging from 0 to 800 minutes in water. The controlled release of SA in *Arabidopsis thaliana* was evaluated by monitoring the expression of PR-1, an SA-responsive marker gene, and assessing GSH accumulation levels following nanoparticle delivery *via* vacuum infiltration. PR-1 expression can be detected across all the days tested in which the plants were treated with SA-loaded MSN-SS-C10 (0–7 days), as the effect of the constant supply of SA released from the pores of the MSNs.

**Fig. 67:**
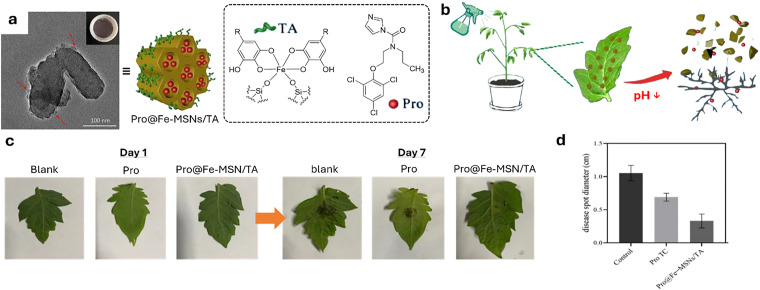
(a) TEM image of Pro@Fe-MSNs/TA. (b) The antifungal activity was tested *in vivo* using three-week-old tomato leaves, which were sprayed with Pro@Fe-MSNs/TA and Pro–TC at a Pro concentration of 1 μg mL^−1^. *Rhizoctonia solani*, a fungus, secretes organic acids during growth and infection that acidify plant tissues, creating favourable conditions for its reproduction. Simultaneously, the disintegration of the Fe–O coordination bond within the MSPs leads to their disintegration. (c) Images of tomato leaves treated with deionised water (blank), Pro, or Pro@Fe-MSNs/TA in fungicidal activity tests and (d) lesion diameters measured at 7 days after the fungi inoculation. Figure adapted with permission from ref. 776.

Later, Zhang and co-workers^776^ developed a pH-responsive, iron-doped mesoporous silica nanoparticle for targeted prochloraz (Pro) release, triggered by pH changes (Fig. 67). Prochloraz loading can be achieved through supramolecular interactions, *i.e.*, the self-assembly of the hydrophobic pesticide within the mesopores of the particles, reaching a loading capacity of 31 wt%. The Fe^3+^ ions embedded in the pore walls facilitate the efficient coating of the particles with tannic acid (TA), which serves to block Pro within the pores and forms a pH-responsive shell that dissolves under acidic conditions, particularly in environments where *Rhizoctonia solani* (*R. solani*) thrives. The resulting Pro@Fe-MSNs/TA nanoparticles, with an elliptical shape and a size of 471 ± 3.9 nm (as from DLS), show a pH-dependent and sustained Pro release. Approximately 63.8% of Pro is released within the first 24 hours at pH 4.0, with a continued slow release up to 72 hours. Authors observed that the TA coating initially inhibits Pro release but, under acidic conditions, the coordination bonds between TA and Fe^3+^ and the pesticide brake due to competitive binding with H^+^, leading to a boosted release. Thus, the IC_50_ value of Pro@Fe-MSN/TA nanoparticles against *R. solani* is 0.24 ± 0.02 mg L^−1^, 16.7% lower than that of the Pro–TC control (0.28 ± 0.04 mg L^−1^). In tomato leaf tests, Pro@Fe-MSNs/TA exhibits significantly greater antifungal activity than Pro alone, reducing the leaf rotten area diameter to 0.33 ± 0.11 cm, compared to 1.05 ± 0.12 cm for the negative control and 0.69 ± 0.06 cm for Pro–TC treatment.

**Fig. 68:**
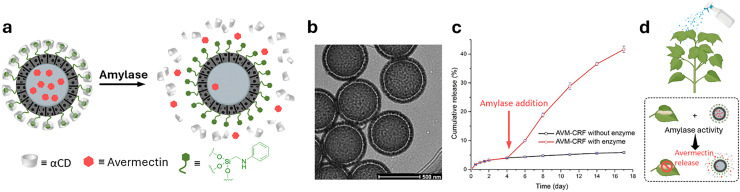
(a) Scheme for the amylase-triggered release of AVM from AVM-HMS9 by the degradation of αCD caps. (b) TEM image of αCD capped HMS. (c) Cumulative AVM release profiles from AVM-CRF in the presence (red line) and absence (black line) of amylase. Figure adapted with permission from ref. 777.

Another example has been reported by He and co-workers,^777^ involving the use of amylase enzyme activity to trigger the release of the insecticide avermectin (AVM) against *Plutella xylostella* from cyclodextrin-capped hollow mesoporous silica nanoparticles (HMS). Briefly, HMS were loaded with avermectin (AVM-HMS, Fig. 68) with an efficiency of 38 wt% using an impregnation method. Subsequently, the entrances of the mesopores were capped by binding βCD to phenylamine pendants that had been covalently attached to the HMS surface, thereby forming a supramolecular gatekeeper *via* host–guest complexation. Later, plant leaves were sprayed with the nanoparticle formulation and given to the insects as food. AVM-HMS at a dosage of 0.60 mg L^−1^ shows toxicological activity against *Plutella xylostella* larvae whereas the αCD caps are cleaved *in vivo* by the enzymes released from the parasite, releasing AVM from the HMS pores and causing larval death. A mortality rate of 83% can be achieved with this stimulatory delivery system, 40% higher than what is obtained with the commercial AVM formulation.

##### 3.3.1.2 Quantum dots-based nanocarriers

**Fig. 69:**
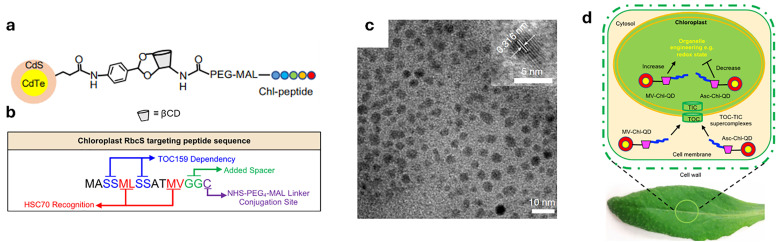
(a) Scheme of chloroplast targeting quantum dots (Chl-QDs) containing βCD and chloroplast targeting peptide (Chl) that is based on a (b) truncated Rubisco small subunit biorecognition motif (RbcS), which guides protein precursors to chloroplast outer membranes. (c) TEM image of QDs lacking the targeting peptide. (d) Quantum dots coated with a chloroplast guiding peptide (in blue) and a β-CD molecular basket (in magenta) enable loading of methyl viologen (MV-Chl-QD) or ascorbic acid (Asc-Chl-QD) and targeted modification of the redox status of chloroplasts in planta. The RbcS targeting peptide is designed to bind to the translocon supercomplex on the chloroplast outer membrane (TOC). Figure adapted with permission from ref. 778.

In 2020, Giraldo and co-workers^778^ reported on βCD-decorated QDs as intrinsically fluorescent nanomaterials to efficiently deliver small molecules to chloroplasts in wild-type *Arabidopsis thaliana* plants (Fig. 69). Here, the βCD molecules were covalently attached to *p*-aminophenylboronic acid-capped QDs (4.30 ± 0.2 nm, core size by TEM) *via* boronic ester formation with mono-(6-ethanediamine-6-deoxy)-βCD (cavcon-βCD). Chloroplast targeting is achieved by functionalising the cavcon-βCD QDs with the NHS-PEG_4_-MA linker, forming an amide bond, and then conjugating the Rubisco small subunit 1A peptide (RbcS; sequence: MASSMLSSATMVGGC), linking the terminal cysteine to maleimide-functionalised QDs. These βCD and peptide-functionalised Chl-QDs colocalise with chloroplasts in *Arabidopsis* plants treated *in vivo* (500 nM), as confirmed by confocal microscopy. Additionally, since βCD can form host–guest inclusion complexes with redox-active or bioactive plant compounds such as methyl viologen (MV^2+^, *K*_d_ = 4.76 × 10^−5^ M, loading capacity 85%) or ascorbic acid (ASC, *K*_d_ = 3.98 × 10^−5^ M, loading capacity 96%), these complexes were shown to facilitate delivery to *Arabidopsis thaliana* mesophyll cells *in vitro*. Notably, the delivery of MV-Chl-QDs to chloroplasts was enhanced twofold compared to the control (MV^2+^ alone).

**Fig. 70:**
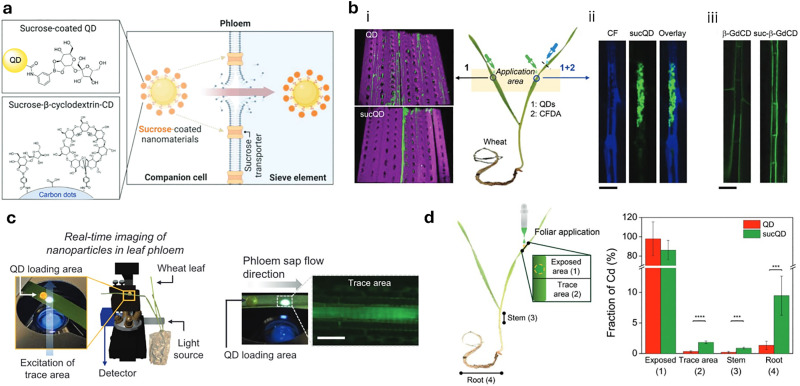
(a) Sucrose-coated QDs (sucQDs) and βCD-carbon dots (suc-β-CDs) are delivered to the phloem *via* foliar application. These nanomaterials are guided through leaf tissues by binding to sucrose transporters in phloem vessels, bypassing cell barriers and penetrating phloem cells by disrupting lipid membranes. (b) (i) 3D confocal microscopy images of leaves near the QD or sucQD foliar application area in intact live plants show that sucQD (in green) was localised in wheat parallel leaf veins between mesophyll cells containing chloroplasts (in magenta). (ii) Representative images showing the high colocalisation of sucQD with carboxyfluorescein (CF) fluorescent dye that labels phloem cells (in blue). Scale bar = 30 μm. (iii) In planta confocal fluorescence microscopy images of β-GdCDs and suc-β-GdCDs in wheat leaf vasculature. The suc-β-GdCD were localised in the vasculature 2.2 times higher than the uncoated GdCD. Scale bar = 30 μm. (c) Real-time imaging of QDs within the phloem of wheat leaves in planta using a customised inverted epifluorescence microscope. Scale bar = 100 μm. (d) The uptake and translocation of QDs and sucQD to various wheat plant organs were analyzed using ICP-MS (targeting the Cd element in the QD core). Shown are the sampled areas, including exposed and trace leaf regions, stems, and roots. After 24 hours of nanoparticle exposure, the fraction of Cd detected in wheat plants reveals significantly greater translocation of sucQD to all sampled areas, including roots, compared to unmodified QDs. Figure adapted with permission from ref. 779.

Recently, the same group also developed some sucrose-coated carbon quantum dots functionalised with βCDs (sucrose-βCD),^779^ which have a size of 9.10 ± 2.8 nm (as from TEM) and 20.3 ± 3.6 nm (as from DLS in TES buffer). These quantum dots (Fig. 70) enabled more efficient delivery of bioactive molecules to plant cells, once again by exploiting the effective host–guest complexation ability of βCD to encapsulate and transport potentially bioactive compounds into plants. Indeed, the study showed that delivery of sucrose-coated QDs through the leaf increased targeted transport to the phloem and improved long-distance translocation in wheat (*Triticum aestivum*). In addition, phloem loading results in 6.8 times more transport to the roots compared to unmodified QDs, with about 70% reaching the roots. Probably, sucrose coating aid membrane penetration either by temporarily disrupting the lipid bilayer or by endocytosis. Notably, these nanoparticles show excellent biocompatibility with negligible cytotoxicity in leaf mesophyll cells after 24 hours of exposure. While the delivery of Rh6G *via* βCD mediated host–guest interaction has been demonstrated, future research could still explore the transport of more biologically relevant substances or the use of higher affinity binders, such as CB*n*, to expand the range of deliverable molecules, particularly for those with low βCD affinity.

##### 3.3.1.3 Plant virus-derived nanocarriers

**Fig. 71:**
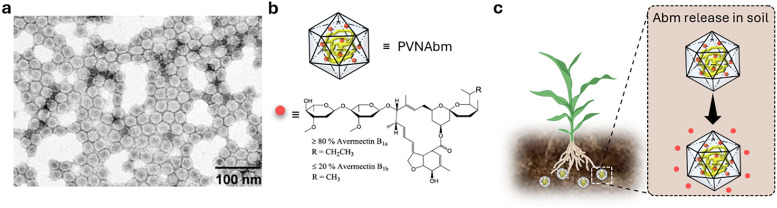
(a) TEM image of RCNMV loaded with Abm (PVNAbm). (b) Schematic representation of PVNAbn and the chemical structures of Abamectins. (c) PVNAbm enhances the soil mobility and controlled release of Abm, resulting in an expanded zone of protection against *Meloidogyne* hapla root-knot nematodes. Figure adapted with permission from ref. 780.

Recently, plant-derived virus-like nanoparticles (PVNs) have been explored for managing nematode infestations in the rhizosphere. In particular, Willoughby and co-workers^780^ used PVNs derived from the red clover necrotic mosaic virus (RCNMV, *D* = 36 nm) to load the nematicide abamectinabamectin (Abm), forming Abm-loaded PVNs (PVNAbm). These PVNs (Fig. 71) can be loaded under low Ca^2+^ and Mg^2+^ concentrations (nM levels), opening 90 pores (11–13 Å) on the capsid surface, and being closed by high cation concentrations (mM levels). Plant viruses are of particular interest due to their dynamic ability to self-assemble into well-defined, uniformly sized nanomaterials *via* supramolecular interactions, such as electrostatic forces. In the present case, electrostatic interactions between cationic species and viral proteins govern the formation of two distinct structural variants: a large-pore and a small-pore form. While the small-pore variant restricts the diffusion of cargo into or out of the protein nanocage, the large-pore form facilitates such molecular exchange. In addition, PVNAbm addressed the issue of Abm's limited soil mobility, enabling controlled release and enhancing bioavailability to nematodes during application. For example, PVNAbm showed equivalent bioavailability to free Abm against *C. elegans* in liquid culture, but offered improved soil mobility, as evidenced by its clearance through a soil column. This enhanced mobility leads to superior crop protection against root-knot nematodes (RKN), compared to the same dose of free Abm.

**Fig. 72:**
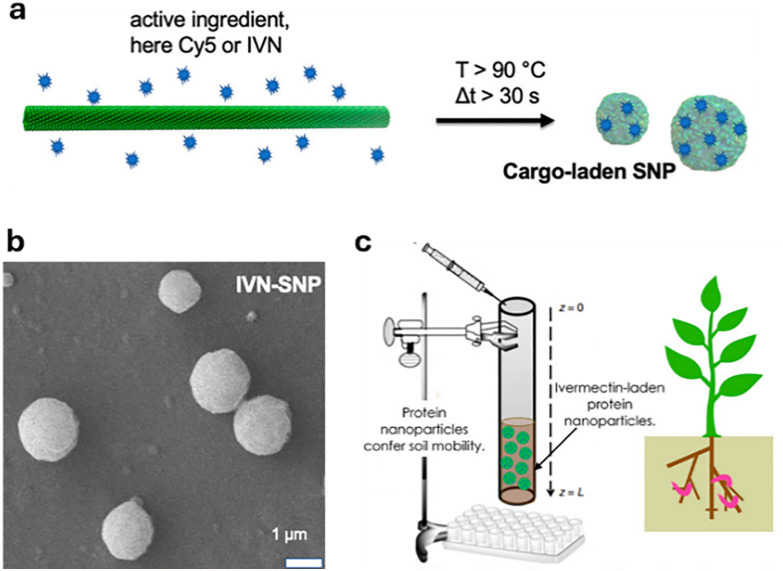
(a) Encapsulation of small molecules (such as the active ingredient, Cy5, or IVN occurs during the thermal shape transition of TMGMV into SNPs, with transparency indicating the incorporation of the small molecules within the SNPs). (b) SEM image of IVN-loaded TMGMV nanoparticles. (c) IVN-loaded nanoparticles have improved mobility and slightly higher soil retention compared to TMGMV rods. Ivermectin delivery to *Caenorhabditis elegans* was confirmed after the SNP formulations passed through the soil. Figure adapted with permission from ref. 781.

In another recent work, Steinmetz and colleagues^781^ utilised nanoparticles from tobacco mild green mosaic virus (TMGMV) for the delivery of ivermectin (IVN, Fig. 72). The virus capsids undergo a transformation from rod-like structures into spherical nanoparticles upon heating, due to the dynamic nature of supramolecular interactions – a process that was exploited to load the spherical particles with ivermectin. With this approach, a high loading efficiency of 60 wt% IVN can be achieved. The resulting nanoparticles, ranging in size from 100 nm to 2 μm, show increased soil mobility compared to free IVN and efficacy against *Caenorhabditis elegans*, with a two-fold reduction in nematode mobility at doses of 5.00 and 10.0 mg mL^−1^.

##### 3.3.1.4 Metal–organic framework-based nanocarriers

**Fig. 73:**
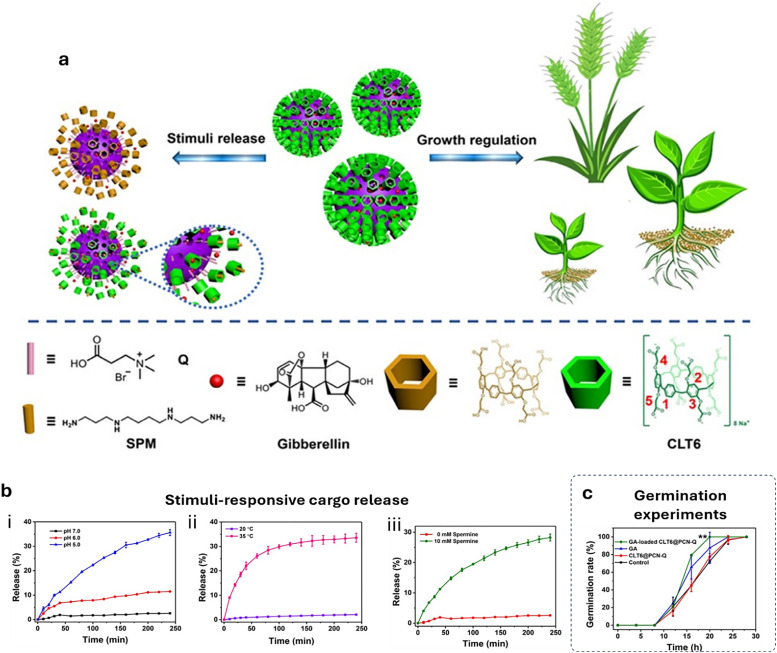
(a) Schematic representation of the fabrication process for the multi-stimuli-responsive supramolecular nano platform (GA-loaded CLT6@PCN-Q), utilising a CLT6-capped MOF and their use as plant growth regulators. (b) The release profiles of RhB from RhB-loaded MOF-based nanoparticles in response to external stimuli, such as (i) pH, (ii) temperature, and (iii) SPM. (c) Germination curves of wheat treated by CLT6@PCN-Q, GA, and GA-loaded CLT6@PCN-Q. Figure adapted with permission from ref. 782.

For the controlled release of GA in response to temperature changes, pH changes or in the presence of biomolecules such as spermine (SPM), a supramolecular MOF-based nano platform with a size of 100 nm was developed (Fig. 73a), able to respond to various stimuli.^782^ The porous MOFs can be synthesised following an already established methodology, using 5,10,15,20-tetrakis(4-carboxyphenyl)porphyrin as an organic linker and zirconyl chloride octahydrate as a metal node. Then, the stimuli-responsive release system was achieved by covalently functionalising the MOF nanoparticle surface with a quaternary ammonium linker, which can bind to the desymmetrised pillar[6]arene (CLT6)-type macrocycle, *i.e.*, tower[6]arene,^783^ which ultimately leads to the formation of CLT6@PCN-Q.^784^ This macrocycle serves essentially as the supramolecular and removable pore-closing component by forming the host–guest complex with the ammonium salt of the nanoparticle. Loading of the MOF with GA was achieved by an impregnation method, resulting in an overall loading efficiency of 26 wt% (in GA-loaded CLT6@PCN-Q). Then, charge release was triggered by increasing the temperature (from 25 to 3 °C), protonating the carboxylate groups of CLT6 (at pH 5–6), or by displacement of CLT6 by SPM, which binds more strongly to the macrocyclic stopper than the quaternary ammonium compound of the nanosized MOFs, as shown for rhodamine-loaded particles in Fig. 73b. In addition, placing plant seedlings of Chinese cabbage or monocotyledonous wheat in Petri dishes containing GA-loaded nanometre-sized MOFs (equivalent to 20 mg L^−1^ free GA) effectively promote the germination of wheat seeds and the stem growth of both dicotyledonous Chinese cabbage and monocotyledonous wheat.

##### 3.3.1.5 Polymer-based nanocarriers

**Fig. 74:**
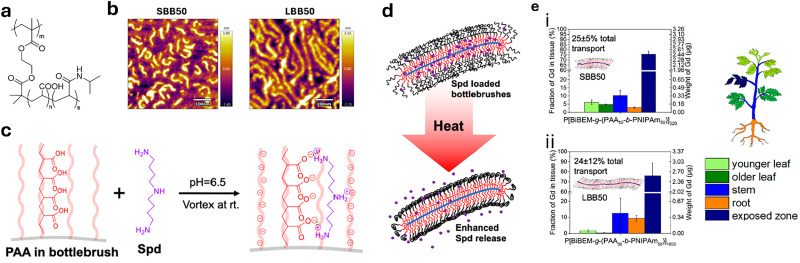
(a) General chemical structure of P[BiBEM-*g*-(PAA-*b*-PNIPAm)] polymer bottlebrushes. (b) Atomic force microscope height images of SBB50 and LBB50. (c) Schematic showing the spermidine (Spd) loading into the polymer bottlebrushes and (d) high temperature-induced Spd release. (e) Uptake and transport of Gd^3+^-loaded bottlebrushes in tomato plants after foliar application of 20 μL of a 1 g L^−1^ suspension in 0.1 v/v% Silwet L-77 for (i) SBB50 and (ii) LBB50. Amounts of Gd detected in the different plant tissues are expressed by both the fraction of Gd mass applied and total Gd mass in each plant compartment (number of experiments per sample = 5). Figure reproduced with permission from ref. 785.

Eventually, temperature-responsive bottle-brush polymer brush blocks,^785^ poly[2-(2-bromoisobutyryloxy)ethyl methacrylate-*g*-poly(acrylic acid)-*block*-poly(*N*-isopropylacrylamide)] (P[BiBEM-*g*-(PAA-*b*-PNIPAm)]), were designed for electrostatic complexation, transport, and release of spermidine (Spd), a plant stress regulator (Fig. 74). These worm-like bottle-brush polymers included: P[BiBEM-*g*-(PAA50-*b*-PNIPAm50)]320 (SBB50), P[BiBEM-*g*-(PAA50-*b*-PNIPAm150)]320 (SBB150), P[BiBEM-*g*-(PAA50-*b*-PNIPAm50)]1600 (LBB50) and P[BiBEM-*g*-(PAA50-*b*-PNIPAm150)]1600 (LBB150). AFM showed lengths of 80 nm for SBB50 and ∼300 nm for LBB50, and the hydrodynamic diameters were proportional to the length of the backbone, with SBB50 and SBB150 at ∼40 nm, and LBB50 and LBB150 at ∼100 nm. The loading efficiency varied from 5500 ± 910 to 7300 ± 1400 Spd molecules per polymer. In addition, these polymers enable temperature-dependent Spd release and thus can alleviate heat stress in plants for 15 days after foliar application to tomato leaves (SBB50 at 0.5 g L^−1^, applied as five 10 μl drops). Indeed, the particles enter the phloem and then can release Spd in the presence of heat. Moreover, the polymers efficiently loaded and released Spd and crystal violet at 40 °C, compared to 20 °C, at pH 4.5 and 7.5 in buffer and pH 7 in simulated phloem.

#### 3.3.2 DNA delivery

The effective protection of DNA from degradation and its transport into cells currently remain a significant challenge in plant sciences. In particular, nanomaterials show promising properties for allowing a less complicated DNA transfection method compared to the classical methodologies of transgenic plant production, such as physical methods (*e.g.*, electroporation^407^ or biolistic particle delivery^408^) or biological methods (*e.g.*, *Agrobacterium*-mediated transfer^786^). In the general introduction, we have emphasized that the use of nanomaterials to advance the agrochemical field has already been acknowledged, as evidenced by initial commercial successes. In particular, nanoparticles are expected to play a pivotal role in the future delivery of nucleic acid-based cargoes by enabling efficient transfection. In the following examples, we will highlight nanomaterials that have been employed to transfect nucleic acids into plants, utilizing supramolecular interactions not only to capture these biomolecules but also to facilitate their release into plant cells. The first examples of nanoparticle-mediated delivery have been reported using viral capsids, positively charged cell-penetrating peptides,^787–789^ lipids^215,790^ or neutrally charged polyethylene glycol.^791^

**Fig. 75:**
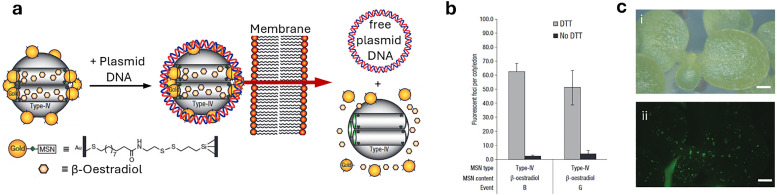
(a) Gold nanoparticle-capped mesoporous silica nanoparticles (MSN) loaded with β-oestradiol can adsorb plasmid DNA on their surface, facilitating the co-delivery of both cargos into plant cells. Once the particles cross the cell membrane, the plasmid DNA is released. In the reductive environment inside the cell, disulfide bonds linking the gold caps to the mesoporous silica are reduced, leading to the disassembly of the caps from the particle surface and enabling the release of the plant hormone β-oestradiol. (b) Fluorescent foci per transgenic cotyledon grown with (grey bar) or without (black bar) DTT after bombardment with MSNs. (c) (i) Bright field and (ii) UV light/GFP filter (scale bar: 0.5 mm) images of non-transgenic plants in DTT-medium and bombarded by DNA-coated type-IV MSNs. Figure reproduced with permission from ref. 792.

In 2007, Lin, Wang, and co-workers^792^ developed MSPs with stimuli-responsive properties for delivering small molecules and plasmid DNA to protoplasts (Fig. 75). These MSPs, loaded with small organic molecules, such as β-oestradiol or dyes, have their pore-entrances blocked by gold nanoparticles attached *via* disulfide linkers on their surface. In addition, this system enables the transport of plasmid DNA into plant cells, as the DNA can adsorb onto the surface of the silica particles through a likely combination of hydrogen bonding and dispersion interactions. A stimuli-responsive release of β-oestradiol into non-transgenic plants (*via* biolistic particle delivery system) can be achieved when the disulfide is reduced by other thiols such as dithiothreitol (DTT). In a follow-up study from 2014,^793^ this system was used to deliver Cre recombinase enzyme (loaded inside the pores of the particles) through the biolistic method to maize (*Zea mays*) cells containing loxP (a specific 34 base pair DNA sequence recognised by the Cre recombinase enzyme) sites integrated into chromosomal DNA (Lox-corn). Due to the supramolecular nature of the DNA–silica interaction in this example, the bound DNA could be released in plants.

**Fig. 76:**
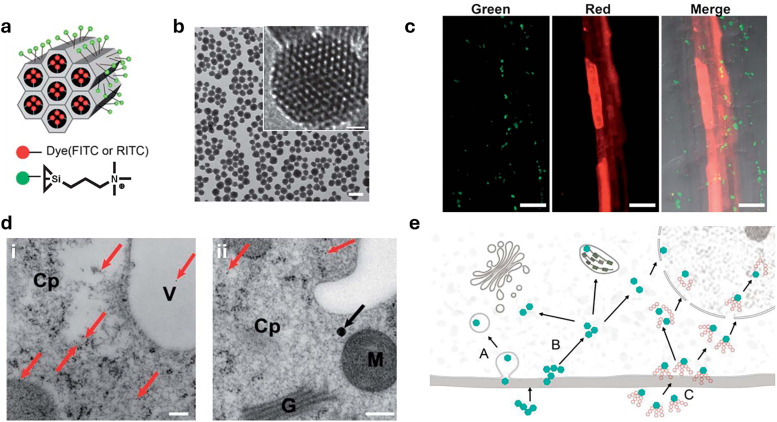
(a) Schematic representation of organically functionalised mesoporous silica nanoparticles. (b) TEM images of TMAPS functionalised FITC-mesoporous silica nanoparticles (TMAPS/F-MSNs). (c) Confocal microscopy of *Arabidopsis* root cells, *i.e.*, endodermal cells, treated with DNA–MSN complexes (1 : 100 ratio). Gene expression (mCherry protein; red). (d) TEM of immunogold-labelled mCherry protein in root cells after incubation with DNA–MSN complexes. Red arrows show the gold-labelled mCherry proteins. Presence of TMAPS/F-MSNs (black arrow) and mCherry protein (red arrows) in the same cell (i) and (ii). Scale bars are 200 nm. Cp, cytoplasm; M, mitochondrion; V, vacuole; G, Golgi apparatus. (e) Possible routes and fates of TMAPS/F-MSNs after internalisation into the *Arabidopsis* root cell. Once passed through the cell wall, TMAPS/F-MSNs may be internalised by endocytosis (A) or penetrate the plasma membrane (B). The DNA-loaded TMAPS/F-MSN complex internalised into the plant cell (C) could then approach the nucleus. Figure reproduced with permission from ref. 228.

Then, a passive DNA delivery system based on MSPs was designed by Hsing, Mou and co-workers,^228^ enabling the passive and non-stimuli-responsive delivery of plasmid DNA (pDNA) into deeper tissues of *Arabidopsis thaliana* root (Fig. 76). A plasmid containing a red fluorescent protein (mCherry) gene driven by a constitutively expressed cauliflower mosaic virus 35S promoter is adsorbed through supramolecular interactions on fluorescein-loaded and *N*-trimethoxysilylpropyl-*N*,*N*,*N*-trimethylammonium chloride (TMAPS) MSNs *via* electrostatic interactions. The pDNA-loaded TMAPS/F-MSN (0.20 μg plasmid DNA encoding mCherry protein; 20.0 μg TMAPS/F-MSN) were used to treat *Arabidopsis* roots for 48 h. MSN are internalised in 52.5 ± 0.1% of the plants studied during the flowering stage and in 3.3% during the vegetative stage, with a transfection efficiency in the flowering plants of 46.5%. Interestingly, effective DNA transfection occurs even though the size of these NPs was larger than the generally accepted pore diameter of the plant cell wall (*i.e.*, 5 to 20 nm).^794,795^ Although the exact reasons are not clear, it is assumed that the relaxation process of the cell wall loosens the network of microfibrils in the cell wall structure and allows the MSNs to pass through.

**Fig. 77:**
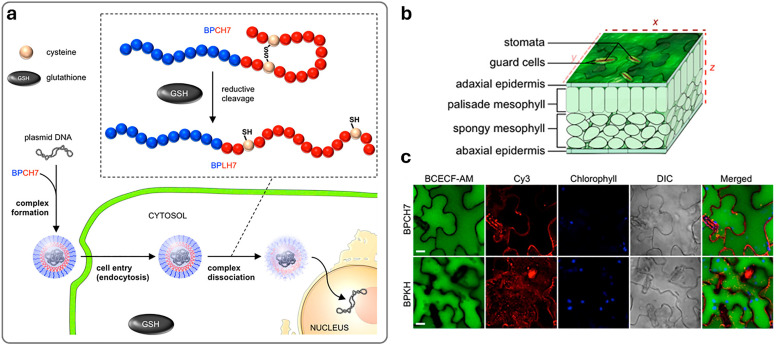
(a) Schematic representation of the glutathione-reducible peptide (BPCH7) and the proposed mechanism for intracellular delivery and subsequent pDNA release. BPCH7 (KKLFKKILKYLHHCRGHTVHSHHHCIR) forms a stable complex with plasmid DNA in the extracellular environment. Once the complex is delivered into the plant cell *via* endocytosis, the reductive intracellular environment, primarily mediated by glutathione (GSH), triggers the cleavage of the intramolecular disulfide bond within the cyclic CH7 domain. This cleavage leads to the dissociation of the complex and the subsequent release of pDNA, allowing its expression in the cell nucleus. (b) Cartoon of a leaf indicating locations of the adaxial and abaxial epidermis as well as palisade and spongy mesophyll cells. (c) Confocal images tKEN from vacuolar compartmentation of BCECF-AM in wild-type *A. thaliana* leaf epidermal cells. Scale bars indicate 10 μm. Figure reproduced with permission from ref. 796.

At this point, it should be highlighted that one major hurdle in this context is represented by the efficient release of the DNA cargo from nanoparticles for successful gene therapies. In the quest for stimuli-responsive systems, enabling a more effective cargo delivery, *i.e.*, nucleic acids, Numata and co-workers^796^ designed dual-domain peptides with cell-penetrating, as well as a DNA-binding site, which is capable of encapsulating and releasing plasmid DNA (pDNA) in the reductive environment of the cell (Fig. 77). Based on previously reported peptide nano-assemblies for pDNA or double-stranded (dsDNA) delivery^797,798^ in this example a peptide (KKLFKKILKYLHHCRGHTVHSHHHCIR) featuring a reducible disulfide bridge within the pDNA-binding domain was used. In this design, the plasmid DNA (pDNA) is entrapped within the micelle core, which forms through the self-assembly of the amphiphile. The DNA is retained in the interior due to an entrapment effect. This design facilitates cellular environment-responsive DNA release, as the reducing environment inside the cell cleaved the disulfide bridge, thereby releasing the DNA. Thus, the spherical plasmid DNA-peptide nanocarrier (*D* = 170–200 nm, with a peptide/DNA ratio of 0.5) can transport the nucleic acid cargo inside the cell with the ability to escape the endosome: once the disulfide bridge is reduced, enabling the peptide to attain a linear conformation, the plasmid DNA was released, since the higher chain flexibility compromises the pDNA packing efficiency. *In vivo* studies on leaves of *Arabidopsis thaliana* show that transfection into leaves enabled plasmid DNA delivery within transgene expression levels reaching 90% already after 3 h and reaching a maximum after 12 h of transfection.

**Fig. 78:**
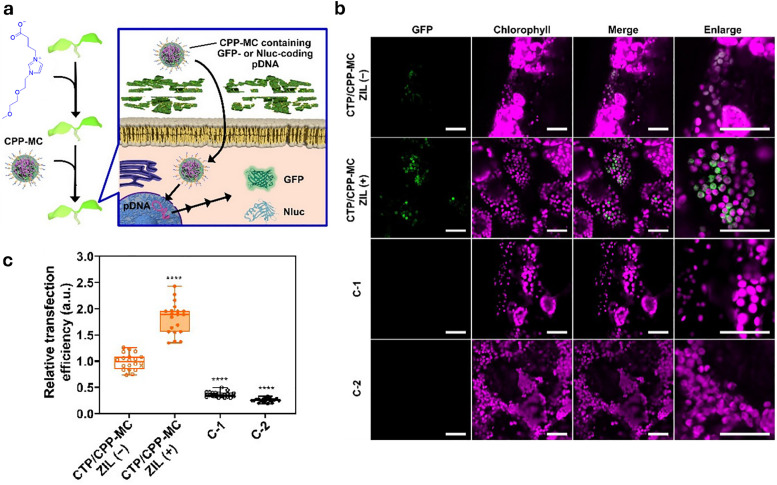
(a) Schematic representation of CTP/CPP-MC-mediated transfection of chloroplasts with reporter genes (GFP or *Renilla luciferase* (Rluc)) in plants pretreated with ZIL. (b) CLSM images showing GFP expression in epidermal cells in ZIL-untreated and ZIL-pretreated *A. thaliana* cotyledons 24 h after transfection with CTP/CPP-MC or controls (naked pDNA or CTP/CPP-MC containing pDNA for nucleus transfection (P35S-GFP-Tnos)). Scale bars = 40 μm. (c) Boxplot showing the relative transfection efficiency of each system, based on Rluc expression levels in ZIL-pretreated *A. thaliana* seedlings 24 hours post-infiltration. Statistical significance is evaluated in comparison to the control (CTP/CPP-MC, ZIL). Figure reproduced with permission from ref. 799.

Numata, Miyamoto, and co-workers^799^ showed that the zwitterionic liquid (ZIL) 4-(1-(2-(2-methoxyethoxy)ethyl)-1*H*-imidazol-3-ium-3-yl)butanoate^800^ enhanced the permeability of plant cell walls to polymeric nanoparticles composed of polycationic peptides (MAL-TEG-(KH)14) and DNA (reporter gene for GFP or NanoLucTM luciferase (Nluc)-encoded pDNA), with the DNA serving as the gene delivery cargo (Fig. 78). Here, the supramolecular interactions are twofold: first, the positive charge of the amphiphile enables effective DNA adsorption through Coulombic interactions; second, the amphiphilic nature of the surfactant drives its self-assembly *via* non-covalent forces, thereby facilitating delivery while protecting the cargo. Additionally, the resulting nanoparticle was further covalently functionalised on its surface with a cell-penetrating peptide (CPP; structure: CKXAKXAKXAGWWG-NH2, X = α-aminoisobutyric acid (Aib)), abbreviated as CPP-MC. Pretreatment of *Arabidopsis thaliana* seedlings and plants with ZIL (0–400 mM for 3 hours) increases the cellular uptake capacity of 100 nm-sized CPP-MC nanoparticles 2-fold in seedlings and 2.4-fold in leaves during transfection experiments. The ZIL pretreatment also exhibits superior efficiency compared to the use of the commercially available agricultural surfactant Silwet L-77. Then, a chloroplast-targeting CPP-MC was also used, in which the targeting capabilities of the nanoparticle were derived from its surface functionalisation with the chloroplast-targeting peptide MASSMLSSATMVGGC-NH_2_ (developed from Rubisco small subunit 1A),^778^ which effectively releases GFP- or *Renilla luciferase* (Rluc)-encoding DNA.

**Fig. 79:**
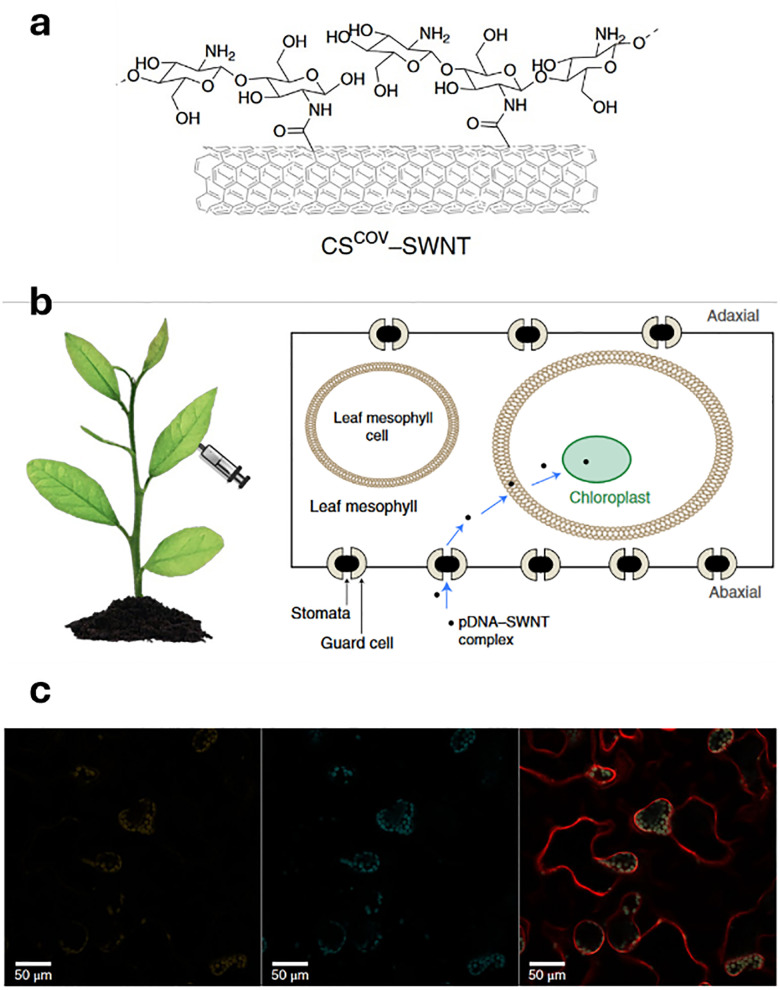
(a) Chemical structure of chitosan-complexed SWCNT. (b) The pDNA–SWCNT complexes enter the leaf mesophyll through the stomatal pores, passing through the plant cell walls, plasma membranes, and ultimately the chloroplast bilayers. The negatively charged pDNA is condensed onto the positively charged surface of the chitosan-complexed SWNTs through electrostatic interactions. (c) Fluorescence confocal micrographs of mesophyll cells from tobacco leaves infiltrated with pDNA–SWNTs (1 : 3 ratio, 1.5 mg L^−1^ of SWNTs) were captured 2 to 3 days post-infiltration. Figure reproduced with permission from ref. 801.

In the first example, Strano and co-workers^801^ were able to show that gene transfer into a specific organelle of mature plants was effectively possible with supramolecularly coated chitosan-modified SWCNTs (Fig. 79). Here, chitosan ensures that the SWCNT has a positively charged surface to which the DNA can bind electrostatically. The advantage of SWCNTs relies in their ability to protect the DNA from degradation by nucleases and can accumulate in the chloroplast^213,802^*via* a lipid exchange envelope penetration (LEEP) model.^269^ Therefore, SWCNTs represent an interesting nanocarrier for the delivery of larger biomolecules, such as pDNA, without the need for additional external agents. Thus, selective DNA delivery to chloroplasts of mature plants was demonstrated by infiltrated pDNA–SWCNT assemblies to the leaves of a four-week-old arugula (*E. sativa*) by a localised infiltration method. The estimated efficiency with which the pDNA cargo was transported into the chloroplast and subsequently transiently expressed reached up to 47% at a SWCNT ratio of 3 : 1 at a concentration of 1.50 mg L^−1^. About 20.0 ng of pDNA is required for transgene delivery and expression in the chloroplast, which is 1000 times less than the typical amount (20.0–50.0 μg) used for PEG-mediated protoplast transformation and 250 times less than the amount needed for biolistic plastid transformation (5.00 μg).

**Fig. 80:**
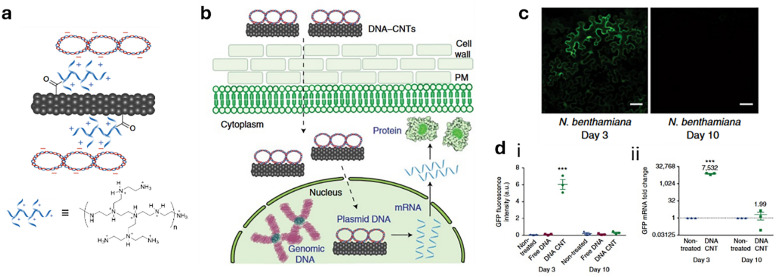
(a) Schematic representation of PEI-modified carboxylated CNTs. (b) Schematic depicting DNA–CNT trafficking in plant cells and subsequent gene expression (dotted lines represent trafficking steps and the rigid lines represent gene expression steps). PM, plasma membrane. (c) Representative confocal microscopy images of pDNA–PEI–CNT-infiltrated mature Nb leaves imaged at days 3 and 10. (d) (i) Quantitative fluorescence intensity analysis of confocal images at 3 and 10 days post-infiltration. (ii) qPCR analysis of GFP mRNA expression levels at day 3 and day 10 in pDNA–PEI–CNT-treated Nb leaves. Figure reproduced with permission from ref. 803.

Moreover, Landry and co-workers^803^ developed a nano platform with SWCNTs functionalised with PEI to electrostatically bind negatively charged pDNA encoding GFP (Fig. 80). Effective gene expression in arugula, cotton and wheat leaves by leaf infiltration was shown, applying to both dicotyledonous and monocotyledonous plants. The pDNA-PEI-SWCNT formulation is more than 700 times more efficient in pDNA transfer than when using pDNA on non-functionalised MWCNT. In *Nicotiana benthamiana* (Nb) leaves treated with pDNA-PEI-CNT, authors observed a more than 7500-fold increase in GFP mRNA on the third day after infiltration, which decreased to an insignificant two-fold mRNA change by the tenth day, indicating that maximal GFP expression occurs on the third day and persists until the tenth day. In a future vision, CNTs combined with genome editing tools – such as zinc finger nucleases (ZFNs), transcription activator-like effector nucleases (TALENs), CRISPR systems including CRISPR-associated protein 9 (Cas9) and Cpf1 from *Prevotella* and *Francisella* – could facilitate highly efficient genome modification without the integration of transgenes, thereby offering a means to bypass stringent GMO regulations. This is particularly advantageous for heterogeneous plants, such as cassava, cocoa and sugar cane, where the removal of transgenes by crossing is not possible.

More recently, the use of polyethylenimine as a DNA-absorbing layer, *via* electrostatic interactions, around magnetic Fe_3_O_4_ nanoparticles has proven to be an effective chemical modification for binding plasmid DNA.^804^ This modification enables the formation of nanoparticles with a magnetic core and PEI shell carrying DNA on their surface, facilitating the development of a new route for the transfer of genetic material to cells, known as pollen magnetofection. This latter approach represents a simple and cost-effective way of transferring genetic material to plants, thereby avoiding the complex procedures requiring regeneration from tissue cultures. In this way, pollen could be loaded with the DNA-loaded particles and sprayed onto the flowers of plants.

**Fig. 81:**
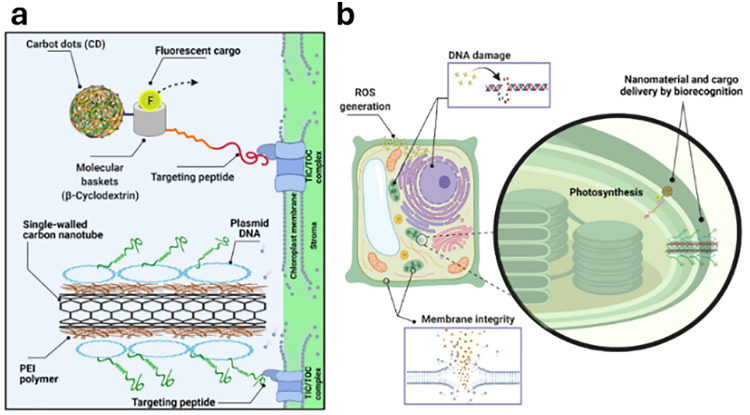
(a) Targeted carbon nanostructures for chloroplast bioengineering were developed to explore their effects on plant cell and molecular biology. Nanomaterials were synthesised for chloroplast-targeted chemical delivery (CDs) and gene delivery (SWCNTs). These carbon nanostructures were functionalised with a guiding peptide that specifically binds to the translocon of the outer chloroplast membrane (TOC) proteins. (b) The impact of targeted carbon nanostructures on leaf cell and molecular biology was assessed by studying the effects on plant cell and chloroplast membrane integrity, the damage to whole plant cell and isolated chloroplast DNA, the generation of ROS, and photosynthesis. Figure adapted with permission from ref. 805.

In addition, Giraldo and co-workers^805^ reported a delivery platform, using CDots and SWCNTs, based on supramolecular design principles and applied directly to plant film using a spray deposition method, as opposed to infiltrating the nanomaterial into the plant (Fig. 81). For the transport of small organic molecules, a system of fluorescent CDots functionalised with βCD, which in turn was conjugated with a chloroplast target peptide (MASSMLSSATMVGGC, TP), abbreviated as TP-βCD, was prepared. The cavity of βCD serves as a macrocyclic host for a small molecule, such as fluorescein (FDA), leading to the formation of an inclusion complex with TP-βCD, forming TP-βCD-FDA. For the chloroplast-targeted delivery of DNA, authors utilised SWCNTs functionalised with the cationic polymer polyethyleneimine, which bound electrostatically to DNA, and a plastid-specific promoter (pATV1).^806^ The promoter was further labelled with a targeting peptide (MASSMLSSATMVGGGGGGKHKHKHKHKHKH), where the KH6 tail of the peptide binds through electrostatic interactions to the DNA, forming TP-pATV1-SWCNTs. When applied topically to *Arabidopsis thaliana* leaves, TP-βCD-FDA and TP-pATV1-SWCNTs enable effective translocation of small molecules and DNA to chloroplasts, improving delivery efficiency from 47% to 70%, and from 39% to 57%, respectively. Since CDots are inherently luminescent, dual imaging and delivery for plants can also be envisioned for potential imaging and delivery applications. However, SWCNTs are better suited for the transport of larger DNA molecules, due to their ability to bypass the cell wall and lipid membranes of plants.^807^

**Fig. 82:**
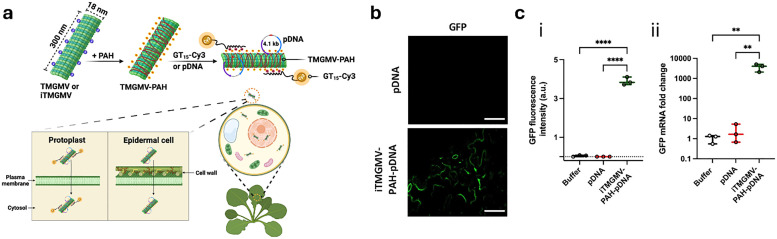
(a) Virus-like nanocarriers facilitated DNA delivery in *Arabidopsis* plant cells. Negatively charged TMGMVs or inactivated TMGMVs (iTMGMVs) were coated with poly(allylamine) hydrochloride (PAH) to impart a positive charge, forming TMGMV-PAH. These were electrostatically loaded with either a DNA oligo (GT15, 30 bp ssDNA) linked to a Cy3 dye (TMGMV-PAH-GT15-Cy3) or pDNA encoding GFP. Nanocarriers and DNA spontaneously entered plant cells through energy-independent mechanisms. iTMGMV-PAH successfully mediated pDNA delivery and expression in *Arabidopsis* epidermal cells. (b) Confocal microscopy images of *Arabidopsis* leaves monitoring the pDNA delivery (encoding for GFP) and expression mediated by iTMGMV-PAH-pDNA. Scale bars 30 μm (c) (i) Fluorescence intensity indicating GFP expression in leaf epidermal cells infiltrated with iTMGMV-PAH-pDNA. (ii) RT-qPCR analysis of GFP mRNA expression levels 2 days post iTMGMV-PAH-pDNA infiltration in *Arabidopsis* leaves. Figure adapted with permission from ref. 808.

DNA delivery using virus-like nanoparticles in protoplasts and intact *Arabidopsis thaliana* plants, which utilises the supramolecular complexation of single-stranded and plasmid DNA, was achieved using polycationic particles of TMGMV.^808^ These TMGMVs (Fig. 82) functionalised with poly(allylamine) hydrochloride (PAH; leading to TMGMV-PAH) have a size of 310 ± 1.3 nm. When TMGMV-PAH is loaded with GFP-encoding pDNA, a loading ratio of TMGMV-PAH/pDNA = 1 : 1 to 1 : 12 was observed, with a loading efficiency always being 100% (as confirmed by gel electrophoresis). Adsorption onto the nanoparticles was shown to prevent DNA degradation by DNases. It should be noted that TMGMV particles must be deactivated by UV light before use (iTMGMV), to prevent infection of the plant by the virus. After UV inactivation, iTMGMV can be used to transfer pDNA to *Arabidopsis* leaves without significant toxicity being observed, up to a dose of 0.15 mg mL^−1^ iTMGMV-PAH. However, a significant increase in cell death (15.8 ± 2.2%; *P* < 0.01) was observed at a concentration of 0.90 mg mL^−1^ pDNA.

**Fig. 83:**
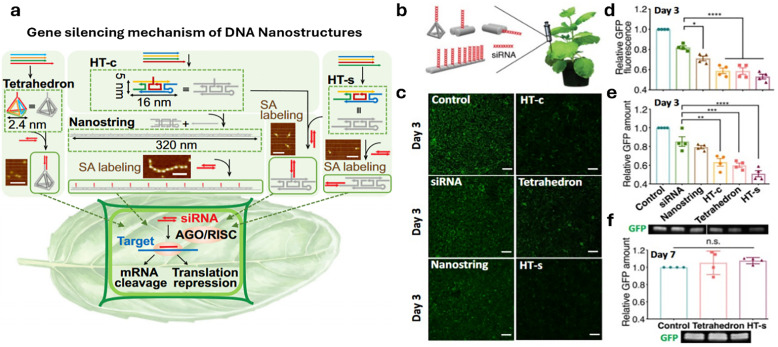
(a) The DNA nanostructures were synthesised from four ssDNA sequences to form tetrahedrons and HT monomers, with 1D nanostrings produced by HT monomer polymerisation using an initiator strand. Cargo attachment sites were located at the apex of the tetrahedron, along the nanostring, and at the side (HT-s) or centre (HT-c) of HT structures. AFM images showed streptavidin-bound biotinylated HT monomers and nanostrings with siRNA cargo. Cy3 or siRNA-loaded nanostructures were infiltrated into transgenic mGFP5 Nb plant leaves for further studies. Scale bars, 100 nm. (b) Infiltration of siRNA-linked DNA nanostructures into mGFP5 Nb leaves. (c) Representative confocal images of leaves infiltrated with siRNA nanostructures 3 d post infiltration, with nontreated control leaves. Scale bars, 100 μm. (d) Fluorescence intensity analysis of confocal images. (e) Representative western blot gel of GFP extracted from nanostructure-treated leaves 2 d post infiltration. (f) Representative western blot of GFP extracted from leaves treated with siRNA linked to tetrahedron or HT-s 7 d post-infiltration. Figure adapted with permission from ref. 809.

DNA nanotechnology represents an exquisite field in which sequence-defined DNA strands are used to construct 3D DNA-based nanostructures with high precision and yield, all directed by Watson–Crick–Franklin base-pairing interactions (see Section 1.5.9). DNA technology and DNA-based nanostructures are nowadays gaining increasing attention for developing nanocarriers for nucleic acid delivery, as demonstrated also by Landry and co-workers (Fig. 83).^809^ Particularly, in their study, three DNA nanostructures were synthesised: a 3D tetrahedron (2.4 nm), a 1D hairpin tile (HT) monomer (2 × 5 × 16 nm), and a 1D high aspect ratio nanostring (2 × 5 × 320 nm), each programmed to bind DNA, RNA, or protein, at predefined sites *via* the above mentioned interactions between nucleobases. In *In vitro* studies on *Nicotiana benthamiana* (Nb), leaves show an energy-dependent internalisation mechanism, and smaller nanostructures present higher internalisation due to their ability to stay below the plant cell wall exclusion limit.^810,811^ Fluorescently labelled DNA strands with siRNA shows that compact nanostructures achieve higher cellular uptake in mGFP5 Nb plants (59.5 ± 1.5% for HT monomer and 54.4 ± 2.7% for tetrahedron, compared to 35.8 ± 0.9% for nanostring). Higher bending stiffness is also correlated with increased uptake. For gene silencing, siRNA-loaded DNA nanostructures were introduced into mGFP5 Nb leaves at 100 nM siRNA concentration. Here, GFP fluorescence decreased by ∼29.0 ± 4.6% in siRNA-functionalised nanostrings, 41 ± 5.4% and 47 ± 4.7% in HT monomers with siRNA bound at the centre and side, respectively, and 42.0 ± 6.5% in siRNA-conjugated tetrahedrons compared to untreated leaves.

**Fig. 84:**
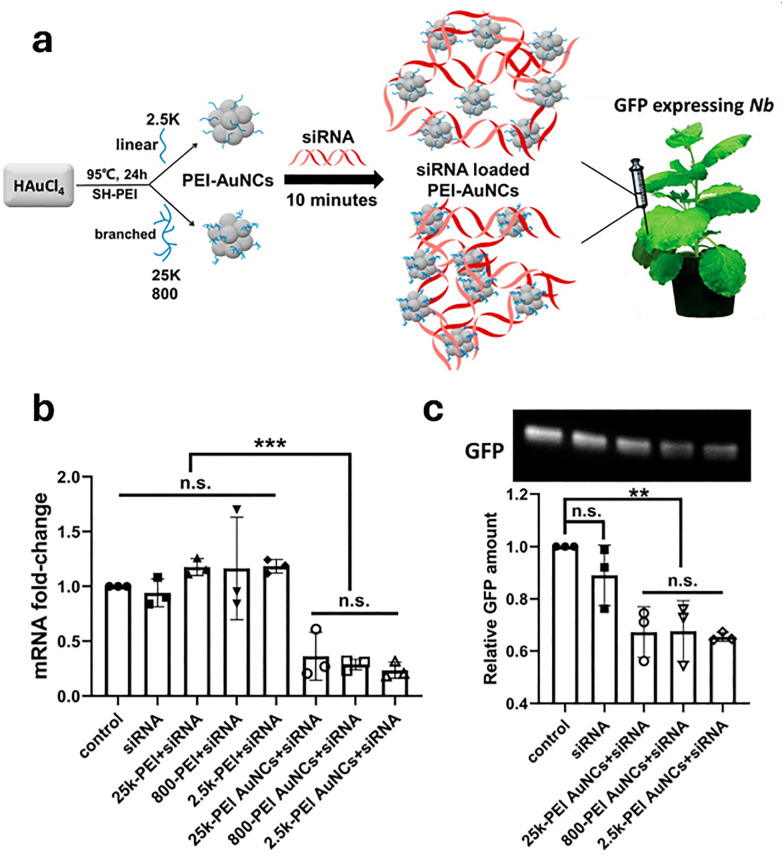
(a) A schematic representation of the synthesis of PEI-AuNCs (utilising PEI with average molecular weights of 800, 2.5k, and 25 kg mol^−1^), followed by siRNA loading *via* electrostatic adsorption and subsequent infiltration-based delivery into mature mGFP5 Nb plant leaves for gene silencing. (b) siRNA delivered by 800-, 2.5k-, and 25k-PEI-AuNCs can induce efficacious gene silencing as shown by qPCR to quantify GFP mRNA fold changes 1-day post-infiltration with water (control), free siRNA, positive control of siRNA mixed with free PEI polymers (800, 2.5k, and 25k), and siRNA-loaded PEI-AuNCs. (c) Representative western blot gel (top image) and statistical analysis of GFP proteins extracted from leaves treated with water (control), free siRNA, or siRNA-loaded PEI-AuNCs 3 days post-infiltration. Figure adapted with permission from ref. 812.

Moreover, Landry and Yang's groups demonstrated that polyethyleneimine-functionalised gold nanoclusters (PEI-AuNCs) can silence GFP transgene expression in transgenic mGFP5 *Nicotiana benthamiana* (Nb) plants *via* abaxial leaf infiltration (Fig. 84).^812^ Indeed, the positively charged PEI-AuNCs (1–2 nm) electrostatically bound siRNA, and gold nanoparticles modified with a 2.50 kg mol^−1^ lipoic acid-PEI polymer showed the highest siRNA loading capacity. More in detail, 80.0 ng of 2.5k-PEI-AuNCs bound 120 ng of siRNA, forming supramolecular aggregates (15–40 nm). These 2.5k-PEI-AuNCs silenced GFP transgenes in mGFP5 Nb plant leaves and the ROQ1 gene in wild-type Nb leaves with efficiencies of 76.5 ± 5.90% and 76.1 ± 9.50%, respectively (1-day post-infiltration). The luminescent properties and ease of preparation of gold nanoclusters are promising, but their cost for synthesis and potential for bioaccumulation in mammals and insects require further evaluation. Furthermore, unlike DNA-based nanovectors or SWCNTs, which can be applied *via* spraying, gold nanoparticles necessitate injection, which may limit their use to research-scale studies or small-scale applications.

**Fig. 85:**
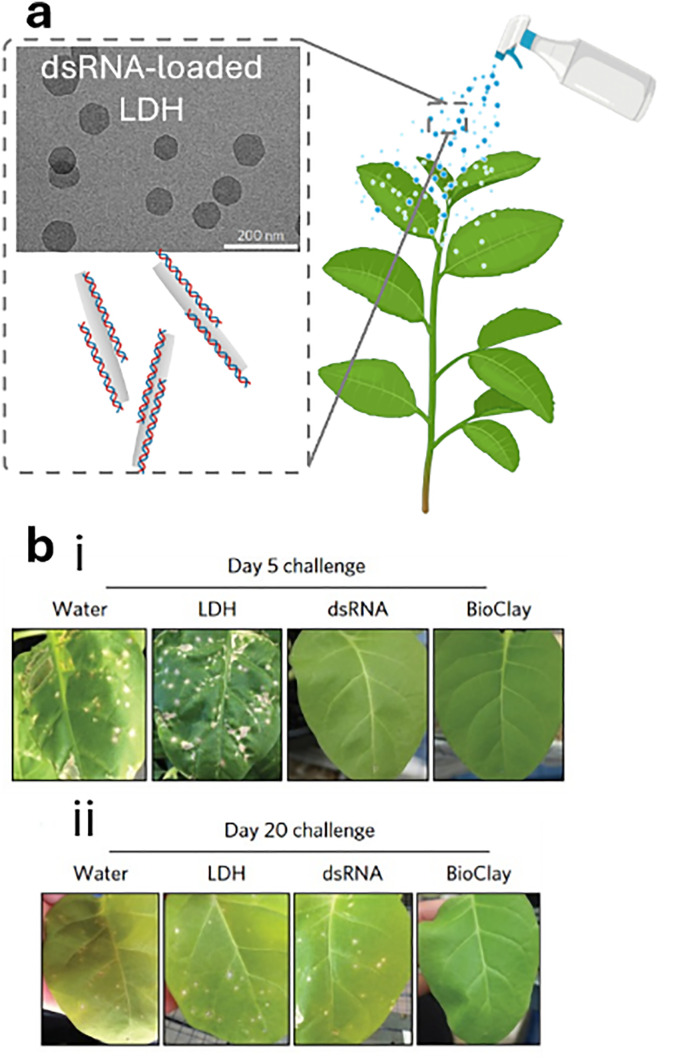
(a) The BioClay experiment was conducted by spraying the plants with LDH, CMV-dsRNA and CMV-BioClay (CMV-dsRNA–LDH). The inset shows the TEM image of LDH nanoclays and a schematic representation of BioClay. (b) (i) Images showing the extent of necrotic lesions on *N. tabacum* cv. *xanthi* leaves challenged with PMMoV 5 days post-spray treatment and, (ii) 20 days post-treatment. Figure adapted with permission from ref. 813.

An interesting approach in delivering dsRNA for RNAi therapies (through spray-on treatments on leaves) has been shown by aluminosilicates, such as layered double hydroxide (LDH) clay nanomaterials with a lamellar structure. In this respect, Mitter, Xu, and co-workers^813^ demonstrated that LDH nanosheets (*D* = 15–120 nm and lateral size of 20–80 nm) could form dsRNA–LDH complexes (*i.e.*, BioClay), which protected dsRNA from nuclease activity and allowed for its detection on leaf surfaces up to 30 days after application (Fig. 85). DNA adsorption onto clay minerals is primarily governed by electrostatic interactions, hydrogen bonding, ligand exchange, and cation bridging. Protonation of amino groups in DNA bases (adenine, guanine, cytosine) enhances binding to the charged clay surface.^814^ Therefore, BioClay enabled sustained dsRNA release under ambient conditions and provided RNAi-based systemic protection against cucumber mosaic virus (CMV) when tested on Cowpea (*Vigna unguiculata*) plants, and against pepper mild mottle virus (PMMoV) on *N. tabacum* cv. *xanthi* leaves, remaining effective even 20 days after a single spray. A 1 : 3 dsRNA–LDH loading ratio was employed in all crop protection experiments, rather than the full 1 : 4 ratio, to ensure the immediate availability of a portion of free dsRNA for enhanced protective efficacy. Spray treatments were conducted at approximately 125 μL cm^−2^ (*i.e.*, 1.25 μg of dsRNA and/or 3.75 μg of LDH) of the leaf surface. Additionally, dsRNA was taken up by plant cells to trigger RNAi against homologous RNA. Furthermore, as the LDH nanomaterials degraded over time and consisted solely of aluminosilicate, these systems pose little biological risk when applied in the environment.

**Fig. 86:**
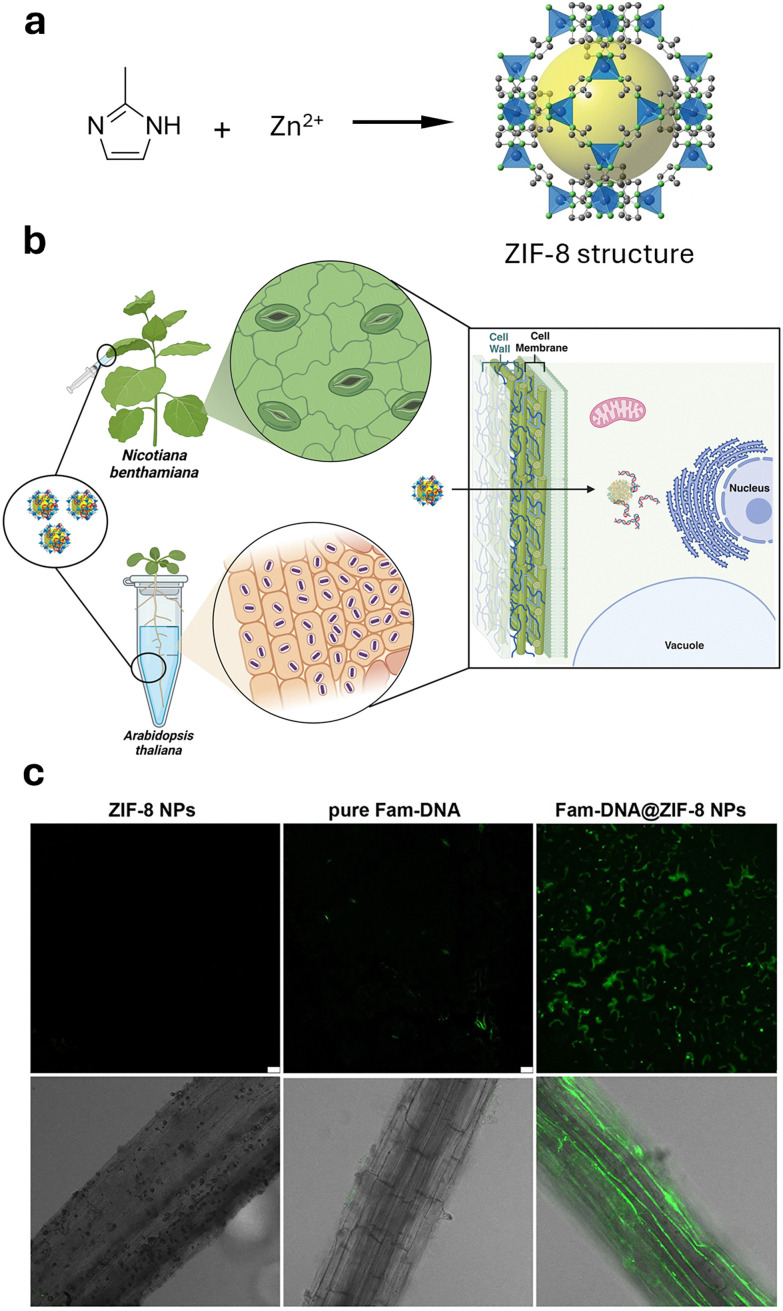
(a) Schematic representation of ZIF-8 building blocks and their structure (H atoms are omitted for clarity). The yellow sphere represents the void volume within the ZIF-8 structure. (b) Schematic representation of ZIF-8 nanoparticle-mediated gene delivery into *Nicotiana benthamiana* leaves and *Arabidopsis thaliana* roots. (c) Confocal images of *Nicotiana benthamiana* leaves and *Arabidopsis thaliana* roots post-infiltration. The representative images display the cellular uptake of pure ZIF-8 NPs, pure FAM-labelled DNA, and FAM-labelled DNA-loaded ZIF-8 NPs in *Nicotiana benthamiana* leaf cells and *Arabidopsis thaliana* root cells. Scale bar: 20 μm. Figure adapted with permission from ref. 815.

As an alternative to this approach, Khashab and co-workers^815^ reported using MOF nanoparticles to infiltrate siRNA into *Nicotiana benthamiana* leaves and *Arabidopsis thaliana* roots (Fig. 86a and b). Small ZIF-8 nanoparticles (<20 nm)^816^ were loaded with siRNA-RNA at a ratio of RNA NPs = 1 : 75. RNA–ZIF-8 interactions likely occur *via* electrostatic adsorption, with nucleic acids released in the plant's acidic interspace. As a result, infiltrated RNA@ZIF-8 NPs (3 μg mL^−1^) in *N. benthamiana* showed over 50% colocalisation with GFP and no leaf damage after three days, with RNA beng significantly protected from RNase degradation. A 22-bp siRNA sequence targeting the cHLH gene confirmed the effective siRNA delivery and gene silencing, with functional–siRNA@ZIF-8 NPs significantly reducing mRNA levels. FAM-labelled DNA-loaded ZIF-8 NPs, tested in both leaves and roots, demonstrated higher DNA uptake and specificity compared to pure FAM-DNA, with effective DNA delivery in *Arabidopsis thaliana* roots, as was shown by confocal imaging (reported in Fig. 86c).

**Fig. 87:**
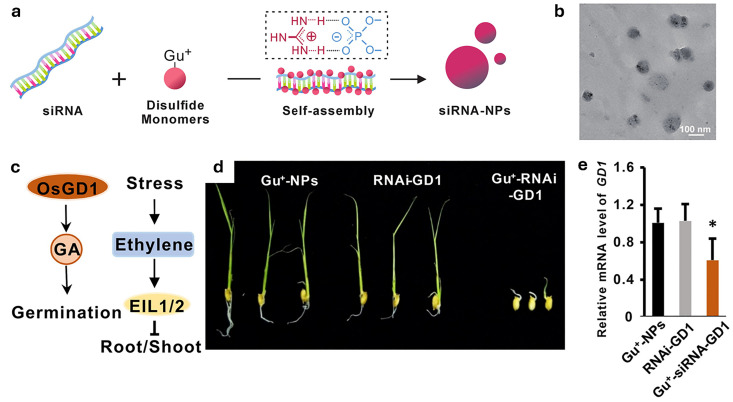
(a) Schematic representation of the formation of Gu^+^–siRNA nanoparticles *via* electrostatic interactions between guanidinium (Gu^+^)-containing disulfide molecules and the phosphate groups (PO_4_^−^) of siRNA. (b) TEM image of Gu^+^–siRNA NPs. (c) Illustration of GD1 and EIL1/2 gene functions in rice seed germination and salt stress response. (d) Gu^+^–siRNA-GD1 NPs inhibit rice seed germination. (e) Relative expression levels of GD1 following treatment. Gu^+^–siRNA-EIL1/2 NPs promote coleoptile elongation *via* long-distance transport from root to shoot. Figure reproduced with permission from ref. 818.

Building on previous work by Zhou *et al.*^817^ demonstrating that guanidinium (Gu^+^)-containing disulfide molecules (GDM) can self-assemble with siRNA into nanoparticles (Gu^+^–siRNA NPs) for endocytosis-independent delivery in mammalian systems, Han, Gu, Yang, and coworkers adapted this strategy for plant systems to address the major challenge of systemic RNA transport (Fig. 87).^818^ The Gu^+^ moieties form electrostatic interactions with siRNA phosphate backbones, inducing disulfide exchange polymerization and yielding stable, spherical nanoparticles (∼200 nm; Fig. 87a and b) with high siRNA loading efficiency (N/P > 15 : 1). These nanoparticles protect siRNA from enzymatic degradation under a broad range of physiological conditions (pH 5.0–9.0, temperatures of 4–37 °C, and up to 3% salt), while maintaining colloidal stability. Importantly, biocompatibility tests in *Arabidopsis thaliana* protoplasts revealed minimal cytotoxicity at concentrations up to 1.5 mM – significantly outperforming polyethylene glycol (PEG), a conventional but stress-inducing transfection agent. At this concentration, Gu^+^–siRNA NPs enabled rapid and efficient siRNA uptake into protoplasts within 20 minutes. Systemic delivery was validated by immersing only root tips of *Arabidopsis* seedlings in Gu^+^–siRNA-FITC NPs, leading to detectable fluorescence in root, shoot, and leaf tissues within 1 hour, demonstrating vascular translocation. In contrast, PEG-delivered siRNA showed limited transport and severe morphological damage. The delivery pathway operates independently of endocytosis, instead utilizing a thiol-mediated mechanism that bypasses lysosomal degradation and immune activation. This enables robust, long-distance siRNA transport throughout the plant vasculature and supports systemic gene silencing. Functional studies confirmed the silencing of key genes such as STM (shoot meristem regulation) in *Arabidopsis* and EIL1/2 (salt tolerance) in rice, highlighting applicability under abiotic stress conditions. Moreover, Gu^+^–siRNA NPs permit co-delivery of multiple siRNAs, enabling simultaneous silencing of multiple targets. This was exemplified through concurrent suppression of WER and MYB23 (root development in *Arabidopsis*) and EIL1/2 in rice (Fig. 87c–e). Overall, Gu^+^–siRNA NPs present a transformative platform for RNAi-based crop improvement by enabling stable, biocompatible, and systemic siRNA delivery without genetic transformation, supporting broader applications in non-transgenic plant biotechnology.

In summary, in the presented chapter the positive effects of nanocarriers in stabilising and controlling pesticide release have been highlighted. Importantly, nanoparticles can in principle enable a high payload delivery of pesticides while also protecting them against degradation. In this context, the greater use of nanoparticles for developing new gene therapies for plants, by increasing the transfection efficiency of nucleic acid cargo and stabilising it for maximum effectiveness, will undoubtedly be an important research area in the future. The primary interaction between the nanocarrier and DNA is electrostatic, whereby the positively charged carrier binds to the negatively charged nucleic acid. As observed with clays, hydrogen bonding may also contribute to the binding. These interactions strengthen with increasing DNA length but remain sufficiently labile to allow cargo release in plants. Notably, electrostatic adsorption can protect nucleic acids from enzymatic degradation. Future studies should aim at achieving more controlled release mechanisms, ideally triggered by specific stimuli, while maintaining cargo protection. This may be accomplished by employing more host–guest-type supramolecular interactions that are dynamic and responsive to plant metabolites. Furthermore, a highly interesting aspect of using nanomaterials lies in their ability to simultaneously serve imaging and delivery applications, among other functions, making them an ideal choice for designing multifunctional nanopesticides. Moreover, they facilitate the preparation of stimuli-responsive release systems for bioactive molecules, allowing the precise release of pesticides and further improving their efficacy. These features contribute to potentially less toxic pesticide use and enhanced sustainability. Therefore, a key future consideration will be their approval by national and federal environmental agencies. For this to occur, a strong biosafety assessment of these materials will be required, making it an essential area of research interest for the future.

## 4. The role of supramolecular chemistry in advancing sustainable agriculture and environmental protection

An ecologically balanced ecosystem is essential for sustainable agricultural production and management, as evidenced by the formal recognition of biodiversity's importance for global sustainability at the 1992 United Nations Conference on Environment and Development.^819^ Therefore, investing in ecosystem and soil health through sustainable agriculture research can mitigate the negative environmental impacts of conventional agriculture and make both ecosystems and agricultural systems more productive and resilient. The preservation or promotion of ecological balance is increasingly difficult, challenged both by the impacts of climate change and by human activities,^820,821^ most notably the intensifying use of pesticides, with global consumption having exceeded 4 million tons since 2014 and projected to increase by 60% to 100% by 2050.^822,823^ Improper pesticide usage can indeed lead to several risks to public health, too, *e.g.*, through the residual contamination of the food chain,^15–17^ giving rise to several diseases, such as Parkinson's,^12,13^ Hodgkin's,^18^ and Alzheimer's disease,^19,20^ as well as being involved in the pathogenesis of neoplasia, oxidative stress, and various respiratory and reproductive disorders.^21,22^ Traditional agricultural practices have led to long-term ecological imbalances,^25,824^ degrading land and soil,^825^ reducing habitats and biodiversity,^826,827^ accelerating species loss, and causing pollution.^821,828^ By contrast, healthy and functioning ecosystems contribute to crop pollination, water filtration, pests and disease control, and provide additional services that are critical to agricultural land use.

The application of supramolecular chemistry to the detection of plant metabolites, pesticides, and xenobiotics plays a significant role in the rapid and cost-effective identification of plant responses to environmental stressors and pollutants. Early and accurate monitoring of such external factors is crucial for detecting ecosystem degradation at an incipient stage, thereby serving as a reliable early warning system for potential contamination events. In this context, supramolecular concepts provide plant science with, tuneable, and minimally invasive analytical tools for investigating plant physiology, signalling pathways, and stress responses. The ability to detect small molecules, hormones, and biochemical markers in real time and *in situ*, often using simple luminescent measurements, provides quantitative and selective means of investigating complex plant processes under environmentally relevant conditions. These approaches are particularly suitable for the development of portable, on-site sensing devices, as they circumvent the need for sophisticated and resource-intensive methods such as mass spectrometry or high-performance liquid chromatography. Consequently, such devices are not only accessible to non-specialized personnel but are also applicable in remote or resource-limited settings where conventional analytical infrastructure is often unavailable. At the same time, the growing awareness of emerging environmental contaminants such as PFAS and microplastics underscores the urgent need for more advanced and selective detection strategies. Supramolecular chemists increasingly contribute to this challenge by enabling the design of luminescent probes, chemosensors and functional materials capable of detecting and binding such pollutants. In particular, PFAS-binding host systems have become a prominent research focus, aiming to achieve both sensitive detection and effective remediation.^507,569,579,829,830^ Likewise, microplastic^831^ pollution has emerged as a high-priority area of environmental research,^832,833^ given its widespread distribution and the accumulating evidence of its detrimental impacts on human and ecosystem health. While conventional detection methods, primarily based on optical microscopy and IR spectroscopy, are effective for the analysis of larger plastic fragments, they often require extensive sample preparation and remain inadequate for detecting smaller particles and complex matrices. In this context, supramolecular strategies, such as the staining of microplastics by exploiting interactions of a supramolecular nature,^834–836^ hold significant promise for the development of new tools with simplified workflows or the detection.^837^

Today, the development and global application of both new and existing pesticides achieve significant commercial success. This success necessitates, however, a comprehensive understanding of their interactions with plant surfaces, their metabolic pathways, accumulation in plants and soils, and their release kinetics.^838^ In the context of pesticide use, supramolecular complexation has been shown to reduce the acute toxicity of pesticides to mammals and insects, as the resulting host–guest complexes exhibit lower bioavailability due to reduced cellular uptake. At the same time, these supramolecular complexes allow for the use of lower quantities of pesticides, as their persistence on leaves increases due to higher chemical stability, lower vapour pressure, and improved leaf wettability, combined with increased hydrophilicity. These factors together can reduce the overall consumption of pesticides and potentially reduce environmental pollution. Furthermore, the development of supramolecular systems allows the development of stimuli-responsive release mechanisms for pesticides and their nanoformulations, enabling precise control of pesticide activation at specific times. In addition, given the large surface areas of porous nanomaterials, *e.g.*, metal–organic frameworks (up to 7140 m^2^ g^−1^) and mesoporous silica particles (up to 1000 m^2^ g^−1^), sustained release over extended periods is becoming a critical concept for maximizing the efficacy of pesticides. Moreover, release kinetics can be fine-tuned by developing nanomaterials that degrade in the presence of light or enzymatic activity of pests, ensuring that pesticides are only released when external stressors are present. In soils, we can envision a more sustainable and controlled release of nematicides that reduce leaching or diffusion of pesticides in the absence of nematodes by using pH-responsive or enzymatically degradable nanocarriers.

More recently, the so-called “second green revolution”^454^ gradually emerged, driven by new technologies and materials, offering the possibility to work with nanotechnology-based products. These systems demonstrated that nanotechnology-enabled pesticides (namely, nano pesticides) could benefit sustainable agriculture practices,^823^ being characterised by high efficiency, durability, and biocompatibility in the application process.^839–841^ However, there is the risk that the “nano” characteristics of nanopesticides could worsen toxicity for non-target organisms.^842^ For this reason, the US National Science Foundation (NSF) and the EPA encouraged the investigation of such toxicity, together with pesticide destination, transportation, and safety in the environment.^843^ As an example, Fraceto and co-workers^844^ evaluated the environmental impact of nano pesticides on non-target organisms, such as honeybees that forage on crops, resulting in morphological alterations in the bees’ midguts. Furthermore, nano-based formulations resulted also in modifying the persistence of active ingredients in the field, and in being sorbed into the soil.^845,846^ From the perspective of supramolecular chemistry, new materials relying on supramolecular interactions – such as hydrogels designed to improve soil structure and moisture retention, enhance nutrient storage, or protect plants from biotic and abiotic stresses – have shown great potential and have already been extensively reviewed elsewhere.^41,847,848^ Thus, geographically gridded data of agricultural pesticides are crucial to assess ecosystem exposure to potential and/or recognised toxicants,^822^ to avoid severe environmental issues, and there is still an increasing need to explore safer alternatives for pesticides^842^ and take into account the possible fate related to their degradation products.

## 5. General conclusions and perspectives

Feeding a growing global population under the constraints of climate change, biodiversity loss, and environmental degradation necessitates a shift in the way agricultural systems are designed, managed, and protected. In this context, as we have shown here, supramolecular chemistry provides a promising and versatile approach for advancing next-generation agrochemical technologies that are aligned with sustainability goals. Furthermore, supramolecular systems developed through chemical design have the potential to revolutionize the study of plant responses, enabling unprecedented insights directly within living plants. This review critically examines the emerging role of supramolecular strategies in improving agricultural sensing and delivery systems. It covers key concepts underlying supramolecular interactions, chemosensors, molecular probes, and delivery platforms, and considers the biological barriers that affect their real-world applications. Representative examples, including nanoparticle-based systems, are used to illustrate their operational principles, advantages, and limitations. The review concludes with a discussion on how supramolecular systems can contribute to sustainable agriculture and environmental preservation.

In the realm of sensing, supramolecular systems based on noncovalent molecular recognition provide a promising alternative to conventional analytical tools. Chemosensors, molecular probes, and nanoparticle-based formulations can be designed to detect biologically and environmentally relevant analytes, including toxic pesticides and endogenous plant metabolites, directly within plant tissues. Luminescence-based platforms are predominant in monitoring plant responses to abiotic and biotic stressors, offering real-time detection possibilities into plant health and defence mechanisms. Compared to traditional techniques such as mass spectrometry or high-performance liquid chromatography, these supramolecular tools offer significant advantages in simplicity, cost-efficiency, and the potential for in-field deployment. However, challenges such as limited analyte selectivity, low binding affinities, and probe deactivation from nonspecific interactions with plant matrices still impede practical applications. This aspect is particularly important, as many plant metabolites must be detected at low concentrations (nanomolar to low micromolar) within complex, salt- and protein-rich environments. Under such conditions, current chemosensors still face significant challenges due to limited affinity and selectivity, as well as insufficient understanding of intracellular trafficking, transport mechanisms, and deactivation processes (*e.g.*, irreversible protein adsorption). Moreover, real-time monitoring of analyte fluctuations and the development of systems capable of multimodal signal readouts are essential to enable the technological translation into practical sensing devices. Looking ahead, research should also focus on designing sensors for potentially toxic analytes, which may include chemically diverse compounds such as PFAS and microplastics, as well as biological targets like proteins and nucleic acids.

With regard to delivery, supramolecular systems have shown significant promise in improving the efficacy, stability, and environmental profile of agrochemicals. In fact, several macrocycle-based agrochemicals include already cyclodextrin for improving crop protection and growth. Applying the concepts of supramolecular chemistry in combination with nanoparticle-based carriers can protect labile payloads from premature degradation, enhance adhesion to plant surfaces, and enable stimuli-responsive, targeted release. These advances are particularly relevant for the emerging field of nucleic acid-based agrochemicals and plant gene therapies, where the delivery of chemically speaking fragile cargos such as RNA remains a major bottleneck. Nanomaterial-based carriers have demonstrated the capacity to enhance nucleic acid stability and plant tissue penetration, but a detailed mechanistic understanding of uptake pathways, translocation dynamics, and interactions with plant barriers is still lacking. Moreover, comprehensive studies on the biodegradability, persistence, and ecological safety of these materials are needed to ensure their long-term compatibility with agricultural ecosystems.

Beyond technical considerations, the broader adoption of supramolecular technologies in agriculture will require the establishment of clear regulatory pathways, transparent safety assessments, and effective science communication strategies. Public concerns about nanopesticides and other novel materials, often fuelled by a lack of accessible data, have slowed progress in this area. Future work should prioritize full life cycle assessments, long-term environmental monitoring, and toxicological evaluations to address these concerns and build public trust. Equally important is fostering interdisciplinary collaboration among chemists, plant scientists, toxicologists, and policymakers to translate laboratory-scale innovations into scalable, field-ready solutions.

In conclusion, supramolecular chemistry holds considerable promise to reshape the landscape of agricultural science by enabling more selective, efficient, and environmentally compatible technologies. Realizing this potential will require coordinated research efforts focused on improving molecular design, delivery efficiency, system integration, and safety evaluation. As the field evolves, the integration of supramolecular tools into mainstream agricultural practice could play a transformative role in advancing sustainable food production and ecosystem protection.

## Author contributions

All authors contributed to the writing, reviewing, and revising of the manuscript.

## Conflicts of interest

There are no conflicts to declare.

## Supplementary Material

CS-054-D4CS00500G-s001

## Data Availability

All data supporting the findings in this review are available from the referenced sources listed in the manuscript. A list of common abbreviation can be found in the ESI.†
