# Supplementary material for: Supramolecular chemistry for optical detection and delivery applications in living plants
Source: Chem Soc Rev. 2025 Jul 17;54(17):7769–869. doi: 10.1039/d4cs00500g (PMC12268990; doi:10.1039/d4cs00500g)
Supplement: CS-054-D4CS00500G-s001 [file CS-054-D4CS00500G-s001.pdf]

Supplementary Information (SI) for Chemical Society Reviews.
This journal is © The Royal Society of Chemistry 2025

Supporting Information

Supramolecular Chemistry for Optical Detection and Delivery Applications in Living Plants

Maria Vittoria Balli,[a] Frank Biedermann,[b] Luca Prodi,*[a,c] Pierre Picchetti*[b]

[a]Department of Chemistry "Giacomo Ciamician", Università degli Studi di Bologna, Via Selmi 2, 40126 Bologna, Italy
[b]Institute of Nanotechnology (INT) Karlsruhe Institute of Technology (KIT), Kaiserstrasse 12, 76131 Karlsruhe, Germany
[c]IRCCS Azienda Ospedaliero-Universitaria di Bologna, via Albertoni 15, 40138 Bologna, Italy

List of common abbreviations

2,4-D 2,4-dichlorophenoxy acetic acid

2-HP-βCD (2-hydroxypropyl)-βCD

2-MBQ 2-mercaptobenzoquinone

2-MHQ 2-mercaptohydroquinone

Å Angstrom

ABA Abscisic acid

Abm abamectinabamectin

AD 1-adamantanamine

AgNPs Silver nanoparticles

AIEgen Aggregation-induced emitting fluorophore

AIETPA aggregation-induced emission-enhancing organic molecules

ALB Albumin

AN (4'-hydroxy-10-methylpyranoflavylium

AuNPs Gold nanoparticles

AVM Avermectin

BA Boronic acid

BSA Bovine serum albumin

C7 Coumarin 7

Cas9 CRISPR associated protein 9

CB*n* Cucurbit[*n*]uril

CBZ Carbendazim

CDots Carbon dots

CDs Cyclodextrins

CHEF Chelation-enhanced fluorescence

CMV Cucumber mosaic virus

CNTs carbon nanotubes

CoPhMoRe Corona phase molecular recognition

CQ Chlormequat

CRISPR Clustered regularly interspaced short palindromic repeats

CuAAC Copper-catalysed azide–alkyne cycloaddition

CX*n* Calix[n]arene

Cy7 Cyanine 7

Cys Cysteine

DCPA Tetrachloroetherphthalate

DD Dodine

DDT 1,1'-(2,2,2-trichloroethane-1,1-diyl)bis(4-chlorobenzene)

DFQ Difenzoquat

DLS Dynamic light scattering

DMSO Dimethyl sulfoxide

DNA Deoxyribonucleic acid

DPU Diphenyl urea

DPV Differential pulse voltammetry

DQ Diquat

dsDNA double-stranded DNA

dsRNA Double-stranded RNA

DTT Dithiothreitol

EC50 Half maximal effective concentration

EFSA European Food Safety Authority

ELISA Enzyme linked Immunosorbent assay

E_{ox} electrochemical oxidation potential

EPA United States Environmental Protection Agency

EtOH Ethanol

FAM Fluorescein

FBZ Fuberidazole

FDA U.S. Food and Drug Administration

FPN Fipronil

FRET Förster resonance energy transfer

FWHM Full width at half maximum

GAs Gibberellins

genFPs Fluorescent proteins

GFP Green fluorescent protein

GRAS Generally recognized as safe

GSH Glutathione

H_2L 4,4′-(propane-2,2-diyl)bis(2-(((2-morpholinoethyl)imino)methyl)phenol)

Hcy Homocysteine

HMS Hollow mesoporous silica nanoparticles

HPLC High-performance liquid chromatography

I Intensity

IC_{50} Half-maximal inhibitory concentration

ICT Intramolecular charge transfer

IMZ Imazilil

IR Infrared

IVN Ivermectin

K_a Binding association constant

kJ Kilojoule

LDA Linear discriminant analysis

LDH Layered double hydroxide

LoD Limit of detection

LoQ Limit of quantification

MES (2-(N-morpholino)ethanesulfonic acid)

miRNA microRNA

MOF Metal-organic framework

MOPS (3-(N-morpholino)propanesulfonic acid)

mPEG monomethyl ether PEG

MQ Mepiquat

mRNA Messenger RNA

MS Mass spectrometry

MSPs Mesoporous silica particles

MV2+ Methyl viologen

NAA 1-naphthalene acetic acid

Nb Nicotiana benthamiana

NIR Near-infrared

Nluc NanoLucTM luciferase

NPs Nanoparticles

PAH poly(allylamine) hydrochloride

PA*n* Pillar[*n*]arene

PB Phosphate buffer

PBS Phosphate-buffered saline

PCR Polymerase chain reaction

pDNA Plasmid DNA

PEG Polyethylene glycole

PEI Polyethylenimine

PFAS Per- and polyfluoroalkyl substances

PFOA Perfluorooctanoic acid

PFOS Perfluorooctane sulfonic acid

pK_a Acid dissociation constant

PLQY Photoluminescence quantum yield

PMMoV Pepper mild mottle virus

PQ Paraquat

Psa Pseudomonas syringae pv. Actinidiae

PVA Polyvinyl alcoho

PVNs Plant-derived virus-like nanoparticles

QDs Quantum dots

RbcS Rubisco small subunit 1A

RCNMV Red clover necrotic mosaic virus

RFD Reference dose

Rh6G Rhodamine 6G

RNA Ribonucleic acid

RNAi RNA interference

ROS Reactive oxygen species

SA Salicylic acid

SC*n* p-sulfonato CX*n*

SERDS Surface-enhanced Raman difference spectroscopy

SERS Surface-enhanced Raman spectroscopy

siRNA Small interfering RNA

Spd Spermidine

SWCNTs Single-walled carbon nanotubes

T Temperature

TA Thioglycolic acid

TBZ Thiabendazole

TCZ Tricyclazole

TDZ Thidiazuron

TEM Transmission electron microscopy

TES 2-{[1,3-Dihydroxy-2-(hydroxymethyl)propan-2-yl]amino}ethane-1-sulfonic acid

TMAPS N-trimethoxysilylpropyl-N,N,N-trimethylammonium chloride

TMGMV Tobacco mild green mosaic virus

Tris Tris(hydroxymethyl)aminomethane

Try Trypsin

UV Ultra-violet

VOC Volatile organic compounds

WHO World Health Organization

WT Wild-type

Xac Xanthomonas axonopodis pv. Citri

Xoo Xanthomonas oryzae pv. Oryzae

ΔG Gibs free energy

ΔH Free enthalpy

ΔS Free entropy

λ_{em} Emission wavelength

λ_{ex} Excitation wavelength
